# Supplementary material for: Effective interventions for improving routine childhood immunisation in low and middle-income countries: a systematic review of systematic reviews
Source: BMJ Open. 2024 Feb 15;14(2):e074370. doi: 10.1136/bmjopen-2023-074370 (PMC10875475; doi:10.1136/bmjopen-2023-074370)
Supplement: Author's manuscript [file bmjopen-2023-074370.draft_revisions.pdf]

BMJ Open is committed to open peer review. As part of this commitment we make the peer review history of every article we publish publicly available.

When an article is published we post the peer reviewers' comments and the authors' responses online. We also post the versions of the paper that were used during peer review. These are the versions that the peer review comments apply to.

The versions of the paper that follow are the versions that were submitted during the peer review process. They are not the versions of record or the final published versions. They should not be cited or distributed as the published version of this manuscript.

BMJ Open is an open access journal and the full, final, typeset and author-corrected version of record of the manuscript is available on our site with no access controls, subscription charges or pay-per-view fees (http://bmjopen.bmj.com).

If you have any questions on BMJ Open's open peer review process please email info.bmjopen@bmj.com

BMJ Open

Effective interventions for improving routine childhood immunisation in low- and middle-income countries: A systematic review of systematic reviews

Journal:	*BMJ Open*
Manuscript ID	bmjopen-2023-074370
Article Type:	Original research
Date Submitted by the Author:	05-Apr-2023

Complete List of Authors:	Jain, Monica; International Initiative for Impact Evaluation Dehli<br>Duvendack, Maren; University of East Anglia<br>Shisler, Shannon; International Initiative for Impact Evaluation<br>Parsekar, Shradha; International Initiative for Impact Evaluation Dehli<br>Leon, Maria Daniela ; International Initiative for Impact Evaluation

Keywords:	Community child health < PAEDIATRICS, Paediatric infectious disease & immunisation < PAEDIATRICS, Health policy < HEALTH SERVICES ADMINISTRATION & MANAGEMENT, Systematic Review

SCHOLARONE™
Manuscripts

I, the Submitting Author has the right to grant and does grant on behalf of all authors of the Work (as defined in the below author licence), an exclusive licence and/or a non-exclusive licence for contributions from authors who are: i) UK Crown employees; ii) where BMJ has agreed a CC-BY licence shall apply, and/or iii) in accordance with the terms applicable for US Federal Government officers or employees acting as part of their official duties; on a worldwide, perpetual, irrevocable, royalty-free basis to BMJ Publishing Group Ltd ("BMJ") its licensees and where the relevant Journal is co-owned by BMJ to the co-owners of the Journal, to publish the Work in this journal and any other BMJ products and to exploit all rights, as set out in our licence.

The Submitting Author accepts and understands that any supply made under these terms is made by BMJ to the Submitting Author unless you are acting as an employee on behalf of your employer or a postgraduate student of an affiliated institution which is paying any applicable article publishing charge ("APC") for Open Access articles. Where the Submitting Author wishes to make the Work available on an Open Access basis (and intends to pay the relevant APC), the terms of reuse of such Open Access shall be governed by a Creative Commons licence – details of these licences and which Creative Commons licence will apply to this Work are set out in our licence referred to above.

Other than as permitted in any relevant BMJ Author's Self Archiving Policies, I confirm this Work has not been accepted for publication elsewhere, is not being considered for publication elsewhere and does not duplicate material already published. I confirm all authors consent to publication of this Work and authorise the granting of this licence.

For peer review only

Effective interventions for improving routine childhood immunisation in low- and middle-income countries: A systematic review of systematic reviews

Monica Jain, Maren Duvendack, Shannon Shisler, Shradha S Parsekar, Maria Daniela Anda Leon

International Initiative for Impact Evaluation (Monica Jain PhD, Shannon Shisler PhD, Shradha S Parsekar MPH and Maria Daniela Anda Leon) and **University of East Anglia, Norwich, UK** (Maren Duvendack PhD)

Correspondence to:

Dr. Monica Jain

Evaluation Office, International Initiative for Impact Evaluation (3ie), New Delhi, 110017, India

Email: mjain@3ieimpact.org

Word count (excluding title page, abstract, strengths and limitations, tables, references, declarations etc.): 3494.

Abstract: 281 words

For peer review only

ABSTRACT

Objective: To provide clarity on the interventions that are effective to improve routine immunisation outcomes for children in low- and middle-income countries (L&MICs).

Design: A systematic review of systematic reviews

Methods: We conducted a comprehensive search. Search results were screened by two reviewers independently applying pre-defined inclusion and exclusion criteria. Data were extracted by two researchers independently. The SURE checklist was used to assess review quality. A mixed-methods synthesis was employed focusing on meta-analytical and narrative elements to accommodate both the quantitative and qualitative information available from the included reviews.

Results: We find caregiver-oriented interventions have large positive and statistically significant effects, especially those focusing on one-time sensitisation and education campaigns as well as written messages to caregivers. For health-systems oriented interventions the evidence base is thin and derived from narrative synthesis suggesting positive effects for home visits, mixed effects for pay for performance schemes and inconclusive effects for contracting out services to non-governmental providers. For all other interventions under this category, the evidence is either limited or not available. For community-oriented interventions, a recent high-quality mixed-methods review suggests positive but small effects. Overall, the evidence base is highly heterogenous in terms of scope, intervention types, and outcomes.

Conclusion: Interventions oriented towards caregivers and communities are effective in improving routine child immunisation outcomes. The evidence base on health-systems oriented interventions is scant not allowing us to reach firm conclusions, except for home visits. Large evidence gaps exist and need to be addressed. For example, more high-quality evidence is needed for specific caregiver-oriented interventions (e.g., monetary incentives) as well as health systems-oriented (e.g., health workers and data systems), and community-oriented

For peer review only - http://bmjopen.bmj.com/site/about/guidelines.xhtml

interventions. We also need to better understand complementarity of different intervention types.

Funding: Bill & Melinda Gates Foundation (OPP1115129).

Keywords: Immunisation; Immunisation Programmes; Mass Vaccination; Children and Adolescents; Poverty Areas

STRENGTHS AND LIMITATIONS OF THIS STUDY

- This is an extensive and novel review that synthesises the evidence base on routine child immunisation interventions in L&MICs at a higher level of abstraction (i.e., at the systematic review of systematic reviews level).
- Our review uses an intervention taxonomy that was developed and refined based on existing literature, extensive discussion, pilot testing, and expert feedback. It allows assessment of interventions oriented towards caregivers, health systems, communities as well as those outside the health realm separately.
- Our review synthesises the evidence both quantitatively through meta-analysis as well as qualitatively. We provide insights into not only where evidence exists but also point out critical evidence synthesis gaps.
- This type of review brings about a wealth of unique methodological challenges such as assessing the quality of studies, grappling with high levels of heterogeneity, lack of detailed descriptions of the intervention types, and wide range of approaches to synthesis.

For peer review only

For peer review only - http://bmjopen.bmj.com/site/about/guidelines.xhtml

INTRODUCTION

In 2019, close to 20 million children in low-and middle-income countries (L&MICs) did not receive the three recommended doses of diphtheria, pertussis and tetanus (DPT) vaccines, often used as an indicator to assess countries' performance on routine immunisation.[1] Ten countries, in particular Nigeria, India, the Democratic Republic of Congo, and Pakistan, account for two out of five unvaccinated children globally.[2] Many L&MICs struggle to achieve high immunisation coverage due to challenges on both the supply and demand side. Examples of supply side constraints include limited availability of health personnel and difficulties in building up their capacity and skills as well as lack of reliable monitoring and surveillance systems to identify and monitor un- and under-vaccinated children.[3] Demand-side issues linked to behavioural, social and practical constraints faced by caregivers also play a role, including concerns about vaccine safety, lack of knowledge of when and where to vaccinate their children, fears about vaccination side effects and difficulties in accessing health services.[4] Governments in L&MICs have strengthened national-level immunisation interventions to address supply side constraints and are increasingly focusing on addressing constraints faced by caregivers and communities. To support policy makers and practitioners in making evidence informed decisions on the interventions that have been effective in addressing the barriers to routine vaccination uptake, it is imperative to provide systematically synthesised evidence.

Several methodologically robust impact evaluations and systematic reviews evaluating the effectiveness of these approaches have been published in the past decade.[5-12] However, there has been no attempt yet to synthesise this extensive evidence base at the meta-level with the exception of Heneghan et al.[13] and Besnier et al.[14] First, Heneghan et al. qualitatively synthesise child and adult immunisation interventions in both L&MICs and high-income countries (HICs) finding inconclusive results for L&MICs due to the small number of reviews for these countries, inadequate definitions of interventions and sub-optimal reporting of

For peer review only - http://bmjopen.bmj.com/site/about/guidelines.xhtml

interventions in the reviews. Second, Besnier et al. examine interventions focusing on improving child health using narrative synthesis tools; they find positive effects of interventions improving immunisation communication, education and social mobilisation on vaccine uptake. While the evidence base on this topic is growing rapidly, Engelbert et al.[15] demonstrate that there are clear evidence gaps preventing us from fully understanding what type of immunisation interventions work best, for whom, how and where. This is the first systematic review of systematic reviews with the primary goal to provide more clarity on the type of interventions that are effective in improving routine immunisation outcomes for children in L&MICs. Establishing what is and is not known about the effectiveness of such interventions is crucially important to inform policymakers and practitioners.

METHODS

Search strategy and selection criteria

This is a systematic review of systematic reviews adopting a mixed-methods approach including a meta-analysis and narrative elements. It draws on the intervention-outcome framework (Appendix 1) developed by Engelbert et al.[15] which distinguishes interventions by different levels, i.e., 1st, 2nd and 3rd tier focusing on the supply side (health-system oriented) as well as the demand side (caregiver-oriented). It also allows for the identification of community-oriented interventions as well as for those which are non-health policy oriented. This framework guides the presentation of our results. In terms of study design, we included systematic reviews with and without meta-analyses. The population of interest included children below the age of five living in L&MICs, but we occasionally included other populations, e.g., caregivers and health workers, especially when these were relevant to understanding the impact of immunisation programmes on intermediate outcomes such as attitudes about vaccination, and access to immunisation services. We included studies examining the impact of any intervention on at least one outcome in relation to routine child

For peer review only

For peer review only - http://bmjopen.bmj.com/site/about/guidelines.xhtml

immunisation. These outcomes are coverage rates or timeliness of full immunisation, third dose of DPT or pentavalent, or measles; additional antigen-specific immunisation coverage outcomes; and intermediate outcomes that precede them in the theoretical causal chain (e.g., attitudes about vaccination, and access to immunisation services). There were no inclusion restrictions by publication status or language, but studies were excluded when they did not meet our definition of systematic reviews, when they were not effectiveness or intervention reviews, or when they only focused on HICs (Appendix 2).

We adopted a comprehensive search strategy that was initially designed for an evidence gap map (EGM)[15] examining the routine child immunisation sector in L&MICs. The search strategy included electronic searches of academic databases and the grey literature, i.e., institutional websites (Appendix 3). The search was completed in October 2021. All search results were screened on title/abstract by a team of trained reviewers. Two reviewers independently screened each abstract. During title and abstract screening, weekly reconciliation meetings were held to discuss and resolve disagreements. Full text screening followed the same approach.

Data analysis

Data were extracted by two researchers independently with reconciliation by others in the review team. Any disagreements were resolved by discussion. We extracted data on context, type of intervention, type of review, design and methods used, outcome measures, quality assessment, study results and findings (Appendix 4). We have only extracted information at the systematic review level.

Non-independence of reviews (or overlap)

Non-independence of reviews (or overlap) is an issue unique to systematic reviews of systematic reviews. Overlap explores to what extent the primary studies included in the pool of systematic reviews are the same or different. We assessed overlap by compiling a citation

For peer review only - http://bmjopen.bmj.com/site/about/guidelines.xhtml

matrix which includes all the primary studies (one per row) included in the individual systematic reviews (one per column). Primary studies were sorted alphabetically, and duplicates removed. The primary studies that were the same across reviews were ticked with a check mark. This allowed us to calculate the corrected covered area (CCA) index that describes the extent of overlap in % terms.[16, 17]

$$CCA = \frac{N - r}{(rxc) - r}$$

Where N is the total number of primary studies included in the systematic reviews (the sum of ticked boxes in the citation matrix), r is the number of rows (the primary studies), and c is the number of columns (included systematic reviews). Pieper et al.[17] provide criteria for the interpretation of CCA, where 0-5% suggests slight overlap, 6-10% moderate overlap, 11-15% high overlap and >15% very high overlap. Hennessy and Johnson[16] recommend further overlap investigations in case of a heterogenous evidence base, hence we compare sub-clusters of reviews examining similar outcomes as well.

Assessment of risk of bias in included studies

The quality of systematic reviews is assessed differently compared to assessing the quality of the primary studies included in systematic reviews, this is due to the unique methodological features of systematic reviews. The quality of the included reviews was assessed independently by two researchers using the checklist developed by the Specialist Unit for Review Evidence (SURE) to allow a critical appraisal to ensure that minimum levels of methodological rigour are met. The SURE[18] checklist was slightly adapted (Appendix 5).

We also extracted information on the quality assessment tools reported in each included review which have been used to assess the quality of the underlying primary evidence base by the reviews.

For peer review only

Data synthesis

We adopted a mixed-methods synthesis approach to best accommodate both the quantitative and qualitative information available. To synthesise the quantitative data, we implemented a robust variance estimation (RVE) meta-regression approach[19] as this performs well even when study numbers are as low as 10.[20]

When an RVE meta-regression approach was not possible, we fitted a random-effects model to the data. In this case, the amount of heterogeneity (i.e., τ^2), was estimated using the DerSimonian-Laird estimator.[21] In addition, we also reported the Q-test for heterogeneity[22] and the I^2 statistic.[23] Studentized residuals and Cook's distances were used to examine whether studies may be outliers and/or influential in the context of the model.[24] Studies with a studentized residual larger than the $100\times(1\text{-}0.05/(2\times k))^{th}$ percentile of a standard normal distribution were considered potential outliers (i.e., using a Bonferroni correction with two-sided $\alpha = 0.05$ for k studies included in the meta-analysis). Studies with a Cook's distance larger than the median plus six times the interquartile range of the Cook's distances were considered to be influential. The rank correlation test[25] and the regression test,[26] using the standard error of the observed outcomes as predictor, were used to check for funnel plot asymmetry but only when we had more than 10 studies.[27]

Risk ratios (RRs) were the most commonly reported effect size, and thus our metric of choice. Odds ratios (ORs) were converted to RRs using the following formula:[28]

$$RR = OR / (1 - p + (p \text{ x } OR))$$

Where p is the risk in the control group implying that ORs can only be converted to RRs when the risk in the control group is known. As this is not always the case, we established a range of plausible risks for the control group (Grant, 2014) drawing on data from Jain et al.[29, 30] on baseline full immunisation rates to find the mean (0·48) and standard deviation (SD) (0·22) of the data to be able to convert ORs to RRs. We did these conversions 3 times as a sensitivity

For peer review only - http://bmjopen.bmj.com/site/about/guidelines.xhtml

analysis. First, we used 1 SD below the mean to establish the control group rate (0.26 – the least conservative model), second, we used the mean as the control group rate (0·48 – the baseline model), and finally, we used 1 SD above the mean (0·70 – the conservative model) as the control group rate.

Patient and public involvement

No patients have been involved in this study as it is a review article, hence no patient consent for publication was required.

Role of the funding source

The funder of the study had no role in the study design, data collection, data analysis, data interpretation, or writing of the report.

RESULTS AND DISCUSSION

Our search builds on Engelbert et al.[15] who had included 58 systematic reviews in their EGM, which we screened against our inclusion criteria. Six of these reviews did not meet our criteria for inclusion and were thus excluded leaving us with 52 studies. We ran additional searches (Appendix 3); the electronic search led to 1,687 records of which 818 were duplicates leaving 869 for screening. We excluded 823 records based on title and abstract screening. The remaining 46 studies were screened by full-text, 39 were excluded, leaving seven new studies for inclusion derived from the electronic search. We identified a further seven records searching the grey literature which all required full-text screening. Four were subsequently excluded leaving three new studies for inclusion (Figure 1). This two-pronged search strategy led to 10 new systematic reviews to be included. Overall, we included a total of 62 systematic reviews - 52 studies from the EGM by Engelbert et al.[15] and 10 new studies from the updated search A summary of the included systematic reviews can be found in Appendix 6.

< Place Figure 1 here >

For peer review only - http://bmjopen.bmj.com/site/about/guidelines.xhtml

Overlap

We test overlap by compiling a citation matrix listing all primary studies which is used to calculate the CCA. Table 1 suggests limited overlap of 0.5% confirming our initial investigations. This is confirmed by Pieper et al.[17] who provide guidance on the interpretation of CCA values, e.g., with values between 0-5% indicating slight overlap.

Table 1: Overlap

	Times studies appeared in reviews	Number of rows	Number of reviews	CCA values	
	N	*r*	*c*	Proportion	Percentage
Overall	1428	1079	62	0·0053	0·5

We compare sub-clusters of reviews examining similar outcomes as well finding limited overlap for most outcomes, e.g., for measles, full routine immunisation for children, DPT3 and vaccination coverage, the CCA values range from 0·7 to 2·7%. Vaccination timeliness has the highest CCA value (6·9%) indicating moderate levels of overlap.[17]

Risk of Bias

Using the SURE checklist, we find that of the 62 included reviews, 18 are categorised as high confidence, six are of medium confidence and 38 are of low confidence (Appendix 7).

For peer review only - http://bmjopen.bmj.com/site/about/guidelines.xhtml

Effectiveness of interventions

Table 2: Summary of findings

Intervention (1st, 2nd, 3rd tier)			# SRs	SRs (Low confidence SRs in parenthesis)	Evidence as reported in the high & medium confidence SRs: Quantitative findings	Evidence as reported in the high & medium confidence SRs: Qualitative findings	Quantitative findings from RoR	Overall findings**
A. Caregiver-oriented interventions (1st tier)	AA. Information & education (2nd tier)	AA1. Sustained sensitisation and education campaigns (3rd tier)	1	(Ryman et al., 2008[31])	None	None	None	None (Low confidence evidence)
		AA2. One-time sensitisation and education campaigns (3rd tier)	12	Kaufman et al., 2018; Gilmore & McAuliffe, 2013; Johri et al., 2015; Lukusa et al., 2018; Oyo-Ita et al., 2016; Saeterdal et al., 2014[6, 8, 9, 11, 12, 32] (Crocker-Buque et al., 2017;	Seven pooled estimates are available from 4 SRs; of which 6 were positive and statistically significant. RR ranged between 1·2 and 1·68.	Most SRs included small number of primary studies and reported favourable effects of AA2 intervention on range of outcomes: DPT3, Measles, Vaccination coverage (unspecified) and	The average RR was 1·38 (95% CI: 1·33 to 1·44). Refer to Figure 2	Positive and statistically significant

For peer review only - http://bmjopen.bmj.com/site/about/guidelines.xhtml

Intervention (1st, 2nd, 3rd tier)			# SRs	SRs (Low confidence SRs in parenthesis)	Evidence as reported in the high & medium confidence SRs		Quantitative findings from RoR	Overall findings**
					Quantitative findings	Qualitative findings		
				Deardorff et al., 2018; Jarette et al., 2015; Kim et al., 2017; Pinzón-Flórez et al., 2015; Shea et al., 2009[33-38])		other secondary outcomes.		
		AA3. Public information campaigns (3rd tier)	3	(Naugle & Hornik, 2014; Shea et al. 2009; Yuan et al. 2014[38-40])	None	None	None	None (Low confidence evidence)

For peer review only - http://bmjopen.bmj.com/site/about/guidelines.xhtml

Intervention (1st, 2nd, 3rd tier)			# SRs	SRs (Low confidence SRs in parenthesis)	Evidence as reported in the high & medium confidence SRs: Quantitative findings	Evidence as reported in the high & medium confidence SRs: Qualitative findings	Quantitative findings from RoR	Overall findings**
	AB. Incentives &	AB1. Material/ monetary incentives for caregivers (3rd tier)	12	Bassani et al., 2013; Lagarde et al., 2009; Oyo-Ita et al., 2016[11, 41, 42] (Bright et al., 2017; Crocker-Buque et al., 2017; de Souza Cruz et al., 2017; Jarette et al., 2015; Onwuchekwa et al., 2021; Omoniyi 2020; Owusu-Addo & Cross, 2014; Pal et al., 2016; Ranganathan &	Meta-analysis was done by two SRs, both found statistically insignificant results (RR: 1·05 [0·90, 1·23], SMD ranging from 0·05 to 0·06).	Most outcomes were narratively synthesised with small number of primary studies, suggesting either inconclusive or favourable effect.	MA was not feasible.	Mixed effects

For peer review only - http://bmjopen.bmj.com/site/about/guidelines.xhtml

Intervention (1st, 2nd, 3rd tier)			# SRs	SRs (Low confidence SRs in parenthesis)	Evidence as reported in the high & medium confidence SRs		Quantitative findings from RoR	Overall findings**
					Quantitative findings	Qualitative findings		
				Lagarde, 2012[33, 35, 43-49])				
		AB2. Non-material incentives for caregivers (3rd tier)	0	NA	None	None	None	No evidence
		AB3. Automated voice messages to caregivers (3rd tier)	1	(Yunusa et al., 2021[50])	None	None	None	None (Low confidence evidence)

For peer review only - http://bmjopen.bmj.com/site/about/guidelines.xhtml

For peer review only

Intervention (1st, 2nd, 3rd tier)			# SRs	SRs (Low confidence SRs in parenthesis)	Evidence as reported in the high & medium confidence SRs		Quantitative findings from RoR	Overall findings**
					Quantitative findings	Qualitative findings		
		AB4. Written or pictorial messages (SMS, stickers, flyers etc.) to caregivers (3rd tier)	13	Atkinson et al., 2019; Chutiyami et al., 2019; Eze et al., 2021; Linde et al., 2019; Mekonnen et al., 2019; Palmer et al., 2020[5, 10, 51-54] (Baequni, 2012; Crocker-Buque et al., 2017; Jarette et al., 2015; Karageorgos et al., 2019; Kim et al., 2017; Omoniyi & Williams, 2020;	Meta-analysis was done by all high & medium confidence SRs reporting nine estimates for a range of outcomes, all found positive and statistically significant effects. RR or OR ranged between 1·13 and 3·69.	Intervention was found to be effective in improving Vaccination coverage (unspecified), DPT 3, Full routine immunization, and Vaccination timeliness.	RR of 1·24 (95% CI: 1·11 to 1·36). Refer to Figure 3	Positive and statistically significant

For peer review only - http://bmjopen.bmj.com/site/about/guidelines.xhtml

Intervention (1st, 2nd, 3rd tier)			# SRs	SRs (Low confidence SRs in parenthesis)	Evidence as reported in the high & medium confidence SRs		Quantitative findings from RoR	Overall findings**
					Quantitative findings	Qualitative findings		
				Yunusa et al., 2021[33, 35, 36, 45, 50, 55, 56])				
		AB5. Changes to health system user fees (3rd tier)	1	(Giedion et al., 2013[57])	None	None	None	None (Low confidence evidence)
B. Health system-oriented (1st tier)	BA. Education & training (2nd tier)	BA1. Formal health worker training and education (3rd tier)	3	Oyo-Ita et al., 2016[11] (Jarette et al., 2015; Pal et al., 2016[35, 48])	None	Inconclusive effect on outcomes based on single primary study.	None	Limited evidence

For peer review only - http://bmjopen.bmj.com/site/about/guidelines.xhtml

Intervention (1st, 2nd, 3rd tier)			# SRs	SRs (Low confidence SRs in parenthesis)	Evidence as reported in the high & medium confidence SRs		Quantitative findings from RoR	Overall findings**
					Quantitative findings	Qualitative findings		
		BA2. Community health worker training and education (3rd tier)	0	None	None	None	None	No evidence
	BB. Planning, implementation, monitoring (2nd tier)	BB1. Formal health worker involvement in planning & monitoring (3rd tier)	0	None	None	None	None	No evidence
		BB2. Community health worker involvement in planning & monitoring (3rd tier)	0	None	None	None	None	No evidence
		BB3. Paper-based tracking (3rd tier)	0	None	None	None	None	No evidence
		BB4. Promoting outreach to vaccine-hesitant groups (3rd tier)	0	None	None	None	None	No evidence

For peer review only - http://bmjopen.bmj.com/site/about/guidelines.xhtml

For peer review only

Intervention (1st, 2nd, 3rd tier)			# SRs	SRs (Low confidence SRs in parenthesis)	Evidence as reported in the high & medium confidence SRs		Quantitative findings from RoR	Overall findings**
					Quantitative findings	Qualitative findings		
		BB5. Outreach to vulnerable populations (hard-to-reach, SES, caste, etc.) (3rd tier)	3	Oyo-Ita et al., 2016[11] (Crocker-Buque et al., 2017; Yuan et al., 2014[33, 40])	None	Low certainty favourable effect on the outcome. However, the results are based on a single primary study.	None	Limited evidence
		BB6. Outreach to migrant populations (3rd tier)	0	None	None	None	None	No evidence
		BB7. Home visits (3rd tier)	5	Glenton et al., 2011; Oyo-Ita et al., 2016[11, 58] (Freeman et al., 2017; Rahman et al., 2013; Shea et al., 2009[38, 59, 60])	None	Favourable effect of intervention on OPV3, measles and full routine immunisation but the results were based on a single primary study for each outcome.	None	Positive

For peer review only - http://bmjopen.bmj.com/site/about/guidelines.xhtml

Intervention (1st, 2nd, 3rd tier)			# SRs	SRs (Low confidence SRs in parenthesis)	Evidence as reported in the high & medium confidence SRs		Quantitative findings from RoR	Overall findings**
					Quantitative findings	Qualitative findings		
		BB8. Campaigns to vaccinate refugee populations (3rd tier)	0	None	None	None	None	No evidence
	BC. Supplementary Immunisation Activities (2nd tier)	BC1. National/sub-national immunisation days (3rd tier)	2	(Deardoff 2018; Freeman et al., 2017[34, 59])	None	None	None	None (Low confidence evidence)
	BD. Incentives & motivation (2nd tier)	BD1. Material/monetary incentives for health workers (3rd tier)	1	(Shea et al., 2009[38])	None	None	None	None (Low confidence evidence)
		BD2. Non-material incentives for health workers (3rd tier)	0	None	None	None	None	No evidence
		BD3. Automated voice messages to health workers (3rd tier)	0	None	None	None	None	No evidence

For peer review only

For peer review only - http://bmjopen.bmj.com/site/about/guidelines.xhtml

Intervention (1st, 2nd, 3rd tier)			# SRs	SRs (Low confidence SRs in parenthesis)	Evidence as reported in the high & medium confidence SRs		Quantitative findings from RoR	Overall findings**
					Quantitative findings	Qualitative findings		
		BD4. Written or pictorial messages (SMS, stickers, flyers etc.) to health workers (3rd tier)	1	(Bright et al., 2017[43])	None	None	None	None (Low confidence evidence)

For peer review only - http://bmjopen.bmj.com/site/about/guidelines.xhtml

Intervention (1st, 2nd, 3rd tier)			# SRs	SRs (Low confidence SRs in parenthesis)	Evidence as reported in the high & medium confidence SRs		Quantitative findings from RoR	Overall findings**
					Quantitative findings	Qualitative findings		
		BD5. Pay-for-performance schemes (3rd tier)	5	Diaconu et al., 2021; Witter et al., 2012[7, 61] (Bright et al., 2017; Das et al., 2016; Parrado-Jiménez, 2013[43, 62, 63])	None	For most vaccination coverage related outcomes, the effect was inconclusive (low certainty evidence). While, for some of the intermediate outcomes it was positive and statistically significant.<br>As per review authors, the aforesaid findings could be because of diversity in these schemes and the	None	Mixed effects

For peer review only - http://bmjopen.bmj.com/site/about/guidelines.xhtml

Intervention (1st, 2nd, 3rd tier)			# SRs	SRs (Low confidence SRs in parenthesis)	Evidence as reported in the high & medium confidence SRs		Quantitative findings from RoR	Overall findings**
					Quantitative findings	Qualitative findings		
						differences in outcome measures by the primary studies.		
	BE. Infrastructure (2nd tier)	BE1. Building & upgrading health clinics (3rd tier)	0	None	None	None	None	No evidence
		BE2. Cold chain infrastructure improvements (3rd tier)	0	None	None	None	None	No evidence

For peer review only - http://bmjopen.bmj.com/site/about/guidelines.xhtml

Intervention (1st, 2nd, 3rd tier)			# SRs	SRs (Low confidence SRs in parenthesis)	Evidence as reported in the high & medium confidence SRs		Quantitative findings from RoR	Overall findings**
					Quantitative findings	Qualitative findings		
	BF. Health system governance, policies and financing (2nd tier)	BF1. Health system strategic planning (3rd tier)	7	Oyo-Ita et al., 2016[11] (Bright et al., 2017; Nelson et al., 2016; Nutman et al., 2013; Omoniyi & Williams, 2020; Smith et al., 2018; Yuan et al., 2014[40, 43, 45, 64-66])	None	Inconclusive results for most outcomes, from a single primary study.	None	Limited evidence
		BF2. Vaccination guidelines (3rd tier)	0	None	None	None	None	No evidence
		BF3. Changes to broader governance systems (beyond health systems) (3rd tier)	0	None	None	None	None	No evidence

For peer review only - http://bmjopen.bmj.com/site/about/guidelines.xhtml

For peer review only

**Intervention (1st, 2nd, 3rd tier)**			**# SRs**	**SRs (Low confidence SRs in parenthesis)**	**Evidence as reported in the high & medium confidence SRs: Quantitative findings**	**Evidence as reported in the high & medium confidence SRs: Qualitative findings**	**Quantitative findings from RoR**	**Overall findings****
		BF4. Health system financing (3rd tier)	3	Lagarde & Palmer 2009; Odendaal et al., 2018[67, 68] (Loevinsohn & Harding, 2004[69])	None	Inconclusive results for a range of outcomes. The results are based on one or two primary studies for each outcome.	None	Inconclusive
	BG. Technology & mHealth (2nd tier)	BG1. New HMIS/Dashboard systems (incl. improved data collection) (3rd tier)	3	(de Cock et al., 2020; Kim et al., 2017; Watterson et al., 2015[36, 70, 71])	None	None	None	None (Low confidence evidence)
		BG2. Capacity building (e.g., training) for existing systems (3rd tier)	0	None	None	None	None	No evidence

For peer review only - http://bmjopen.bmj.com/site/about/guidelines.xhtml

Intervention (1st, 2nd, 3rd tier)			# SRs	SRs (Low confidence SRs in parenthesis)	Evidence as reported in the high & medium confidence SRs		Quantitative findings from RoR	Overall findings**
					Quantitative findings	Qualitative findings		
C. Other community member-oriented (1st tier)	CA. Other community member- oriented (2nd tier)	CA1. Faith-based outreach/outreach using local leaders (3rd tier)	1	(Jarrette et al., 2015[35])	None	None	None	None (Low confidence evidence)
D. Community-level (1st tier)	DA. Communication & dialogue (2nd tier)	DA1. Collaborating with whole community (3rd tier)	1	Molina et al., 2016[72]	None	Inconclusive results based on a single primary study.	None	Limited evidence
		DA2. Collaborating with selected community groups and networks (3rd tier)	1	Desai et al., 2020[73]	None	Small and statistically insignificant result based on two primary studies.	None	Limited evidence
	DB. Tracking & registering (2nd tier)	DB1. Community tracking and registering (3rd tier)	0	None	None	None	None	No evidence

For peer review only - http://bmjopen.bmj.com/site/about/guidelines.xhtml

Intervention (1st, 2nd, 3rd tier)			# SRs	SRs (Low confidence SRs in parenthesis)	Evidence as reported in the high & medium confidence SRs		Quantitative findings from RoR	Overall findings**
					Quantitative findings	Qualitative findings		
E. Policies and institutions (1st tier)	EA. Education policy and infrastructure (2nd tier)	EA1. Education policy and infrastructure (3rd tier)	0	None	None	None	None	No evidence
	EB. Non-health/ education infrastructure (2nd tier)	EB1. Non-health/ education infrastructure (e.g., electrification) (3rd tier)	0	None	None	None	None	No evidence

For peer review only - http://bmjopen.bmj.com/site/about/guidelines.xhtml

Intervention (1st, 2nd, 3rd tier)	# SRs	SRs (Low confidence SRs in parenthesis)	Evidence as reported in the high & medium confidence SRs		Quantitative findings from RoR	Overall findings**
			Quantitative findings	Qualitative findings		
F. Multicomponent (1st tier)	1*	Jain et al., 2022[29, 30]	Meta-analysis done on a range of outcomes and found positive and statistically significant effect on most of the outcomes. The SMD ranged between 0·06 and 0·24	None	None	Positive and statistically significant

Notes: *Apart from Jain et al.[29, 30], there were 25 systematic reviews that assessed the effects of more than one intervention that are not part of this table. Of these reviews, the majority (18) were low confidence SRs and the intervention components were heterogenous. We provide summary of Jain et al.[29, 30] in this table because it is one of the only included SRs that assessed community engagement interventions. Intervention and outcome codes are taken from Appendix 1.

** The last column, 'overall findings' are summarized from previous three columns i.e., evidence as reported in the systematic reviews, qualitative and quantitative findings and quantitative findings from review of reviews (RoR).

For peer review only - http://bmjopen.bmj.com/site/about/guidelines.xhtml

For peer review only

For peer review only - http://bmjopen.bmj.com/site/about/guidelines.xhtml

< Place Figure 2 here >

<Place Figure 3 here >

Overall, the evidence base is highly heterogenous in terms of scope, intervention types and outcomes. Table 2 summarises the key findings across all high and medium confidence reviews across all intervention categories. Most of the evidence we uncovered is centred on caregiver-oriented interventions (A) that have positive and statistically significant effects at the 1st and 2nd tier analysis level (Appendices 8 and 9 for findings with details on intervention coding and level of analysis in Table 2). At the 3rd tier level, our analysis confirms the favourable trends emerging from the 1st and 2nd tier analyses. For one-time sensitisation and education campaigns (AA2 – Figure 2), we find that the average RR was 1·38 (95% CI: 1·33 to 1·44) suggesting that the average outcome differed significantly from zero (z = 47·37, p < 0·001). As for written or pictorial messages (SMS, stickers, flyers etc.) to caregivers (AB4 – Figure 3), we find that the average RR of the baseline model stands at 1.24 ([95% CI: 1·11 to 1·36], p < 0·001). Overall, 3rd tier intervention categories are reporting positive and statistically significant effects, which suggest that treated children are more likely to be vaccinated than untreated children. Another intervention category at the 3rd tier for which there is a large evidence base is monetary incentives (AB1)[1] and the evidence is mixed.

For the health systems-oriented interventions (B) we had no quantitative evidence, thus we could only rely on narrative synthesis and the results are mixed (Appendix 10). Depending on the exact nature of the intervention, the contextual background and the specific outcome, the findings range from favourable to inconclusive effects. Among these interventions, the only intervention category at the 3rd tier for which we find positive effects are home visits (BB7).

[1] The evidence on monetary incentives (AB1) interventions is dominated by qualitative narrative methods. There are only two reviews with quantitative evidence, but we could not use them for quantitative synthesis as we were not able to convert effect sizes from SMD to RR.

For peer review only - http://bmjopen.bmj.com/site/about/guidelines.xhtml

We find mixed results for pay for performance schemes (BD5) which have a large evidence base both at the primary study and review level. Additionally, we find inconclusive results based on a thin evidence base for health systems financing (BF4) interventions in which health services are contracted out to non-governmental providers and they are compensated for it. For a 3rd tier category like health system strategic planning (BF1), though the evidence base is large when it occurs in combination with other interventions, i.e., as multicomponent interventions, the evidence base for single interventions is rather limited and inconclusive.

As for community-oriented interventions (C and D), the evidence base is very limited, focused on narrative synthesis and finding inconclusive results (Appendix 11). However, a recent review[29, 30] on single and multicomponent community engagement interventions (F) uses a nuanced framework to classify them based on the process of engaging communities and finds them to be effective using meta-analytical methods. We found no evidence on interventions related to non-health related policies and institutions (E).

This review also assessed the interventions aiming to improve zero dose outcomes through DPT1 and BCG vaccine uptake. We find 4 reviews suggesting inconclusive evidence for interventions improving DPT1 outcomes. However, for BCG vaccine uptake we find pay for performance schemes (BD5 – 1 qualitative review) and community engagement interventions (C and D – 1 quantitative review) to be effective.

Strengths and weaknesses

We have contributed an extensive and novel study that synthesises the evidence base on routine child immunisation interventions in L&MICs at a higher level of abstraction (i.e., at the systematic review of systematic reviews level) and this brings about a wealth of unique methodological challenges such as assessing the quality of studies, grappling with high levels of heterogeneity, the lack of detailed descriptions of the intervention types and the wide range

For peer review only - http://bmjopen.bmj.com/site/about/guidelines.xhtml

of approaches to synthesis. We have dealt with these challenges following guidance provided by the Cochrane and Campbell Collaborations.

Implications for policy and practice

We find caregiver-oriented interventions to be effective, especially one-time sensitisation and education campaigns (AA2), and written messages to caregivers (AB4). Sensitisation and education campaigns (AA2) mostly address the knowledge gaps of caregivers on the importance of vaccinations, of maintaining its schedule and potential misconceptions around vaccinations. This information is delivered by either frontline health workers or trained facilitators at the health facility, at caregiver homes or in community groups. The SMS reminders (AB4) delivered to the mobile phones of caregivers address the practical barriers that they face regarding when and where to take their child for a vaccine or follow-up doses. These interventions can be effective in improving vaccine uptake in communities where such barriers are prevalent.

In the last decade, a lot of the attention of researchers, programme implementers and policymakers has been on how to motivate caregivers to vaccinate their children through monetary incentives and there have been a substantial number of experiments involving conditional and/or unconditional cash transfer schemes to assess their effectiveness in improving immunisation outcomes.[15] However, the evidence does not provide a clear answer on their effectiveness and there is a need for an updated review with a more inclusive search strategy for identifying relevant articles published in non-health journals.

Among the health-system oriented interventions, home visits (BB7) are worth considering for improvement in vaccine uptake as we find them to be effective based on narrative evidence, however, the evidence base is thin. As for pay-for-performance schemes (BD5) we find both positive and inconclusive results depending on the outcome of interest. As a recent review[7]

For peer review only - http://bmjopen.bmj.com/site/about/guidelines.xhtml

is available and of high quality, there is no need for an update and policymakers can consult it for more details. The thin evidence does not seem to support effectiveness of health financing (BF4) interventions in which health services are contracted out to non-governmental providers.

For interventions focusing on formal health worker training and education (BA1), we found only one review of reasonable quality; the narrative evidence is mixed with both positive and inconclusive results depending on the outcome of interest. Engelbert et al.[15] found a substantial number of primary studies on this intervention (BA1) and on interventions focusing on incentives and motivation of health workers (BD1), though most were combined with other intervention components. As capacity, motivation and skills of frontline health providers can be critical factors in accessing immunisation services as well as motivating caregivers, a review of the interventions targeting them and exploring their complementarities with other intervention components can be an important contribution to the future evidence base.

For interventions categorised as outreach to vulnerable populations (BB6) and health system strategic planning (BF1), we found limited and inconclusive evidence based on a small number of primary studies assessing impact across heterogenous outcomes. For other sub-categories of interventions oriented towards health systems, we found no evidence or only low-quality evidence. There are some interventions, like the new HMIS/dashboards systems (BG1), for which Engelbert et al.[15] found a substantial number of primary studies, though most of these interventions were combined with other intervention components, but no systematic review was found. As these interventions leveraging digital technology are increasingly being adopted and expanded, a related review would fill a critical evidence gap.

While the evidence on health-system oriented interventions in the context of immunisation is limited, policymakers seeking to strengthen health systems for the purpose of improving immunisation outcomes can consult the broader literature on health system strengthening for

For peer review only - http://bmjopen.bmj.com/site/about/guidelines.xhtml

guidance. In fact, there is a systematic review of reviews available,[74] which synthesised evidence that assessed the effects of health systems strengthening interventions on health status and health system outcomes (service utilisation, quality service provision, uptake of healthy behaviours, and financial protection) in L&MICs, which may be helpful depending on its quality.

For guidance on effectiveness of community-oriented interventions, the recent review by Jain et al.[29, 30] can be consulted as it is comprehensive, provides quantitative evidence and uses a nuanced framework to classify community-oriented interventions based on the process of engaging communities.

Implications for research

Besides addressing critical evidence gaps as described in the section above, we also need to better understand complementarity of interventions, e.g., some reviews analysed combinations of caregiver and health-systems oriented intervention types (bridging the demand and supply side gaps) finding favourable but also inconclusive effects. We do not yet know which intervention combinations work best in terms of improving immunisation outcomes. Further theory development may be a starting point for contributing to a better understanding of the enablers and barriers of interventions as well as unpacking underlying causal mechanisms and thus improving the selection and targeting of immunisation programmes.

In addition, future research needs to engage more with cost effectiveness of interventions as we need to not only learn about what works but also at what cost to enable selection of not only the most impactful but also most cost-effective interventions to improve routine child immunisation outcomes. Further work is also needed in improving the quality of both systematic reviews and primary studies. At the review level future research should attempt more subgroup analyses by levels of confidence in primary studies to instil more trust in the

For peer review only - http://bmjopen.bmj.com/site/about/guidelines.xhtml

results. At the primary study level, researchers should use more robust evaluation methods to minimise the potential biases in the measurement of effects. More work is required to develop user friendly quality assessment tools for systematic reviewers that minimise the scope for subjective judgements.

Author contributors

MJ conceived the review, MJ, SS and MD wrote the protocol. SP and DA did the systematic search. SP and DA screened and identified studies and MJ and MD made final decisions regarding study inclusion. SP and DA did the data extraction and critical appraisal. SS, DA and MD did the statistical analysis. MD and SP did the qualitative analysis. MD and MJ wrote the manuscript. MJ provided critical inputs on the whole analysis, checked data, coordinated the review and had full access to all materials and results. All authors critically reviewed and revised the manuscript and approved the final document for submission. MJ is responsible for the overall content and is the guarantor. All authors attest they meet the ICMJE criteria for authorship.

Declaration of interests

This research was funded through a grant from the Bill & Melinda Gates Foundation (OPP1115129) to the International Initiative for Impact Evaluation (3ie). Through this grant the following work has been commissioned: a) 3ie provided funding and technical assistance for seven impact evaluations of community engagement interventions for immunisation, b) 3ie conducted a systematic review of community engagement interventions to assess their effectiveness in improving routine child immunisation in L&MICs; and c) 3ie conducted an evidence gap-map on routine child immunisation in L&MICs. As members of 3ie staff, author MJ had been involved in reviewing deliverables for the evaluations funded by 3ie and providing research teams with technical assistance. MJ, SS, DA & SP have also worked as

For peer review only - http://bmjopen.bmj.com/site/about/guidelines.xhtml

For peer review only

authors on the systematic review[29, 30] and MJ and SP have been co-authors of the EGM report[15, 75]. Among these, only the work on the systematic review could pose a potential conflict of interest. However, several procedural safeguards and transparency measures were put in place to mitigate the risk this conflict of interest imposed. All candidate systematic reviews, including the one carried out by 3ie, have undergone a rigorous screening and critical appraisal process by two independent screeners. The authors have no financial interest in this area. The remaining study author MD has authored systematic reviews and systematic reviews of reviews but largely on issues related to financial inclusion. MD is also an editor of the International Development Coordinating Group at the Campbell Collaboration, but this will not pose any conflicts of interest.

Role of the funding source

The funder of the study had no role in study design, data collection, data analysis, data interpretation, or writing of the report.

Data sharing

The systematic reviews and other documents included in this study are public and available. The data extracted from these studies are available from the corresponding author upon request.

Acknowledgements

This research has been undertaken as a part of 3ie's immunisation evidence programme, supported by the Bill and Melinda Gates Foundation, Seattle, USA. We would like to thank Sohail Agha, previous Senior Program Officer, Gates Foundation, Seattle, for his support in conceptualisation as well as engagement on the review. Special thanks to Tove Ryman, Senior Program Officer, Gates Foundation, Seattle, whose guidance helped shape the analysis and dissemination of this review. We would like to further thank Mark Engelbert who was

For peer review only - http://bmjopen.bmj.com/site/about/guidelines.xhtml
For peer review only

instrumental in guiding the search process and Avantika Bagai who provided support in the initial phase of the review. We would also like to thank Birte Sniltsveit at 3ie for her comments on this review and for Sebastian Martinez at 3ie for his overall support of the review.

REFERENCES

1. WHO. Progress and Challenges with Achieving Universal Immunization Coverage: 2019 WHO/UNICEF Estimates of National Immunization Coverage Geneva: World Health Organization; 2019 [Available from: https://www.who.int/publications/m/item/progress-and-challenges-with-achievinguniversal-immunization-coverage.

2. WHO. Immunization coverage: Key facts Geneva: World Health Organization; 2021 [Available from: https://www.who.int/news-room/fact-sheets/detail/immunization-coverage.

3. WHO. Immunization Coverage Geneva: World Health Organization; 2018 [Available from: https://www.who.int/news-room/fact-sheets/detail/immunization-coverage.

4. Harvey H, Reissland N, Mason J. Parental reminder, recall and educational interventions to improve early childhood immunisation uptake: A systematic review and meta-analysis. Vaccine. 2015;33(25):2862-80.

5. Atkinson KM, Wilson K, Murphy MSQ, El-Halabi S, Kahale LA, Laflamme LL, et al. Effectiveness of digital technologies at improving vaccine uptake and series completion - A systematic review and meta-analysis of randomized controlled trials. Vaccine. 2019;37(23):3050-60.

6. Lukusa LA, Ndze VN, Mbeye NM, Wiysonge CS. A systematic review and meta-analysis of the effects of educating parents on the benefits and schedules of childhood vaccinations in low and middle-income countries. Human vaccines & immunotherapeutics. 2018;14(8):2058-68.

For peer review only - http://bmjopen.bmj.com/site/about/guidelines.xhtml

7. Diaconu K, Falconer J, Verbel A, Fretheim A, Witter S. Paying for performance to improve the delivery of health interventions in low- and middle-income countries. The Cochrane database of systematic reviews. 2021;5(5):Cd007899.

8. Johri M, Pérez MC, Arsenault C, Sharma JK, Pai NP, Pahwa S, et al. Strategies to increase the demand for childhood vaccination in low- and middle-income countries: a systematic review and meta-analysis. Bulletin of the World Health Organization. 2015;93(5):339-46c.

9. Kaufman J, Ryan R, Walsh L, Horey D, Leask J, Robinson P, et al. Face-to-face interventions for informing or educating parents about early childhood vaccination. The Cochrane database of systematic reviews. 2018;5(5):Cd010038.

10. Mekonnen ZA, Gelaye KA, Were MC, Gashu KD, Tilahun BC. Effect of mobile text message reminders on routine childhood vaccination: a systematic review and meta-analysis. Systematic reviews. 2019;8(1):154.

11. Oyo-Ita A, Wiysonge CS, Oringanje C, Nwachukwu CE, Oduwole O, Meremikwu MM. Interventions for improving coverage of childhood immunisation in low- and middle-income countries. The Cochrane database of systematic reviews. 2016;7(7):Cd008145.

12. Saeterdal I, Lewin S, Austvoll-Dahlgren A, Glenton C, Munabi-Babigumira S. Interventions aimed at communities to inform and/or educate about early childhood vaccination. The Cochrane database of systematic reviews. 2014(11):Cd010232.

13. Heneghan C, Plüddemann A, Spencer E, Brassey J, Rosca E, Onakpoya I, et al. Interventions designed to improve vaccination uptake: Scoping review of systematic reviews and meta-analyses - (version 1). medRxiv. 2022:01.26.22269890.

14. Besnier E, Thomson K, Stonkute D, Mohammad T, Akhter N, Todd A, et al. Which public health interventions are effective in reducing morbidity, mortality and health inequalities from infectious diseases amongst children in low- and middle-income countries (LMICs): An umbrella review. PloS one. 2021;16(6):e0251905.

For peer review only - http://bmjopen.bmj.com/site/about/guidelines.xhtml

15. Engelbert M, Jain M, Bagai A, Parsekar SS. Improving routine childhood immunisation outcomes in low-income and middle-income countries: an evidence gap map. BMJ open. 2022;12(11):e058258.

16. Hennessy EA, Johnson BT. Examining overlap of included studies in meta-reviews: Guidance for using the corrected covered area index. Research synthesis methods. 2020;11(1):134-45.

17. Pieper D, Antoine SL, Mathes T, Neugebauer EA, Eikermann M. Systematic review finds overlapping reviews were not mentioned in every other overview. Journal of clinical epidemiology. 2014;67(4):368-75.

18. SURE. Questions to assist with the critical appraisal of systematic reviews. The Specialist Unit for Review Evidence (SURE); 2018.

19. Pustejovsky JE, Tipton E. Meta-analysis with Robust Variance Estimation: Expanding the Range of Working Models. Prevention Science. 2022;23(3):425-38.

20. Tipton E. Robust variance estimation in meta-regression with binary dependent effects. Research synthesis methods. 2013;4(2):169-87.

21. DerSimonian R, Laird N. Meta-analysis in clinical trials. Controlled clinical trials. 1986;7(3):177-88.

22. Cochran WG. The Combination of Estimates from Different Experiments. Biometrics. 1954;10(1):101-29.

23. Higgins JP, Thompson SG. Quantifying heterogeneity in a meta-analysis. Statistics in medicine. 2002;21(11):1539-58.

24. Viechtbauer W, Cheung MW. Outlier and influence diagnostics for meta-analysis. Research synthesis methods. 2010;1(2):112-25.

25. Begg CB, Mazumdar M. Operating characteristics of a rank correlation test for publication bias. Biometrics. 1994;50(4):1088-101.

For peer review only - http://bmjopen.bmj.com/site/about/guidelines.xhtml

26. Sterne JAC, Egger M. Regression methods to detect publication and other bias in meta-analysis. In: H. R. Rothstein AJS, & M. Borenstein editor. Publication bias in meta-analysis: Prevention, assessment and adjustment Chichester: Wiley; 2005. p. 99-110.

27. Viechtbauer W. Conducting Meta-Analyses in R with the metafor Package. Journal of Statistical Software. 2010;36(3):1 - 48.

28. Grant RL. Converting an odds ratio to a range of plausible relative risks for better communication of research findings. BMJ : British Medical Journal. 2014;348:f7450.

29. Jain M, Shisler S, Lane C, Bagai A, Brown E, Engelbert M. Use of community engagement interventions to improve child immunisation in low-income and middle-income countries: a systematic review and meta-analysis. BMJ open. 2022;12(11):e061568.

30. Jain M, Shisler S, Lane C, Bagai A, Brown E, Engelbert M, et al. Use of community engagement interventions to improve child immunisation in low- and middle-income countries: A systematic review and meta-analysis. Campbell Systematic Reviews. 2022;18(3):e1253.

31. Ryman TK, Dietz V, Cairns KL. Too little but not too late: results of a literature review to improve routine immunization programs in developing countries. BMC Health Serv Res. 2008;8:134.

32. Gilmore B, McAuliffe E. Effectiveness of community health workers delivering preventive interventions for maternal and child health in low- and middle-income countries: a systematic review. BMC public health. 2013;13:847.

33. Crocker-Buque T, Mindra G, Duncan R, Mounier-Jack S. Immunization, urbanization and slums - a systematic review of factors and interventions. BMC public health. 2017;17(1):556.

34. Deardorff KV, Rubin Means A, Ásbjörnsdóttir KH, Walson J. Strategies to improve treatment coverage in community-based public health programs: A systematic review of the literature. PLoS neglected tropical diseases. 2018;12(2):e0006211.

For peer review only - http://bmjopen.bmj.com/site/about/guidelines.xhtml

35. Jarrett C, Wilson R, O'Leary M, Eckersberger E, Larson HJ. Strategies for addressing vaccine hesitancy - A systematic review. Vaccine. 2015;33(34):4180-90.

36. Kim SS, Patel M, Hinman A. Use of m-Health in polio eradication and other immunization activities in developing countries. Vaccine. 2017;35(10):1373-9.

37. Pinzón Flórez C, Díaz-Quijano D, Yáñez Álvarez I, Catalina Mesa D. Effectiveness of community workers on preventive measures to maternal and child health in low and middle income countries: systematic review of the literature. Revista Salud Uninorte. 2015;31(2):309-28.

38. Shea B, Andersson N, Henry D. Increasing the demand for childhood vaccination in developing countries: a systematic review. BMC international health and human rights. 2009;9 Suppl 1(Suppl 1):S5.

39. Naugle DA, Hornik RC. Systematic review of the effectiveness of mass media interventions for child survival in low- and middle-income countries. Journal of health communication. 2014;19 Suppl 1(sup1):190-215.

40. Yuan B, Målqvist M, Trygg N, Qian X, Ng N, Thomsen S. What interventions are effective on reducing inequalities in maternal and child health in low- and middle-income settings? A systematic review. BMC public health. 2014;14:634.

41. Lagarde M, Haines A, Palmer N. The impact of conditional cash transfers on health outcomes and use of health services in low and middle income countries. The Cochrane database of systematic reviews. 2009;2009(4):Cd008137.

42. Bassani DG, Arora P, Wazny K, Gaffey MF, Lenters L, Bhutta ZA. Financial incentives and coverage of child health interventions: a systematic review and meta-analysis. BMC public health. 2013;13 Suppl 3(Suppl 3):S30.

For peer review only

For peer review only - http://bmjopen.bmj.com/site/about/guidelines.xhtml

43. Bright T, Felix L, Kuper H, Polack S. A systematic review of strategies to increase access to health services among children in low and middle income countries. BMC Health Services Research. 2017;17(1):252.

44. de Souza Cruz RC, Moura LBA, Soares Neto JJ. Conditional cash transfers and the creation of equal opportunities of health for children in low and middle-income countries: a literature review. International journal for equity in health. 2017;16(1):161.

45. Omoniyi OS, Williams I. Realist Synthesis of the International Theory and Evidence on Strategies to Improve Childhood Vaccination in Low- and Middle-Income Countries: Developing Strategies for the Nigerian Healthcare System. International journal of health policy and management. 2020;9(7):274-85.

46. Onwuchekwa C, Verdonck K, Marchal B. Systematic Review on the Impact of Conditional Cash Transfers on Child Health Service Utilisation and Child Health in Sub-Saharan Africa. Frontiers in public health. 2021;9:643621.

47. Owusu-Addo E, Cross R. The impact of conditional cash transfers on child health in low- and middle-income countries: a systematic review. International journal of public health. 2014;59(4):609-18.

48. Pal M, Goodyear-Smith F, Exeter D. Systematic review of pertussis immunisation among Asians. International Journal of Human Rights in Healthcare. 2016;9(2):135-46.

49. Ranganathan M, Lagarde M. Promoting healthy behaviours and improving health outcomes in low and middle income countries: a review of the impact of conditional cash transfer programmes. Preventive medicine. 2012;55 Suppl:S95-s105.

50. Yunusa U, Garba SN, Umar AB, Idris SH, Bello UL, Abdulrashid I, et al. Mobile phone reminders for enhancing uptake, completeness and timeliness of routine childhood immunization in low and middle income countries: A systematic review and meta-analysis. Vaccine. 2021;39(2):209-21.

For peer review only - http://bmjopen.bmj.com/site/about/guidelines.xhtml

51. Chutiyami M, Wyver S, Amin J. Are Parent-Held Child Health Records a Valuable Health Intervention? A Systematic Review and Meta-Analysis. International journal of environmental research and public health. 2019;16(2).

52. Eze P, Lawani LO, Acharya Y. Short message service (SMS) reminders for childhood immunisation in low-income and middle-income countries: a systematic review and meta-analysis. BMJ Global Health. 2021;6(7):e005035.

53. Linde DS, Korsholm M, Katanga J, Rasch V, Lundh A, Andersen MS. One-way SMS and healthcare outcomes in Africa: Systematic review of randomised trials with meta-analysis. PloS one. 2019;14(6):e0217485.

54. Palmer MJ, Henschke N, Bergman H, Villanueva G, Maayan N, Tamrat T, et al. Targeted client communication via mobile devices for improving maternal, neonatal, and child health. The Cochrane database of systematic reviews. 2020;8(8):Cd013679.

55. Baequni NY. Is Maternal and Child Health Handbook effective? : Meta-Analysis of the Effects of MCH Handbook. Kokusai Hoken Iryo (Journal of International Health). 2012;27(2):121-7.

56. Karageorgos G, Andreadis I, Psychas K, Mourkousis G, Kiourti A, Lazzi G, et al. The Promise of Mobile Technologies for the Health Care System in the Developing World: A Systematic Review. IEEE reviews in biomedical engineering. 2019;12:100-22.

57. Giedion U, Andrés Alfonso E, Díaz Y. The impact of universal coverage schemes in the developing world: a review of the existing evidence. Washington DC: World Bank; 2013. Contract No.: 25.

58. Glenton C, Scheel IB, Lewin S, Swingler GH. Can lay health workers increase the uptake of childhood immunisation? Systematic review and typology. Tropical medicine & international health : TM & IH. 2011;16(9):1044-53.

For peer review only - http://bmjopen.bmj.com/site/about/guidelines.xhtml

59. Freeman PA, Schleiff M, Sacks E, Rassekh BM, Gupta S, Perry HB. Comprehensive review of the evidence regarding the effectiveness of community-based primary health care in improving maternal, neonatal and child health: 4. child health findings. Journal of global health. 2017;7(1):010904.

60. Rahman A, Fisher J, Bower P, Luchters S, Tran T, Yasamy MT, et al. Interventions for common perinatal mental disorders in women in low- and middle-income countries: a systematic review and meta-analysis. Bulletin of the World Health Organization. 2013;91(8):593-601i.

61. Witter S, Fretheim A, Kessy FL, Lindahl AK. Paying for performance to improve the delivery of health interventions in low- and middle-income countries The Cochrane database of systematic reviews. 2012(2):Cd007899.

62. Das A, Gopalan SS, Chandramohan D. Effect of pay for performance to improve quality of maternal and child care in low- and middle-income countries: a systematic review. BMC public health. 2016;16:321.

63. Parrado Jiménez D. Revisión sistemática de la literatura del efecto de pago por desempeño en la calidad de la atención en países de bajos y medianos ingresos. Bogota Colombia Universidad de Los Andes; 2013.

64. Nelson KN, Wallace AS, Sodha SV, Daniels D, Dietz V. Assessing strategies for increasing urban routine immunization coverage of childhood vaccines in low and middle-income countries: A systematic review of peer-reviewed literature. Vaccine. 2016;34(46):5495-503.

65. Nutman S, McKee D, Khoshnood K. Externalities of prevention of mother-to-child transmission programs: a systematic review. AIDS and behavior. 2013;17(2):445-60.

For peer review only - http://bmjopen.bmj.com/site/about/guidelines.xhtml

66. Smith BL, Zizzo S, Amzel A, Wiant S, Pezzulo MC, Konopka S, et al. Integration of Neonatal and Child Health Interventions with Pediatric HIV Interventions in Global Health. International journal of MCH and AIDS. 2018;7(1):192-206.

67. Lagarde M, Palmer N. The impact of contracting out on health outcomes and use of health services in low and middle-income countries. The Cochrane database of systematic reviews. 2009(4):Cd008133.

68. Odendaal WA, Ward K, Uneke J, Uro-Chukwu H, Chitama D, Balakrishna Y, et al. Contracting out to improve the use of clinical health services and health outcomes in low- and middle-income countries. The Cochrane database of systematic reviews. 2018;4(4):Cd008133.

69. Loevinsohn B, & , Harding A. Contracting for the delivery of community health services: A review of Global Experience. Washington, DC: World Bank; 2004.

70. de Cock C, van Velthoven M, Milne-Ives M, Mooney M, Meinert E. Use of Apps to Promote Childhood Vaccination: Systematic Review. JMIR mHealth and uHealth. 2020;8(5):e17371.

71. Watterson JL, Walsh J, Madeka I. Using mHealth to Improve Usage of Antenatal Care, Postnatal Care, and Immunization: A Systematic Review of the Literature. BioMed research international. 2015;2015:153402.

72. Molina E, Carella L, Pacheco A, Cruces G, Gasparini L. Community monitoring interventions to curb corruption and increase access and quality of service delivery in low- and middle-income countries: a systematic review. Campbell Systematic Reviews. 2016;12(1):1-204.

73. Desai S, Misra M, Das A, Singh RJ, Sehgal M, Gram L, et al. Community interventions with women's groups to improve women's and children's health in India: a mixed-methods systematic review of effects, enablers and barriers. BMJ Glob Health. 2020;5(12).

For peer review only

For peer review only - http://bmjopen.bmj.com/site/about/guidelines.xhtml

74. Hatt L, Johns C, Connor C, Meline M, Kukla M, & , Moat K. Impact of Health Systems Strengthening on Health. Bethesda, MD: Health Finance & Governance Project, Abt Associates; 2015.

75. Engelbert M, Jain M., Bagai A, Berkovics B, Atika D, Parsekar SS. Improving routine childhood immunisation outcomes in low- and middle-income countries: an evidence gap map. 2022.

Supplementary material

Supplementary material includes:

Appendix 1: Intervention-outcome framework

Appendix 2: List of excluded studies – after full-text screening

Appendix 3: List of sources and search strings

Appendix 4: Data extraction template

Appendix 5: Quality appraisal tool

Appendix 6: Summary of included systematic reviews

Appendix 7: Risk of bias of included systematic reviews

Appendix 8: RVE analysis – 1st tier synthesis

Appendix 9: RVE analysis – 2nd tier synthesis

Appendix 10: Health-systems oriented interventions

Appendix 11: Community-level interventions

Figure legends

Figure 1. PRISMA flow diagram

Figure 2: Random-effects model of one-time sensitisation and education campaigns (AA2)

Figure 3: RVE of interventions of written or pictorial messages to caregivers (AB4), mean baseline coverage values

For peer review only

For peer review only - http://bmjopen.bmj.com/site/about/guidelines.xhtml

Figure 1: PRISMA flow diagram

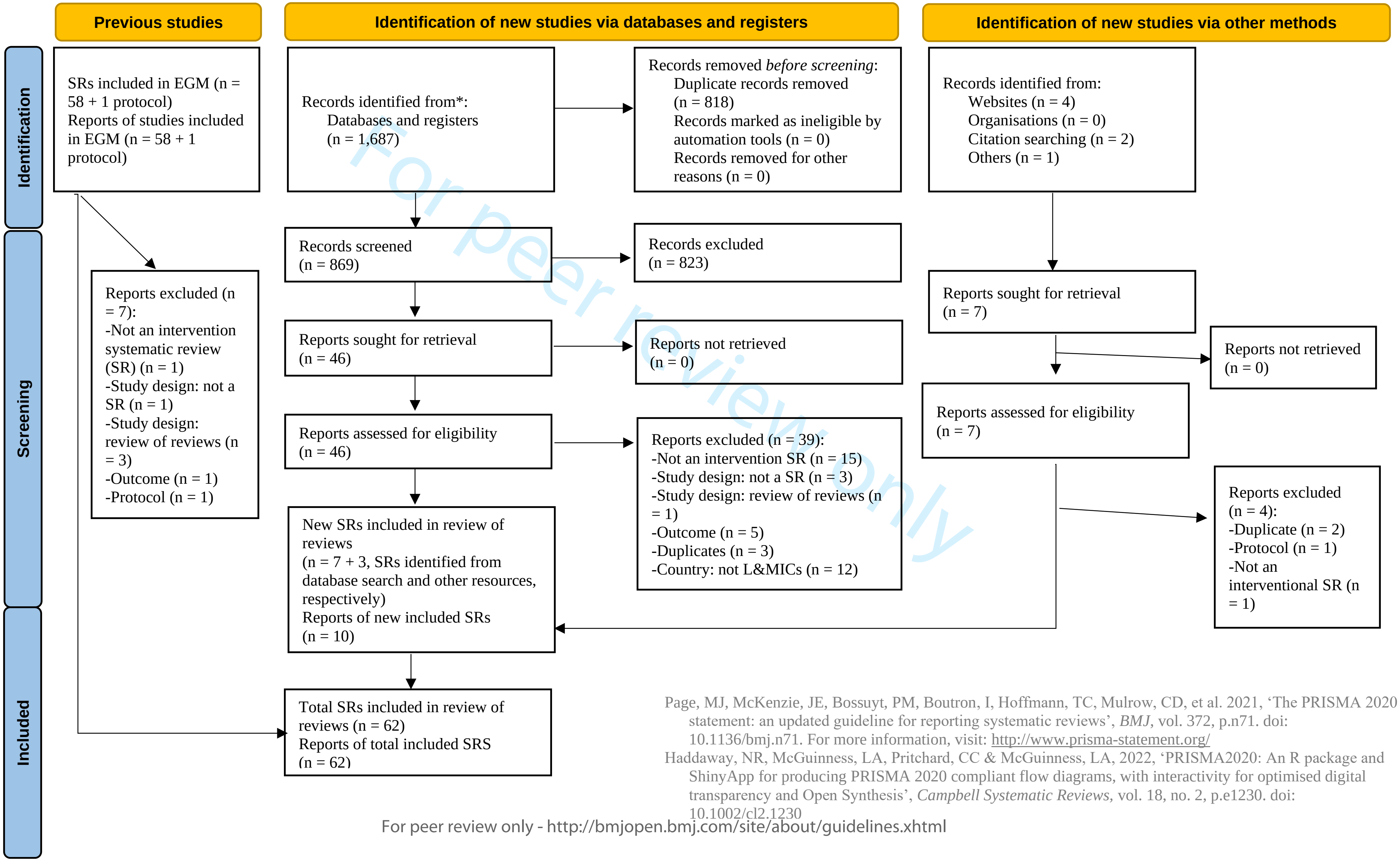

Page, MJ, McKenzie, JE, Bossuyt, PM, Boutron, I, Hoffmann, TC, Mulrow, CD, et al. 2021, 'The PRISMA 2020 statement: an updated guideline for reporting systematic reviews', *BMJ*, vol. 372, p.n71. doi: 10.1136/bmj.n71. For more information, visit: http://www.prisma-statement.org/

Haddaway, NR, McGuinness, LA, Pritchard, CC & McGuinness, LA, 2022, 'PRISMA2020: An R package and ShinyApp for producing PRISMA 2020 compliant flow diagrams, with interactivity for optimised digital transparency and Open Synthesis', *Campbell Systematic Reviews*, vol. 18, no. 2, p.e1230. doi: 10.1002/cl2.1230

For peer review only

Figure 2: Random-effects model of one-time sensitisation and education campaigns (AA2)

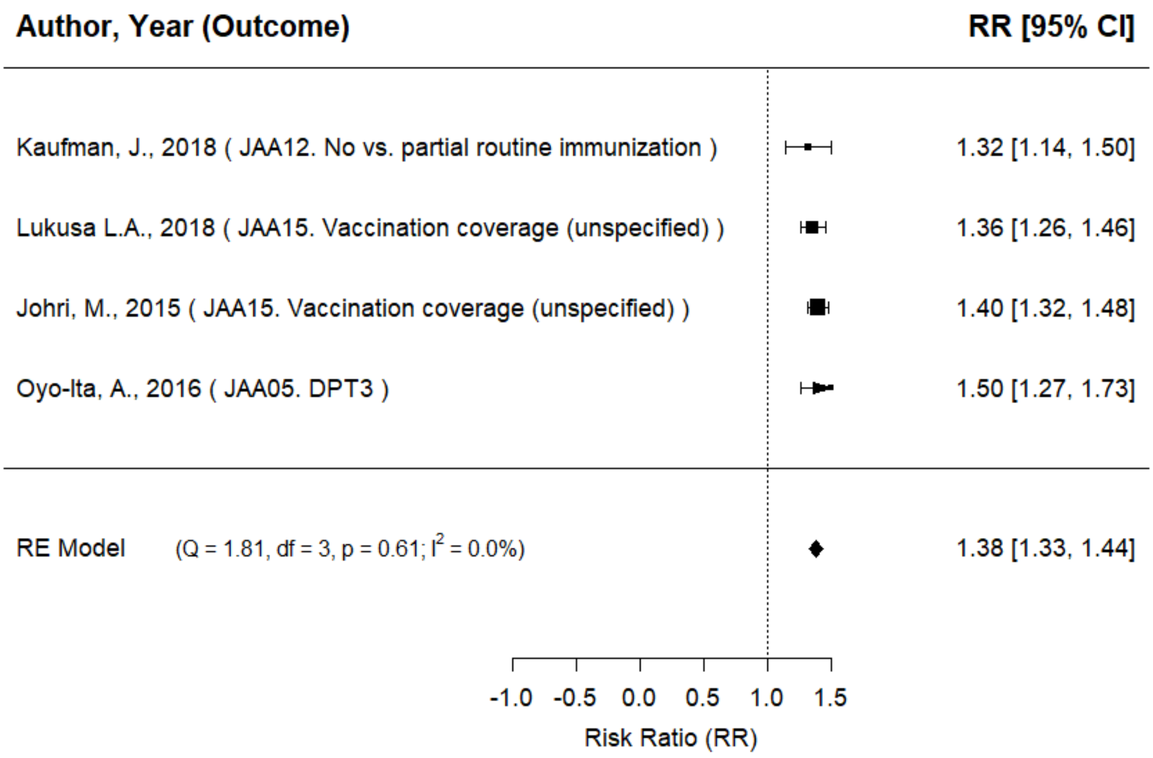

Note: Intervention and outcome codes are taken from Appendix 1.

For peer review only

Figure 3: RVE of interventions of written or pictorial messages to caregivers (AB4), mean baseline coverage values

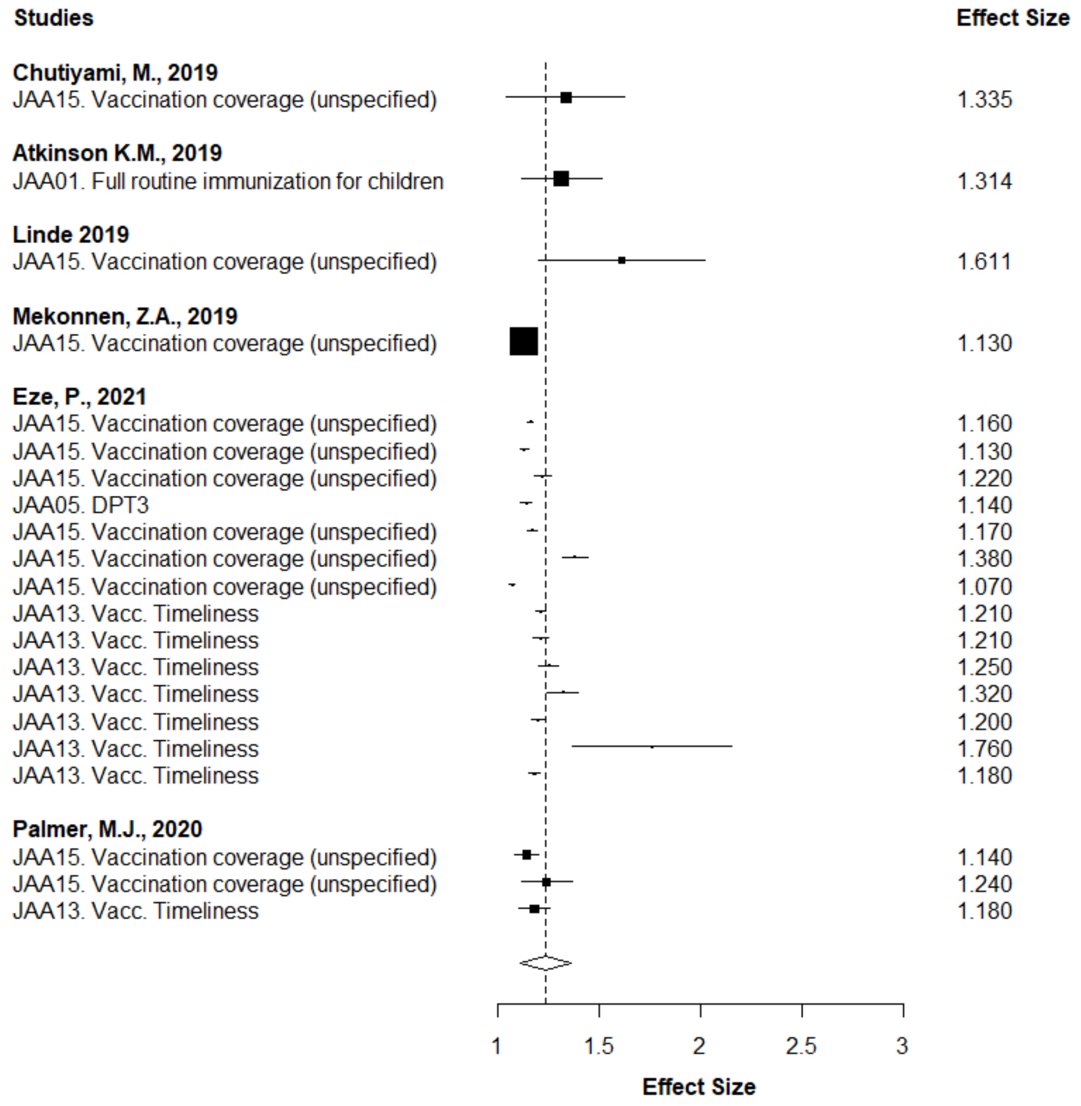

Note: Intervention and outcome codes are taken from Appendix 1.

Supplementary file

Contents

Appendix 1: Intervention-outcome framework	2
Appendix 2: List of excluded studies – after full-text screening	10
Appendix 3: List of sources and search strings	13
Appendix 4: Data extraction template	17
Appendix 5: Quality appraisal tool	20
Appendix 6: Summary of included systematic reviews	33
Appendix 7: Risk of bias of included systematic reviews	49
Appendix 8: RVE analysis – 1st tier synthesis	53
Appendix 9: RVE analysis – 2nd tier synthesis	56
Appendix 10: Health-systems oriented interventions	59
Appendix 11: Community-level interventions	63
References	64

For peer review only

Appendix 1: Intervention-outcome framework

Interventions

Intervention			Definition/guidance
A. Caregiver-oriented	AA. Information & education	AA1. Sustained sensitisation and education campaigns	Sustained interventions (i.e., those that are *not* designed with a fixed end date in mind) that provide targeted caregivers with information about immunisation and its importance, the vaccination schedule, or where and how to access immunisation services. For example, village health and nutrition days (VHNDs) in India in which health education and counselling services are provided to pregnant women and mothers of young children on a regular basis.
		AA2. One-time sensitisation and education campaigns	One-off interventions (i.e., those designed with a fixed end date in mind) that provide targeted caregivers with information about immunisation and its importance, the vaccination schedule, or where and how to access immunisation services.
		AA3. Public information campaigns	Mass media campaigns through newspapers, radio and TV which provide caregivers with information about immunisation and its importance, the vaccination schedule, or where and how to access immunisation services. Because of the nature of communication mediums they cannot be targeted to a specific audience.
	AB. Incentives & motivation	AB1. Material/ monetary incentives for caregivers	Interventions that incentivise caregivers to vaccinate through items with monetary value. This could be cash transfers or material goods like food or home goods.
		AB2. Non-material incentives for caregivers	Interventions that seek to motivate caregivers to vaccinate through non-material incentives like social recognition. Unlike reminder messages, interventions in this category should seek to *create* or *strengthen* a desire to vaccinate, rather than activating a standing intention to vaccinate.
		AB3. Automated voice messages to caregivers	Use of automatically-generated voice messages (usually delivered to a mobile phone) that remind caregivers about upcoming vaccinations, provide them information on place and time of vaccination and encourage them to vaccinate. While these messages may contain some motivational component (e.g., stressing the importance of vaccination in addition to reminding caregivers about an upcoming appointment), voice messages should be categorised only here and not also under "non-material incentives", unless they rely on a substantive motivational factor like social recognition.
		AB4. Written or pictorial messages (SMS, stickers, flyers etc.) to caregivers	Use of written messages/pictorial that remind caregivers about upcoming vaccinations, provide them information on place and time of vaccination and encourage them to vaccinate. While these messages may contain some motivational component (e.g., stressing the importance of vaccination in addition to reminding caregivers about an upcoming appointment), messages should be categorised only here and not also under "non-material incentives", unless they rely on a substantive motivational factor like social recognition.
		AB5. Changes to health system user fees	Any change to the monetary costs to users for accessing the health system. This can include introduction or elimination of fees at the point of service, or pre-payment or insurance schemes.

For peer review only

Intervention			Definition/guidance
B. Health system-oriented	BA. Education & training	BA1. Formal health worker training and education	Programmes that train or educate formal health workers (FHWs). FHWs are typically vaccinators (and they tend to provide/prescribe medication or administer tests such as recording blood glucose level, etc.). (The only likely exception to this would be oral polio vaccination (OPV), especially supplementary polio campaigns, where CHWs or community volunteers may be enlisted to administer the vaccination.)
		BA2. Community health worker training and education	Programmes that train or educate community health workers (CHWs). CHWs are defined as "paraprofessionals or lay individuals with an in-depth understanding of the community culture and language, have received standardised job-related training of a shorter duration than health professionals, and their primary goal is to provide culturally appropriate health services to the community."[1]
	BB. Planning, implementation, monitoring	BB1. Formal health worker involvement in planning & monitoring	Interventions that give FHWs substantive roles in creating plans/strategies to deliver vaccination services and/or monitor vaccination coverage in the community.
		BB2. Community health worker involvement in planning & monitoring	Interventions that give CHWs substantive roles in creating plans/strategies to deliver vaccination services and/or monitor vaccination coverage in the community.
		BB3. Paper-based tracking	Paper-based systems (e.g., logbooks) used by health workers to keep track of children in the community who are due for upcoming vaccinations or have not received scheduled vaccinations. Note that if a study merely mentions the existence of logbooks, that is not sufficient to code it as this intervention. The logbooks must be specifically used for tracking upcoming and missed vaccinations.
		BB4. Promoting outreach to vaccine-hesitant groups	Outreach to groups that, because of religious, cultural, or other reasons, are suspicious of vaccination or have specific fears about it (e.g., that vaccinations cause infertility or spread disease).
		BB5. Outreach to vulnerable populations (hard-to-reach, SES, caste, etc.)	Outreach to groups that are vulnerable in a way that affects their access to vaccination services. They may be in hard-to-reach geographical areas, have low socioeconomic status (including wealth and education), or be from groups marginalised based on caste, ethnicity, etc. This also includes interventions that set up temporary and mobile clinics to deliver vaccines, to make vaccination services more accessible.
		BB6. Outreach to migrant populations	Outreach to populations who have migrated temporarily or seasonally because of cultural or employment reasons.
		BB7. Home visits	Use of visits to caregivers' homes by health workers. This includes both visits to encourage caregivers to vaccinate their children, and visits to deliver vaccines, provided they are *targeted* visits to specific households for routine immunization. General door-to-door campaigns to deliver specific vaccines go under BC1.

[1] From Olaniran, A., Smith, H., Unkels, R., Bar-Zeev, S., & van den Broek, N. (2017). Who is a community health worker? – A systematic review of definitions. *Global Health Action*, *10*(1), 1272223. https://doi.org/10.1080/16549716.2017.1272223.

For peer review only - http://bmjopen.bmj.com/site/about/guidelines.xhtml

Intervention			Definition/guidance
		BB8. Campaigns to vaccinate refugee populations	Interventions that make a special effort to vaccinate populations which have been displaced temporarily or permanently because of conflict, war or famine.
	BC. Supplementary Immunisation Activities	BC1. National/sub-national immunisation days	Supplementary immunisation activities (SIA) are mass immunization campaigns which complement routine immunisation activities whereby a vaccine is taken simultaneously to many residents of a community within a defined short space of time. They have generally been conducted for polio and measles. They may be called national or sub-national immunisation days. They may happen through booth days or door to door vaccination campaigns. On a booth day a large number of fixed site booths are set up throughout the target area for children to be brought to receive a specific vaccine like polio. In door-to-door campaigns vaccination teams go door to door to every house, checking each child under five to see if they have received the specific vaccine, and if they have not that vaccination is provided.
	BD. Incentives & motivation	BD1. Material/monetary incentives for health workers	Interventions that incentivise formal or community health workers to deliver vaccination services through items with monetary value. This could be cash transfers or material goods like food or home goods.
		BD2. Non-material incentives for health workers	Interventions that use non-material incentives like social recognition to incentivise formal or community health workers to deliver vaccination services.
		BD3. Automated voice messages to health workers	Use of automatically-generated voice messages (usually delivered to a mobile phone) that remind health workers about upcoming vaccinations for community members, provide them information to help plan their work, and encourage them to conduct outreach to those community members.
		BD4. Written or pictorial messages (SMS, stickers, flyers etc.) to health workers	Use of written/pictorial messages that remind health workers about upcoming vaccinations for community members, provide them information to help plan their work, and encourage them to conduct outreach to those community members.
		BD5. Pay-for-performance schemes	Schemes whereby health centres or districts receive funding based on their performance in delivering health services (e.g., they receive a given amount of funding for each child vaccinated or each antenatal care visit completed). Also known as “results-based financing”.
	BE. Infrastructure	BE1. Building & upgrading health clinics	Projects that build new permanent health clinics, or provide physical upgrades to existing clinics.
		BE2. Cold chain infrastructure improvements	Interventions that improve the ability of health systems to maintain vaccine cold chains.
	BF. Health system governance,	BF1. Health system strategic planning	Initiatives at the national or sub-national level to develop plans and governance structures designed to improve vaccination services. This also includes interventions that improve the human resource availability, strategies, policies and plans in existing health governance and delivery structures that may or may not be directly related to immunisation services.

For peer review only

Intervention			Definition/guidance
	policies and financing	BF2. Vaccination guidelines	Changes to official national or sub-national guidelines about when and how vaccinations should be administered. This includes studies comparing two different approaches to administering vaccines (e.g., one measles dose vs. two), which could be made into guidelines.
		BF3. Changes to broader governance systems (beyond health systems)	Interventions that modify general governance systems not directly related to health. An example would be a policy dictating a certain level of representation for women in local or national governing bodies.
		BF4. Health system financing	Interventions that increase the national or sub-national financing of health or specifically for vaccination in absolute terms or as a proportion of GDP. This also includes results- or performance-based financing.
	BG. Technology & mHealth	BG1. New HMIS/Dashboard systems (incl. improved data collection)	New digital tools and systems designed to improve health system capacity to monitor and deliver vaccination services. This includes apps for mobile phones or tablets, as well as desktop-based software and setting up online dashboards. A common type of intervention in this category is giving health workers tablets with an app that allows them to register vaccination information (and for other health services) for community members, track their vaccination schedules and provide them readily-accessible information about vaccination.
		BG2. Capacity building (e.g., training) for existing systems	Initiatives to train people working in the health system (including frontline health workers and administrative personnel) to improve their ability to use existing digital tools and systems more effectively.
C. Other community member-oriented	CA. Other community member-oriented	CA1. Faith-based outreach/outreach using local leaders	Interventions that enlist influential community members (often religious or other traditional leaders) to promote vaccination in the community.
D. Community-level	DA. Communication & dialogue	DA1. Collaborating with whole community	Interventions that involve or plan to involve ALL community members beyond health workers in various aspects of the intervention, such as developing plans and solutions to improve immunisation outcomes in the community.
		DA2. Collaborating with selected community groups and networks	Interventions that involve *selected* groups or networks of community members beyond health workers (other than the traditional or religious leaders who fall under CA1) in developing plans and solutions to improve immunisation outcomes in the community. This includes interventions focusing on mother's groups, father clubs, self-help groups, etc.
	DB. Tracking & registering	DB1. Community tracking and registering	Interventions that involve community members beyond health workers (e.g., caregivers or any other community members other than the traditional leaders who fall under CA1) in registering children with the health system so their vaccination status can be tracked, and/or tracking which children are due for vaccinations.
E. Policies and institutions	EA. Education policy and infrastructure	EA1. Education policy and infrastructure	Policy interventions that affect education levels of people in a country or region. An example would be an intervention that makes education mandatory for a particular population where it had previously been optional.

For peer review only

Intervention			Definition/guidance
	EB. Non-health/ education infrastructure	EB1. Non-health/ education infrastructure (e.g., electrification)	General improvements in physical infrastructure beyond the health system. This may include electrification, roads, sanitation improvements, etc.
F. Multicomponent			

Outcomes

Outcome			Definition/guidance
H. Behavioural, social and practical barriers faced by caregivers and communities	HAA. Thinking and feeling (attitudes, confidence)	HAA1. Knowledge about immunisation	Caregivers' knowledge about immunisation in general (i.e., its purpose and role in preventing disease)
		HAA2. Attitudes about immunisation	Caregivers' attitudes towards immunisation in general (i.e., whether they view it favourably or unfavourably or have high or low confidence in its efficacy)
		HAA3. Attitudes about health providers	Caregivers' attitudes about health providers in general (i.e., whether they generally trust health providers to provide high-quality and appropriate care)
	HAB. Social processes	HAB1. Community norms	Community-level attitudes and beliefs about immunisation, including whether there is social pressure to vaccinate or not vaccinate. This can be measured either objectively through aggregating community-level responses or subjectively by soliciting individual community members' beliefs about the norms in their community. This includes attitudes and beliefs about immunization of key influencers in the community like traditional or religious leaders.
		HAB2. Household norms & decision-making	Norms and practices determining who in a household (e.g., mother, father, mother-in-law) provides input to decisions about whether to vaccinate, and how much decision-making power individual household members have. This also covers attitudes towards immunisation of household members other than the primary caregiver.
	HAC. Readiness to vaccinate	HAC1. Readiness to vaccinate	Caregivers' motivation, intention and plan to vaccinate their children. Note this is more specific than general attitudes towards immunisation covered under HAA2
		HAC2. Reasons for not vaccinating	Caregivers' stated reasons for not vaccinating children. This may include factors such as convenience (which would also be coded under "perceived convenience of vaccination"), but only use this code if the factors mentioned are specifically framed as reasons for not vaccinating. Also, only use this code when the study measures *effects* of the intervention on this outcome. If the study gathers data on reasons for not vaccinating but does not provide an effect size for this as an outcome, use the cross-cutting theme but not this outcome code.
	HAD. Practical factors	HAD1. Awareness of place, time, schedule for vaccination	Caregivers' knowledge about when and where they should go for vaccinations.
		HAD2. Actual cost of vaccinating	Actual cost of vaccinating the child, including vaccine cost, transportation cost, loss of wage/income due to missed work, and providing gifts/unofficial payments to the health providers

For peer review only

Outcome				Definition/guidance
			HAD3. Perceived convenience of vaccination	Subjective measures (i.e., caregivers' beliefs) of the convenience of taking the child for vaccination, such as opportunity costs of vaccinating a child (e.g., not able to care for a younger child), long lines at health clinic, and inconvenient day/time of vaccination.
			HAD4. Experience and satisfaction with health services	The actual experience of health services in the last visit such as duration of waiting time, availability of vaccine or vaccinator, and behaviour of the health staff (respect, rudeness). This also includes level of satisfaction with the health services, professionals and facilities.
			HAD5. Vaccination health card availability/ retention	Measures of whether caregivers possess vaccination health cards provided by the health system, and/or whether caregivers can show the vaccination health card.
			HAD6. Perception of vaccination side effects	Caregivers' perceptions of the likelihood and severity of side effects from vaccination, and their knowledge of how to recognise and treat normal side effects.
I. Delivery of vaccination services	IA. Health workforce	IAA. Comm. health workers	IAA1. Community HW motivation, capacity & performance	Any measure of CHWs' capacity to deliver quality and timely vaccination services.
			IAA2. Supply of CHWs	The total availability of CHW services in the community, taking into account both the number of CHWs and the time they have available.
		IAB. Vaccinators	IAB1. Formal HW supply	The total availability of FHW services in the community, taking into account both the number of FHWs and the time they have available.
			IAB2. Availability of HWs at vacc. point of service	Whether vaccinators are present at vaccination point of service (e.g., health clinic) when vaccination services are supposed to be offered. This includes measures of health worker "absenteeism". This is different from perception or experience of health workers from caregivers' point of view. The source of information for this outcome can be administrative data or survey of health facilities and staff.
			IAB3. Formal HW motivation, capacity & performance	Any measure of FHWs' capacity to deliver quality and timely vaccination services, or of their performance in doing so.
		IAC. Administrators	IAC1. Admin staffing	The number of staff in administrative posts in the health system (i.e., those not directly involved in health service provision).
			IAC2. Capacity of health admin. responsible for vaccination	The knowledge, skills, and motivation of staff in administrative posts in the health system, including leadership positions.
	IBA. Health information systems		IBA1. Immunisation data collection (quality, completeness)	The health system's capacity for and success in collecting data about vaccination coverage and service quality for regular monitoring and accountability.

For peer review only

Outcome			Definition/guidance
		IBA2. Defaulter tracing	The health system's capacity for and success in identifying vaccination "defaulters" (i.e., those whose children receive early vaccine doses but do not return for subsequent vaccinations).
		IBA3. Supply chain management	The health system's capacity to monitor the supply of vaccines at points of service, ensure reliable supply chains, and avoid supply "bottlenecks".
		IBA4. Immunisation data availability/ transparency	How easy it is for stakeholders within and beyond the health system to access data about vaccination service quality, coverage and timeliness.
	ICA. Vaccine availability	ICA1. Stockouts	The frequency and duration of incidents when vaccines are out of stock at points of service, or when vaccines are in stock but cannot be administered to children who are brought in (e.g., because health workers are instructed not to open a new vial if there are not enough children to receive all doses in the vial, meaning that some of the vaccine would go to waste). Source of this information is administrative data and/or survey of health facilities and staff
		ICA2. Quality of cold chain infrastructure	The availability, quality and upkeep of physical equipment and place for cold chain storage of vaccines
	IDA. Resources	IDA1. National or sub-national vaccine financing	Change in financial resources for national or sub-national vaccination programmes, policies or strategies.

JAA. Vaccination coverage	JAA01. Full routine immunisation for children	Binary measure of whether or not children have received all routine vaccinations for the relevant country or region.
	JAA02. BCG	Binary measure of whether or not children have received the BCG vaccine. This may be measured by checking whether children have a BCG vaccination scar.
	JAA03. DPT1	Binary measure of whether children have received first dose of the DPT/penta vaccine. If study does not specify which doses were included in the outcome measures (i.e., the outcome is just "DPT/pentavalent vaccination" then apply this code unless it is clear they are talking about full DPT/penta vaccination, in which case code it as JAA05: DPT3.
	JAA04. DPT2	Binary measure of whether or not children have received the second dose of the DPT or pentavalent
	JAA05. DPT3	Binary measure of whether or not children have received the third dose of the DPT or pentavalent vaccine. If the study does not specifically say "DPT3" (or "pentavalent 3"), but refers to "complete DPT/penta vaccination", then use this code.
	JAA06. OPV0	Binary measure of whether children have received 1st dose of oral polio vaccine (recommended for administration at birth).
	JAA07. OPV1	Binary measure of whether children have received 2nd dose of the oral polio vaccine (recommended for administration at 6 weeks).

For peer review only

	JAA08. OPV2	Binary measure of whether children have received the 3rd dose of the oral polio vaccine (recommended for administration at 10 weeks).
	JAA09. OPV3	Binary measure of whether children have received the 4th and final dose of the oral polio vaccine (recommended for administration at 14 weeks).
	JAA10. IPV	Binary measure of whether children have received inactivated polio vaccine, given as injection. Countries differ in their guidelines/practices regarding IPV, so, please note the number of doses and age(s) when administered.
	JAA11. Measles	Binary measure of whether or not children have received the measles vaccine
	JAA12. No vs. partial routine immunisation	Proportion of children who receive at least one vaccination versus those who are completely unvaccinated.
	JAA13. Vacc. timeliness	Proportion of vaccinations delivered on time according to the recommended schedule, vs those that are delivered late.
	JAA14. Drop out rate for multidose vaccines	Proportion of children who fail to receive the complete course of a multidose vaccine (DPT/penta, OPV, or in some cases measles) after receiving the first dose.
	JAA15. Vaccination coverage (unspecified)	Use this code if the evaluation or systematic review refers to impacts on routine vaccination coverage for children, but without specifying which vaccines.
KAA. Health outcomes	KAA1. Childhood morbidity	Incidence of vaccine-preventable diseases or symptoms associated with those diseases (e.g., diarrhoea) among children under 5.
	KAA2. Neonatal/Infant/ Child Mortality	Incidence of mortality among children below five years from all causes. Neonatal mortality refers to death of a live-born baby within the first 28 days of life. *Infant mortality* is the death of young children under the age of 1. Child mortality, refers to the mortality of children under the age of five. This category includes all-cause mortality.

For peer review only

Appendix 2: List of excluded studies – after full-text screening

Academic databases

Sr. No.	Authors & Year	Title	Reasons
1	Asciutto et al., 2020[1]	"A systematic review of economic evaluations of neonatal and maternal healthcare in immigrant and ethnic minority women"	Exclude on country
2	Balzarini et al., 2020[2]	Does the use of personal electronic health records increase vaccine uptake? A systematic review	Exclude on country
3	Diks, Hiligsmann, & van der Putten, 2021[3]	Vaccine preferences driving vaccine-decision making of different target groups: a systematic review of choice-based experiments	Exclude on country
4	Haroune & King, 2020[4]	Factors contributing to parental 'vaccine hesitancy' for childhood immunisations	Exclude on country
5	Hutchinson & Smith, 2020[5]	Effectiveness of strategies to increase uptake of pertussis vaccination by new parents and family caregivers: A systematic review	Exclude on country
6	Jia et al., 2021[6]	Payment methods for healthcare providers working in outpatient healthcare settings	Exclude on country
7	Lambert et al., 2021[7]	Reducing burden from respiratory infections in refugees and immigrants: a systematic review of interventions in OECD, EU, EEA and EU-applicant countries	Exclude on country
8	Machado et al., 2021[8]	Effective interventions to increase routine childhood immunization coverage in low socioeconomic status communities in developed countries: A systematic review and critical appraisal of peer-reviewed literature	Exclude on country
9	Odone et al., 2021[9]	Current understandings of the impact of mandatory vaccination laws in Europe	Exclude on country
10	Ortiz-Sánchez et al., 2020[10]	Analysis of the Anti-Vaccine Movement in Social Networks: A Systematic Review	Exclude on country
11	Pires, 2021[11]	What is the state-of-the-art in clinical trials on vaccine hesitancy 2015-2020?	Exclude on country
12	Reñosa et al., 2021[12]	Nudging toward vaccination: a systematic review	Exclude on country
13	Bednarek & Klepacz, 2020[13]	Vaccinology education of nurses and the current immunoprophylaxis recommendations for children with juvenile idiopathic arthritis	Exclude on outcome
14	Chenet & Tapia-Limonchi, 2021[14]	Reaching the theoretical herd immunity threshold in Iquitos, Peru: are seroprevalence data enough?	Exclude on outcome
15	Chitkara et al., 2020[15]	Pertussis vaccination in mixed markets: Recommendations from the Global Pertussis Initiative	Exclude on outcome
16	Meteke et al., 2020[16]	Delivering infectious disease interventions to women and children in conflict settings: A systematic review	Exclude on outcome
17	Nnaji et al., 2021[17]	Implementation research approaches to promoting universal health coverage in Africa: a scoping review	Exclude on outcome
18	Chaney et al., 2021[18]	Every child on the map: A theory of change framework for improving childhood immunization coverage and equity using geospatial data and technologies	Exclude on study design, not a systematic review
19	Soans et al., 2021[19]	Vaccination in preterm and low birth weight infants in India	Exclude on study design, not a systematic review

For peer review only

20	Tankwanchi et al., 2020[20]	Taking stock of vaccine hesitancy among migrants: A scoping review protocol	Exclude on study design, not a systematic review
21	Agrawal et al., 2020[21]	Vaccine Hesitancy as a Challenge or Vaccine Confidence as an Opportunity for Childhood Immunisation in India	Not an effectiveness/intervention systematic review
22	Barbosa de Souza et al., 2021[22]	EXPERIÊNCIAS BRASILEIRAS NO ACOMPANHAMENTO DE CRESCIMENTO E DESENVOLVIMENTO INFANTIL NO CONTEXTO DA ATENÇÃO BÁSICA	Not an effectiveness/intervention systematic review
23	Berrueta et al., 2021[23]	Maternal and neonatal data collection systems in low- and middle-income countries for maternal vaccines active safety surveillance systems: A scoping review	Not an effectiveness/intervention systematic review
24	Cassocera et al., 2020[24]	40 years of immunization in Mozambique: a narrative review of literature, accomplishments, and perspectives	Not an effectiveness/intervention systematic review
25	Dansereau et al., 2020[25]	A systematic review of the agreement of recall, home-based records, facility records, BCG scar, and serology for ascertaining vaccination status in low and middle-income countries	Not an effectiveness/intervention systematic review
26	Farida, Widyaningsih, & Murti, 2020[26]	The effect of maternal education and antenatal care on basic immunization completeness in children aged 12-23 months in Asian and African: meta-analysis	Not an effectiveness/intervention systematic review
27	Galadima et al., 2021[27]	Factors influencing childhood immunisation uptake in Africa: a systematic review	Not an effectiveness/intervention systematic review
28	Kalaij, Sugiyanto, & Ilham, 2021[28]	Factors Associated With Vaccination Compliance in Southeast Asian Children: A Systematic Review	Not an effectiveness/intervention systematic review
29	Kulkarni et al., 2021[29]	Trends in Urban Immunization Coverage in India: A Meta-Analysis and Meta-Regression	Not an effectiveness/intervention systematic review
30	Laserna Jiménez et al., 2021[30]	Paediatric nursing clinical competences in primary healthcare: A systematic review	Not an effectiveness/intervention systematic review
31	Lassi et al., 2021[31]	The impact of the COVID-19 pandemic on immunization campaigns and programs: A systematic review	Not an effectiveness/intervention systematic review
32	Lieneck, Herzog, & Krips, 2021[32]	Analysis of Facilitators and Barriers to the Delivery of Routine Care during the COVID-19 Global Pandemic: A Systematic Review	Not an effectiveness/intervention systematic review
33	Wariri et al., 2021[33]	Timeliness of routine childhood vaccination in low- And middle-income countries, 1978-2021: Protocol for a scoping review to map methodologic gaps and determinants	Not an effectiveness/intervention systematic review
34	Priya et al., 2020[34]	Vaccination coverage and vaccine hesitancy among vulnerable population of India	Not an effectiveness/intervention systematic review
35	Smith, Hodson, & Rubin, 2021[35]	Parental attitudes towards mandatory vaccination; a systematic review	Not an effectiveness/intervention systematic review
36	Besnier et al., 2021[36]	Which public health interventions are effective in reducing morbidity, mortality and health inequalities from infectious diseases amongst children in low- and middle-income countries (L&MICs): An umbrella review	Exclude on study design, it is a review of reviews
37	Munk et al., 2019[37]	Systematic review of the costs and effectiveness of interventions to increase infant vaccination coverage in low- and middle-income countries	Duplicate

For peer review only

38	Omoniyi & Williams, 2020[38]	Realist Synthesis of the International Theory and Evidence on Strategies to Improve Childhood Vaccination in Low- and Middle-Income Countries: Developing Strategies for the Nigerian Healthcare System	Duplicate
39	Barbosa de Souza et al., 2021[22]	Brazilian experiences in monitoring growth and child development in the context of primary care (EXPERIÊNCIAS BRASILEIRAS NO ACOMPANHAMENTO DE CRESCIMENTO E DESENVOLVIMENTO INFANTIL NO CONTEXTO DA ATENÇÃO BÁSICA)	Duplicate

Grey literature

Sr. No.	Authors & Year	Title	Reasons
1	Siribaddana, Sahay, & Hewapathirana, 2021[39]	Deploying Electronic Immunization Information Systems in Low and Middle Income Countries in Response to COVID-19: Systematic Review of Best Evidence Practices	Not an effectiveness/intervention systematic review
2	Jain et al., 2020[40]	PROTOCOL: Use of community participation interventions to improve child immunisation in low- and middle-income countries: A systematic review and meta-analysis	Exclude as it is a protocol: completed review (unpublished at the time of updated search) has been included, Jain et al., 2022[41, 42]
3	Onwucheckwa et al., 2021[43]	Systematic Review on the Impact of Conditional Cash Transfers on Child Health Service Utilisation and Child Health in Sub-Saharan Africa	Duplicate
4	Eze, Lawani & Acharya, 2021[44]	Short message service (SMS) reminders for childhood immunisation in low-income and middle-income countries: a systematic review and meta-analysis.	Duplicate

Systematic reviews previously included in EGM, excluded with reasons:

Sr. No.	Authors & Year	Title	Reasons
1	Pantoja et al., 2017[45]	Implementation Strategies for Health Systems in Low-Income Countries: An Overview of Systematic Reviews (Review)	Exclude on study design, it is a review of reviews
2	Hatt et al., 2015[46]	Impact of Health Systems Strengthening on Health	Exclude on study design, it is a review of reviews
3	Wiysonge, 2012[47]	Building evidence for improving childhood immunisation coverage in Africa	Exclude on study design, it is a review of reviews
4	Van Velthoven et al., 2020[48]	Use of Apps to Promote Childhood Vaccination: Protocol for a Systematic Review	Exclude as it is a protocol: final published review is included, de Cock 2020
5	Abdulrahman et al., 2017[49]	mHealth: a narrative synthesis of evidence of its application in improving childhood immunization coverage	Exclude on study design, not a systematic review
6	Aaby et al., 2016[50]	Randomized Trials Comparing Inactivated Vaccine After Medium-or High-titer Measles Vaccine With Standard Titer Measles Vaccine After Inactivated Vaccine	Not an effectiveness/intervention systematic review
7	Bhutta et al., 2008[51]	Interventions to address maternal, newborn, and child survival: what difference can integrated primary health care strategies make?	Exclude on outcomes

For peer review only

Appendix 3: List of sources and search strings

Electronic searches
We conducted electronic searches of the following databases of published sources:

1. MEDLINE
2. CAB Global Health
3. EMBASE
4. Cochrane Controlled Trials Register (CENTRAL)
5. CINAHL
6. PsycINFO
7. Popline
8. Africa-wide information
9. Academic search complete
10. Scopus
11. Campbell Library

Searching other resources
To identify relevant grey literature, we searched the following databases and institutional websites:

1. Google Scholar
2. EconLit
3. IDEAS/RePEc
4. WHO Global Index Medicus
5. Pascal-Francis
6. Open-Grey
7. Grey Literature Report
8. Social Science Research Network (SSRN)
9. Eldis
10. GAVI
11. Epistemonikos
12. Innovations for Poverty Action (IPA)
13. Abdul Latif Jameel Poverty Action Lab (J-PAL)
14. 3ie Impact Evaluation Repository
15. 3ie Systematic Review Repository
16. Registry of International Development Impact Evaluations (RIDIE)
17. Global Development Network
18. World Bank Development Impact Evaluation (DIME) and Impact Evaluation Policy Papers
19. Inter-American Development Bank
20. Center for Global Development
21. Center for Effective Global Action (CEGA)
22. DFID Research for Development (R4D)
23. USAID

Search terms
L&MICs
1. (Afghanistan or Albania or Algeria or Angola or Argentina or Armenia or Armenian or Azerbaijan or Bangladesh or Benin or Byelarus or Byelorussian or Belarus or Belorussian or Belorussia or Belize or Bhutan or Bolivia or Bosnia or Herzegovina or Hercegovina or Botswana or Brazil or Bulgaria or Burkina Faso or Burkina Fasso or Upper Volta or Burundi or Urundi or Cambodia or Khmer Republic or Kampuchea or Cameroon or Cameroons or Cameron or Camerons or Cape Verde or Cabo Verde or Central African Republic or Chad or Tchad or China or Colombia or Comoros or Comoro Islands or Comores or Mayotte or Congo or Zaire or Costa Rica or Cote d'Ivoire or Ivory Coast or Cuba or Djibouti or French Somaliland or Dominica or Dominican Republic or East Timor or East Timur or Timor Leste or Ecuador or Egypt or United Arab Republic or El Salvador or Eritrea or Ethiopia or Fiji or Gabon or Gabonese Republic or Gambia or Gaza or Georgia Republic or Georgian Republic or Ghana or Grenada or Guatemala or Guinea or Guiana or Guyana or Haiti or Honduras or India or Maldives or Indonesia or Iran or Iraq or Jamaica or Jordan or Kazakhstan or Kazakh or Kenya or Kiribati or Korea or Kosovo or Kyrgyzstan or Kirghizia or Kyrgyz Republic or Kirghiz or Kirgizstan or Lao PDR or Laos or Lebanon or Lesotho or Basutoland or Liberia or Libya or Macedonia or Madagascar or Malagasy Republic or Malaysia or Malaya or Malay or Sabah or Sarawak or Malawi or Mali or Marshall Islands or Mauritania or Mauritius or Agalega Islands or Mexico or Micronesia or Middle East or Moldova or Moldovia or Moldovian or Mongolia or Montenegro or Morocco or Ifni or Mozambique or Myanmar or Myanma or Burma or Namibia or Nepal or Netherlands Antilles or Nicaragua or Niger or Nigeria or Muscat or Pakistan or Palau or Palestine or Panama or Paraguay or Peru or Philippines or Philipines or Phillipines or Phillippines or Papua New Guinea or Romania or

For peer review only

Rumania or Roumania or Rwanda or Ruanda or Saint Lucia or St Lucia or Saint Vincent or St Vincent or Grenadines or Samoa or Samoan Islands or Navigator Island or Navigator Islands or Sao Tome or Senegal or Serbia or Montenegro or Seychelles or Sierra Leone or Sri Lanka or Solomon Islands or Somalia or Sudan or Suriname or Surinam or Swaziland or Eswatini or South Africa or Syria or Tajikistan or Tadzhikistan or Tadjikistan or Tadzhik or Tanzania or Thailand or Togo or Togolese Republic or Tonga or Tunisia or Turkey or Turkmenistan or Turkmen or Uganda or Ukraine or Uzbekistan or Uzbek or Vanuatu or New Hebrides or Venezuela or Vietnam or Viet Nam or West Bank or Yemen or Zambia or Zimbabwe or Rhodesia)

2. (Africa or Asia or Caribbean or West Indies or South America or Latin America or Central America)
3. ((developing or less* developed or least developed or under developed or underdeveloped or middle income or low* income or underserved or under served or deprived or poor* or resource limited or resource constrained) adj (countr* or nation? or population? or world or state* or emerging econom* or global south))
4. ((developing or less* developed or least developed or under developed or underdeveloped or middle income or low* income or resource limited or resource constrained) adj (economy or economies))
5. (low* adj (gdp or gnp or gross domestic or gross national))
6. (low adj3 middle adj3 countr*)
7. (lmic or lmics or third world or lami countr*)
8. transitional countr*
9. 1 OR 2 OR 3 OR 4 OR 5 OR 6 OR 7

Immunisation

1. (immuniz* or immunis* or vaccin* or inoculat* or innoculat* or immunotherap* or prophyla*)

Young children and caregivers

24. (child* or infant* or newborn* or neonat* or prenatal or pre natal or antenatal or ante natal or baby or babies or toddler* or preschool* or parent* or mother* or father* or maternal or paternal or caregiver* or grandparent* or grandmother* or grandfather* or family member*)

Impact evaluation methods

12. (random* or experiment* or (match* adj2 (propensity or coarsened or covariate)) or "propensity score" or "difference in difference*" or "difference-in-difference*" or "differences in difference*" or "differences-in-difference*" or "double difference*" or "quasi-experimental" or "quasi experimental" or "quasi-experiment" or "quasi experiment" or ((estimator or counterfactual) and evaluation*) or "instrumental variable*" or (IV adj2 (estimation or approach)) or regression discontinuity or time series or segment* regression)

Example full search strategy

Below we present a draft of the full search strategy used to search MEDLINE. Note that in MEDLINE's syntax, terms with strokes (e.g., Immunization/) denote Medical Subject Heading (MeSH) terms, while strings appended with "ti,ab,kw" are searched in the title, abstract, and keyword fields of records in the database.

Ovid MEDLINE(R) and Epub Ahead of Print, In-Process & Other Non-Indexed Citations, Daily and Versions(R) <1946 to May 15, 2019> Searched 16th May 2019

1 (immuniz* or immunis* or vaccin* or inoculat* or innoculat* or immunotherap* or prophyla*).ti,ab,kw. (672732)

2 immunization/ or immunization, passive/ or immunization schedule/ or immunization, secondary/ or immunotherapy, active/ or vaccination/ or Immunization Programs/ or mass vaccination/ (158163)

3 Tuberculosis Vaccines/ or BCG Vaccine/ or Diphtheria-Tetanus Vaccine/ or Meningococcal Vaccines/ or Pertussis Vaccine/ or Diphtheria-Tetanus-acellular Pertussis Vaccines/ or Diphtheria-Tetanus-Pertussis Vaccine/ or Diphtheria-Tetanus Vaccine/ or Measles Vaccine/ or Mumps Vaccine/ or Rubella Vaccine/ or Measles-Mumps-Rubella Vaccine/ or Poliovirus Vaccines/ or Poliovirus Vaccine, Inactivated/ or Poliovirus Vaccine, Oral/ or Japanese Encephalitis Vaccines/ or Rotavirus Vaccine/ (49639)

4 or/1-3 (720583)

5 developing countries.sh,kf. (83271)

6 (Africa or Asia or Caribbean or West Indies or South America or Latin America or Central America).ti,ab,kw. (200981)

7 Africa/ or Asia/ or Caribbean/ or West Indies/ or South America/ or Latin America/ or Central America/ (73389)

8 (Africa or Central America or South America or Caribbean or Central Asia or Afghanistan or Albania or Algeria or Angola or Argentina or Armenia or Armenian or Azerbaijan or Bangladesh or Benin or Byelarus or Byelorussian or Belarus or Belorussian or Belorussia or Belize or Bhutan or Bolivia or Bosnia or Herzegovina or Hercegovina or Botswana or Brazil or Bulgaria or Burkina Faso or Burkina Fasso or Upper Volta or Burundi or Urundi or Cambodia or Khmer Republic or Kampuchea or Cameroon or Cameroons or Cameron or Camerons or Cape Verde or Cabo Verde or Central African Republic or Chad or Tchad or China or Colombia or Comoros or

For peer review only

Comoro Islands or Comores or Mayotte or Congo or Zaire or Costa Rica or Cote d'Ivoire or Ivory Coast or Cuba or Djibouti or French Somaliland or Dominica or Dominican Republic or East Timor or East Timur or Timor Leste or Ecuador or Egypt or United Arab Republic or El Salvador or Eritrea or Ethiopia or Fiji or Gabon or Gabonese Republic or Gambia or Gaza or Georgia Republic or Georgian Republic or Ghana or Grenada or Guatemala or Guinea or Guiana or Guyana or Haiti or Honduras or India or Maldives or Indonesia or Iran or Iraq or Jamaica or Jordan or Kazakhstan or Kazakh or Kenya or Kiribati or Korea or Kosovo or Kyrgyzstan or Kirghizia or Kyrgyz Republic or Kirghiz or Kirgizstan or Lao PDR or Laos or Lebanon or Lesotho or Basutoland or Liberia or Libya or Macedonia or Madagascar or Malagasy Republic or Malaysia or Malaya or Malay or Sabah or Sarawak or Malawi or Mali or Marshall Islands or Mauritania or Mauritius or Agalega Islands or Mexico or Micronesia or Middle East or Moldova or Moldovia or Moldovian or Mongolia or Montenegro or Morocco or Ifni or Mozambique or Myanmar or Myanma or Burma or Namibia or Nepal or Netherlands Antilles or Nicaragua or Niger or Nigeria or Muscat or Pakistan or Palau or Palestine or Panama or Paraguay or Peru or Philippines or Philipines or Phillipines or Phillippines or Papua New Guinea or Romania or Rumania or Roumania or Rwanda or Ruanda or Saint Lucia or St Lucia or Saint Vincent or St Vincent or Grenadines or Samoa or Samoan Islands or Navigator Island or Navigator Islands or Sao Tome or Senegal or Serbia or Montenegro or Seychelles or Sierra Leone or Sri Lanka or Solomon Islands or Somalia or Sudan or Suriname or Surinam or Swaziland or Eswatini or South Africa or Syria or Tajikistan or Tadzhikistan or Tadjikistan or Tadzhik or Tanzania or Thailand or Togo or Togolese Republic or Tonga or Tunisia or Turkey or Turkmenistan or Turkmen or Uganda or Ukraine or Uzbekistan or Uzbek or Vanuatu or New Hebrides or Venezuela or Vietnam or Viet Nam or West Bank or Yemen or Zambia or Zimbabwe or Rhodesia).ti,ab,kw,sh. (1387951)

9 ((developing or less* developed or least developed or under developed or underdeveloped or middle income or low* income or underserved or under served or deprived or poor* or resource limited or resource constrained) adj (countr* or nation? or population? or world or state*)).ti,ab,kw. (91955)

10 ((developing or less* developed or least developed or under developed or underdeveloped or middle income or low* income or resource limited or resource constrained) adj (economy or economies)).ti,ab,kw. (504)

11 (low* adj (gdp or gnp or gross domestic or gross national)).ti,ab,kw. (234)

12 (low adj3 middle adj3 countr*).ti,ab,kw. (12819)

13 (lmic or lmics or third world or lami countr*).ti,ab,kw. (6486)

14 (transitional countr* or emerging econom* or global south).ti,ab,kw. (873)

15 or/5-14 (1487811)

16 4 and 15 (80257)

17 Parents/ or Fathers/ or Mothers/ or Grandparents/ or Caregivers/ or Single Parent/ or Pregnant Women/ or Child, Preschool/ or Infant/ or Infant, Newborn/ or Infant, Low Birth Weight/ or Infant, Small for Gestational Age/ or Infant, Very Low Birth Weight/ or Infant, Extremely Low Birth Weight/ or Infant, Postmature/ or Infant, Premature/ or Infant, Extremely Premature/ (1597959)

18 (child* or infant* or newborn* or neonat* or neo nat* or prenatal or pre natal or ante natal or antenatal or baby or babies or toddler* or preschool* or parent* or mother* or father* or maternal or paternal).ti,ab,kw. (2284298)

19 or/17-18 (2893768)

20 16 and 19 (28515)

21 (random* or experiment* or (match* adj2 (propensity or coarsened or covariate)) or "propensity score" or ("difference in difference*" or "difference-in-difference*" or "differences in difference*" or "differences-in-difference*" or "double difference*") or ("quasi-experimental" or "quasi experimental" or "quasi-experiment" or "quasi experiment") or ((estimator or counterfactual) and evaluation*) or "instrumental variable*" or (IV adj2 (estimation or approach)) or regression discontinuity or time series or segment* regression).ti,ab,kw. (2983909)

22 Randomized Controlled Trial/ or Random Allocation/ or Evaluation Studies/ or Propensity Score/ or Interrupted Time Series Analysis/ or Controlled Before-After Studies/ or Controlled Clinical Trial/ or Non-Randomized Controlled Trials as Topic/ (896249)

23 or/21-22 (3415351)

24 Cost Analysis/ or Cost-Benefit Analysis/ or Quality-Adjusted Life Years/ or Economics, Medical/ or Cost of Illness/ or Health Care Costs/ or Direct Service Costs/ or Budgets/ or Health Care Sector/ or Public Expenditures/ (193420)

25 (cost-effective* or cost-benefit).ti,ab,kw. (130711)

26 ("life year" or "life years" or qaly* or daly*).ti,ab,kw. (18749)

27 ((economic* or cost*) adj6 (mortality or death* or markov)).ti,ab,kw. (17246)

28 ("cost minimi*" or "cost-utilit*" or "economic evaluation*" or "economic review*" or "cost outcome" or "cost analys*" or "economic analys*" or "budget* impact analys*").ti,ab,kw. (27594)

29 or/24-28 (301485)

30 (review or meta-analysis).pt. (2557796)

31 meta-analysis/ or "systematic review"/ (163880)

32 cochrane database of systematic reviews.jn. (14162)

33 (systematic review or literature review).ti. (130870)

34 or/30-33 (2590211)

For peer review only

35	23 or 29 or 34 (5946083)
36	20 and 35 (8309)
37	exp Animals/ (22312530)
38	Humans/ (17732234)
39	37 not (37 and 38) (4580296)
40	36 not 39 (7984)

For peer review only

Appendix 4: Data extraction template

1) General Information

Sr. Number	
Study ID	
Short title	
Minutes taken to complete data extraction for this study	
Who's completing this form?	
Study title	
Year of publication	
Publication type	Journal article
	Published report
	Published working paper
	Thesis
Journal name/ publisher	
Journal Volume	
Journal Issue	
Research funding agency category	Academic institutions
	Charitable or private foundations
	Government agencies
	International aid agencies
	International financial institutions
	Non-profit organisation
	Not specified
Research funding agency name	
Included geographies	Sub-Saharan Africa
	South Asia
	East Asia and Pacific
	Latin America & the Caribbean
	Middle East & North Africa
	Eastern Europe and Central Asia
	North America
	Western Europe
	Global
	Unclear / unspecified
	If the review focuses on only one country, specify it
Location of intervention	Urban areas
	Rural areas
	Urban and rural areas
	Not reported
Any other relevant information you'd like to add for this section?	
Overarching main research question or primary hypothesis	
Includable comparators	No intervention
	"Business as usual" (existing ongoing treatment)
	Alternative intervention
	Placebo intervention
	No information on includable comparators provided
Search and inclusion	Have academic databases been searched?
	Have grey literature sites been searched?
	Dates of search reported?
	Was the review part of the update?
	PRISMA flow diagram presented?
	Number of included studies
	List of excluded studies provided?
	Explanations for exclusions provided?
Types of studies included, by study design	Quantitative studies included
	Qualitative studies included
	Mixed-methods studies included
	Unclear or information not provided
	Experimental design / RCT

For peer review only

Types of quantitative study designs included	Pipeline design
	Panel design or before/after AND with/without
	Either before/after OR with/without
	Natural experiment
	Basic survey
	Unclear or information not provided
Types of qualitative study designs included	Ethnographic study design
	Semi-structured interviews
	Narrative or life history
	Visual and/or participatory methods
	Documentary and/or content analysis
	Focus group discussions
	Unclear or information not provided
Types of quantitative analysis techniques applied in included studies	Econometric techniques (IV, PSM, 2SLS, DID, RDD, etc.)
	Multivariable analysis (OLS / regression-based approaches)
	Cross-tabulation / descriptive statistics
	Unclear or information not provided
Types of qualitative analysis techniques applied in included studies	Content or framework analysis
	Grounded theory or IPA
	Discourse analysis
	Narrative analysis
	Thematic analysis
	Unclear or information not provided
	Any other relevant information you'd like to add for this section?
What type of QUANTITATIVE data synthesis mechanisms were applied in the systematic review/meta-analysis?	Meta-regression
	Meta-analysis
	Description of range of effect sizes
	Vote counting - statistical significance
	Vote counting - direction of effect
	Descriptive (usually for narrative synthesis)
	Unclear or information not provided
What type of QUALITATIVE data synthesis mechanisms were applied in the systematic review/meta-analysis?	Narrative synthesis
	Thematic synthesis
	Meta-ethnography
	Context-mechanism analysis
	Qualitative comparative analysis
	Unclear or information not provided
Subgroup analysis	Was subgroup analysis conducted?
	Please briefly describe the nature of the subgroup analysis
Publication bias and funnel	Did the authors include a discussion of publication bias?
	Were funnel plots reported?
	Were Funnel Asymmetry and Precision Effects tests reported?
	Did the FAT-PET show evidence of publication bias? (Specific test for meta-regression)
	Any other relevant information you'd like to add for this section?
Other	Does the systematic review/meta-analysis provide a GRADE rating?
	What are the quality assessment tool(s) / risk of bias procedure in the study?
	Has the cost effectiveness analysis of the intervention(s) been included?
	If yes, what was the main cost measure that was used/calculated? (e.g., total intervention costs, intervention costs per person, incremental costs, incremental cost-effectiveness ratios (ICER) etc.)
	Did the study do sensitivity analysis?
	If yes, describe the kind of sensitivity analysis done. If no, what was the reason if acknowledged.
	Limitations of the systematic review/meta-analysis acknowledged by the authors

2) Outcome data

Intervention Code (from immunisation EGM)

For peer review only

Intervention description
Intervention notes
Outcome code (from immunisation EGM)
Outcome description
Outcome notes
Further information about outcome classification
Unit of measurement
Sample size (number of studies and number of participants)
Population level analysed
Sub-population(s) analysed
Direction of outcome (favourable, unfavourable, inconclusive)
Are meta-analysis regression results presented for this outcome?
How many effect sizes are reported for this outcome?
Summary of the key conclusions drawn
Narrative or other type of synthesis trend result
Narrative or other type of synthesis summary quote
Any other relevant information you'd like to add for this section?
Grade rating
Source (page number)

3) Meta-analysis data

Quantitative data extraction
Intervention Code (from immunisation EGM)
Outcome code (from immunisation EGM)
Dependent variable name for the effect size (exact terminology as listed in the systematic review/meta-analysis)
Type of effect size reported
Effect size (the actual figure)
Significance level
Confidence interval (95%)
Sample size (number of studies)
Heterogeneity
Did the authors describe likely explanatory factors of heterogeneity?
If so, please note the key factors put forward
Was a sensible method used to explore the extent to which key factors explained heterogeneity?
If so, which
If applicable, mention the test for heterogeneity
If applicable, provide heterogeneity statistics
If applicable, provide confidence interval of heterogeneity statistics
If meta-analysis is done by subgroup, then describe it
Effect size by subgroup, actual figure
Confidence interval of effect size by subgroup
Source (page number, table number, etc.)
Additional notes

For peer review only

Appendix 5: Quality appraisal tool

Checklist for making judgements about how much confidence to place in a systematic review of effects (adapted version of SURE checklist)

Assessed by:
Date:

Section A: *Methods used to identify, include and critically appraise studies*

A.1 Were the criteria used for deciding which studies to include in the review reported?<br>Did the authors specify:<br>□ Did the authors specify Types of studies?<br>□ Did the authors specify Participants/ settings/ population?<br>□ Did the authors specify Intervention(s)?<br>□ Did the authors specify Outcome(s)?	□ Yes<br>□ Partially<br>□ No<br>*Coding guide - check the answers above*<br>*YES: All four should be yes*<br>*NO: All four should be no*<br>*PARTIALLY: Any other*

For peer review only

A.2 Was the search for evidence reasonably comprehensive?<br>Were the following done:<br>□ Language bias avoided (no restriction of inclusion based on language)<br>□ No restriction of inclusion based on publication status<br>□ Relevant databases searched<br><u>Minimum criteria</u>: at least one database that includes grey/unpublished literature, as well as either: (a) for health, at least two relevant comprehensive subject databases (such as PubMed/MEDLINE, EMBASE and CENTRAL), or (b) for social sciences, at least two relevant comprehensive subject databases (such as IDEAS) and one comprehensive general database (such as EconLit, PsychInfo, Scopus. If the search is for studies in China, the CNKI (China National Knowledge infrastructure) is acceptable as a comprehensive database.<br>□ Reference lists in included articles checked<br>□ Authors/experts contacted	□ Yes<br>□ Partially<br>□ No<br>□ Can't tell<br>*Coding guide - check the answers above:*<br>*YES: All five should be yes*<br>*PARTIALLY: Relevant databases and reference lists are both reported*<br>*NO: Any other*<br>Note: Grey literature typically means research that is not published in sources such as books or journal articles. The following databases include grey literature: Academic Search Complete (includes many conference proceedings), CAB Abstracts, search conducted using CADATH checklist, clinicaltrials.gov, Cochrane Central Register of Controlled Trials (CENTRAL), Cochrane Library, Embase (includes 3.6m+ conference abstracts), Google, Google Scholar, Healthcare Management Information Consortium (HMIC), IDEAS/RePEc, National Technical Information Service (NTIS), OpenSIGLE/OpenGrey, PsycEXTRA, Scopus (includes ~10m conference papers). If you identify additional sources, please add to the list. Searching websites of relevant governmental agencies and non-governmental organisations or other organisations such as the WHO can also identify grey literature. Note that MEDLINE/PubMed, a comprehensive data base of journals, does not include grey literature: "For indexing in MEDLINE, NLM currently selects publications that it considers to be journals."; see also Citrome L. Beyond PubMed: Searching the "Grey Literature" for Clinical Trial Results. Innov Clin Neurosci. 2014;11(7-8):42-46.

For peer review only

A.3 Does the review cover an appropriate time period?<br>*Is the search period comprehensive enough that relevant literature is unlikely to be omitted?*	□ Yes<br>□ Can't tell<br>□ No<br>*Coding guide:*<br>*YES: Generally, this means searching the literature at least back to 1990*<br>*NO: Generally, if the search does not go back to 1990*<br>*CAN'T TELL: No information about time period for search*<br>*Note: With reference to the above – there may be important reasons for adopting different dates for the search, e.g., depending on the intervention. If you think there are limitations with the timeframe adopted for the search which have not been noted and justified by the authors, you should code this item as a NO and specify your reason for doing so in the comment box below. Older reviews should not be downgraded, but the fact that the search was conducted some time ago should be noted in the quality assessment. Always report the time period for the search in the comment box. If the authors do not report the search period, check the publication date of the earliest included study. If the study was published before 1990 this can be coded Yes.*
A.4 Was bias in the selection of articles avoided?<br>Did the authors specify:<br>□ Independent screening of full text by at least 2 reviewers<br>□ List of included studies provided<br>□ List of excluded studies provided	□ Yes<br>□ Partially<br>□ No<br>*Coding guide:*<br>*YES: All three should be yes, although reviews published in journals are unlikely to have a list of excluded studies (due to limits on word count) and the review should not be penalised for this.*<br>*PARTIALLY: Independent screening and list of included studies provided are both reported*<br>*NO: All other. If list of included studies provided, but the authors do not report whether or not the screening has been done by 2 reviewers review is downgraded to NO.*<br>*Note: The answer linked to list of included studies provided criteria is from the question "Was a table or summary of the results of the included studies provided?" in the data extraction form.*

For peer review only

A.5 Did the authors use appropriate criteria to assess the quality and risk of bias in analysing the studies that are included?<br>□ The criteria used for assessing the quality/ risk of bias were reported<br>□ A table or summary of the assessment of each included study for each criterion was reported<br>□ Sensible criteria were used that focus on the quality/ risk of bias (and not other qualities of the studies, such as precision or applicability/external validity).<br>*"Sensible" is defined as a recognised quality appraisal tool/ checklist, or similar tool which assesses bias in included studies. Please see footnotes for details of the main types of bias such a tool should assess.*<br>*Identified tools with sensible criteria include: Academy of Nutrition and Dietetics Quality Criteria Checklist, Child Health Epidemiology Reference Group (CHERG) study design & quality standards , Cochrane Handbook, The Delphi List, Effective Public Health Practice Project (EPHPP) Quality Assessment Tool, Grading of Recommendations Assessment, Development and Evaluation (GRADE) RoB criteria, Guide to Community Preventative Services Study Quality tool, Joanna Briggs Institute Checklists for RCT/QED, National Institutes of Health's Quality Assessment Tool for Controlled Intervention Studies (sometimes labelled NHLBI tool). For case-control studies and cohort studies, the Newcastle-Ottawa Scale uses sensible criteria that are focused on risk of bias as does Methodological Index for Non-Randomised Studies (MINORS). Note that these designs typically are not as rigorous as RCTs or even QEDs.*	□ Yes<br>□ Partially<br>□ No<br>*Coding guide:*<br>*YES: All three should be yes*<br>*PARTIALLY: The first and third criteria should be reported. If the authors report the criteria for assessing risk of bias and report a summary of this assessment for each criterion, but the criteria may be only partially sensible (e.g., do not address all possible risks of bias, but do address some), we downgrade to PARTIALLY.*<br>*NO: Any other*
A.6 Overall – how much confidence do you have in the methods used to identify, include and critically appraise studies?<br>*Summary assessment score A relates to the 5 questions above.*<br>*High confidence applicable when the answers to the questions in section A are all assessed as 'yes'*<br>*Low confidence applicable when any of the following are assessed as 'NO' above: not reporting explicit selection criteria (A1), not conducting reasonably comprehensive search (A2), not avoiding bias in selection of articles (A4), not assessing the risk of bias in included studies (A5)*<br>*Medium confidence applicable for any other – i.e. section A3 is assessed as 'NO' or can't tell and remaining sections are assessed as 'partially' or 'can't tell'*	□ Low confidence (limitations are important enough that the results of the review are not reliable)<br>□ Medium confidence (limitations are important enough that it would be worthwhile to search for another systematic review and to interpret the results of this review cautiously, if a better review cannot be found)<br>□ High confidence (only minor limitations)

Section B: *Methods used to analyse the findings*

For peer review only

B.1 Were the characteristics and results of the included studies reliably reported? Was there:<br>□ Independent data extraction by at least 2 reviewers<br>□ A table or summary of the characteristics of the participants, interventions and outcomes for the included studies (Forest plots are an appropriate summary of the results, as is reporting summarises the findings by outcome domain.)<br>□ A table or summary of the results of all the included studies	□ Yes<br>□ No<br>□ Partially<br>□ Not applicable (e.g., no included studies)<br>*Coding guide:*<br>*YES: All three should be yes*<br>*PARTIALLY: Criteria one and three are yes, but some information is lacking on second criteria.*<br>*No: None of these are reported. If the review does not report whether data was independently extracted by 2 reviewers (possibly a reporting error), we downgrade to NO.*<br>*NOT APPLICABLE: if no studies/no data*<br>*Note: Independent extraction means that both extractors extracted all data without knowing what the other extractor decided (that is, one extractor and one verifier does not meet criterion). If the authors note two reviewers and do not use the word "independent" but mention a third reconciler to resolve differences, assume independence. When authors do not mention whether independent extraction was conducted by at least two reviewers, code No.*
B.2 Are the methods used by the review authors to analyse the findings of the included studies clear, including methods for calculating effect sizes if applicable?	□ Yes<br>□ Partially<br>□ No<br>□ Not applicable (e.g., no studies or no data)<br>*Coding guide:*<br>*YES: Methods used clearly reported. If it is clear that the authors use narrative synthesis, they don't need to say this explicitly.*<br>*PARTIALLY: Some reporting on methods but lack of clarity*<br>*NO: Nothing reported on methods*<br>*NOT APPLICABLE: if no studies/no data*

For peer review only

B.3 Did the review describe the extent of heterogeneity?<br>□ Did the review ensure that included studies were similar enough that it made sense to combine them, sensibly divide the included studies into homogeneous groups, or sensibly conclude that it did not make sense to combine or group the included studies?<br>□ Did the review discuss the extent to which there were important differences in the results of the included studies?<br>□ If a meta-analysis was done, was the I^2, chi square test for heterogeneity or other appropriate statistic reported? If no statistical test was reported, is a qualitative justification made for the use of random effects?	□ Yes<br>□ Partially<br>□ No<br>□ Not applicable (e.g., no studies or no data)<br><br>*Coding guide:*<br>*YES: First two should be yes, and third category should be yes if applicable should be yes*<br>*PARTIALLY: The first category is yes*<br>*NO: Any other*<br>*NOT APPLICABLE: if no studies/no data*<br>*Note: Code B3.1 No if analyses include studies with implausibly different interventions, comparisons, or populations. If a narrative synthesis, the authors need to have a rationale for why studies were not combined (such as interventions were too different) For meta-analyses, reporting a metric for heterogeneity is sufficient for B3.2. For non-meta-analysis, mentioning heterogeneity in results is enough (for example, The impacts varied from X to Y or Study A found X and Study B found Y).*

For peer review only

B.4 Were the findings of the relevant studies combined (or not combined) appropriately relative to the primary question the review addresses and the available data?<br>How was the data analysis done?<br>□ Descriptive only<br>□ Vote counting based on direction of effect<br>□ Vote counting based on statistical significance<br>□ Description of range of effect sizes<br>□ Meta-analysis<br>□ Meta-regression<br>□ Other: specify<br>□ Not applicable (e.g., no studies or no data)<br>How were the studies weighted in the analysis?<br>□ Equal weights (this is what is done when vote counting is used)<br>□ By quality or study design (this is rarely done)<br>□ Inverse variance (this is what is typically done in a meta-analysis)<br>□ Number of participants (sample size)<br>□ Other: specify<br>□ Not clear<br>□ Not applicable (e.g., no studies or no data)<br>Did the review address unit of analysis errors?<br>□ Yes - took clustering into account in the analysis (e.g., used intra-cluster correlation coefficient)<br>□ No, but acknowledged problem of unit of analysis errors<br>□ No mention of issue<br>□ Not applicable - no clustered trials or studies included	□ Yes<br>□ Partially<br>□ No<br>□ Not applicable (e.g., no studies or no data)<br>□ Can't tell<br>*Coding guide:*<br>*YES: If appropriate table, graph or meta-analysis AND appropriate weights AND unit of analysis errors addressed (if appropriate).*<br>*PARTIALLY: If appropriate table, graph or meta-analysis AND appropriate weights AND unit of analysis errors not addressed (and should have been).*<br>*NO: If narrative OR vote counting (where quantitative analyses would have been possible) OR inappropriate reporting of table, graph or meta-analyses.*<br>*NOT APPLICABLE: if no studies/no data*<br>*CAN'T TELL: if unsure (note reasons in comments below)*

For peer review only

B. 5 Does the review report evidence appropriately?<br>□ The review makes clear which evidence is subject to low risk of bias in assessing causality (attribution of outcomes to intervention), and which is likely to be biased, and does so appropriately<br>□ Where studies of differing risk of bias are included, results are reported and analysed separately by risk of bias status (No)	□ Yes<br>□ No<br>□ Partially<br>□ Not applicable<br>*Coding guide:*<br>*YES: Both criteria should be fulfilled (where applicable)*<br>*NO: Criteria not fulfilled*<br>*PARTIALLY: Only one criterion fulfilled, or when there is limited reporting of quality appraisal (the latter applies only when inclusion criteria for study design are appropriate)*<br>*NOT APPLICABLE: No included studies*<br>*Note on reporting evidence and risk of bias: For reviews of effects of 'large n' interventions, experimental and quasi-experimental designs should be included (if available). For reviews of effects of 'small n' interventions, designs appropriate to attribute changes to the intervention should be included (e.g., pre-post with assessment of confounders)*<br>*Making clear which evidence is subject to low risk of bias can be accomplished in a table listing RoB for each study or by listing RoB for each study on each RoB criterion; that is, if A5.2 is Yes, then B5.1 is Yes (but the reverse is not true). Reporting only study design is not sufficient to meet B5.1. For B5.2, narrative analysis must group or report by RoB, it is not sufficient to simply report RoB of each study. If the* systematic review *does not use sensible criteria to assess RoB, then B5.1 is No.*
B.6 Did the review examine the extent to which specific factors might explain differences in the results of the included studies?<br>□ Were factors that the review authors considered as likely explanatory factors clearly described?<br>□ Was a sensible method used to explore the extent to which key factors explained heterogeneity?<br>□ Descriptive/textual<br>□ Graphical<br>□ Meta-analysis by subgroups<br>□ Meta-regression<br>□ Other	□ Yes<br>□ Partially<br>□ No<br>□ Not applicable<br>*Coding guide:*<br>*YES: Explanatory factors clearly described and appropriate methods used to explore heterogeneity*<br>*PARTIALLY: Explanatory factors described but for meta-analyses, subgroup analysis or meta-regression not reported (when they should have been)*<br>*NO: No description or analysis of likely explanatory factors*<br>*NOT APPLICABLE: e.g., too few studies, no important differences in the results of the included studies, or the included studies were so dissimilar that it would not make sense to explore heterogeneity of the results*

For peer review only

B.7 Overall - how much confidence do you have in the methods used to analyse the findings relative to the primary question addressed in the review?<br>*Summary assessment score B relates to the 5 questions in this section, regarding the analysis.*<br>*High confidence applicable when all the answers to the questions in section B are assessed as 'yes'.*<br>*Low confidence applicable when any of the following are assessed as 'NO' above: critical characteristics of the included studies not reported (B1), not describing the extent of heterogeneity (B3), combining results inappropriately (B4), reporting evidence inappropriately (B5).*<br>*Medium confidence applicable for any other: i.e., the "Partial" option is used for any of the 6 preceding questions or questions and/or B.2 and/ or B.6 are assessed as 'no'.*	□ Low confidence (limitations are important enough that the results of the review are not reliable)<br>□ Medium confidence (limitations are important enough that it would be worthwhile to search for another systematic review and to interpret the results of this review cautiously, if a better review cannot be found)<br>□ High confidence (only minor limitations)

For peer review only

Section C: Overall assessment of the reliability of the review

C.1 Are there any other aspects of the review not mentioned before which lead you to question the results?	□ Additional methodological concerns – only one person reviewing<br>□ Robustness<br>□ Interpretation<br>□ Conflicts of interest (of the review authors or for included studies)<br>□ Other<br>□ No other quality issues identified
C.2 Are there any mitigating factors which should be taken into account in determining the reviews reliability?	□ Limitations acknowledged<br>□ No strong policy conclusions drawn (including in abstract/ summary)<br>□ Any other factors
C.3 Based on the above assessments of the methods how would you rate the reliability of the review?<br>□ <u>Low confidence in conclusions about effects</u>:<br>This review differs from the typical effectiveness review in that it combines evaluations with descriptive studies, and has no methodological exclusion criteria. Moreover, the authors do not perform any sort of risk of bias assessment on the evaluative studies, nor clearly report the methods used in included studies, making it difficult to know how much confidence to place in the results. There are major gaps in the reporting of the search (databases not listed) and screening (no discussion of how this was done). The authors do note the limitations of the review, including the language restrictions and the limited reporting on pre- and post-intervention coverage rates in the included studies.<br>□ <u>Medium confidence in conclusions about effects</u>:<br>The systematic review has the following limitations.<br>□ <u>High confidence in conclusions about effects</u>:<br>If applicable: The review has the following minor limitations...<br>*Coding guide:*<br>High confidence in conclusions about effects: high confidence noted overall for sections A and B, unless moderated by answer to C1.<br>Medium confidence in conclusions about effects: medium confidence noted overall for sections A or B, unless moderated by answer to C1 or C2.<br>Low confidence in conclusions about effects: low confidence noted overall for sections A or B, unless moderated by answer to C1 or C2.<br>Limitations should be summarised above, based on what was noted in Sections A, B and C.	

Section D

D.1. Does the review use a programme theory?<br>□ Did the authors present: A logic model articulating the intervention causal chain from inputs/activities through to outcomes?<br>□ Did the authors present: Assumptions, either in the logic model itself or discussed in supporting text?<br>□ Did the authors present: A (middle-range) theory (economic theory, e.g. trade theory; social theory, e.g. diffusion theory; etc.) which informed the logic models and/or from which inferences can be made about mechanisms and contexts under which outcomes might occur?	□ Yes<br>□ Partially<br>□ No<br>*Coding guide:*<br>*YES: Some theory is used, whether an intervention level logic model or causal chain, or formal theory, and underlying assumptions are explicitly described.*<br>*NO: None are reported.*<br>*PARTIALLY: A theory is used but underlying assumptions are not reported.*

For peer review only

D.2. Does the review incorporate qualitative evidence in the design?<br>□ Was the logic model or theory articulated at protocol stage?<br>□ Is qualitative evidence cited in the development or explanation of the theoretical approach/logic model?<br>□ Is qualitative evidence incorporated systematically (i.e., based on systematic searches), in order to develop the theoretical approach/logic model?<br>□ Is qualitative evidence cited in development of relevant questions?	□ Yes<br>□ Partially<br>□ No<br>*Coding guide:*<br>*YES: At least 1 and 2 or 3 are reported.*<br>*NO: None are reported.*<br>*PARTIALLY: 1 or 4 are reported.*
D.3. Did the review conduct analysis of intermediate and endpoint outcomes along causal chain?<br>□ Did the review conduct analysis of primary endpoint outcomes?<br>□ Did the review conduct analysis of primary intermediate outcomes?<br>□ Did the review conduct analysis of secondary endpoint outcomes?<br>□ Did the review conduct analysis of secondary intermediate outcomes?	□ Yes<br>□ Partially<br>□ No<br>□ Not applicable<br>□ Can't tell<br>*Coding guide:*<br>*YES: Boxes 1 and 2 are ticked*<br>*PARTIALLY: Boxes 1 and 4 or 2 and 3 are ticked.*<br>*NO: analysis of outcomes along causal chain is not undertaken and only endpoint outcomes are analysed (and outcomes at different stages of the causal chain were excluded).*<br>*NOT APPLICABLE: if no studies/no data*<br>*CAN'T TELL: if unsure (note reasons in comments below)*
D.4. Does the review incorporate qualitative evidence in the analysis?<br>□ To answer specific review questions about intervention design (e.g., project portfolio information)<br>□ To answer specific review questions about barriers and facilitators/enablers of implementation (assumptions or risks in the causal chain/ logic model)<br>□ To answer specific review questions about adherence or participant views?<br>□ To provide information on context for included quantitative studies (e.g., moderators or implementation fidelity)?<br>□ To provide evidence on 'middle-range' causal mechanisms or contextual factors (e.g., policy context, second order changes e.g., general equilibrium effects, sustained adherence)	□ Yes<br>□ Partially<br>□ No<br>*Coding guide:*<br>*YES: 1, 2, or 3 plus 4 or 5 are reported.*<br>*NO: None are reported.*<br>*PARTIALLY: Any other combination.*

For peer review only

D.5. Does the review incorporate qualitative evidence in other aspects of the analysis?<br>□ To assess applicability of synthesised evidence to particular contexts?<br>□ To discuss the quantitative findings in light of qualitative evidence, drawing on qualitative evidence collected systematically?<br>□ To discuss the quantitative findings in light of qualitative evidence (i.e., in the discussion section only), not drawing on evidence collected systematically?	□ Yes<br>□ Partially<br>□ No<br>*Coding guide:*<br>*YES: 1 or 2 are reported.*<br>*NO: None are reported.*<br>*PARTIALLY: 3 is reported.*
D.6. Does the review integrate the findings from quantitative and qualitative evidence?<br>□ Is qualitative evidence: Presented in a separate results section?<br>□ Is qualitative evidence: Integrated with evidence on effects using qualitative methods (e.g., further iterations of the theory of change)<br>□ Is qualitative evidence: Integrated with evidence on effects using quantitative methods (e.g. through formal statistical testing of moderators and subgroups identified in qualitative analysis)<br>□ Is qualitative evidence: Some other method to integrate the evidence? (specify)	□ Yes<br>□ Partially<br>□ No<br>*Coding guide:*<br>*YES: 1 and 2 or 3 are reported.*<br>*NO: None are reported.*<br>*PARTIALLY: 1 is reported only.*
D.7. Is quantitative and qualitative evidence integrated to form conclusions and implications?<br>□ Weight of quantitative evidence assessed using GRADE or other methods<br>□ Weight of qualitative evidence assessed using cerQUAL or other methods<br>□ Some method is used to integrate findings from quantitative and qualitative approaches (e.g. summary of findings table) to determine conclusions and implications?	□ Yes<br>□ Partially<br>□ No<br>*Coding guide:*<br>*YES: All are reported.*<br>*NO: None are reported.*<br>*PARTIALLY: Only 1, 2 or 3 are reported.*
Overall - how much confidence do you have in the causal chain used in the review to analyse studies and the type of evidence incorporated to inform the analysis and reporting?	□ Low confidence<br>□ Medium confidence<br>□ High confidence

For peer review only

Notes:

i. Adapted from the Specialist Unit for Review Evidence (SURE) 2018. Questions to assist with the critical appraisal of systematic reviews available at: https://www.cardiff.ac.uk/__data/assets/pdf_file/0007/1142962/SURE-CA-form-for-SR_2018.pdf

ii. **Risk of bias** is the extent to which bias may be responsible for the findings of a study.

Bias is a systematic error or deviation from the truth in results or inferences. In studies of the effects of social, economic and health care interventions, the main types of bias arise from systematic differences in the groups that are compared (selection bias), the intervention that is provided, or exposure to other factors apart from the intervention of interest (performance bias/contamination), withdrawals or exclusions of people entered into a study (attrition bias) or how outcomes are assessed (detection bias) and reported (reporting bias). Reviews of social science studies may be particularly affected by reporting bias, where a biased subset of all the relevant data and analyses is presented.

Assessments of the risk of bias are sometimes also referred to as assessments of the **validity** or **quality** of a study.

Validity is the extent to which a result (of a measurement or study) is likely to be true.

Quality is a vague notion of the strength or validity of a study, often indicating the extent of control over bias.

For peer review only

Appendix 6: Summary of included systematic reviews

Table 6.1. Moderate and high confidence systematic reviews

Authors & Year	Source	Type of review	Geographic focus		No. primary studies	Types of intervention included	Types of outcomes included	Population level analysed
			No. of regions	Regions				
High confidence reviews (18 in total)								
Diaconu et al., 2021[52]	Journal article	Systematic review	4	Sub-Saharan Africa, South Asia, East Asia and Pacific, Latin America & the Caribbean	59	BD5. Pay-for-performance schemes	JAA15. Vaccination coverage (unspecified); JAA01. Full routine immunisation for children; JAA02. BCG; JAA05. DPT3; JAA11. Measles; JAA09. OPV3; IAA1. Community HW motivation, capacity & performance; ICA1. Stockouts; HAD4. Experience and satisfaction with health services	Child; Patients; Health facility; Healthcare workers
Eze, Lawani, & Acharya, 2021[44]	Journal article	Systematic review & Meta-analysis	4	Sub-Saharan Africa, South Asia, East Asia and Pacific, Latin America & the Caribbean	18	AB4. Written or pictorial messages (SMS, stickers, flyers etc.) to caregivers	JAA15. Vaccination coverage (unspecified); JAA13. Vaccination. Timeliness	Child
Gera et al., 2016[53]	Journal article	Systematic review & Meta-analysis	2	Sub-Saharan Africa, South Asia	4	BA1. Formal health worker training and education; BF1. Health system strategic planning	JAA11. Measles; JAA05. DPT3	Child
Glenton et al., 2011[54]	Journal article	Systematic review & Meta-analysis	7	Sub-Saharan Africa, South Asia, East Asia and Pacific, Latin America & the Caribbean, Eastern Europe and Central Asia, North America, Western Europe	12	AA2. One-time sensitization and education campaigns; BB7. Home visits; BB4. Promoting outreach to vaccine-hesitant groups; BC1. National/sub-national immunisation days; BB7. Home visits	JAA01. Full routine immunisation for children	Child

For peer review only

Authors & Year	Source	Type of review	Geographic focus		No. primary studies	Types of intervention included	Types of outcomes included	Population level analysed
			No. of regions	Regions				
Jain et al., 2022[41, 42][2]	Journal article	Systematic review & Meta-analysis	3	Sub-Saharan Africa, South Asia, Latin America & the Caribbean	61	F. Multicomponent; AB1. Material/ monetary incentives for caregivers; AB4. Written or pictorial messages (SMS, stickers, flyers etc.) to caregivers; CA1. Faith-based outreach/outreach using local leaders; DA1. Collaborating with whole community; BB5. Outreach to vulnerable populations (hard-to-reach, SES, caste, etc.); DA1. Collaborating with whole community; BD5. Pay-for-performance schemes; BD5. Pay-for-performance schemes; BF1. Health system strategic planning	JAA01. Full routine immunisation for children; JAA05. DPT3; JAA11. Measles; JAA13. Vaccination. Timeliness; JAA12. No vs. partial routine immunisation; JAA02. BCG; JAA03. DPT1; JAA04. DPT2; JAA06. OPV0; JAA07. OPV1; JAA08. OPV2; JAA09. OPV3; JAA14. Drop out rate for multidose vaccines; HAA1. Knowledge about immunisation; HAA2. Attitudes about immunisation; HAD5. Vaccination health card availability/ retention; HAD4. Experience and satisfaction with health services; IAB3. Formal HW motivation, capacity & performance	Child; Caregivers; Healthcare workers
Johri et al., 2015[55]	Journal Article	Systematic review & Meta-analysis	3	Sub-Saharan Africa, South Asia, Latin America & the Caribbean	11	F. Multicomponent; AA2. One-time sensitization and education campaigns; AB. Incentives & motivation	JAA15. Vaccination coverage (unspecified)	Child
Kaufman et al., 2018[56]	Journal article	Systematic review & Meta-analysis	4	South Asia, East Asia and Pacific, North America, Western Europe	10	AA2. One-time sensitization and education campaigns	JAA12. No vs. partial routine immunisation;	Child

[2] Our search timeframe was up to and including 2021, thus Jain et al. (2022) may appear to be at odds with this. However, Jain et al. (2021) was identified through the grey literature search but was subsequently published in 2022, hence we cite it as Jain et al. (2022) throughout.

For peer review only

Authors & Year	Source	Type of review	Geographic focus		No. primary studies	Types of intervention included	Types of outcomes included	Population level analysed
			No. of regions	Regions				
Lagarde & Palmer, 2009[57]	Journal article	Systematic review	3	South Asia, East Asia and Pacific, Latin America & the Caribbean	3	BF4. Health system financing	JAA01. Full routine immunisation for children	Child
Lagarde, Haines, & Palmer, 2009[58]	Journal article	Systematic review	2	Sub-Saharan Africa, Latin America & the Caribbean	10	AB1. Material/ monetary incentives for caregivers	JAA02. BCG; JAA11. Measles; JAA03. DPT1; JAA05. DPT3; JAA01. Full routine immunisation for children	Child
Linde et al., 2009[59]	Journal article	Systematic review & Meta-analysis	2	Sub-Saharan Africa, Middle East & North Africa	38	AB4. Written or pictorial messages (SMS, stickers, flyers etc.) to caregivers	JAA15. Vaccination coverage (unspecified)	Child
Mekonnen et al., 2019[60]	Journal Article	Systematic review & Meta-analysis	3	Sub-Saharan Africa, Latin America & the Caribbean, North America	10	AB4. Written or pictorial messages (SMS, stickers, flyers etc.) to caregivers	JAA15. Vaccination coverage (unspecified)	Child
Molina et al., 2016[61]	Published report	Systematic review & Meta-analysis	4	Sub-Saharan Africa, South Asia, East Asia and Pacific, Latin America & the Caribbean	15	DA1. Collaborating with whole community; AA2. One-time sensitization and education campaigns; DA1. Collaborating with whole community	JAA15. Vaccination coverage (unspecified)	Child
Odendaal et al., 2018[62]	Journal article	Systematic review	2	East Asia and Pacific, Latin America & the Caribbean	2	BF4. Health system financing	JAA15. Vaccination coverage (unspecified); ICA1. Stockouts	Child; Health facility

For peer review only

Authors & Year	Source	Type of review	Geographic focus		No. primary studies	Types of intervention included	Types of outcomes included	Population level analysed
			No. of regions	Regions				
Oyo-Ita et al., 2016[63]	Journal article	Systematic review & Meta-analysis	4	Sub-Saharan Africa, South Asia, Latin America & the Caribbean, Eastern Europe and Central Asia	14	AA1. Sustained sensitization and education campaigns; AA2. One-time sensitization and education campaigns; AA2. One-time sensitization and education campaigns; AA2. One-time sensitization and education campaigns; AB4. Written or pictorial messages (SMS, stickers, flyers etc.) to caregivers; BA1. Formal health worker training and education; BF1. Health system strategic planning; AB1. Material/ monetary incentives for caregivers; BB5. Outreach to vulnerable populations (hard-to-reach, SES, caste, etc.); BB5. Outreach to vulnerable populations (hard-to-reach, SES, caste, etc.); AB1. Material/ monetary incentives for caregivers; BB7. Home visits; AB1. Material/ monetary incentives for caregivers; BF1. Health system strategic planning	JAA05. DPT3; JAA01. Full routine immunisation for children; JAA11. Measles; JAA12. No vs. partial routine immunisation; JAA13. Vaccination Timeliness; JAA02. BCG; JAA03. DPT1; JAA09. OPV3	Child
Palmer et al., 2020[64]	Journal article	Systematic review & Meta-analysis	6	Sub-Saharan Africa, South Asia, East Asia and Pacific, Latin America & the Caribbean, Eastern Europe and Central Asia, North America	27	AB4. Written or pictorial messages (SMS, stickers, flyers etc.) to caregivers; AB4. Written or pictorial messages (SMS, stickers, flyers etc.) to caregivers; AB3. Automated voice messages to caregivers	JAA15. Vaccination coverage (unspecified); JAA13. Vaccination Timeliness	Child

For peer review only

Authors & Year	Source	Type of review	Geographic focus		No. primary studies	Types of intervention included	Types of outcomes included	Population level analysed
			No. of regions	Regions				
Saeterdal et al., 2014[65]	Journal Article	Systematic review	1	South Asia	2	AA2. One-time sensitization and education campaigns	HAA1. Knowledge about immunisation; JAA15. Vaccination coverage (unspecified); JAA11. Measles; JAA05. DPT3; JAA09. OPV3; HAA2. Attitudes about immunisation; HAB2. Household norms & decision-making	Caregivers; Child
Willey et al., 2013[66]	Published report	Systematic review	4	Sub-Saharan Africa, South Asia, East Asia and Pacific, Latin America & the Caribbean	12	BA1. Formal health worker training and education; BF1. Health system strategic planning	JAA15. Vaccination coverage (unspecified); JAA11. Measles	Child
Witter et al., 2012[67]	Journal article	Systematic review	2	Sub-Saharan Africa, East Asia and Pacific	9	BD5. Pay-for-performance schemes	JAA15. Vaccination coverage (unspecified)	Child
Medium confidence reviews (6 in total)								
Atkinson et al., 2019[68]	Journal article	Systematic review & Meta-analysis	4	Sub-Saharan Africa, Latin America & the Caribbean, Middle East & North Africa, North America	12	AB4. Written or pictorial messages (SMS, stickers, flyers etc.) to caregivers	JAA01. Full routine immunisation for children	Child
Bassani et al., 2013[69]	Journal article	Systematic review & Meta-analysis	3	Sub-Saharan Africa, South Asia, Latin America & the Caribbean	25	AB1. Material/ monetary incentives for caregivers	JAA15. Vaccination coverage (unspecified); JAA01. Full routine immunisation for children	Child
Chutiyami, Wyver, & Amin, 2019[70]	Journal article	Systematic review & Meta-analysis	7	Sub-Saharan Africa, South Asia, East Asia and Pacific, Latin America & the Caribbean, Middle East & North Africa, North America, Western Europe	32	AB4. Written or pictorial messages (SMS, stickers, flyers etc.) to caregivers	JAA15. Vaccination coverage (unspecified); HAA1. Knowledge about immunisation	Child; Caregivers

For peer review only

Authors & Year	Source	Type of review	Geographic focus		No. primary studies	Types of intervention included	Types of outcomes included	Population level analysed
			No. of regions	Regions				
Desai et al., 2020[71]	Journal article	Systematic review	1	South Asia	99	DA2. Collaborating with selected community groups and networks	JAA15. Vaccination coverage (unspecified)	Child
Gilmore & McAuliffe 2013[72]	Journal Article	Systematic review	4	Sub-Saharan Africa, South Asia, East Asia and Pacific, Latin America & the Caribbean	17	AA2. One-time sensitization and education campaigns	JAA01. Full routine immunisation for children; JAA15. Vaccination coverage (unspecified)	Child
Lukusa et al., 2018[73]	Journal Article	Systematic review & Meta-analysis	1	South Asia	6	AA2. One-time sensitization and education campaigns	JAA15. Vaccination coverage (unspecified); JAA05. DPT3	Child

For peer review only

Table 6.2. Low confidence systematic reviews

Authors & Year	Source	Type of review	Geographic focus		No. primary studies	Types of intervention included	Types of outcomes included	Population level analysed
			No. of regions	Regions				
Baequni, 2012[74]	Journal article	Systematic review & Meta-analysis	2	South Asia, East Asia and Pacific	4	AB4. Written or pictorial messages (SMS, stickers, flyers etc.) to caregivers	JAA15. Vaccination coverage (unspecified)	Child
Batt, Fox-Rushby, & Castillo-Riquelme, 2004[75]	Journal Article	Systematic review	5	Sub-Saharan Africa, South Asia, East Asia and Pacific, Latin America & the Caribbean, Middle East & North Africa	34	F. Multicomponent	JAA01. Full routine immunisation for children	Child
Bright et al., 2017[76]	Journal article	Systematic review	5	Sub-Saharan Africa, South Asia, East Asia and Pacific, Latin America & the Caribbean, Middle East & North Africa	57	BB5. Outreach to vulnerable populations (hard-to-reach, SES, caste, etc.); AB1. Material/ monetary incentives for caregivers; BB7. Home visits; BF1. Health system strategic planning; BD5. Pay-for-performance schemes; AA2. One-time sensitization and education campaigns; AB4. Written or pictorial messages (SMS, stickers, flyers etc.) to caregivers; DA2. Collaborating with selected community groups and networks; BD4. Written or pictorial messages (SMS, stickers, flyers etc.) to health workers; AB1. Material/ monetary incentives for caregivers; F. Multicomponent	JAA15. Vaccination coverage (unspecified)	Child

For peer review only

Authors & Year	Source	Type of review	Geographic focus		No. primary studies	Types of intervention included	Types of outcomes included	Population level analysed
			No. of regions	Regions				
Crocker-Buque et al., 2017[77]	Journal article	Systematic review	4	Sub-Saharan Africa, South Asia, East Asia and Pacific, Latin America & the Caribbean	20	F. Multicomponent; BB5. Outreach to vulnerable populations (hard-to-reach, SES, caste, etc.); AB4. Written or pictorial messages (SMS, stickers, flyers etc.) to caregivers; AA2. One-time sensitization and education campaigns; AB1. Material/ monetary incentives for caregivers	JAA15. Vaccination coverage (unspecified)	Child
Das, Gopalan, & Chandramohan, 2016[78]	Journal article	Systematic review	3	Sub-Saharan Africa, East Asia and Pacific, Middle East & North Africa	8	BD5. Pay-for-performance schemes	ICA1. Stockouts	Health facility
de Cock et al., 2020[79]	Journal Article	Systematic review	5	Sub-Saharan Africa, South Asia, East Asia and Pacific, North America, Western Europe	28	BG1. New HMIS/Dashboard systems (incl. improved data collection)	JAA15. Vaccination coverage (unspecified)	Child
de Souza Cruz et al., 2017[80]	Journal Article	Systematic review	3	Sub-Saharan Africa, South Asia, Latin America & the Caribbean	17	AB1. Material/ monetary incentives for caregivers	JAA15. Vaccination coverage (unspecified); JAA01. Full routine immunisation for children	Child
Deardorff et al., 2018[81]	Journal article	Systematic review & Meta-analysis	5	Sub-Saharan Africa, South Asia, East Asia and Pacific, Latin America & the Caribbean, Middle East & North Africa	28	AA2. One-time sensitization and education campaigns; AA3. Public information campaigns; CA1. Faith-based outreach/outreach using local leaders; BC1. National/sub-national immunisation days	JAA15. Vaccination coverage (unspecified); JAA11. Measles	Child

For peer review only

Authors & Year	Source	Type of review	Geographic focus		No. primary studies	Types of intervention included	Types of outcomes included	Population level analysed
			No. of regions	Regions				
Freeman et al., 2017[82]	Journal article	Systematic review	8	Sub-Saharan Africa, South Asia, East Asia and Pacific, Latin America & the Caribbean, Middle East & North Africa, Eastern Europe and Central Asia, North America, Western Europe	489	BB2. Community health worker involvement in planning & monitoring; BB7. Home visits; CA1. Faith-based outreach/outreach using local leaders; AA3. Public information campaigns; AA2. One-time sensitization and education campaigns; AB1. Material/ monetary incentives for caregivers; AA2. One-time sensitization and education campaigns; BC1. National/sub-national immunisation days; BB7. Home visits	JAA15. Vaccination coverage (unspecified); JAA01. Full routine immunisation for children	Child
Giedion, Andrés Alfonso, & Díaz, 2013[83]	Published report	Systematic review	6	Sub-Saharan Africa, South Asia, East Asia and Pacific, Latin America & the Caribbean, Middle East & North Africa, Eastern Europe and Central Asia	105	AB5. Changes to health system user fees	JAA01. Full routine immunisation for children	Child
Harvey et al., 2015[84]	Journal article	Systematic review & Meta-analysis	5	Sub-Saharan Africa, South Asia, East Asia and Pacific, North America, Western Europe	28	AA2. One-time sensitization and education campaigns; AB4. Written or pictorial messages (SMS, stickers, flyers etc.) to caregivers	JAA15. Vaccination coverage (unspecified)	Child
Iwu et al., 2019[85]	Journal Article	Systematic review	3	Sub-Saharan Africa, South Asia, North America	4	BF1. Health system strategic planning; BG1. New HMIS/Dashboard systems (incl. improved data collection); BG2. Capacity building (e.g., training) for existing systems	ICA1. Stockouts; IBA1. Immunisation data collection (quality, completeness); HAD2. Actual cost of vaccinating	Health facility; Child

Authors & Year	Source	Type of review	Geographic focus		No. primary studies	Types of intervention included	Types of outcomes included	Population level analysed
			No. of regions	Regions				
Jarrett et al., 2015[86]	Journal Article	Systematic review	4	Sub-Saharan Africa, South Asia, Eastern Europe and Central Asia, Western Europe	13	CA1. Faith-based outreach/outreach using local leaders; F. Multicomponent; AA2. One-time sensitization and education campaigns ; AA2. One-time sensitization and education campaigns; BA1. Formal health worker training and education; AB1. Material/ monetary incentives for caregivers; AB4. Written or pictorial messages (SMS, stickers, flyers etc.) to caregivers	JAA09. OPV3; JAA15. Vaccination coverage (unspecified); JAA05. DPT3	Child
Karageorgos et al., 2019[87]	Journal Article	Systematic review	5	Sub-Saharan Africa, South Asia, East Asia and Pacific, Latin America & the Caribbean, Middle East & North Africa	98	AB4. Written or pictorial messages (SMS, stickers, flyers etc.) to caregivers	JAA15. Vaccination coverage (unspecified)	Child
Kim, Patel, & Hinman, 2017[88]	Journal Article	Systematic review	4	Sub-Saharan Africa, South Asia, East Asia and Pacific, Latin America & the Caribbean	26	AB4. Written or pictorial messages (SMS, stickers, flyers etc.) to caregivers; BG1. New HMIS/Dashboard systems (incl. improved data collection); AA2. One-time sensitization and education campaigns	JAA15. Vaccination coverage (unspecified); JAA13. Vacc. Timeliness; IBA1. Immunisation data collection (quality, completeness); HAC1. Readiness to vaccinate	Child; Healthcare workers; Caregivers
Loevinsohn & Harding, 2004[89]	Discussion paper	Systematic review	4	Sub-Saharan Africa, South Asia, East Asia and Pacific, Latin America & the Caribbean	10	BF4. Health system financing	JAA15. Vaccination coverage (unspecified);	Child;

For peer review only

Authors & Year	Source	Type of review	Geographic focus		No. primary studies	Types of intervention included	Types of outcomes included	Population level analysed
			No. of regions	Regions				
Munk et al., 2019[37]	Journal Article	Systematic review	4	Sub-Saharan Africa, South Asia, East Asia and Pacific, Latin America & the Caribbean	14	F. Multicomponent	JAA15. Vaccination coverage (unspecified); HAD2. Actual cost of vaccinating	Child
Mureed et al., 2015[90]	Journal Article	Systematic review & Meta-analysis	3	Sub-Saharan Africa, South Asia, Middle East & North Africa	10	F. Multicomponent	JAA15. Vaccination coverage (unspecified); JAA05. DPT3; JAA09. OPV3; JAA11. Measles; JAA01. Full routine immunisation for children	Child
Naugle & Hornik, 2014[91]	Journal Article	Systematic review	6	Sub-Saharan Africa, South Asia, East Asia and Pacific, Latin America & the Caribbean, Middle East & North Africa, Eastern Europe and Central Asia	106	AA3. Public information campaigns	JAA15. Vaccination coverage (unspecified)	Child
Nelson et al., 2016[92]	Journal Article	Systematic review	6	Sub-Saharan Africa, South Asia, East Asia and Pacific, Latin America & the Caribbean, Middle East & North Africa, Eastern Europe and Central Asia	15	F. Multicomponent; BF1. Health system strategic planning	JAA01. Full routine immunisation for children; JAA05. DPT3	Child
Nutman, McKee, & Khoshnood, 2013[93]	Journal Article	Systematic review	1	Sub-Saharan Africa	21	BF1. Health system strategic planning	JAA15. Vaccination coverage (unspecified)	Child
Oliver-Williams et al., 2017[94]	Journal article	Systematic review	3	Sub-Saharan Africa, South Asia, East Asia and Pacific	21	AB3. Automated voice messages to caregivers; AB4. Written or pictorial messages (SMS, stickers, flyers etc.) to caregivers	JAA15. Vaccination coverage (unspecified)	Child

For peer review only

Authors & Year	Source	Type of review	Geographic focus		No. primary studies	Types of intervention included	Types of outcomes included	Population level analysed
			No. of regions	Regions				
Omoniyi & Williams, 2020[38]	Journal article	Systematic review	5	Sub-Saharan Africa, South Asia, East Asia and Pacific, Latin America & the Caribbean, Middle East & North Africa	27	AA2. One-time sensitization and education campaigns; CA1. Faith-based outreach/outreach using local leaders; AB4. Written or pictorial messages (SMS, stickers, flyers etc.) to caregivers; AB1. Material/ monetary incentives for caregivers; BB7. Home visits; CA1. Faith-based outreach/outreach using local leaders; BA1. Formal health worker training and education; BF1. Health system strategic planning; BF1. Health system strategic planning; F. Multicomponent	JAA15. Vaccination coverage (unspecified)	Child
Onwuchekwa, Verdonck, & Marchal, 2021[43]	Journal Article	Systematic review	1	Sub-Saharan Africa	9	AB1. Material/ monetary incentives for caregivers	JAA15. Vaccination coverage (unspecified)	Child
Owusu-Addo & Cross, 2014[95]	Journal article	Systematic review	2	Sub-Saharan Africa, Latin America & the Caribbean	16	AB1. Material/ monetary incentives for caregivers	JAA15. Vaccination coverage (unspecified)	Child
Ozawa, Yemeke, & Thompson, 2018[96]	Journal article	Systematic review	4	Sub-Saharan Africa, South Asia, East Asia and Pacific, Latin America & the Caribbean	42	F. Multicomponent	0	Child

For peer review only

Authors & Year	Source	Type of review	Geographic focus		No. primary studies	Types of intervention included	Types of outcomes included	Population level analysed
			No. of regions	Regions				
Pal, Goodyear-Smith, & Exeter, 2016[97]	Journal article	Systematic review	2	South Asia, East Asia and Pacific	14	AA2. One-time sensitization and education campaigns; AB4. Written or pictorial messages (SMS, stickers, flyers etc.) to caregivers; BA1. Formal health worker training and education; AA2. One-time sensitization and education campaigns; DA1. Collaborating with whole community; AB1. Material/ monetary incentives for caregivers	JAA05. DPT3; JAA03. DPT1	Child
Parrado-Jiménez, 2013[98]	Unpublished research	Systematic review	5	Sub-Saharan Africa, South Asia, East Asia and Pacific, Latin America & the Caribbean, Middle East & North Africa	21	BD5. Pay-for-performance schemes	JAA01. Full routine immunisation for children	Child
Pegurri, Fox-Rushby, & Damian, 2005[99]	Journal article	Systematic review	3	Sub-Saharan Africa, South Asia, Latin America & the Caribbean	60	F. Multicomponent	JAA15. Vaccination coverage (unspecified)	Child
Pinzón-Flórez et al., 2015[100]	Journal article	Systematic review	4	Sub-Saharan Africa, South Asia, East Asia and Pacific, Latin America & the Caribbean	19	AA2. One-time sensitization and education campaigns	JAA01. Full routine immunisation for children; HAD5. Vaccination health card availability/ retention	Child
Rahman et al., 2013[101]	Journal Article	Systematic review & Meta-analysis	4	Sub-Saharan Africa, South Asia, East Asia and Pacific, Latin America & the Caribbean	13	BB7. Home visits	JAA01. Full routine immunisation for children	Child

For peer review only

Authors & Year	Source	Type of review	Geographic focus		No. primary studies	Types of intervention included	Types of outcomes included	Population level analysed
			No. of regions	Regions				
Ranganathan & Lagarde, 2012[102]	Journal Article	Systematic review	3	Sub-Saharan Africa, South Asia, Latin America & the Caribbean	18	AB1. Material/ monetary incentives for caregivers	JAA15. Vaccination coverage (unspecified)	Child
Ryman, Dietz, & Cairns, 2008[103]	Journal Article	Systematic review	4	Sub-Saharan Africa, South Asia, East Asia and Pacific, Latin America & the Caribbean	25	AA2. One-time sensitization and education campaigns; BB5. Outreach to vulnerable populations (hard-to-reach, SES, caste, etc.); BB7. Home visits; AA1. Sustained sensitization and education campaigns; AA2. One-time sensitization and education campaigns; AA3. Public information campaigns; AA1. Sustained sensitization and education campaigns; BF1. Health system strategic planning; AB4. Written or pictorial messages (SMS, stickers, flyers etc.) to caregivers; AA2. One-time sensitization and education campaigns; BF4. Health system financing; BA1. Formal health worker training and education; BD1. Material/monetary incentives for health workers	JAA15. Vaccination coverage (unspecified); JAA13. Vacc. Timeliness	Child

For peer review only

Authors & Year	Source	Type of review	Geographic focus		No. primary studies	Types of intervention included	Types of outcomes included	Population level analysed
			No. of regions	Regions				
Shea, Andersson, & Henry, 2009[104]	Journal Article	Systematic review	5	Sub-Saharan Africa, South Asia, East Asia and Pacific, Latin America & the Caribbean, Middle East & North Africa	8	AA3. Public information campaigns; F. Multicomponent; BB5. Outreach to vulnerable populations (hard-to-reach, SES, caste, etc.); AA2. One-time sensitization and education campaigns; AA2. One-time sensitization and education campaigns; BD1. Material/monetary incentives for health workers; BD1. Material/monetary incentives for health workers; BB5. Outreach to vulnerable populations (hard-to-reach, SES, caste, etc.); BB7. Home visits; AA. Information & education	JAA01. Full routine immunisation for children; HAA1. Knowledge about immunisation; JAA15. Vaccination coverage (unspecified); JAA11. Measles; HAD2. Actual cost of vaccinating	Child; Caregivers
Smith et al., 2018[105]	Journal Article	Systematic review	1	Sub-Saharan Africa	28	BF1. Health system strategic planning	JAA15. Vaccination coverage (unspecified)	Child
Watterson, Walsh, & Madeka, 2015[106]	Journal Article	Systematic review	2	Sub-Saharan Africa, East Asia and Pacific	10	AB4. Written or pictorial messages (SMS, stickers, flyers etc.) to caregivers; BG1. New HMIS/Dashboard systems (incl. improved data collection); AB1. Material/ monetary incentives for caregivers; AB4. Written or pictorial messages (SMS, stickers, flyers etc.) to caregivers; AB3. Automated voice messages to caregivers; AA2. One-time sensitization and education campaigns; BG1. New HMIS/Dashboard systems (incl. improved data collection)	JAA13. Vacc. Timeliness; HAC1. Readiness to vaccinate; JAA11. Measles	Child; Caregivers

For peer review only

Authors & Year	Source	Type of review	Geographic focus		No. primary studies	Types of intervention included	Types of outcomes included	Population level analysed
			No. of regions	Regions				
Yuan et al., 2014[107]	Journal Article	Systematic review	4	Sub-Saharan Africa, South Asia, East Asia and Pacific, Latin America & the Caribbean	22	BB5. Outreach to vulnerable populations (hard-to-reach, SES, caste, etc.); AA3. Public information campaigns; BF1. Health system strategic planning	JAA11. Measles	Child
Yunusa et al., 2021[108]	Journal article in progress	Systematic review & Meta-analysis	3	Sub-Saharan Africa, South Asia, Latin America & the Caribbean	11	AB4. Written or pictorial messages (SMS, stickers, flyers etc.) to caregivers; AB3. Automated voice messages to caregivers; AB3. Automated voice messages to caregivers; AB4. Written or pictorial messages (SMS, stickers, flyers etc.) to caregivers	JAA01. Full routine immunisation for children; JAA15. Vaccination coverage (unspecified); JAA13. Vacc. Timeliness	Child

For peer review only

Appendix 7: Risk of bias of included systematic reviews

Sr. No.	Included systematic review	Confidence rating in RoR	Confidence rating in EGM	Type of synthesis	Reasons, if confidence rating differs between RoR and EGM
**High confidence studies (18 in total)**					
1	Diaconu et al., 2021[52]	High	NA[#]	Range of ES + DC	
2	Eze et al., 2021[44]	High	NA	MR + MA + VC direction of effect	
3	Gera et al., 2016[53]	High	High	MA + NS	
4	Glenton et al., 2011[54]	High	NA	MA + NS	
5	Jain et al., 2022[41, 42]	High	NA	MR + MA + TS	
6	Johri et al., 2015[55]	High	Medium	MR + MA	Upgraded to high confidence: EGM coders marked section A as low confidence because the search was not comprehensive. RoR coders marked section A as high confidence as search included studies from other published reviews dating back to 1996. However, we acknowledge some limitations of the search, but these were not sufficient for a low confidence rating.
7	Kaufman et al., 2018[56]	High	High	MA	
8	Lagarde et al., 2009[58]	High	High	NS	
9	Lagarde & Palmer, 2009[57] (update cited as Odendaal et al., 2018[62])	High	High	DC + NS	
10	Linde et al., 2009[59]	High	High	MA + NS	
11	Mekonnen et al., 2019[60]	High	Medium	MR + MA	Upgraded to high confidence: No obvious difference between grading of RoR and EGM coders, however, mitigating factors (e.g., Kappa statistics and Grade assessment) which influenced the overall assessment to high confidence. We acknowledge the search was restricted to English only and unit of analysis error was not reported but this is not sufficient to justify a medium confidence rating.
12	Molina et al., 2016[61]	High	High	MA + DC + NS	

Sr. No.	Included systematic review	Confidence rating in RoR	Confidence rating in EGM	Type of synthesis	Reasons, if confidence rating differs between RoR and EGM
13	Odendaal et al., 2018[62]	High	High	DC + NS	
14	Oyo-Ita et al., 2016[63]	High	High	MA + DC	
15	Palmer et al., 2020[64]	High	NA	MA + DC + NS	
16	Saeterdal et al., 2014[65]	High	High~	DC + NS	
17	Willey et al., 2012[66]	High	High	NS + NS	
18	Witter et al., 2012[67] (update cited as Diaconu et al., 2021[52])	High	High	DC + NS	
**Medium confidence studies (6 in total)**					
19	Atkinson et al., 2019[68]	Medium	Medium	MA	
20	Bassani et al., 2013[69]	Medium	Medium	MA	
21	Chutiyami et al., 2019[70]	Medium	Low	MA + Range of ES + VC statistical significance + VC direction of effect + DC + NS	Upgraded to medium confidence: EGM coders marked section A as low confidence as search was partial, search period not documented and no sufficient criteria to assess study quality. RoR coders marked section A as medium because included studies dated back to 1984 and sufficient criteria were used to assess quality while acknowledging some search limitations. EGM coders marked section B as low. RoR coders upgraded this to medium because 20% of studies were randomly extracted by two independent researchers. Also, section B3 was upgraded as heterogeneity was acknowledged and statistical tests to demonstrate heterogeneity were performed. Unit of analysis error was not documented.
22	Desai et al., 2020[71]	Medium	NA	NS + TS	
23	Gilmore & McAuliffe, 2013[72]	Medium	Medium	Range of ES + NS	
24	Lukusa et al., 2018[73]	Medium	Medium	MA	

Sr. No.	Included systematic review	Confidence rating in RoR	Confidence rating in EGM	Type of synthesis	Reasons, if confidence rating differs between RoR and EGM
**Low confidence studies (38 in total):**					
25	Baequni, 2012[74]	Low	Low	MA	
26	Batt et al., 2004[75]	Low	Low	DC	
27	Bright et al., 2017[76]	Low	Low	VC statistical significance + NS	
28	Crocker-Buque et al., 2017[77]	Low	Low	NS + TS	
29	Das et al., 2016[78]	Low	Low	DC + NS	
30	de Cock et al., 2020[79]	Low	NA	VC direction of effect + NS	
31	de Souza Cruz et al., 2017[80]	Low	Low	NS	
32	Deardorff et al., 2018[81]	Low	Low	MA + DC + NS	
33	Freeman et al., 2017[82]	Low	Low	NS	
34	Giedion et al., 2013[83]	Low	Low	NS	
35	Harvey et al., 2015[84]	Low	Low	MA + Range of ES	
36	Iwu et al., 2019[85]	Low	Low	NS	
37	Jarrett et al., 2015[86]	Low	Low	DC + NS	
38	Karageorgos et al., 2018[87]	Low	Low	NS	
39	Kim et al., 2017[88]	Low	Low	NS	
40	Loevinsohn & Harding, 2004[89]	Low	Low	Range of ES + DC + NS	
41	Munk et al., 2019[37]	Low	Low	Range of ES + DC	
42	Mureed et al., 2015[90]	Low	Low	MA + VC direction of effect	
43	Naugle & Hornik, 2014[91]	Low	Low	VC direction of effect + DC + NS	
44	Nelson et al., 2016[92]	Low	Low	Range of ES + NS	
45	Nutman et al., 2013[93]	Low	Low	Range of ES + VC statistical significance + DC + NS	
46	Oliver-Williams et al., 2017[94]	Low	Low	NS	
47	Omoniyi & Williams, 2020[38]	Low	Low	TS	
48	Onwuchekwa et al., 2021[43]	Low	NA	VC direction of effect + NS + TS	
49	Owusu-Addo & Cross, 2014[95]	Low	Low	NS	

Sr. No.	Included systematic review	Confidence rating in RoR	Confidence rating in EGM	Type of synthesis	Reasons, if confidence rating differs between RoR and EGM
50	Ozawa et al., 2018[96]	Low	Low	DC	
51	Pal et al., 2016[97]	Low	Low	NS	
52	Parrado-Jiménez, 2013[98]	Low	Low	VC statistical significance & direction of effect	
53	Pegurri et al., 2005[99]	Low	Low	DC	
54	Pinzón-Flórez et al., 2015[100]	Low	NA	NS	
55	Rahman et al., 2013[101]	Low	Low	MA + NS	
56	Ranganathan & Lagarde, 2012[102]	Low	Low	NS	
57	Ryman et al., 2008[103]	Low	Low	NS	
58	Shea et al., 2009[104]	Low	Low	NS	
59	Smith et al., 2018[105]	Low	Low	NS	
60	Watterson et al., 2015[106]	Low	Low	NS	
61	Yuan et al. 2014[107]	Low	Medium	NS	Downgraded to low confidence: Authors did not extract data independently. Additionally had further limitations: A summary of the assessment of each included study for each quality assessment criterion was not reported, unit of analysis error was not reported in spite of including cRCTs.
62	Yunusa et al., 2021[108]	Low	NA	MA + NS	

Notes: ~Saeterdal et al., 2014[65] was mistakenly marked as medium confidence in the earlier draft of the EGM manuscript and online map, but this will be rectified before publication.

#NA in column, confidence rating in EGM: these are the systematic reviews identified through our updated search and were not included in the EGM.

Abbreviations: cRCTs: Cluster randomised controlled trials; DC: Descriptive; EGM: Evidence Gap Maps; ES: Effect sizes; MA: Meta-analysis; MR: Meta regression; NS: Narrative synthesis; RoR: Review of reviews; TS: Thematic synthesis; VC: Vote counting.

Appendix 8: RVE analysis – 1st tier synthesis

Figure A8.1: RVE of all interventions under Type A, 1st tier, mean baseline coverage values

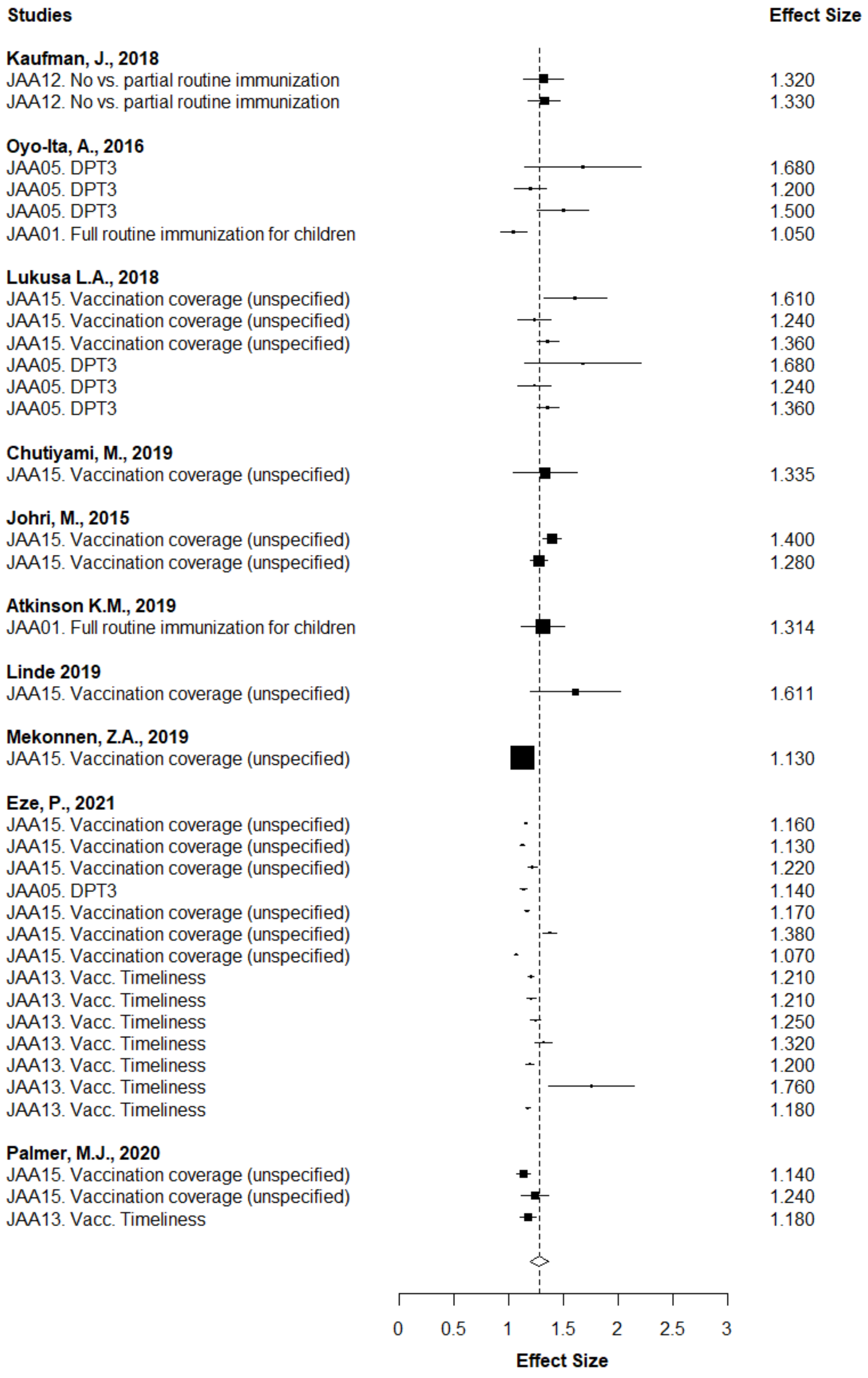

Notes: Drawing on k = 10 and 35 effect sizes.

Additional RVE analysis – 1st tier synthesis

Figure A8.2: RVE of all interventions under Type A, 1st tier, one standard deviation above the mean (baseline coverage rate of 70%)

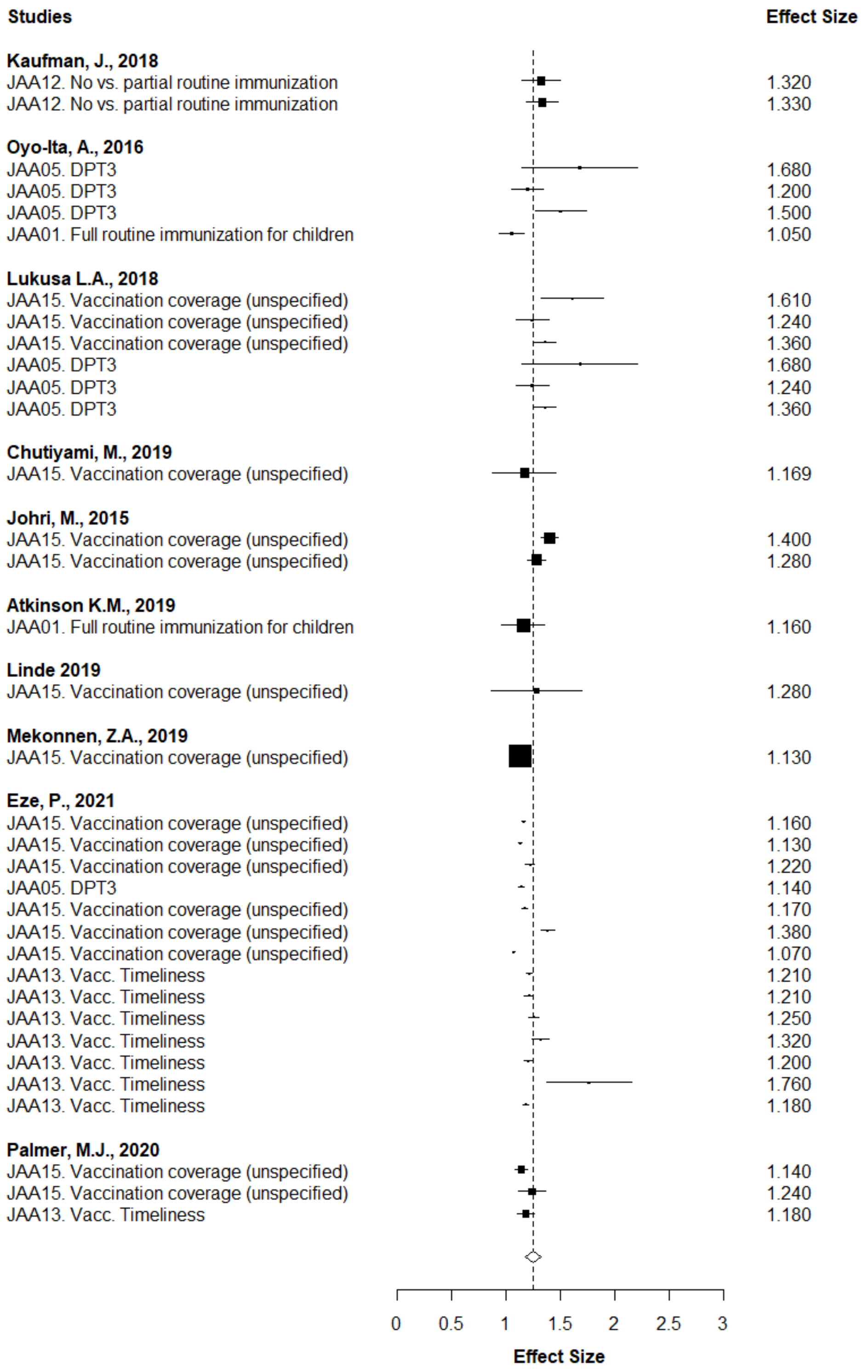

Notes: Drawing on k = 10 and 35 effect sizes.

Figure A8.3: RVE of all interventions under Type A, 1st tier, one standard deviation below the mean (baseline coverage rate of 26%)

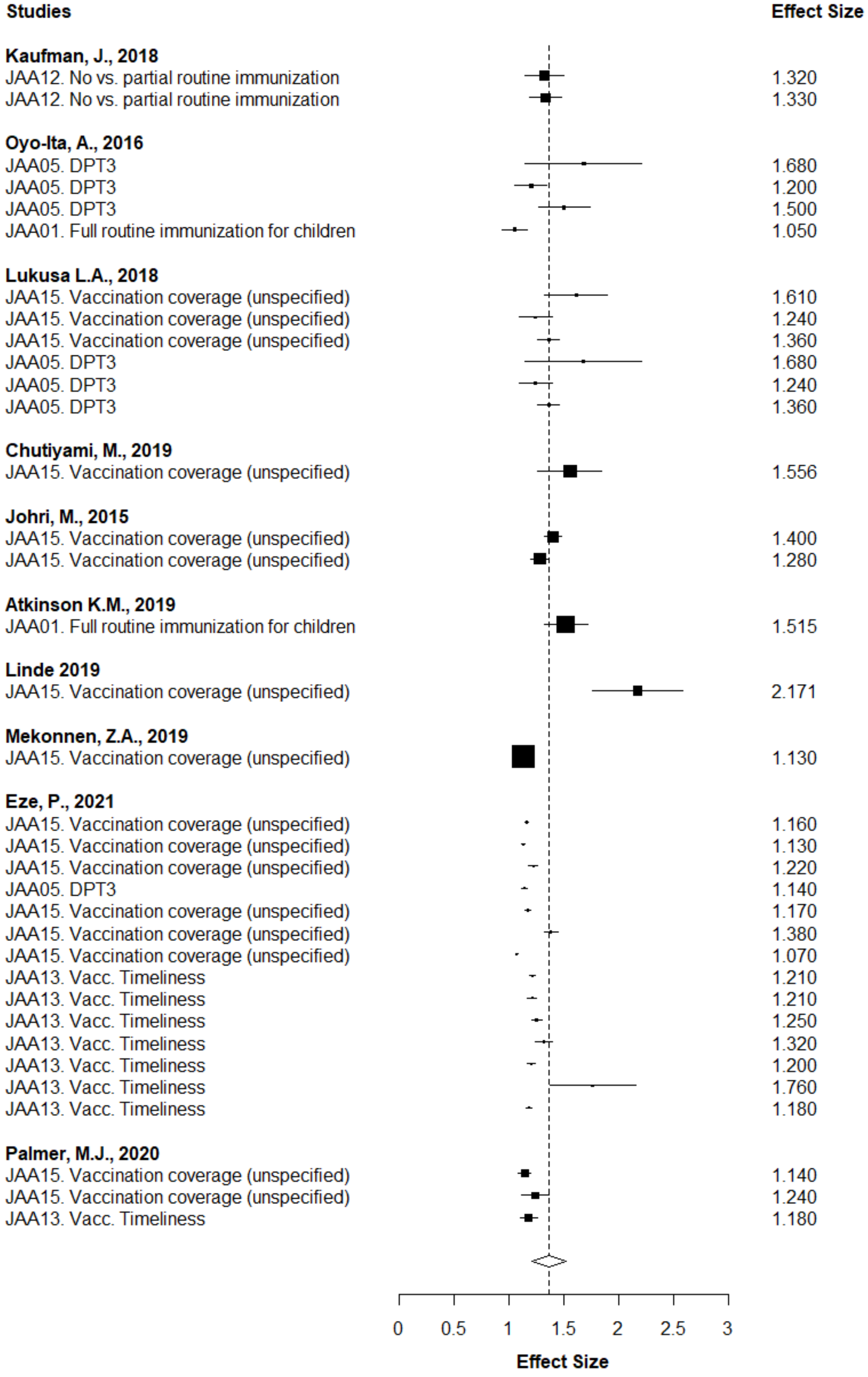

Notes: Drawing on k = 10 and 35 effect sizes.

For peer review only - http://bmjopen.bmj.com/site/about/guidelines.xhtml

Appendix 9: RVE analysis – 2nd tier synthesis

Figure 9.1: RVE of all interventions under Type AB, 2nd tier, mean baseline coverage values

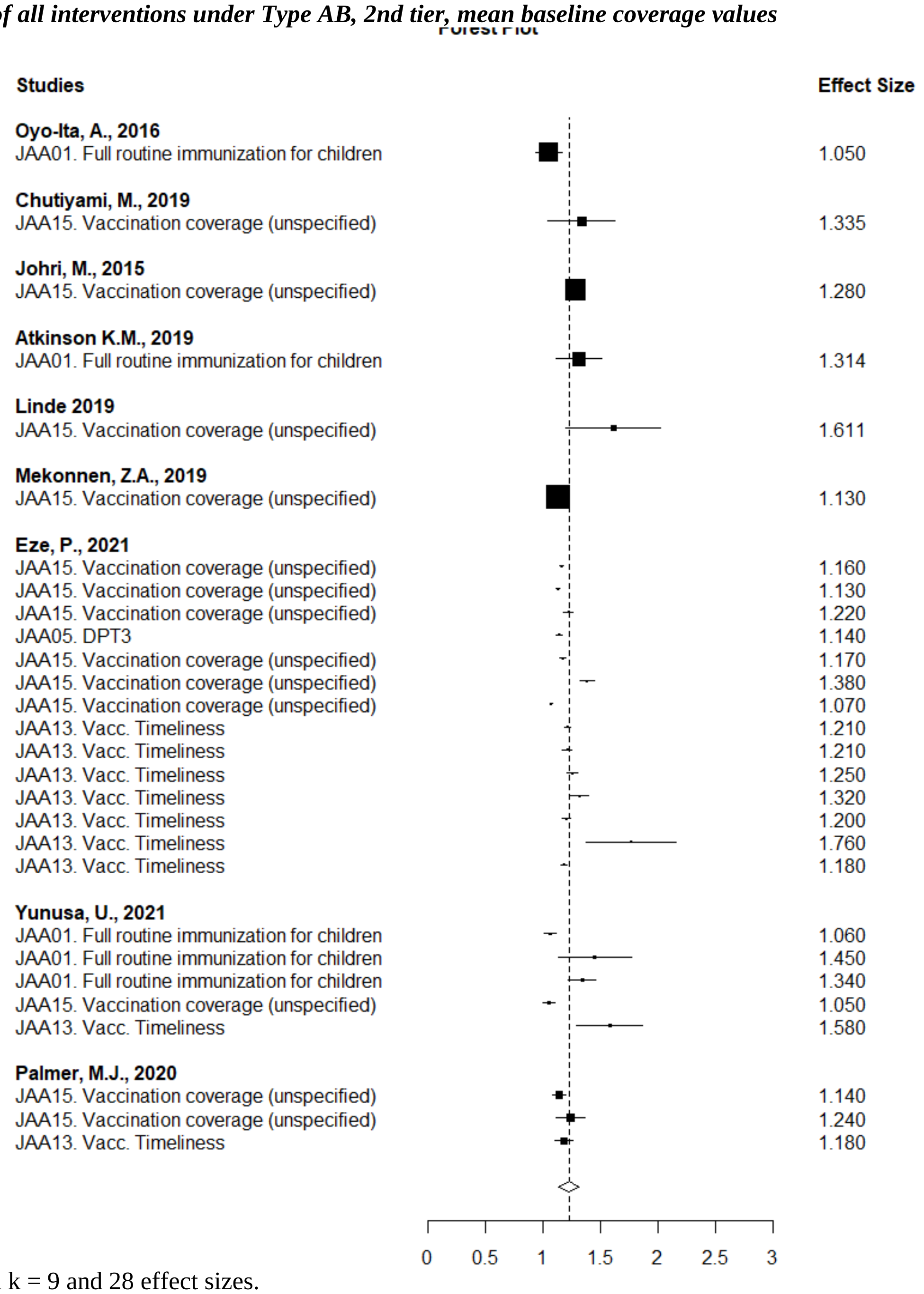

Notes: Drawing on k = 9 and 28 effect sizes.

Additional RVE analysis – 2nd tier synthesis

Figure A9.2: RVE of all interventions under Type AB, 2nd tier, one standard deviation above the mean (baseline coverage rate of 70%)

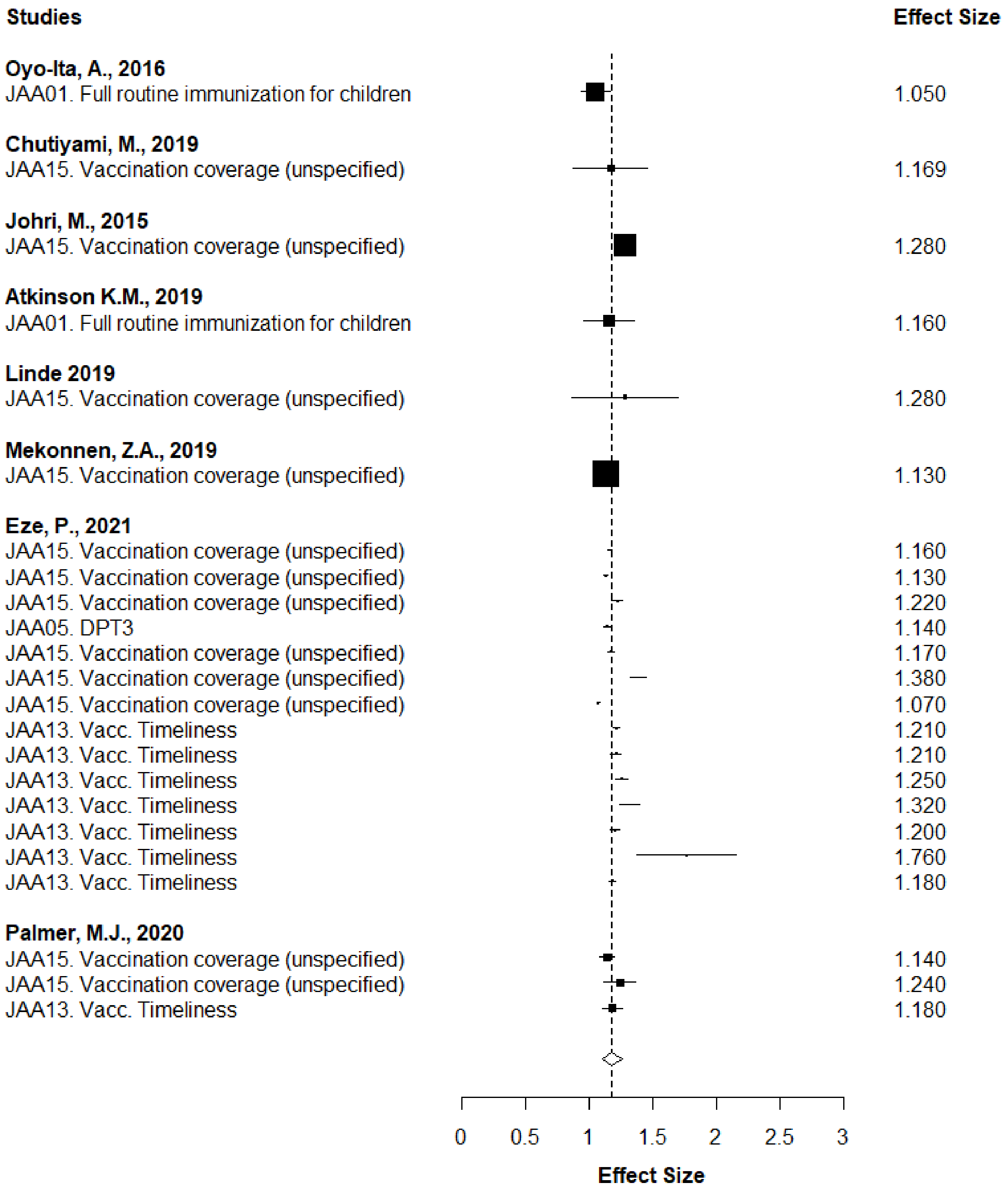

Notes: Drawing on k = 8 and 23 effect sizes.

Figure A9.3: RVE of all interventions under Type AB, 2nd tier, one standard deviation below the mean (baseline coverage rate of 26%)

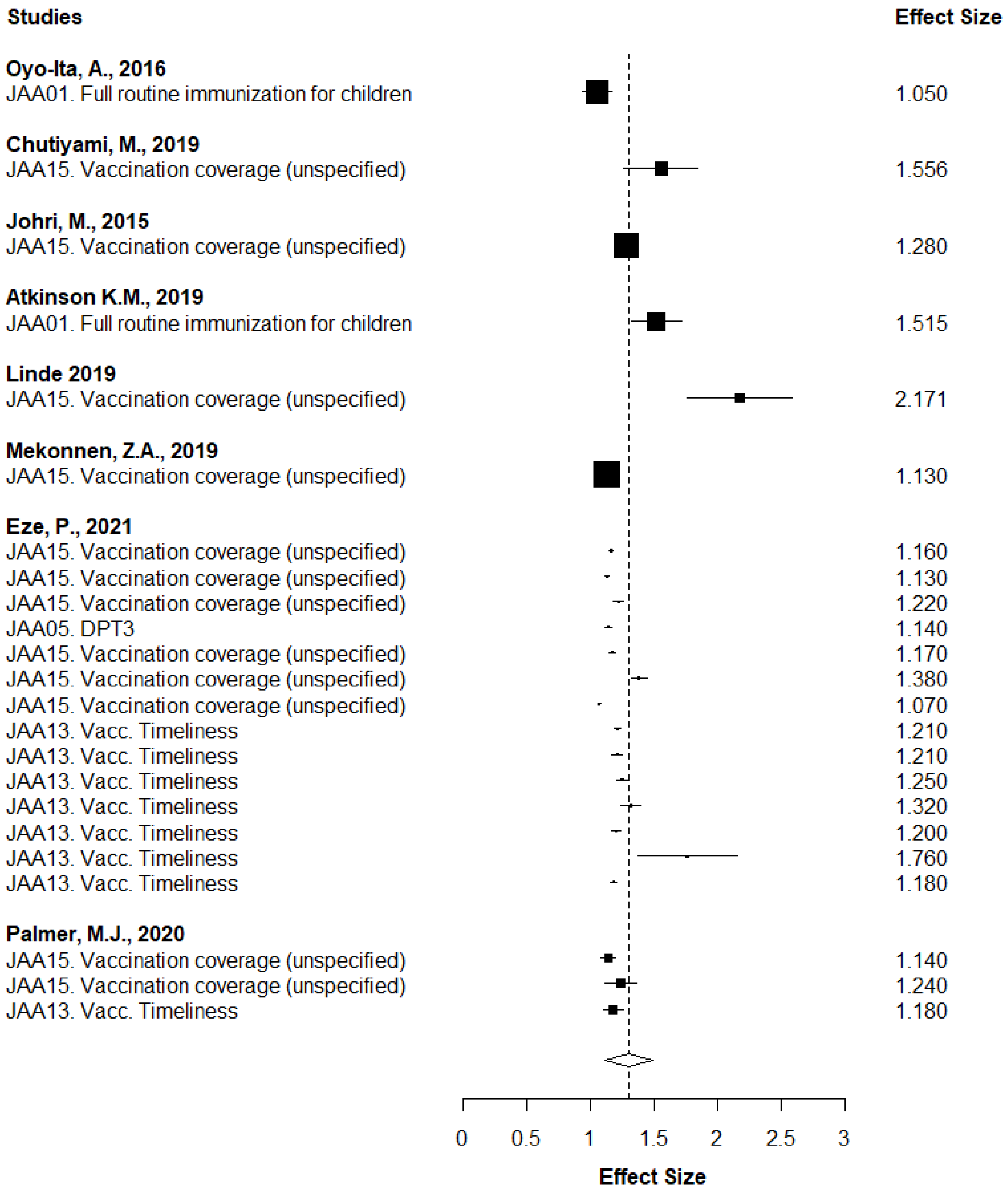

Notes: Drawing on k = 8 and 23 effect sizes.

Appendix 10: Health-systems oriented interventions

This section synthesises the high and medium confidence evidence in relation to health-systems oriented interventions (B). We have insufficient quantitative data for health-systems oriented interventions (B), hence, this section adopts a narrative approach. We begin with a summary of what we know about health-systems oriented interventions (B). The two tables below are based on 9 reviews that discuss health-systems oriented intervention categories (B), 2 of these reviews[54, 63] are captured in Table 11 above already as they are discussing complementary interventions spanning the broader categories caregiver (A) and health-systems oriented interventions (B).

Table 12 lists six reviews presenting a mixed picture in terms of intervention categories, outcomes and findings. 2 reviews each examine home visits (BB7), pay-for-performance schemes (BD5) and health system financing (BF4) in more depth. As with caregiver-oriented intervention categories (A), the evidence is based on very small study numbers. E.g., for home visits (BB7), two reviews[54, 63], find favourable effects on OPV3, measles and full routine immunisation for children. Diaconu et al.[52] and Witter et al.[67] examine pay-for-performance schemes (BD5) finding mixed results. E.g., Diaconu et al. (2021)[52] drawing on 2 to 9 studies (all low certainty) across a wide range of outcomes suggest that pay-for-performance schemes (BD5) are effective in terms of improving full routine immunisation for children, measles coverage and community health worker motivation with the remaining results being inconclusive. Witter et al. (2012)[67] only investigate the link between pay-for-performance schemes (BD5) and vaccination coverage more broadly finding inconclusive results drawing on 2 low certainty studies. Caution is required regarding over-interpreting the results of pay-for-performance schemes. A review of payment-by-results schemes for health interventions in L&MICs[109] argues that these schemes typically focus on short-term output measures such as vaccination coverage that obscure the bigger picture, e.g., the need to reform the wider health system to ensure better delivery of immunisation interventions, and not necessarily provide a long-term measure of the effectiveness of a programme. Lagarde & Palmer (2009)[57] and Odendaal et al. (2018)[62] examine health system financing (BF4) finding inconclusive results, again working with a small number of studies and across heterogenous outcomes. The high and medium confidence evidence for the remaining health-systems oriented interventions (B) is difficult to unpack systematically as we only have 1 review each for formal health worker training and education (BA1), outreach to vulnerable populations (hard-to-reach, SES, caste, etc.) (BB5) and health system strategic planning (BF1). In summary, we have no evidence on the majority of the 3rd tier health-systems oriented interventions (B) except for formal health worker training and education (BA1), promoting outreach to vaccine-hesitant groups (BB4) (in combination with national/sub-national immunisation days (BC1), see Table 13), outreach to vulnerable populations (hard-to-reach, SES, caste, etc.) (BB5), home visits (BB7), pay-for-performance schemes (BD5), health system strategic planning (BF1) and health system financing (BF4).

Table A10.1: B. Health-systems oriented interventions (all high confidence systematic reviews) – one intervention only

Systematic review	Intervention, 3rd tier level	Outcome	Finding
Oyo-Ita et al., 2016[63]	BA1. Formal health worker training and education	JAA05. DPT3; JAA09. OPV3	• DPT3: inconclusive effect, low certainty (k = 1 study) [p.16]<br>• OPV3: inconclusive effect, low certainty (k = 1 study) [p.17]
Oyo-Ita et al., 2016[63]	BB5. Outreach to vulnerable populations (hard-to-reach, SES, caste, etc.)	JAA01. Full routine immunization for children	• Full routine immunization for children: favourable effect, low certainty (k = 1 study) [p.17]
Oyo-Ita et al., 2016[63]	BB7. Home visits	JAA09. OPV3; JAA11. Measles	• OPV3: favourable effect, low certainty (k = 1 study) [p.18]<br>• Measles: favourable effect, low certainty (k = 1 study) [p.18]
Glenton et al., 2011[54]	BB7. Home visits	JAA01. Full routine immunization for children	• Full routine immunization for children: favourable effect (k = 1 study) [p.1049]

For peer review only

Diaconu et al., 2021[52]	BD5. Pay-for-performance schemes	JAA15. Vaccination coverage (unspecified); JAA01. Full routine immunisation for children; JAA02. BCG; JAA05. DPT3; JAA11. Measles; JAA09. OPV3; IAA1. Community HW motivation, capacity & performance; ICA1. Stockouts; HAD4. Experience and satisfaction with health services	• Vaccination coverage (unspecified): Inconsistent effects of small magnitude, low certainty evidence (k = 5 studies) [p. 177, 210]. Effective among poorest group for the targeted outcome (k = 2 studies), low certainty evidence, however, not for untargeted outcome, wherein it was very low certainty evidence (k = 1 study) [p.193]<br>• Full immunisation coverage: effective, low certainty evidence (k = 8 studies) [p. 178], however, for targeted measures for provider performance there was inconclusive effects, low certainty evidence (k = 3 studies) [p. 210]<br>• DPT3 coverage: Little or no effects, low certainty evidence for DPT 3 (k = 6 studies) or Pentavalent (k = 3 studies) [p. 178, 210]<br>• Measles coverage: Desirable effects, low certainty evidence (k = 6 studies) [p. 178]<br>• BCG: inconclusive (k = 9 studies) [Appendix 1, 2]<br>• OPV 3 coverage: inconclusive (k = 7 studies) [Appendix 1]<br>• Community health worker motivation, capacity and performance: favourable (k = 8 studies) [Appendix 1, 2]<br>• Stockouts: inconclusive (k = 4 studies) [Appendix 1]<br>• Experience and satisfaction with health services: favourable for overall satisfaction untargeted outcome (k = 4 studies) and inconclusive for targeted outcome (k = 2 studies) [Appendix 1]
Witter et al., 2012[67]	BD5. Pay-for-performance schemes	JAA15. Vaccination coverage (unspecified)	• Vaccination coverage (unspecified): (k = 2 studies): inconclusive, very low certainty evidence [p. 3, pp. 60-1, Table 8]
Oyo-Ita et al., 2016[63]	BF1. Health system strategic planning	JAA05. DPT3; JAA11. Measles; JAA03. DPT1; JAA02. BCG	• DPT3: favourable effect, low certainty (k = 1 study) [p.17]<br>• Measles: inconclusive effect, low certainty (k = 2 studies) [p.18]<br>• DPT 1: inconclusive effect, low certainty (k = 1 study) [p.18]<br>• BCG: inconclusive effect, low certainty (k = 1 study) [p.18]
Odendaal et al., 2018[62]	BF4. Health system financing	JAA15. Vaccination coverage (unspecified); ICA1. Stockouts	• Vaccination coverage (unspecified): little or no impact (k = 1 study), moderate certainty evidence [p.4 & p.21]<br>• Stockouts: inconclusive (k = 1 study) [refer p.22]
Lagarde & Palmer, 2009[57]	BF4. Health system financing	JAA01. Full routine immunization for children	• Full immunisation coverage: no significant impact (k = 1 study) [Table 2, p.18]

As in the case of caregiver-oriented intervention categories (A), we find a number of combinations where 3rd tier intervention categories are combined with each other. Table 13 below summarises these combinations in the context of health-systems oriented interventions (B). Table 13 is based on only 4 reviews. Gera et al. (2016)[53] and Willey et al. (2013)[66] focus on formal health worker training and education (BA1) and health system strategic planning (BF1) with Gera et al. (2016)[53] finding no evidence suggesting that DPT3 or measles coverage has improved while Willey et al. (2013)[66] find favourable evidence on vaccination (unspecified) and measles coverage. Both reviews rely on small study numbers (1-3 studies) not making any suggestions as to which one of the interventions components may have driven the results. Glenton et al. (2011)[54] looked at promoting outreach to vaccine-hesitant groups (BB4) and national/sub-national immunisation days (BC1) suggesting inconclusive effects on full routine immunisation for children and Jain et al. (2022)[41, 42] focus on outreach to vulnerable populations (hard-to-reach, SES, caste, etc.) (BB5) and collaborating with whole community (DA1) also presenting inconclusive effects. Jain et al. (2022)[41, 42] also looked at pay-for-

For peer review only

performance schemes (BD5) in combination with health system strategic planning (BF1) and collaborating with whole community (DA1), again, finding inconclusive effects drawing on small study numbers. It is not clear what we conclude from this rather heterogenous evidence base in relation to different combinations of health-systems oriented interventions (B) as the study numbers are too small, the outcomes too varied and the quality of the evidence base is questionable.

For peer review only

Table A10.2: B. Health-systems oriented interventions - more than one intervention (all high confidence systematic reviews)

Systematic review	Intervention, 3rd tier level	Outcome	Finding
Gera et al., 2016[53]	BA1. Formal health worker training and education; BF1. Health system strategic planning	JAA11. Measles; JAA05. DPT3	• DPT 3 coverage: little or no evidence of improved coverage (k = 2 studies) [p.18, Analysis 1.6]<br>• Measles coverage: little or no evidence of improved coverage, moderate certainty evidence (k = 3 studies) [p.18, Analysis 1.5
Willey et al., 2013[66]	BA1. Formal health worker training and education; BF1. Health system strategic planning	JAA15. Vaccination coverage (unspecified); JAA11. Measles;	• Vaccination coverage (unspecified): improved (k = 1 study) [p. 58, Table 3.5]<br>• Measles coverage: improved (k = 1 study) [p. 65, p.69, Table 3.6]
Glenton et al., 2011	BB4. Promoting outreach to vaccine-hesitant groups; BC1. National/sub-national immunisation days	JAA01. Full routine immunization for children	• Full routine immunization for children: inconclusive effect (k = 2 studies) [p.1050]
Jain et al., 2022[41, 42]	BB5. Outreach to vulnerable populations (hard-to-reach, SES, caste, etc.); DA1. Collaborating with whole community	HAD5. Vaccination health card availability/ retention	• Vaccination health cared availability/ retention: inconclusive (k = 4 studies) [see Supplementary Figure CECA, Appendix 8].
Jain et al., 2022[41, 42]	BD5. Pay-for-performance schemes; BF1. Health system strategic planning	IAB3. Formal HW motivation, capacity & performance	• Formal HW motivation, capacity & performance: inconclusive effect (k = 2 studies) [refer fig. 94, Appendix 8]
Jain et al., 2022[41, 42]	BD5. Pay-for-performance schemes; DA1. Collaborating with whole community	HAD4. Experience and satisfaction with health services	• Experience and satisfaction with health services: inconclusive (k = 2 studies) [see Supplementary Figure 17].

Notes: Oyo-Ita et al. (2016)[63] captures intervention combinations spanning across A. caregiver-oriented and B. health-systems oriented interventions (e.g., BB5. pay-for-performance schemes and AB1. material/ monetary incentives for caregivers; BF1. Health system strategic planning and AB1. material/ monetary incentives for caregivers), thus this study has been taken out from this table as captured in Table 11 already. Likewise, Glenton et al. (2011)[54] examining BB7. home visits And AA2. one-time sensitization and education campaigns. This table contains studies examining more than one intervention: Two health-system oriented interventions or one health system-oriented intervention with any other intervention.

For peer review only - http://bmjopen.bmj.com/site/about/guidelines.xhtml

Appendix 11: Community-level interventions

We are combining intervention categories C., D, and F. as we have very limited evidence on these categories. As none of this evidence contains sufficient quantitative information, we adopt a narrative approach. For other community-member oriented interventions (C), we have identified no high or medium confidence studies that provide information on this intervention category. As for community-level interventions (D), Table A7 summarises the only 2 high and medium reviews we have found for this intervention category. For multicomponent interventions (F), we have identified 2 reviews.[41, 42, 55] However, only Jain et al. (2022)[41, 42] contains information on community-level interventions (as well as on health-systems oriented interventions (B), thus Jain et al (2022)[41, 42] has been included into the discussion here.

Table A11: D. Community-level interventions

Systematic review	Confidence	Intervention, 3rd tier level	Outcome	Finding
Molina et al., 2016[61]	High	DA1. Collaborating with whole community	JAA15. Vaccination coverage (unspecified)	Vaccination coverage (unspecified): significantly improved (k = 2 studies) and inconclusive (k = 1 study) [P.60, table 10, Figure 7]
Desai et al., 2020[71]	Medium	DA2. Collaborating with selected community groups and networks	JAA15. Vaccination coverage (unspecified)	Vaccination coverage (unspecified): Inconclusive (k = 2 studies) [p.4]

Let us take a closer look at community-level interventions (D), we only have 2 reviews to consider, the one by Molina et al. (2016)[61] investigates collaborating with whole community (DA1) presenting mixed results, 2 of their included studies seem to suggest significant improvements for vaccination coverage (unspecified) while 1 of their studies suggests inconclusive effects for the same outcome. Jain et al. (2022)[41, 42] have also examined collaborating with whole community (DA1) in relation to vaccination health card availability and satisfaction with health services finding inconclusive effects. The other study listed in Table A8 is the one by Desai et al. (2020)[71] which is of medium confidence and focusing on collaborating with selected community groups and networks (DA2). The outcome of interest is vaccination coverage (unspecified), and the results are inconclusive drawing on 2 studies. These results do not fill us with much confidence with regard to understanding what type of community-level interventions are effective in terms of improving immunisation outcomes. Looking at Jain et al. (2022)[41, 42] more closely, we can detect some favourable effects. They use a nuanced framework to classify single and multicomponent interventions based on the process of engaging communities, which does not fit neatly into the community-oriented categorisation of interventions used in this review. Nevertheless, drawing on 28 studies, which are a combination of demand and supply side interventions that include aspects of caregiver-oriented interventions (A) focusing on education, incentives, written messages and general community engagement, have favourable effects on full immunisation coverage. The same combination of interventions has favourable effects also on DPT3 vaccination coverage (drawing on 22 studies), on measles coverage (20 studies), on BCG (12 studies), on partial immunisation, OPV3, and immunisation knowledge (all 9 studies) as well as on vaccination timeliness of DPT3 (7 studies) and on vaccination timeliness for full immunisation, DPT2, OPV1 and 2 (all 5 studies). However, these findings confirm our previous observations, namely that the evidence base is highly heterogenous in terms of intervention categories and outcomes, it is also relying on a very limited pool of studies with varying quality. Except for caregiver-oriented interventions (A), we have an insufficient knowledge of the effectiveness of health-systems oriented interventions (B) and single community-level interventions (C and D). We found no evidence on non-health related policies and institutions (E) and have yet to understand which interventions best complement one another to enhance immunisation outcomes.

For peer review only

References

1. Asciutto R, Di Napoli A, Vecchi S, Sicuro J, Mirisola C, Petrelli A. A systematic review of economic evaluations of neonatal and maternal healthcare in immigrant and ethnic minority women. Epidemiologia e prevenzione. 2020;44(5-6 Suppl 1):142-52.
2. Balzarini F, Frascella B, Oradini-Alacreu A, Gaetti G, Lopalco PL, Edelstein M, et al. Does the use of personal electronic health records increase vaccine uptake? A systematic review. Vaccine. 2020;38(38):5966-78.
3. Diks ME, Hiligsmann M, van der Putten IM. Vaccine preferences driving vaccine-decision making of different target groups: a systematic review of choice-based experiments. BMC infectious diseases. 2021;21(1):879.
4. Haroune V, King L. Factors contributing to parental 'vaccine hesitancy' for childhood immunisations. Nursing children and young people. 2020;32(4):20-5.
5. Hutchinson AF, Smith SM. Effectiveness of strategies to increase uptake of pertussis vaccination by new parents and family caregivers: A systematic review. Midwifery. 2020;87:102734.
6. Jia L, Meng Q, Scott A, Yuan B, Zhang L. Payment methods for healthcare providers working in outpatient healthcare settings. The Cochrane database of systematic reviews. 2021;1(1):Cd011865.
7. Lambert JF, Stete K, Balmford J, Bockey A, Kern W, Rieg S, et al. Reducing burden from respiratory infections in refugees and immigrants: a systematic review of interventions in OECD, EU, EEA and EU-applicant countries. BMC infectious diseases. 2021;21(1):872.
8. Machado AA, Edwards SA, Mueller M, Saini V. Effective interventions to increase routine childhood immunization coverage in low socioeconomic status communities in developed countries: A systematic review and critical appraisal of peer-reviewed literature. Vaccine. 2021;39(22):2938-64.
9. Odone A, Dallagiacoma G, Frascella B, Signorelli C, Leask J. Current understandings of the impact of mandatory vaccination laws in Europe. Expert review of vaccines. 2021;20(5):559-75.
10. Ortiz-Sánchez E, Velando-Soriano A, Pradas-Hernández L, Vargas-Román K, Gómez-Urquiza JL, Cañadas-De la Fuente GA, et al. Analysis of the Anti-Vaccine Movement in Social Networks: A Systematic Review. International journal of environmental research and public health. 2020;17(15).
11. Pires C. What Is the State-of-the-Art in Clinical Trials on Vaccine Hesitancy 2015-2020? Vaccines. 2021;9(4).
12. Reñosa MDC, Landicho J, Wachinger J, Dalglish SL, Bärnighausen K, Bärnighausen T, et al. Nudging toward vaccination: a systematic review. BMJ Glob Health. 2021;6(9).
13. Bednarek A, Klepacz R. Vaccinology Education of Nurses and the Current Immunoprophylaxis Recommendations for Children with Juvenile Idiopathic Arthritis. Journal of clinical medicine. 2020;9(11).
14. Chenet SM, Tapia-Limonchi R. Reaching the theoretical herd immunity threshold in Iquitos, Peru: are seroprevalence data enough? The Lancet Global health. 2021;9(7):e881-e2.
15. Chitkara AJ, Pujadas Ferrer M, Forsyth K, Guiso N, Heininger U, Hozbor DF, et al. Pertussis vaccination in mixed markets: Recommendations from the Global Pertussis Initiative. International journal of infectious diseases : IJID : official publication of the International Society for Infectious Diseases. 2020;96:482-8.
16. Meteke S, Stefopulos M, Als D, Gaffey M, Kamali M, Siddiqui FJ, et al. Delivering infectious disease interventions to women and children in conflict settings: a systematic reviefw. BMJ Glob Health. 2020;5(Suppl 1).
17. Nnaji CA, Wiysonge CS, Okeibunor JC, Malinga T, Adamu AA, Tumusiime P, et al. Implementation research approaches to promoting universal health coverage in Africa: a scoping review. BMC Health Serv Res. 2021;21(1):414.
18. Chaney SC, Mechael P, Thu NM, Diallo MS, Gachen C. Every Child on the Map: A Theory of Change Framework for Improving Childhood Immunization Coverage and Equity Using Geospatial Data and Technologies. Journal of medical Internet research. 2021;23(8):e29759.
19. Soans S, Mihalyi A, Berlaimont V, Kolhapure S, Dash R, Agrawal A. Vaccination in preterm and low birth weight infants in India. Human vaccines & immunotherapeutics. 2022;18(1):1-12.
20. Tankwanchi AS, Jaca A, Larson HJ, Wiysonge CS, Vermund SH. Taking stock of vaccine hesitancy among migrants: a scoping review protocol. BMJ open. 2020;10(5):e035225.
21. Agrawal A, Kolhapure S, Di Pasquale A, Rai J, Mathur A. Vaccine Hesitancy as a Challenge or Vaccine Confidence as an Opportunity for Childhood Immunisation in India. Infectious diseases and therapy. 2020;9(3):421-32.
22. de Souza L, da Silva Jacob L, de Souza Lucena E, de Oliveira Costa R. Experiências brasileiras no acompanhamento de crescimento e desenvolvimento infantil no contexto da Atenção Básica. Enfermagem em Foco. 2021;12(2).
23. Berrueta M, Ciapponi A, Bardach A, Cairoli FR, Castellano FJ, Xiong X, et al. Maternal and neonatal data collection systems in low- and middle-income countries for maternal vaccines active safety surveillance systems: A scoping review. BMC pregnancy and childbirth. 2021;21(1):217.

For peer review only

24. Cassocera M, Chissaque A, Martins MRO, Deus N. 40 years of immunization in Mozambique: a narrative review of literature, accomplishments, and perspectives. Cadernos de saude publica. 2020;36Suppl 2(Suppl 2):e00038320.
25. Dansereau E, Brown D, Stashko L, Danovaro-Holliday MC. A systematic review of the agreement of recall, home-based records, facility records, BCG scar, and serology for ascertaining vaccination status in low and middle-income countries. Gates open research. 2019;3:923.
26. Farida Farida, Vitri Widyaningsih, Murti B. The Effect of Maternal Education and Antenatal Care on Basic Immunization Completeness in Children aged 12-23 Months in Asian and African: Meta-Analysis. Journal of Maternal and Child Health. 2020;5(6):614-28.
27. Galadima AN, Zulkefli NAM, Said SM, Ahmad N. Factors influencing childhood immunisation uptake in Africa: a systematic review. BMC public health. 2021;21(1):1475-.
28. Kalaij AGI, Sugiyanto M, Ilham AF. Factors Associated With Vaccination Compliance in Southeast Asian Children: A Systematic Review. Asia-Pacific journal of public health. 2021;33(5):479-88.
29. Kulkarni S, Thampi V, Deshmukh D, Gadhari M, Chandrasekar R, Phadke M. Trends in Urban Immunization Coverage in India: A Meta-Analysis and Meta-Regression. Indian journal of pediatrics. 2021:1-11.
30. Laserna Jiménez C, López Poyato M, Casado Montañés I, Guix-Comellas EM, Fabrellas N. Paediatric nursing clinical competences in primary healthcare: A systematic review. Journal of advanced nursing. 2021;77(6):2662-79.
31. Lassi ZS, Naseem R, Salam RA, Siddiqui F, Das JK. The Impact of the COVID-19 Pandemic on Immunization Campaigns and Programs: A Systematic Review. International journal of environmental research and public health. 2021;18(3).
32. Lieneck C, Herzog B, Krips R. Analysis of Facilitators and Barriers to the Delivery of Routine Care during the COVID-19 Global Pandemic: A Systematic Review. Healthcare (Basel, Switzerland). 2021;9(5).
33. Wariri O, Okomo U, Kwarshak YK, Murray KA, Grundy C, Kampmann B. Timeliness of routine childhood vaccination in low- and middle-income countries, 1978-2021: Protocol for a scoping review to map methodologic gaps and determinants. PloS one. 2021;16(6):e0253423.
34. Priya PK, Pathak VK, Giri AK. Vaccination coverage and vaccine hesitancy among vulnerable population of India. Human vaccines & immunotherapeutics. 2020;16(7):1502-7.
35. Smith LE, Hodson A, Rubin GJ. Parental attitudes towards mandatory vaccination; a systematic review. Vaccine. 2021;39(30):4046-53.
36. Besnier E, Thomson K, Stonkute D, Mohammad T, Akhter N, Todd A, et al. Which public health interventions are effective in reducing morbidity, mortality and health inequalities from infectious diseases amongst children in low- and middle-income countries (LMICs): An umbrella review. PloS one. 2021;16(6):e0251905.
37. Munk C, Portnoy A, Suharlim C, Clarke-Deelder E, Brenzel L, Resch SC, et al. Systematic review of the costs and effectiveness of interventions to increase infant vaccination coverage in low- and middle-income countries. BMC Health Services Research. 2019;19(1):741.
38. Omoniyi OS, Williams I. Realist Synthesis of the International Theory and Evidence on Strategies to Improve Childhood Vaccination in Low- and Middle-Income Countries: Developing Strategies for the Nigerian Healthcare System. International journal of health policy and management. 2020;9(7):274-85.
39. Siribaddana P, Sahay S, Hewapathirana R. Deploying Electronic Immunization Information Systems in Low and Middle Income Countries in Response to COVID-19: Systematic Review of Best Evidence Practices. SSRN; 2021.
40. Jain M, Engelbert M, Gaarder M, Bagai A, Eyers J. PROTOCOL: Use of community participation interventions to improve child immunisation in low- and middle-income countries: A systematic review and meta-analysis. Campbell Systematic Reviews. 2020;16(4):e1119.
41. Jain M, Shisler S, Lane C, Bagai A, Brown E, Engelbert M. Use of community engagement interventions to improve child immunisation in low-income and middle-income countries: a systematic review and meta-analysis. BMJ open. 2022;12(11):e061568.
42. Jain M, Shisler S, Lane C, Bagai A, Brown E, Engelbert M, et al. Use of community engagement interventions to improve child immunisation in low- and middle-income countries: A systematic review and meta-analysis. Campbell Systematic Reviews. 2022;18(3):e1253.
43. Onwuchekwa C, Verdonck K, Marchal B. Systematic Review on the Impact of Conditional Cash Transfers on Child Health Service Utilisation and Child Health in Sub-Saharan Africa. Frontiers in public health. 2021;9:643621.
44. Eze P, Lawani LO, Acharya Y. Short message service (SMS) reminders for childhood immunisation in low-income and middle-income countries: a systematic review and meta-analysis. BMJ Global Health. 2021;6(7):e005035.

For peer review only

45. Pantoja T, Opiyo N, Lewin S, Paulsen E, Ciapponi A, Wiysonge CS, et al. Implementation strategies for health systems in low-income countries: an overview of systematic reviews. The Cochrane database of systematic reviews. 2017;9(9):Cd011086.
46. Hatt L, Johns B, Connor C, Meline M, Kukla M, Moat K. Impact of health systems strengthening on health. Bethesda, MD: Health Finance and Governance Project, Abt Associates Inc; 2015.
47. Wiysonge S. Building evidence for improving childhood immunisation coverage in Africa: University of Cape Town; 2012.
48. Van Velthoven MH, Milne-Ives M, de Cock C, Mooney M, Meinert E. Use of Apps to Promote Childhood Vaccination: Protocol for a Systematic Review. JMIR research protocols. 2020;9(2):e16929.
49. Abiola Abdulrahman S, Olaosebikan MO. mHealth: a narrative synthesis of evidence of its application in improving childhood immunization coverage. Journal of Hospital Management and Health Policy. 2017;1(4).
50. Aaby P, Ravn H, Benn CS, Rodrigues A, Samb B, Ibrahim SA, et al. Randomized Trials Comparing Inactivated Vaccine After Medium- or High-titer Measles Vaccine With Standard Titer Measles Vaccine After Inactivated Vaccine: A Meta-analysis. The Pediatric Infectious Disease Journal. 2016;35(11).
51. Bhutta ZA, Ali S, Cousens S, Ali TM, Haider BA, Rizvi A, et al. Alma-Ata: Rebirth and Revision 6 Interventions to address maternal, newborn, and child survival: what difference can integrated primary health care strategies make? Lancet (London, England). 2008;372(9642):972-89.
52. Diaconu K, Falconer J, Verbel A, Fretheim A, Witter S. Paying for performance to improve the delivery of health interventions in low- and middle-income countries. The Cochrane database of systematic reviews. 2021;5(5):Cd007899.
53. Gera T, Shah D, Garner P, Richardson M, Sachdev HS. Integrated management of childhood illness (IMCI) strategy for children under five. The Cochrane database of systematic reviews. 2016(6):Cd010123.
54. Glenton C, Scheel IB, Lewin S, Swingler GH. Can lay health workers increase the uptake of childhood immunisation? Systematic review and typology. Tropical medicine & international health : TM & IH. 2011;16(9):1044-53.
55. Johri M, Pérez MC, Arsenault C, Sharma JK, Pai NP, Pahwa S, et al. Strategies to increase the demand for childhood vaccination in low- and middle-income countries: a systematic review and meta-analysis. Bulletin of the World Health Organization. 2015;93(5):339-46c.
56. Kaufman J, Ryan R, Walsh L, Horey D, Leask J, Robinson P, et al. Face-to-face interventions for informing or educating parents about early childhood vaccination. The Cochrane database of systematic reviews. 2018;5(5):Cd010038.
57. Lagarde M, Palmer N. The impact of contracting out on health outcomes and use of health services in low and middle-income countries. The Cochrane database of systematic reviews. 2009(4):Cd008133.
58. Lagarde M, Haines A, Palmer N. The impact of conditional cash transfers on health outcomes and use of health services in low and middle income countries. The Cochrane database of systematic reviews. 2009;2009(4):Cd008137.
59. Linde DS, Korsholm M, Katanga J, Rasch V, Lundh A, Andersen MS. One-way SMS and healthcare outcomes in Africa: Systematic review of randomised trials with meta-analysis. PloS one. 2019;14(6):e0217485.
60. Mekonnen ZA, Gelaye KA, Were MC, Gashu KD, Tilahun BC. Effect of mobile text message reminders on routine childhood vaccination: a systematic review and meta-analysis. Systematic reviews. 2019;8(1):154.
61. Molina E, Carella L, Pacheco A, Cruces G, Gasparini L. Community monitoring interventions to curb corruption and increase access and quality of service delivery in low- and middle-income countries: a systematic review. Campbell Systematic Reviews. 2016;12(1):1-204.
62. Odendaal WA, Ward K, Uneke J, Uro-Chukwu H, Chitama D, Balakrishna Y, et al. Contracting out to improve the use of clinical health services and health outcomes in low- and middle-income countries. The Cochrane database of systematic reviews. 2018;4(4):Cd008133.
63. Oyo-Ita A, Wiysonge CS, Oringanje C, Nwachukwu CE, Oduwole O, Meremikwu MM. Interventions for improving coverage of childhood immunisation in low- and middle-income countries. The Cochrane database of systematic reviews. 2016;7(7):Cd008145.
64. Palmer MJ, Henschke N, Bergman H, Villanueva G, Maayan N, Tamrat T, et al. Targeted client communication via mobile devices for improving maternal, neonatal, and child health. The Cochrane database of systematic reviews. 2020;8(8):Cd013679.
65. Saeterdal I, Lewin S, Austvoll-Dahlgren A, Glenton C, Munabi-Babigumira S. Interventions aimed at communities to inform and/or educate about early childhood vaccination. The Cochrane database of systematic reviews. 2014(11):Cd010232.
66. Willey B, Smith Paintain L, Mangham-Jefferies L, Car J, Armstrong Schellenberg J. Effectiveness of interventions to strengthen national health service delivery on coverage, access, quality and equity in the use of health services in low and lower middle income countries. Armstrong: EPPI-Centre, Social Science Research Unit, Institute of Education, University of London; 2013.

For peer review only - http://bmjopen.bmj.com/site/about/guidelines.xhtml

67. Witter S, Fretheim A, Kessy FL, Lindahl AK. Paying for performance to improve the delivery of health interventions in low- and middle-income countries The Cochrane database of systematic reviews. 2012(2):Cd007899.
68. Atkinson KM, Wilson K, Murphy MSQ, El-Halabi S, Kahale LA, Laflamme LL, et al. Effectiveness of digital technologies at improving vaccine uptake and series completion - A systematic review and meta-analysis of randomized controlled trials. Vaccine. 2019;37(23):3050-60.
69. Bassani DG, Arora P, Wazny K, Gaffey MF, Lenters L, Bhutta ZA. Financial incentives and coverage of child health interventions: a systematic review and meta-analysis. BMC public health. 2013;13 Suppl 3(Suppl 3):S30.
70. Chutiyami M, Wyver S, Amin J. Are Parent-Held Child Health Records a Valuable Health Intervention? A Systematic Review and Meta-Analysis. International journal of environmental research and public health. 2019;16(2).
71. Desai S, Misra M, Das A, Singh RJ, Sehgal M, Gram L, et al. Community interventions with women's groups to improve women's and children's health in India: a mixed-methods systematic review of effects, enablers and barriers. BMJ Glob Health. 2020;5(12).
72. Gilmore B, McAuliffe E. Effectiveness of community health workers delivering preventive interventions for maternal and child health in low- and middle-income countries: a systematic review. BMC public health. 2013;13:847.
73. Lukusa LA, Ndze VN, Mbeye NM, Wiysonge CS. A systematic review and meta-analysis of the effects of educating parents on the benefits and schedules of childhood vaccinations in low and middle-income countries. Human vaccines & immunotherapeutics. 2018;14(8):2058-68.
74. Baequni NY. Is Maternal and Child Health Handbook effective? : Meta-Analysis of the Effects of MCH Handbook. Kokusai Hoken Iryo (Journal of International Health). 2012;27(2):121-7.
75. Batt K, Fox-Rushby JA, Castillo-Riquelme M. The costs, effects and cost-effectiveness of strategies to increase coverage of routine immunizations in low- and middle-income countries: systematic review of the grey literature. Bulletin of the World Health Organization. 2004;82(9):689-96.
76. Bright T, Felix L, Kuper H, Polack S. A systematic review of strategies to increase access to health services among children in low and middle income countries. BMC Health Services Research. 2017;17(1):252.
77. Crocker-Buque T, Mindra G, Duncan R, Mounier-Jack S. Immunization, urbanization and slums - a systematic review of factors and interventions. BMC public health. 2017;17(1):556.
78. Das A, Gopalan SS, Chandramohan D. Effect of pay for performance to improve quality of maternal and child care in low- and middle-income countries: a systematic review. BMC public health. 2016;16:321.
79. de Cock C, van Velthoven M, Milne-Ives M, Mooney M, Meinert E. Use of Apps to Promote Childhood Vaccination: Systematic Review. JMIR mHealth and uHealth. 2020;8(5):e17371.
80. de Souza Cruz RC, Moura LBA, Soares Neto JJ. Conditional cash transfers and the creation of equal opportunities of health for children in low and middle-income countries: a literature review. International journal for equity in health. 2017;16(1):161.
81. Deardorff KV, Rubin Means A, Ásbjörnsdóttir KH, Walson J. Strategies to improve treatment coverage in community-based public health programs: A systematic review of the literature. PLoS neglected tropical diseases. 2018;12(2):e0006211.
82. Freeman PA, Schleiff M, Sacks E, Rassekh BM, Gupta S, Perry HB. Comprehensive review of the evidence regarding the effectiveness of community-based primary health care in improving maternal, neonatal and child health: 4. child health findings. Journal of global health. 2017;7(1):010904.
83. Giedion U, Andrés Alfonso E, Díaz Y. The impact of universal coverage schemes in the developing world: a review of the existing evidence. Washington DC: World Bank; 2013. Contract No.: 25.
84. Harvey H, Reissland N, Mason J. Parental reminder, recall and educational interventions to improve early childhood immunisation uptake: A systematic review and meta-analysis. Vaccine. 2015;33(25):2862-80.
85. Iwu CJ, Jaca A, Abdullahi LH, Ngcobo NJ, Wiysonge CS. A scoping review of interventions for vaccine stock management in primary health-care facilities. Human vaccines & immunotherapeutics. 2019;15(11):2666-72.
86. Jarrett C, Wilson R, O'Leary M, Eckersberger E, Larson HJ. Strategies for addressing vaccine hesitancy - A systematic review. Vaccine. 2015;33(34):4180-90.
87. Karageorgos G, Andreadis I, Psychas K, Mourkousis G, Kiourti A, Lazzi G, et al. The Promise of Mobile Technologies for the Health Care System in the Developing World: A Systematic Review. IEEE reviews in biomedical engineering. 2019;12:100-22.
88. Kim SS, Patel M, Hinman A. Use of m-Health in polio eradication and other immunization activities in developing countries. Vaccine. 2017;35(10):1373-9.
89. Loevinsohn B, & , Harding A. Contracting for the delivery of community health services: A review of Global Experience. Washington, DC: World Bank; 2004.

For peer review only

90. Mureed S, Somronghtong R, Kumar R, Ghaffar A, Chapman RS. Enhanced immunization coverage through interventions for childhood cluster diseases in developing countries. Journal of Ayub Medical College, Abbottabad : JAMC. 2015;27(1):223-7.
91. Naugle DA, Hornik RC. Systematic review of the effectiveness of mass media interventions for child survival in low- and middle-income countries. Journal of health communication. 2014;19 Suppl 1(sup1):190-215.
92. Nelson KN, Wallace AS, Sodha SV, Daniels D, Dietz V. Assessing strategies for increasing urban routine immunization coverage of childhood vaccines in low and middle-income countries: A systematic review of peer-reviewed literature. Vaccine. 2016;34(46):5495-503.
93. Nutman S, McKee D, Khoshnood K. Externalities of prevention of mother-to-child transmission programs: a systematic review. AIDS and behavior. 2013;17(2):445-60.
94. Oliver-Williams C, Brown E, Devereux S, Fairhead C, Holeman I. Using Mobile Phones to Improve Vaccination Uptake in 21 Low- and Middle-Income Countries: Systematic Review. JMIR mHealth and uHealth. 2017;5(10):e148.
95. Owusu-Addo E, Cross R. The impact of conditional cash transfers on child health in low- and middle-income countries: a systematic review. International journal of public health. 2014;59(4):609-18.
96. Ozawa S, Yemeke TT, Thompson KM. Systematic review of the incremental costs of interventions that increase immunization coverage. Vaccine. 2018;36(25):3641-9.
97. Pal M, Goodyear-Smith F, Exeter D. Systematic review of pertussis immunisation among Asians. International Journal of Human Rights in Healthcare. 2016;9(2):135-46.
98. Parrado Jiménez D. Revisión sistemática de la literatura del efecto de pago por desempeño en la calidad de la atención en países de bajos y medianos ingresos. Bogota Colombia Universidad de Los Andes; 2013.
99. Pegurri E, Fox-Rushby JA, Damian W. The effects and costs of expanding the coverage of immunisation services in developing countries: a systematic literature review. Vaccine. 2005;23(13):1624-35.
100. Pinzón Flórez C, Díaz-Quijano D, Yáñez Álvarez I, Catalina Mesa D. Effectiveness of community workers on preventive measures to maternal and child health in low and middle income countries: systematic review of the literature. Revista Salud Uninorte. 2015;31(2):309-28.
101. Rahman A, Fisher J, Bower P, Luchters S, Tran T, Yasamy MT, et al. Interventions for common perinatal mental disorders in women in low- and middle-income countries: a systematic review and meta-analysis. Bulletin of the World Health Organization. 2013;91(8):593-601i.
102. Ranganathan M, Lagarde M. Promoting healthy behaviours and improving health outcomes in low and middle income countries: a review of the impact of conditional cash transfer programmes. Preventive medicine. 2012;55 Suppl:S95-s105.
103. Ryman TK, Dietz V, Cairns KL. Too little but not too late: results of a literature review to improve routine immunization programs in developing countries. BMC Health Serv Res. 2008;8:134.
104. Shea B, Andersson N, Henry D. Increasing the demand for childhood vaccination in developing countries: a systematic review. BMC international health and human rights. 2009;9 Suppl 1(Suppl 1):S5.
105. Smith BL, Zizzo S, Amzel A, Wiant S, Pezzulo MC, Konopka S, et al. Integration of Neonatal and Child Health Interventions with Pediatric HIV Interventions in Global Health. International journal of MCH and AIDS. 2018;7(1):192-206.
106. Watterson JL, Walsh J, Madeka I. Using mHealth to Improve Usage of Antenatal Care, Postnatal Care, and Immunization: A Systematic Review of the Literature. BioMed research international. 2015;2015:153402.
107. Yuan B, Målqvist M, Trygg N, Qian X, Ng N, Thomsen S. What interventions are effective on reducing inequalities in maternal and child health in low- and middle-income settings? A systematic review. BMC public health. 2014;14:634.
108. Yunusa U, Garba SN, Umar AB, Idris SH, Bello UL, Abdulrashid I, et al. Mobile phone reminders for enhancing uptake, completeness and timeliness of routine childhood immunization in low and middle income countries: A systematic review and meta-analysis. Vaccine. 2021;39(2):209-21.
109. Duvendack M. Payment-by-results for health interventions in low- and middle-income countries: A critical review. Development Policy Review. 2022;40(1):e12538.

For peer review only

BMJ Open

Effective interventions for improving routine childhood immunisation in low- and middle-income countries: A systematic review of systematic reviews

Journal:	*BMJ Open*
Manuscript ID	bmjopen-2023-074370.R1
Article Type:	Original research
Date Submitted by the Author:	20-Jan-2024
Complete List of Authors:	Jain, Monica; International Initiative for Impact Evaluation Dehli<br>Duvendack, Maren; University of East Anglia<br>Shisler, Shannon; International Initiative for Impact Evaluation<br>Parsekar, Shradha; International Initiative for Impact Evaluation Dehli<br>Leon, Maria Daniela ; International Initiative for Impact Evaluation
**Primary Subject Heading**:	Evidence based practice
Secondary Subject Heading:	Health policy, Health services research, Public health, Paediatrics
Keywords:	Community child health < PAEDIATRICS, Paediatric infectious disease & immunisation < PAEDIATRICS, Health policy < HEALTH SERVICES ADMINISTRATION & MANAGEMENT, Systematic Review

SCHOLARONE™
Manuscripts

I, the Submitting Author has the right to grant and does grant on behalf of all authors of the Work (as defined in the below author licence), an exclusive licence and/or a non-exclusive licence for contributions from authors who are: i) UK Crown employees; ii) where BMJ has agreed a CC-BY licence shall apply, and/or iii) in accordance with the terms applicable for US Federal Government officers or employees acting as part of their official duties; on a worldwide, perpetual, irrevocable, royalty-free basis to BMJ Publishing Group Ltd ("BMJ") its licensees and where the relevant Journal is co-owned by BMJ to the co-owners of the Journal, to publish the Work in this journal and any other BMJ products and to exploit all rights, as set out in our licence.

The Submitting Author accepts and understands that any supply made under these terms is made by BMJ to the Submitting Author unless you are acting as an employee on behalf of your employer or a postgraduate student of an affiliated institution which is paying any applicable article publishing charge ("APC") for Open Access articles. Where the Submitting Author wishes to make the Work available on an Open Access basis (and intends to pay the relevant APC), the terms of reuse of such Open Access shall be governed by a Creative Commons licence – details of these licences and which Creative Commons licence will apply to this Work are set out in our licence referred to above.

Other than as permitted in any relevant BMJ Author's Self Archiving Policies, I confirm this Work has not been accepted for publication elsewhere, is not being considered for publication elsewhere and does not duplicate material already published. I confirm all authors consent to publication of this Work and authorise the granting of this licence.

For peer review only

Effective interventions for improving routine childhood immunisation in low- and middle-income countries: A systematic review of systematic reviews

Monica Jain, Maren Duvendack, Shannon Shisler, Shradha S Parsekar, Maria Daniela Anda Leon

International Initiative for Impact Evaluation (Monica Jain PhD, Shannon Shisler PhD, Shradha S Parsekar MPH and Maria Daniela Anda Leon) and **University of East Anglia, Norwich, UK** (Maren Duvendack PhD)

Correspondence to:

Dr. Monica Jain

Evaluation Office, International Initiative for Impact Evaluation (3ie), New Delhi, 110017, India

Email: mjain@3ieimpact.org

Word count (excluding title page, abstract, strengths and limitations, tables, references, declarations etc.): 3692.

Abstract: 366 words

For peer review only

ABSTRACT

Objective: An umbrella review providing a comprehensive synthesis of the interventions that are effective in providing routine immunisation outcomes for children in low- and middle-income countries (L&MICs).

Design: A systematic review of systematic reviews, or an umbrella review.

Data sources: We comprehensively searched 11 academic databases and 23 grey literature sources. The search was adopted from an evidence gap map on routine child immunisation sector in L&MICs, which was done on 5 May 2020. We updated the search in October 2021.

Eligibility criteria: We included systematic reviews assessing the effectiveness of any intervention on routine childhood immunization outcomes in L&MICs.

Data extraction and synthesis: Search results were screened by two reviewers independently applying pre-defined inclusion and exclusion criteria. Data were extracted by two researchers independently. The SURE checklist was used to assess review quality. A mixed-methods synthesis was employed focusing on meta-analytical and narrative elements to accommodate both the quantitative and qualitative information available from the included reviews.

Results: 62 systematic reviews are included in this umbrella review. We find caregiver-oriented interventions have large positive and statistically significant effects, especially those focusing on short-term sensitisation and education campaigns as well as written messages to caregivers. For health-systems oriented interventions the evidence base is thin and derived from narrative synthesis suggesting positive effects for home visits, mixed effects for pay for performance schemes and inconclusive effects for contracting out services to non-governmental providers. For all other interventions under this category, the evidence is either limited or not available. For community-oriented interventions, a recent high-quality mixed-

For peer review only

methods review suggests positive but small effects. Overall, the evidence base is highly heterogenous in terms of scope, intervention types, and outcomes.

Conclusion: Interventions oriented towards caregivers and communities are effective in improving routine child immunisation outcomes. The evidence base on health-systems oriented interventions is scant not allowing us to reach firm conclusions, except for home visits. Large evidence gaps exist and need to be addressed. For example, more high-quality evidence is needed for specific caregiver-oriented interventions (e.g., monetary incentives) as well as health systems-oriented (e.g., health workers and data systems), and community-oriented interventions. We also need to better understand complementarity of different intervention types.

Funding: Bill & Melinda Gates Foundation (INV-008461).

Keywords: Immunisation; Immunisation Programmes; Mass Vaccination; Children and Adolescents; Poverty Areas

STRENGTHS AND LIMITATIONS OF THIS STUDY

- This review synthesises the evidence base on routine child immunisation interventions in L&MICs at a higher level of abstraction (i.e., at the systematic review of systematic reviews level), thus filling a gap in the literature.
- Our review uses an intervention taxonomy that was developed and refined based on existing literature, extensive discussion, pilot testing, and expert feedback allowing an assessment of interventions oriented towards caregivers, health systems, communities as well as those outside the health realm separately.
- Our review synthesises the evidence both quantitatively through meta-analysis as well as qualitatively, therefore providing insights into not only where evidence exists but also point out critical evidence synthesis gaps.

For peer review only - http://bmjopen.bmj.com/site/about/guidelines.xhtml

- The review cautions on the interpretation of our findings because of methodological challenges, including mixed quality of the included systematic reviews and of the primary studies that have included in their synthesis as well as small sample bias.

For peer review only

For peer review only - http://bmjopen.bmj.com/site/about/guidelines.xhtml

INTRODUCTION

In 2019, close to 20 million children in low-and middle-income countries (L&MICs) did not receive the three recommended doses of diphtheria, pertussis and tetanus (DPT) vaccines, often used as an indicator to assess countries' performance on routine immunisation.[1] Ten countries, in particular Nigeria, India, the Democratic Republic of Congo, and Pakistan, account for two out of five unvaccinated children globally.[2] Many L&MICs struggle to achieve high immunisation coverage due to challenges on both the supply and demand side. Examples of supply side constraints include limited availability of health personnel and difficulties in building up their capacity and skills as well as lack of reliable monitoring and surveillance systems to identify and monitor un- and under-vaccinated children.[3] Demand-side issues linked to behavioural, social and practical constraints faced by caregivers also play a role, including concerns about vaccine safety, lack of knowledge of when and where to vaccinate their children, fears about vaccination side effects and difficulties in accessing health services.[4] Governments in L&MICs have strengthened national-level immunisation interventions to address supply side constraints and are increasingly focusing on addressing constraints faced by caregivers and communities. To support policy makers and practitioners in making evidence informed decisions on the interventions that have been effective in addressing the barriers to routine vaccination uptake, it is imperative to provide systematically synthesised evidence.

Several methodologically robust impact evaluations and systematic reviews evaluating the effectiveness of these approaches have been published in the past decade.[5-12] However, there has been no attempt yet to synthesise this extensive evidence base at the meta-level with the exception of Heneghan et al.[13] and Besnier et al.[14] First, Heneghan et al. qualitatively synthesise child and adult immunisation interventions in both L&MICs and high-income countries (HICs) finding inconclusive results for L&MICs due to the small number of reviews

For peer review only - http://bmjopen.bmj.com/site/about/guidelines.xhtml

for these countries, inadequate definitions of interventions and sub-optimal reporting of interventions in the reviews. Second, Besnier et al. examine interventions focusing on improving child health using narrative synthesis tools; they find positive effects of interventions improving immunisation communication, education and social mobilisation on vaccine uptake. While the evidence base on this topic is growing rapidly, Engelbert et al.[15] demonstrate that there are clear evidence gaps preventing us from fully understanding what type of immunisation interventions work best, for whom, how and where. This is the first systematic review of systematic reviews with the primary goal to provide more clarity on the type of interventions that are effective in improving routine immunisation outcomes for children in L&MICs. Establishing what is and is not known about the effectiveness of such interventions is crucially important to inform policymakers and practitioners.

METHODS

Search strategy and selection criteria

This is a systematic review of systematic reviews adopting a mixed-methods approach including a meta-analysis and narrative elements. It draws on the intervention-outcome framework (Supplementary file - Appendix 1) developed by Engelbert et al.[15] which distinguishes interventions by different levels, i.e., 1st, 2nd and 3rd tier focusing on the supply side (health-system oriented) as well as the demand side (caregiver-oriented). It also allows for the identification of community-oriented interventions as well as for those which are non-health policy oriented. This framework guides the presentation of our results. In terms of study design, we included systematic reviews with and without meta-analyses. The population of interest included children below the age of five living in L&MICs, but we occasionally included other populations, e.g., caregivers and health workers, especially when these were relevant to understanding the impact of immunisation programmes on intermediate outcomes

For peer review only - http://bmjopen.bmj.com/site/about/guidelines.xhtml

such as attitudes about vaccination, and access to immunisation services. We included studies examining the impact of any intervention on at least one outcome in relation to routine child immunisation. These outcomes are coverage rates or timeliness of full immunisation, third dose of DPT or pentavalent, or measles; additional antigen-specific immunisation coverage outcomes; and intermediate outcomes that precede them in the theoretical causal chain (e.g., attitudes about vaccination, and access to immunisation services). The detailed eligibility criteria can be found in Supplementary file - Appendix 2. There were no inclusion restrictions by publication status or language, but studies were excluded when they did not meet our definition of systematic reviews, when they were not effectiveness or intervention reviews, or when they only focused on HICs (Appendix 3).

We adopted a comprehensive search strategy that was initially designed for an evidence gap map (EGM)[15] examining the routine child immunisation sector in L&MICs. The search strategy included electronic searches of academic databases and the grey literature, i.e., institutional websites (Appendix 4). The search was completed in October 2021. All search results were screened on title/abstract by a team of trained reviewers. Two reviewers independently screened each abstract. During title and abstract screening, weekly reconciliation meetings were held to discuss and resolve disagreements. Full text screening followed the same approach.

Data analysis

Data were extracted by two researchers independently with reconciliation by others in the review team. Any disagreements were resolved by discussion. We extracted data on context, type of intervention, type of review, design and methods used, outcome measures, quality assessment, study results and findings (Appendix 5). We have only extracted information at the systematic review level.

For peer review only - http://bmjopen.bmj.com/site/about/guidelines.xhtml

Non-independence of reviews (or overlap)

Non-independence of reviews (or overlap) is an issue unique to systematic reviews of systematic reviews. Overlap explores to what extent the primary studies included in the pool of systematic reviews are the same or different. We assessed overlap by compiling a citation matrix which includes all the primary studies (one per row) included in the individual systematic reviews (one per column). Primary studies were sorted alphabetically, and duplicates removed. The primary studies that were the same across reviews were ticked with a check mark. This allowed us to calculate the corrected covered area (CCA) index that describes the extent of overlap in % terms.[16, 17]

$$CCA = \frac{N - r}{(rxc) - r}$$

Where N is the total number of primary studies included in the systematic reviews (the sum of ticked boxes in the citation matrix), r is the number of rows (the primary studies), and c is the number of columns (included systematic reviews). Pieper et al.[17] provide criteria for the interpretation of CCA, where 0-5% suggests slight overlap, 6-10% moderate overlap, 11-15% high overlap and >15% very high overlap. Hennessy and Johnson[16] recommend further overlap investigations in case of a heterogenous evidence base, hence we compare sub-clusters of reviews examining similar outcomes as well.

Assessment of risk of bias in included studies

The quality of systematic reviews is assessed differently compared to assessing the quality of the primary studies included in systematic reviews, this is due to the unique methodological features of systematic reviews. The quality of the included reviews was assessed independently by two researchers using the checklist developed by the Specialist Unit for Review Evidence (SURE) to allow a critical appraisal to ensure that minimum levels of methodological rigour are met. The SURE[18] checklist was slightly adapted (Appendix 6).

For peer review only - http://bmjopen.bmj.com/site/about/guidelines.xhtml

We also extracted information on the quality assessment tools reported in each included review which have been used to assess the quality of the underlying primary evidence base by the reviews. Nevertheless, risk of bias of primary or supplementary primary studies was not assessed.

Data synthesis

We adopted a mixed-methods synthesis approach to best accommodate both the quantitative and qualitative information available. To synthesise the quantitative data, we implemented a robust variance estimation (RVE) meta-regression approach[19] as this performs well even when study numbers are as low as 10.[20]

When an RVE meta-regression approach was not possible, we fitted a random-effects model to the data. In this case, the amount of heterogeneity (i.e., τ^2), was estimated using the DerSimonian-Laird estimator.[21] In addition, we also reported the Q-test for heterogeneity[22] and the I^2 statistic.[23] Studentized residuals and Cook's distances were used to examine whether studies may be outliers and/or influential in the context of the model.[24] Studies with a studentized residual larger than the $100\times(1-0.05/(2\times k))^{th}$ percentile of a standard normal distribution were considered potential outliers (i.e., using a Bonferroni correction with two-sided $\alpha = 0.05$ for k studies included in the meta-analysis). Studies with a Cook's distance larger than the median plus six times the interquartile range of the Cook's distances were considered to be influential. The rank correlation test[25] and the regression test,[26] using the standard error of the observed outcomes as predictor, were used to check for funnel plot asymmetry but only when we had more than 10 studies.[27]

Risk ratios (RRs) were the most commonly reported effect size, and thus our metric of choice. Odds ratios (ORs) were converted to RRs using the following formula:[28]

$$RR = OR / (1 – p + (p \times OR))$$

For peer review only - http://bmjopen.bmj.com/site/about/guidelines.xhtml

Where p is the risk in the control group implying that ORs can only be converted to RRs when the risk in the control group is known. As this is not always the case, we established a range of plausible risks for the control group (Grant, 2014) drawing on data from Jain et al.[29, 30] on baseline full immunisation rates to find the mean (0·48) and standard deviation (SD) (0·22) of the data to be able to convert ORs to RRs. We did these conversions 3 times as a sensitivity analysis. First, we used 1 SD below the mean to establish the control group rate (0.26 – the least conservative model), second, we used the mean as the control group rate (0·48 – the baseline model), and finally, we used 1 SD above the mean (0·70 – the conservative model) as the control group rate.

Patient and public involvement

No patients have been involved in this study as it is a review article, hence no patient consent for publication was required.

Role of the funding source

The funder of the study had no role in the study design, data collection, data analysis, data interpretation, or writing of the report.

RESULTS

Our search builds on Engelbert et al.[15] who had included 58 systematic reviews in their EGM, which we screened against our inclusion criteria. Six of these reviews did not meet our criteria for inclusion and were thus excluded leaving us with 52 studies. We ran additional searches (Appendix 4); the electronic search led to 1,687 records of which 818 were duplicates leaving 869 for screening. We excluded 823 records based on title and abstract screening. The remaining 46 studies were screened by full-text, 39 were excluded, leaving seven new studies for inclusion derived from the electronic search. We identified a further seven records searching the grey literature which all required full-text screening. Four were subsequently

For peer review only - http://bmjopen.bmj.com/site/about/guidelines.xhtml

excluded leaving three new studies for inclusion (Figure 1). This two-pronged search strategy led to 10 new systematic reviews to be included. Overall, we included a total of 62 systematic reviews - 52 studies from the EGM by Engelbert et al.[15] and 10 new studies from the updated search A summary of the included systematic reviews can be found in Appendix 7.

< Place Figure 1 here >

Overlap

We test overlap by compiling a citation matrix listing all primary studies which is used to calculate the CCA. Table 1 suggests limited overlap of 0.5% confirming our initial investigations. This is confirmed by Pieper et al.[17] who provide guidance on the interpretation of CCA values, e.g., with values between 0-5% indicating slight overlap.

Table 1: Overlap

	Times studies appeared in reviews	Number of rows	Number of reviews	CCA values	
	N	*r*	*c*	Proportion	Percentage
Overall	1428	1079	62	0·0053	0·5

We compare sub-clusters of reviews examining similar outcomes as well finding limited overlap for most outcomes, e.g., for measles, full routine immunisation for children, DPT3 and vaccination coverage, the CCA values range from 0·7 to 2·7%. Vaccination timeliness has the highest CCA value (6·9%) indicating moderate levels of overlap.[17]

For peer review only - http://bmjopen.bmj.com/site/about/guidelines.xhtml

Risk of Bias

Using the SURE checklist, we find that of the 62 included reviews, 18 are categorised as high confidence, six are of medium confidence and 38 are of low confidence (Appendix 8).

For peer review only

For peer review only - http://bmjopen.bmj.com/site/about/guidelines.xhtml

Effectiveness of interventions

Table 2: Summary of findings

Intervention (1st, 2nd, 3rd tier)			# SRs	SRs (Low confidence SRs in parenthesis)	Overall findings**
1st tier	2nd tier	3rd tier			
A. Caregiver-oriented interventions	AA. Information & education	AA1. Sustained sensitisation and education campaigns	1	([31])	None (Low confidence evidence)
		AA2. Short-term sensitisation and education campaigns	12	[6, 8, 9, 11, 12, 32] ([33-38])	Positive and statistically significant
		AA3. Public information campaigns	3	([38-40])	None (Low confidence evidence)
	AB. Incentives & motivation	AB1. Material/ monetary incentives for caregivers	12	[11, 41, 42] ([33, 35, 43-49])	Mixed effects
		AB3. Automated voice messages to caregivers	1	([50])	None (Low confidence evidence)
		AB4. Written or pictorial messages (SMS, stickers, flyers etc.) to caregivers	13	[5, 10, 51-54] ([33, 35, 36, 45, 50, 55, 56])	Positive and statistically significant
		AB5. Changes to health system user fees	1	([57])	None (Low confidence evidence)
B. Health system-oriented	BA. Education & training	BA1. Formal health worker training and education	3	[11] ([35, 48])	Limited evidence
	BB. Planning, implementation, monitoring	BB5. Outreach to vulnerable populations (hard-to-reach, etc.)	3	[11] ([33, 40])	Limited evidence
		BB7. Home visits	5	[11, 58] ([38, 59, 60])	Positive

For peer review only - http://bmjopen.bmj.com/site/about/guidelines.xhtml

Intervention (1st, 2nd, 3rd tier)			# SRs	SRs (Low confidence SRs in parenthesis)	Overall findings**
1st tier	2nd tier	3rd tier			
	BC. Supplementary Immunisation Activities	BC1. National/sub-national immunisation days	2	([34, 59])	None (Low confidence evidence)
	BD. Incentives & motivation	BD1. Material/ monetary incentives for health workers	1	([38])	None (Low confidence evidence)
		BD4. Written or pictorial messages to health workers	1	([43])	None (Low confidence evidence)
		BD5. Pay-for-performance schemes	5	[7, 61] ([43, 62, 63])	Mixed effects
	BF. Health system governance, policies and financing	BF1. Health system strategic planning	7	[11] ([40, 43, 45, 64-66])	Limited evidence
		BF4. Health system financing	3	[67, 68] ([69])	Inconclusive
	BG. Technology & mHealth	BG1. New HMIS/Dashboard systems	3	([36, 70, 71])	None (Low confidence evidence)
C. Other community member-oriented	CA. Other community member-oriented	CA1. Faith-based outreach/outreach using local leaders	1	([35])	None (Low confidence evidence)
D. Community-level	DA. Communication & dialogue	DA1. Collaborating with whole community	1	[72]	Limited evidence
		DA2. Collaborating with selected community groups and networks	1	[73]	Limited evidence
F. Multicomponent			1*	[29, 30]	Positive and statistically significant

Notes: *Apart from Jain et al.[29, 30], there were 25 systematic reviews that assessed the effects of more than one intervention that are not part of this table. Of these reviews, the majority (18) were low confidence SRs and the intervention components were heterogenous. We provide summary of Jain et al.[29, 30] in

For peer review only - http://bmjopen.bmj.com/site/about/guidelines.xhtml

this table because it is one of the only included SRs that assessed community engagement interventions. Intervention and outcome codes are taken from Appendix 1.
** The last column, 'overall findings' are summarized from evidence as reported in the systematic reviews, qualitative and quantitative findings and quantitative findings from review of reviews (RoR). Kindly refer to Supplementary file – Appendix 9 for the expanded version of this table.

For peer review only

< Place Figure 2 here >

<Place Figure 3 here >

Overall, the evidence base is highly heterogenous in terms of scope, intervention types and outcomes. Table 2 (and Supplementary file – Appendix 9) summarises the key findings across all high and medium confidence reviews across all intervention categories. Most of the evidence we uncovered is centred on caregiver-oriented interventions (A) that have positive and statistically significant effects at the 1st and 2nd tier analysis level (Appendices 10 and 11 for findings with details on intervention coding and level of analysis in Table 2 – Appendix 9). At the 3rd tier level, our analysis confirms the favourable trends emerging from the 1st and 2nd tier analyses. For short-term sensitisation and education campaigns (AA2 – Figure 2), we find that the average RR was 1·38 (95% CI: 1·33 to 1·44) suggesting that the average outcome differed significantly from zero ($z = 47{\cdot}37$, $p < 0{\cdot}001$). As for written or pictorial messages (SMS, stickers, flyers etc.) to caregivers (AB4 – Figure 3), we find that the average RR of the baseline model stands at 1.24 ([95% CI: 1·11 to 1·36], $p < 0{\cdot}001$). Overall, 3rd tier intervention categories are reporting positive and statistically significant effects, which suggest that treated children are more likely to be vaccinated than untreated children. Another intervention category at the 3rd tier for which there is a large evidence base is monetary incentives (AB1)[1] and the evidence is mixed.

For the health systems-oriented interventions (B) we had no quantitative evidence, thus we could only rely on narrative synthesis and the results are mixed (Appendix 12). Depending on the exact nature of the intervention, the contextual background and the specific outcome, the findings range from favourable to inconclusive effects. Among these interventions, the only

[1] The evidence on monetary incentives (AB1) interventions is dominated by qualitative narrative methods. There are only two reviews with quantitative evidence, but we could not use them for quantitative synthesis as we were not able to convert effect sizes from SMD to RR.

For peer review only - http://bmjopen.bmj.com/site/about/guidelines.xhtml

intervention category at the 3rd tier for which we find positive effects are home visits (BB7). We find mixed results for pay for performance schemes (BD5) which have a large evidence base both at the primary study and review level. Additionally, we find inconclusive results based on a thin evidence base for health systems financing (BF4) interventions in which health services are contracted out to non-governmental providers and they are compensated for it. For a 3rd tier category like health system strategic planning (BF1), though the evidence base is large when it occurs in combination with other interventions, i.e., as multicomponent interventions, the evidence base for single interventions is rather limited and inconclusive.

As for community-oriented interventions (C and D), the evidence base is very limited, focused on narrative synthesis and finding inconclusive results (Appendix 13). However, a recent review[29, 30] on single and multicomponent community engagement interventions (F) uses a nuanced framework to classify them based on the process of engaging communities and finds them to be effective using meta-analytical methods. We found no evidence on interventions related to non-health related policies and institutions (E).

This review also assessed the interventions aiming to improve zero dose outcomes through DPT1 and BCG vaccine uptake. We find 4 reviews suggesting inconclusive evidence for interventions improving DPT1 outcomes. However, for BCG vaccine uptake we find pay for performance schemes (BD5 – 1 qualitative review) and community engagement interventions (C and D – 1 quantitative review) to be effective.

DISCUSSION

Through this review we have demonstrated that a wealth of systematic reviews on interventions impacting routine child immunisation outcomes exist, but many are very broad in their scope and hence, may not provide a clear answer on all the different types of interventions that may or may not work best for improving immunisation outcomes of children in L&MICs.

For peer review only - http://bmjopen.bmj.com/site/about/guidelines.xhtml

For caregiver-oriented interventions (A) the evidence base is of reasonable quality and sufficiently large compared to that on health-systems (B) and community-oriented (C and D) interventions. We also find that caregiver-oriented interventions, like those focusing on short term sensitisation and education campaigns (AA2) as well as written messages to caregivers (AB4), are effective with most findings suggesting positive and statistically significant results. On the other hand, for health-systems oriented interventions the evidence base is thin with narrative synthesis approaches dominating and suggesting inconclusive results. For community-oriented interventions the evidence base is limited, but a recent review [29, 30] provides high quality evidence on the effectiveness of community engagement interventions with mostly positive and statistically significant results. Overall, the evidence base provides clearer answers on the effectiveness of caregiver-oriented interventions and to a certain degree of community-oriented interventions than of health-systems oriented interventions.

Strengths and weaknesses

We have contributed to the literature by synthesising the evidence base on routine child immunisation interventions in L&MICs at a higher level of abstraction (i.e., at the systematic review of systematic reviews level) and this brings about a number of methodological challenges. For example, the validity of this review depends on the coverage and quality of the underlying evidence base and thus using the SURE checklist we drew attention to the mixed quality of the primary evidence base that informed the findings of our included systematic reviews. Consequently, caution must be exercised in the interpretation of the systematic review evidence, especially as no mitigating actions were taken to deal with potential biases. One of the ways to ensure robustness of evidence is to provide subgroup analysis that disaggregates by levels of confidence. However, the majority of the included systematic reviews did not

For peer review only - http://bmjopen.bmj.com/site/about/guidelines.xhtml

provide subgroup analysis because their analyses were mostly based on small study samples that were not sufficient for credible subgroup analyses. In addition, we were faced with high levels of heterogeneity and a wide range of synthesis approaches limiting our pool of studies for meta-analysis. We addressed these limitations to a certain extent by also considering qualitative evidence. Generally, we have dealt with these methodological challenges as much as possible following guidance provided by the Cochrane and Campbell Collaborations. Finally, most of the included studies did not pay sufficient attention to unpacking causal mechanisms which limited our ability to firmly conclude how and for whom routine child immunization interventions are working.

Implications for policy and practice

Implications for caregiver-oriented interventions: The caregiver-oriented interventions are effective. Sensitisation and education campaigns (AA2) mostly address the knowledge gaps of caregivers on the importance of vaccinations, importance of maintaining a schedule, potential misconceptions around vaccinations. This information is delivered by either frontline health workers or trained facilitators at the health facility, at caregiver homes or in community groups. The SMS reminders (AB4) delivered to the mobile phones of caregivers address the practical barriers that they face regarding when and where to take their child for a vaccine or follow-up doses. Thus, these interventions can be effective in improving vaccine uptake in communities where such barriers are prevalent.

In the last decade, a lot of the attention of researchers, programme implementers and policymakers has been on how to motivate caregivers to vaccinate their children through monetary incentives and there have been a substantial number of experiments involving conditional and/or unconditional cash transfer schemes to assess their effectiveness in improving immunisation outcomes.[15] However, the evidence does not provide a clear answer

For peer review only - http://bmjopen.bmj.com/site/about/guidelines.xhtml

on their effectiveness and there is a need for an updated review with a more inclusive search strategy for identifying relevant articles published in non-health journals.

Implications for health-system oriented interventions: Among the health-system oriented interventions, home visits (BB7) are worth considering for improvement in vaccine uptake as we find them to be effective. For pay-for-performance schemes (BD5) schemes we find mixed evidence. As a recent review[7] of this intervention category is available and of high quality, there is no need for an update and policymakers can consult it for more details.

Two intervention categories which are of specific policy relevance and would benefit from a stronger evidence base are: a) Interventions focusing on building skills, capacity and motivation of formal health workers (BA1, BA2, BD1, BD2) and b) new HMIS/dashboards systems (BG1), i.e. interventions leveraging digital technologies, which are increasingly being adopted and expanded across L&MICs. For both these intervention categories, Engelbert et al.[15] found a substantial number of primary studies, though most were not single but multicomponent interventions.

While the evidence on health-system oriented interventions in the context of immunisation is limited, policymakers seeking to strengthen health systems for the purpose of improving immunisation outcomes can consult the broader literature on health system strengthening for guidance. In fact, there is a systematic review of reviews available,[74] which synthesised evidence that assessed the effects of health systems strengthening interventions on health status and health system outcomes (service utilisation, quality service provision, uptake of healthy behaviours, and financial protection) in L&MICs, which may be helpful depending on its quality.

Implications for community-oriented interventions: For guidance on effectiveness of community-oriented interventions, the recent review by Jain et al.[29, 30] can be consulted as

For peer review only - http://bmjopen.bmj.com/site/about/guidelines.xhtml

it is comprehensive, provides quantitative evidence and uses a nuanced framework to classify community-oriented interventions based on the process of engaging communities.

Implications for research

Besides addressing critical evidence gaps as described in the section above, we also need to better understand complementarity of interventions, e.g., some reviews analysed combinations of caregiver and health-systems oriented intervention types (bridging the demand and supply side gaps) finding favourable but also inconclusive effects. We do not yet know which intervention combinations work best in terms of improving immunisation outcomes. Further theory development may be a starting point for contributing to a better understanding of the enablers and barriers of interventions as well as unpacking underlying causal mechanisms and thus improving the selection and targeting of immunisation programmes.

In addition, future research needs to engage more with cost effectiveness of interventions as we need to not only learn about what works but also at what cost to enable selection of not only the most impactful but also most cost-effective interventions to improve routine child immunisation outcomes. Further work is also needed in improving the quality of both systematic reviews and primary studies. At the review level future research should attempt more subgroup analyses by levels of confidence in primary studies to instil more trust in the results. At the primary study level, researchers should use more robust evaluation methods to minimise the potential biases in the measurement of effects. More work is required to develop user friendly quality assessment tools for systematic reviewers that minimise the scope for subjective judgements.

For peer review only - http://bmjopen.bmj.com/site/about/guidelines.xhtml

DECLARATION

Registration and protocol

This umbrella review was not registered. Although we prepared a detailed protocol, it was not published. There were no amendments made.

Author contributors

Monica Jain (MJ) conceived the review and along with Shannon Shisler (SS) and Maren Duvendack (MD) wrote the protocol. Shradha S. Parsekar (SP) and Maria Daniela Anda Leon (DA) did the systematic search. SP and DA screened and identified studies and MJ and MD made final decisions regarding study inclusion. SP and DA did the data extraction and critical appraisal. SS, DA and MD did the statistical analysis. MD and SP did the qualitative analysis. MD and MJ wrote the manuscript. MJ provided critical inputs on the whole analysis, checked data, coordinated the review and had full access to all materials and results. All authors critically reviewed and revised the manuscript and approved the final document for submission. MJ is responsible for the overall content and is the guarantor. All authors attest they meet the ICMJE criteria for authorship. MJ is identified as the guarantor of this review.

Declaration of interests

This research was funded through a grant from the Bill & Melinda Gates Foundation (INV-008461) to the International Initiative for Impact Evaluation (3ie). Through this grant the following work has been commissioned: a) 3ie provided funding and technical assistance for seven impact evaluations of community engagement interventions for immunisation, b) 3ie conducted a systematic review of community engagement interventions to assess their effectiveness in improving routine child immunisation in L&MICs; and c) 3ie conducted an evidence gap-map on routine child immunisation in L&MICs. As members of 3ie staff, author MJ had been involved in reviewing deliverables for the evaluations funded by 3ie and

For peer review only

For peer review only - http://bmjopen.bmj.com/site/about/guidelines.xhtml

providing research teams with technical assistance. MJ, SS, DA & SP have also worked as authors on the systematic review [29, 30] and MJ and SP have been co-authors of the EGM report [15]. Among these, only the work on the systematic review could pose a potential conflict of interest. However, several procedural safeguards and transparency measures were put in place to mitigate the risk this conflict of interest imposed. All candidate systematic reviews, including the one carried out by 3ie, have undergone a rigorous screening and critical appraisal process by two independent screeners. The authors have no financial interest in this area. The remaining study author MD has authored systematic reviews and systematic reviews of reviews but largely on issues related to financial inclusion. MD is also an editor of the International Development Coordinating Group at the Campbell Collaboration, but this will not pose any conflicts of interest.

Role of the funding source

The funder of the study had no role in study design, data collection, data analysis, data interpretation, or writing of the report.

Data sharing

The systematic reviews and other documents included in this study are public and available. The data extracted from these studies are available from the corresponding author upon request.

Acknowledgements

This research has been undertaken as a part of 3ie's immunisation evidence programme, supported by the Bill and Melinda Gates Foundation, Seattle, USA. We would like to thank Sohail Agha, previous Senior Program Officer, Gates Foundation, Seattle, for his support in conceptualisation as well as engagement on the review. Special thanks to Tove Ryman, Senior Program Officer, Gates Foundation, Seattle, whose guidance helped shape the analysis and

For peer review only - http://bmjopen.bmj.com/site/about/guidelines.xhtml

dissemination of this review. We would like to further thank Mark Engelbert who was instrumental in guiding the search process and Avantika Bagai who provided support in the initial phase of the review. We would also like to thank Birte Sniltsveit at 3ie for her comments on this review and for Sebastian Martinez at 3ie for his overall support of the review.

Ethical statements:

Patient consent for publication: Not applicable.

Ethical approval: The ethical approval was not required for this umbrella review as all the data were generated from published systematic reviews.

REFERENCES

1. WHO. Progress and Challenges with Achieving Universal Immunization Coverage: 2019 WHO/UNICEF Estimates of National Immunization Coverage Geneva: World Health Organization; 2019 [Available from: https://www.who.int/publications/m/item/progress-and-challenges-with-achievinguniversal-immunization-coverage.

2. WHO. Immunization coverage: Key facts Geneva: World Health Organization; 2021 [Available from: https://www.who.int/news-room/fact-sheets/detail/immunization-coverage.

3. WHO. Immunization Coverage Geneva: World Health Organization; 2018 [Available from: https://www.who.int/news-room/fact-sheets/detail/immunization-coverage.

4. Harvey H, Reissland N, Mason J. Parental reminder, recall and educational interventions to improve early childhood immunisation uptake: A systematic review and meta-analysis. Vaccine. 2015;33(25):2862-80.

5. Atkinson KM, Wilson K, Murphy MSQ, El-Halabi S, Kahale LA, Laflamme LL, et al. Effectiveness of digital technologies at improving vaccine uptake and series completion - A systematic review and meta-analysis of randomized controlled trials. Vaccine. 2019;37(23):3050-60.

6. Lukusa LA, Ndze VN, Mbeye NM, Wiysonge CS. A systematic review and meta-analysis of the effects of educating parents on the benefits and schedules of childhood vaccinations in low and middle-income countries. Human vaccines & immunotherapeutics. 2018;14(8):2058-68.

For peer review only - http://bmjopen.bmj.com/site/about/guidelines.xhtml

7. Diaconu K, Falconer J, Verbel A, Fretheim A, Witter S. Paying for performance to improve the delivery of health interventions in low- and middle-income countries. The Cochrane database of systematic reviews. 2021;5(5):Cd007899.

8. Johri M, Pérez MC, Arsenault C, Sharma JK, Pai NP, Pahwa S, et al. Strategies to increase the demand for childhood vaccination in low- and middle-income countries: a systematic review and meta-analysis. Bulletin of the World Health Organization. 2015;93(5):339-46c.

9. Kaufman J, Ryan R, Walsh L, Horey D, Leask J, Robinson P, et al. Face-to-face interventions for informing or educating parents about early childhood vaccination. The Cochrane database of systematic reviews. 2018;5(5):Cd010038.

10. Mekonnen ZA, Gelaye KA, Were MC, Gashu KD, Tilahun BC. Effect of mobile text message reminders on routine childhood vaccination: a systematic review and meta-analysis. Systematic reviews. 2019;8(1):154.

11. Oyo-Ita A, Wiysonge CS, Oringanje C, Nwachukwu CE, Oduwole O, Meremikwu MM. Interventions for improving coverage of childhood immunisation in low- and middle-income countries. The Cochrane database of systematic reviews. 2016;7(7):Cd008145.

12. Saeterdal I, Lewin S, Austvoll-Dahlgren A, Glenton C, Munabi-Babigumira S. Interventions aimed at communities to inform and/or educate about early childhood vaccination. The Cochrane database of systematic reviews. 2014(11):Cd010232.

13. Heneghan C, Plüddemann A, Spencer E, Brassey J, Rosca E, Onakpoya I, et al. Interventions designed to improve vaccination uptake: Scoping review of systematic reviews and meta-analyses - (version 1). medRxiv. 2022:01.26.22269890.

14. Besnier E, Thomson K, Stonkute D, Mohammad T, Akhter N, Todd A, et al. Which public health interventions are effective in reducing morbidity, mortality and health inequalities from infectious diseases amongst children in low- and middle-income countries (LMICs): An umbrella review. PloS one. 2021;16(6):e0251905.

15. Engelbert M, Jain M, Bagai A, Parsekar SS. Improving routine childhood immunisation outcomes in low-income and middle-income countries: an evidence gap map. BMJ open. 2022;12(11):e058258.

16. Hennessy EA, Johnson BT. Examining overlap of included studies in meta-reviews: Guidance for using the corrected covered area index. Research synthesis methods. 2020;11(1):134-45.

17. Pieper D, Antoine SL, Mathes T, Neugebauer EA, Eikermann M. Systematic review finds overlapping reviews were not mentioned in every other overview. Journal of clinical epidemiology. 2014;67(4):368-75.

For peer review only - http://bmjopen.bmj.com/site/about/guidelines.xhtml

18. SURE. Questions to assist with the critical appraisal of systematic reviews. The Specialist Unit for Review Evidence (SURE); 2018.

19. Pustejovsky JE, Tipton E. Meta-analysis with Robust Variance Estimation: Expanding the Range of Working Models. Prevention Science. 2022;23(3):425-38.

20. Tipton E. Robust variance estimation in meta-regression with binary dependent effects. Research synthesis methods. 2013;4(2):169-87.

21. DerSimonian R, Laird N. Meta-analysis in clinical trials. Controlled clinical trials. 1986;7(3):177-88.

22. Cochran WG. The Combination of Estimates from Different Experiments. Biometrics. 1954;10(1):101-29.

23. Higgins JP, Thompson SG. Quantifying heterogeneity in a meta-analysis. Statistics in medicine. 2002;21(11):1539-58.

24. Viechtbauer W, Cheung MW. Outlier and influence diagnostics for meta-analysis. Research synthesis methods. 2010;1(2):112-25.

25. Begg CB, Mazumdar M. Operating characteristics of a rank correlation test for publication bias. Biometrics. 1994;50(4):1088-101.

26. Sterne JAC, Egger M. Regression methods to detect publication and other bias in meta-analysis. In: H. R. Rothstein AJS, & M. Borenstein editor. Publication bias in meta-analysis: Prevention, assessment and adjustment Chichester: Wiley; 2005. p. 99-110.

27. Viechtbauer W. Conducting Meta-Analyses in R with the metafor Package. Journal of Statistical Software. 2010;36(3):1 - 48.

28. Grant RL. Converting an odds ratio to a range of plausible relative risks for better communication of research findings. BMJ : British Medical Journal. 2014;348:f7450.

29. Jain M, Shisler S, Lane C, Bagai A, Brown E, Engelbert M. Use of community engagement interventions to improve child immunisation in low-income and middle-income countries: a systematic review and meta-analysis. BMJ open. 2022;12(11):e061568.

30. Jain M, Shisler S, Lane C, Bagai A, Brown E, Engelbert M, et al. Use of community engagement interventions to improve child immunisation in low- and middle-income countries: A systematic review and meta-analysis. Campbell Systematic Reviews. 2022;18(3):e1253.

31. Ryman TK, Dietz V, Cairns KL. Too little but not too late: results of a literature review to improve routine immunization programs in developing countries. BMC Health Serv Res. 2008;8:134.

For peer review only

32. Gilmore B, McAuliffe E. Effectiveness of community health workers delivering preventive interventions for maternal and child health in low- and middle-income countries: a systematic review. BMC public health. 2013;13:847.

33. Crocker-Buque T, Mindra G, Duncan R, Mounier-Jack S. Immunization, urbanization and slums - a systematic review of factors and interventions. BMC public health. 2017;17(1):556.

34. Deardorff KV, Rubin Means A, Ásbjörnsdóttir KH, Walson J. Strategies to improve treatment coverage in community-based public health programs: A systematic review of the literature. PLoS neglected tropical diseases. 2018;12(2):e0006211.

35. Jarrett C, Wilson R, O'Leary M, Eckersberger E, Larson HJ. Strategies for addressing vaccine hesitancy - A systematic review. Vaccine. 2015;33(34):4180-90.

36. Kim SS, Patel M, Hinman A. Use of m-Health in polio eradication and other immunization activities in developing countries. Vaccine. 2017;35(10):1373-9.

37. Pinzón Flórez C, Díaz-Quijano D, Yáñez Álvarez I, Catalina Mesa D. Effectiveness of community workers on preventive measures to maternal and child health in low and middle income countries: systematic review of the literature. Revista Salud Uninorte. 2015;31(2):309-28.

38. Shea B, Andersson N, Henry D. Increasing the demand for childhood vaccination in developing countries: a systematic review. BMC international health and human rights. 2009;9 Suppl 1(Suppl 1):S5.

39. Naugle DA, Hornik RC. Systematic review of the effectiveness of mass media interventions for child survival in low- and middle-income countries. Journal of health communication. 2014;19 Suppl 1(sup1):190-215.

40. Yuan B, Målqvist M, Trygg N, Qian X, Ng N, Thomsen S. What interventions are effective on reducing inequalities in maternal and child health in low- and middle-income settings? A systematic review. BMC public health. 2014;14:634.

41. Lagarde M, Haines A, Palmer N. The impact of conditional cash transfers on health outcomes and use of health services in low and middle income countries. The Cochrane database of systematic reviews. 2009;2009(4):Cd008137.

42. Bassani DG, Arora P, Wazny K, Gaffey MF, Lenters L, Bhutta ZA. Financial incentives and coverage of child health interventions: a systematic review and meta-analysis. BMC public health. 2013;13 Suppl 3(Suppl 3):S30.

For peer review only

For peer review only - http://bmjopen.bmj.com/site/about/guidelines.xhtml

43. Bright T, Felix L, Kuper H, Polack S. A systematic review of strategies to increase access to health services among children in low and middle income countries. BMC Health Services Research. 2017;17(1):252.

44. de Souza Cruz RC, Moura LBA, Soares Neto JJ. Conditional cash transfers and the creation of equal opportunities of health for children in low and middle-income countries: a literature review. International journal for equity in health. 2017;16(1):161.

45. Omoniyi OS, Williams I. Realist Synthesis of the International Theory and Evidence on Strategies to Improve Childhood Vaccination in Low- and Middle-Income Countries: Developing Strategies for the Nigerian Healthcare System. International journal of health policy and management. 2020;9(7):274-85.

46. Onwuchekwa C, Verdonck K, Marchal B. Systematic Review on the Impact of Conditional Cash Transfers on Child Health Service Utilisation and Child Health in Sub-Saharan Africa. Frontiers in public health. 2021;9:643621.

47. Owusu-Addo E, Cross R. The impact of conditional cash transfers on child health in low- and middle-income countries: a systematic review. International journal of public health. 2014;59(4):609-18.

48. Pal M, Goodyear-Smith F, Exeter D. Systematic review of pertussis immunisation among Asians. International Journal of Human Rights in Healthcare. 2016;9(2):135-46.

49. Ranganathan M, Lagarde M. Promoting healthy behaviours and improving health outcomes in low and middle income countries: a review of the impact of conditional cash transfer programmes. Preventive medicine. 2012;55 Suppl:S95-s105.

50. Yunusa U, Garba SN, Umar AB, Idris SH, Bello UL, Abdulrashid I, et al. Mobile phone reminders for enhancing uptake, completeness and timeliness of routine childhood immunization in low and middle income countries: A systematic review and meta-analysis. Vaccine. 2021;39(2):209-21.

51. Chutiyami M, Wyver S, Amin J. Are Parent-Held Child Health Records a Valuable Health Intervention? A Systematic Review and Meta-Analysis. International journal of environmental research and public health. 2019;16(2).

52. Eze P, Lawani LO, Acharya Y. Short message service (SMS) reminders for childhood immunisation in low-income and middle-income countries: a systematic review and meta-analysis. BMJ Global Health. 2021;6(7):e005035.

53. Linde DS, Korsholm M, Katanga J, Rasch V, Lundh A, Andersen MS. One-way SMS and healthcare outcomes in Africa: Systematic review of randomised trials with meta-analysis. PloS one. 2019;14(6):e0217485.

For peer review only

For peer review only - http://bmjopen.bmj.com/site/about/guidelines.xhtml

54. Palmer MJ, Henschke N, Bergman H, Villanueva G, Maayan N, Tamrat T, et al. Targeted client communication via mobile devices for improving maternal, neonatal, and child health. The Cochrane database of systematic reviews. 2020;8(8):Cd013679.

55. Baequni NY. Is Maternal and Child Health Handbook effective? : Meta-Analysis of the Effects of MCH Handbook. Kokusai Hoken Iryo (Journal of International Health). 2012;27(2):121-7.

56. Karageorgos G, Andreadis I, Psychas K, Mourkousis G, Kiourti A, Lazzi G, et al. The Promise of Mobile Technologies for the Health Care System in the Developing World: A Systematic Review. IEEE reviews in biomedical engineering. 2019;12:100-22.

57. Giedion U, Andrés Alfonso E, Díaz Y. The impact of universal coverage schemes in the developing world: a review of the existing evidence. Washington DC: World Bank; 2013. Contract No.: 25.

58. Glenton C, Scheel IB, Lewin S, Swingler GH. Can lay health workers increase the uptake of childhood immunisation? Systematic review and typology. Tropical medicine & international health : TM & IH. 2011;16(9):1044-53.

59. Freeman PA, Schleiff M, Sacks E, Rassekh BM, Gupta S, Perry HB. Comprehensive review of the evidence regarding the effectiveness of community-based primary health care in improving maternal, neonatal and child health: 4. child health findings. Journal of global health. 2017;7(1):010904.

60. Rahman A, Fisher J, Bower P, Luchters S, Tran T, Yasamy MT, et al. Interventions for common perinatal mental disorders in women in low- and middle-income countries: a systematic review and meta-analysis. Bulletin of the World Health Organization. 2013;91(8):593-601i.

61. Witter S, Fretheim A, Kessy FL, Lindahl AK. Paying for performance to improve the delivery of health interventions in low- and middle-income countries The Cochrane database of systematic reviews. 2012(2):Cd007899.

62. Das A, Gopalan SS, Chandramohan D. Effect of pay for performance to improve quality of maternal and child care in low- and middle-income countries: a systematic review. BMC public health. 2016;16:321.

63. Parrado Jiménez D. Revisión sistemática de la literatura del efecto de pago por desempeño en la calidad de la atención en países de bajos y medianos ingresos. Bogota Colombia Universidad de Los Andes; 2013.

64. Nelson KN, Wallace AS, Sodha SV, Daniels D, Dietz V. Assessing strategies for increasing urban routine immunization coverage of childhood vaccines in low and middle-

For peer review only - http://bmjopen.bmj.com/site/about/guidelines.xhtml

income countries: A systematic review of peer-reviewed literature. Vaccine. 2016;34(46):5495-503.

65. Nutman S, McKee D, Khoshnood K. Externalities of prevention of mother-to-child transmission programs: a systematic review. AIDS and behavior. 2013;17(2):445-60.

66. Smith BL, Zizzo S, Amzel A, Wiant S, Pezzulo MC, Konopka S, et al. Integration of Neonatal and Child Health Interventions with Pediatric HIV Interventions in Global Health. International journal of MCH and AIDS. 2018;7(1):192-206.

67. Lagarde M, Palmer N. The impact of contracting out on health outcomes and use of health services in low and middle-income countries. The Cochrane database of systematic reviews. 2009(4):Cd008133.

68. Odendaal WA, Ward K, Uneke J, Uro-Chukwu H, Chitama D, Balakrishna Y, et al. Contracting out to improve the use of clinical health services and health outcomes in low- and middle-income countries. The Cochrane database of systematic reviews. 2018;4(4):Cd008133.

69. Loevinsohn B, & , Harding A. Contracting for the delivery of community health services: A review of Global Experience. Washington, DC: World Bank; 2004.

70. de Cock C, van Velthoven M, Milne-Ives M, Mooney M, Meinert E. Use of Apps to Promote Childhood Vaccination: Systematic Review. JMIR mHealth and uHealth. 2020;8(5):e17371.

71. Watterson JL, Walsh J, Madeka I. Using mHealth to Improve Usage of Antenatal Care, Postnatal Care, and Immunization: A Systematic Review of the Literature. BioMed research international. 2015;2015:153402.

72. Molina E, Carella L, Pacheco A, Cruces G, Gasparini L. Community monitoring interventions to curb corruption and increase access and quality of service delivery in low- and middle-income countries: a systematic review. Campbell Systematic Reviews. 2016;12(1):1-204.

73. Desai S, Misra M, Das A, Singh RJ, Sehgal M, Gram L, et al. Community interventions with women's groups to improve women's and children's health in India: a mixed-methods systematic review of effects, enablers and barriers. BMJ Glob Health. 2020;5(12).

74. Hatt L, Johns C, Connor C, Meline M, Kukla M, & , Moat K. Impact of Health Systems Strengthening on Health. Bethesda, MD: Health Finance & Governance Project, Abt Associates; 2015.

Supplementary material

Supplementary material includes:

For peer review only

Appendix 1: Intervention-outcome framework

Appendix 2: Detailed inclusion and exclusion criteria

Appendix 3: List of excluded studies – after full-text screening

Appendix 4: List of sources and search strings

Appendix 5: Data extraction template

Appendix 6: Quality appraisal tool

Appendix 7: Summary of included systematic reviews

Appendix 8: Risk of bias of included systematic reviews

Appendix 9: Summary of main findings

Appendix 10: RVE analysis – 1st tier synthesis

Appendix 11: RVE analysis – 2nd tier synthesis

Appendix 12: Health-systems oriented interventions

Appendix 13: Community-level interventions

Figure legends

Figure 1. PRISMA flow diagram

Figure 2: Random-effects model of short-term sensitisation and education campaigns (AA2)

Figure 3: RVE of interventions of written or pictorial messages to caregivers (AB4), mean baseline coverage values

For peer review only - http://bmjopen.bmj.com/site/about/guidelines.xhtml

Figure 1: PRISMA flow diagram

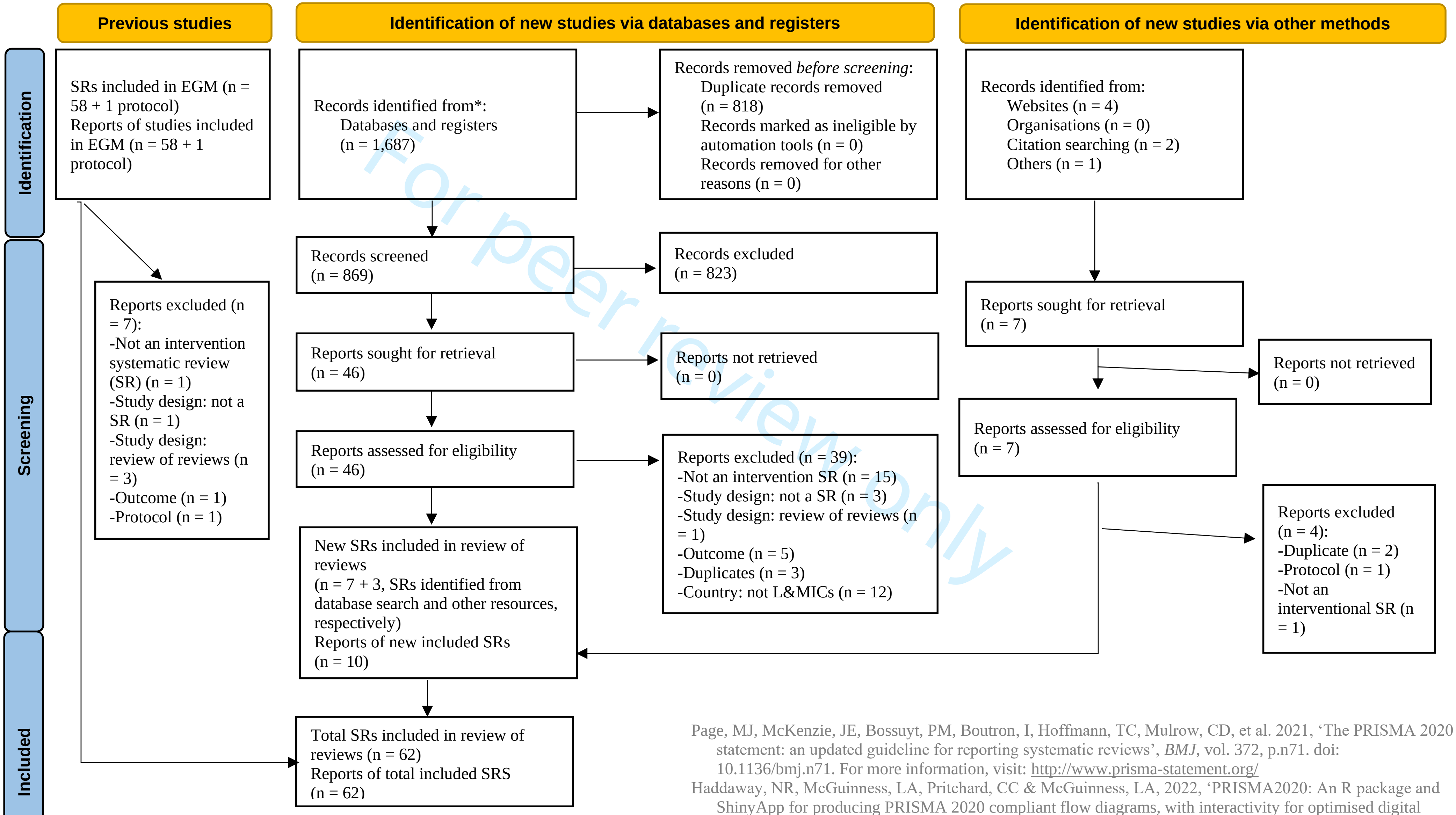

Page, MJ, McKenzie, JE, Bossuyt, PM, Boutron, I, Hoffmann, TC, Mulrow, CD, et al. 2021, 'The PRISMA 2020 statement: an updated guideline for reporting systematic reviews', *BMJ*, vol. 372, p.n71. doi: 10.1136/bmj.n71. For more information, visit: http://www.prisma-statement.org/

Haddaway, NR, McGuinness, LA, Pritchard, CC & McGuinness, LA, 2022, 'PRISMA2020: An R package and ShinyApp for producing PRISMA 2020 compliant flow diagrams, with interactivity for optimised digital transparency and Open Synthesis', *Campbell Systematic Reviews*, vol. 18, no. 2, p.e1230. doi: 10.1002/cl2.1230

For peer review only

Figure 2: Random-effects model of short-term sensitisation and education campaigns (AA2)

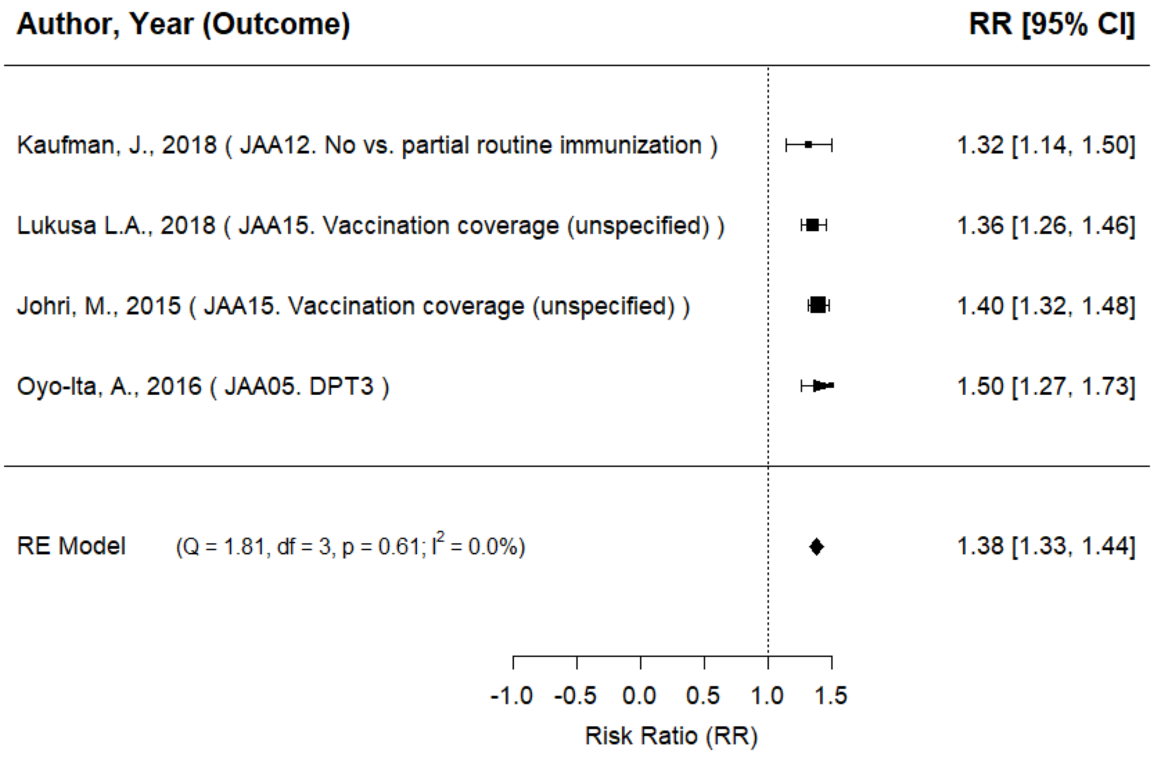

Note: Intervention and outcome codes are taken from Appendix 1.

Figure 3: RVE of interventions of written or pictorial messages to caregivers (AB4), mean baseline coverage values

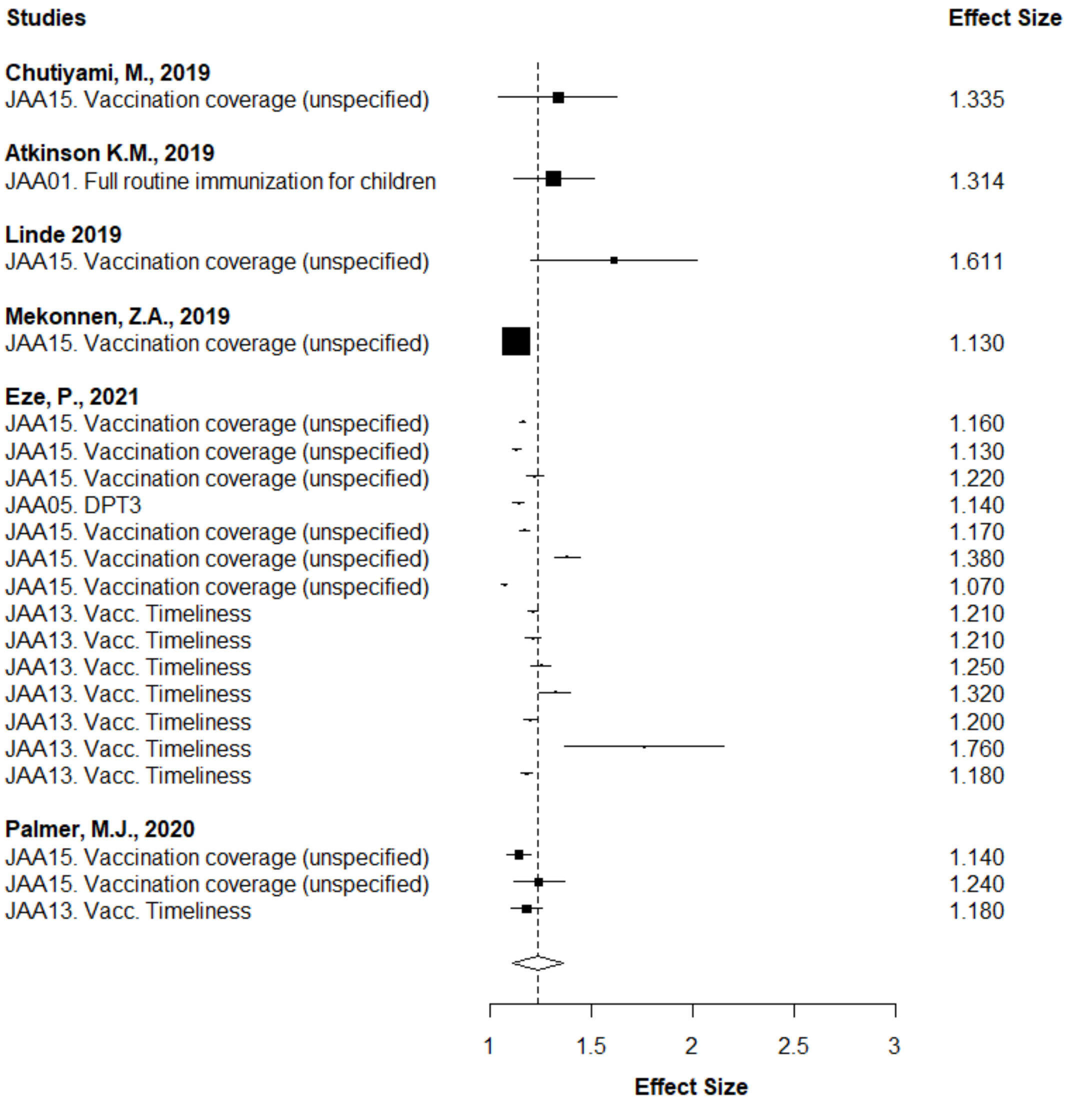

Note: Intervention and outcome codes are taken from Appendix 1.

Supplementary file

Contents

Appendix 1: Intervention-outcome framework	2
Appendix 2: Detailed inclusion and exclusion criteria	10
Appendix 3: List of excluded studies – after full-text screening	12
Appendix 4: List of sources and search strings	15
Appendix 5: Data extraction template	19
Appendix 6: Quality appraisal tool	22
Appendix 7: Summary of included systematic reviews	35
Appendix 8: Risk of bias of included systematic reviews	50
Appendix 9: Summary of main findings	54
Appendix 10: RVE analysis – 1st tier synthesis	71
Appendix 11: RVE analysis – 2nd tier synthesis	74
Appendix 12: Health-systems oriented interventions	77
Appendix 13: Community-level interventions	81
References	82

For peer review only

Appendix 1: Intervention-outcome framework

Interventions

Intervention			Definition/guidance
A. Caregiver-oriented	AA. Information & education	AA1. Sustained sensitisation and education campaigns	Sustained interventions (i.e., those that are *not* designed with a fixed end date in mind) that provide targeted caregivers with information about immunisation and its importance, the vaccination schedule, or where and how to access immunisation services. For example, village health and nutrition days (VHNDs) in India in which health education and counselling services are provided to pregnant women and mothers of young children on a regular basis.
		AA2. Short-term[1] sensitisation and education campaigns	One-off interventions (i.e., those designed with a fixed end date in mind) that provide targeted caregivers with information about immunisation and its importance, the vaccination schedule, or where and how to access immunisation services.
		AA3. Public information campaigns	Mass media campaigns through newspapers, radio and TV which provide caregivers with information about immunisation and its importance, the vaccination schedule, or where and how to access immunisation services. Because of the nature of communication mediums they cannot be targeted to a specific audience.
	AB. Incentives & motivation	AB1. Material/ monetary incentives for caregivers	Interventions that incentivise caregivers to vaccinate through items with monetary value. This could be cash transfers or material goods like food or home goods.
		AB2. Non-material incentives for caregivers	Interventions that seek to motivate caregivers to vaccinate through non-material incentives like social recognition. Unlike reminder messages, interventions in this category should seek to *create* or *strengthen* a desire to vaccinate, rather than activating a standing intention to vaccinate.
		AB3. Automated voice messages to caregivers	Use of automatically-generated voice messages (usually delivered to a mobile phone) that remind caregivers about upcoming vaccinations, provide them information on place and time of vaccination and encourage them to vaccinate. While these messages may contain some motivational component (e.g., stressing the importance of vaccination in addition to reminding caregivers about an upcoming appointment), voice messages should be categorised only here and not also under "non-material incentives", unless they rely on a substantive motivational factor like social recognition.
		AB4. Written or pictorial messages (SMS, stickers, flyers etc.) to caregivers	Use of written messages/pictorial that remind caregivers about upcoming vaccinations, provide them information on place and time of vaccination and encourage them to vaccinate. While these messages may contain some motivational component (e.g., stressing the importance of vaccination in addition to reminding caregivers about an upcoming appointment), messages should be categorised only here and not also under "non-material incentives", unless they rely on a substantive motivational factor like social recognition.
		AB5. Changes to health system user fees	Any change to the monetary costs to users for accessing the health system. This can include introduction or elimination of fees at the point of service, or pre-payment or insurance schemes.

[1] In this review, we have amended the terminology from one-time sensitisation to short-term sensitization.

For peer review only - http://bmjopen.bmj.com/site/about/guidelines.xhtml

Intervention			Definition/guidance
B. Health system-oriented	BA. Education & training	BA1. Formal health worker training and education	Programmes that train or educate formal health workers (FHWs). FHWs are typically vaccinators (and they tend to provide/prescribe medication or administer tests such as recording blood glucose level, etc.). (The only likely exception to this would be oral polio vaccination (OPV), especially supplementary polio campaigns, where CHWs or community volunteers may be enlisted to administer the vaccination.)
		BA2. Community health worker training and education	Programmes that train or educate community health workers (CHWs). CHWs are defined as "paraprofessionals or lay individuals with an in-depth understanding of the community culture and language, have received standardised job-related training of a shorter duration than health professionals, and their primary goal is to provide culturally appropriate health services to the community."[2]
	BB. Planning, implementation, monitoring	BB1. Formal health worker involvement in planning & monitoring	Interventions that give FHWs substantive roles in creating plans/strategies to deliver vaccination services and/or monitor vaccination coverage in the community.
		BB2. Community health worker involvement in planning & monitoring	Interventions that give CHWs substantive roles in creating plans/strategies to deliver vaccination services and/or monitor vaccination coverage in the community.
		BB3. Paper-based tracking	Paper-based systems (e.g., logbooks) used by health workers to keep track of children in the community who are due for upcoming vaccinations or have not received scheduled vaccinations. Note that if a study merely mentions the existence of logbooks, that is not sufficient to code it as this intervention. The logbooks must be specifically used for tracking upcoming and missed vaccinations.
		BB4. Promoting outreach to vaccine-hesitant groups	Outreach to groups that, because of religious, cultural, or other reasons, are suspicious of vaccination or have specific fears about it (e.g., that vaccinations cause infertility or spread disease).
		BB5. Outreach to vulnerable populations (hard-to-reach, SES, caste, etc.)	Outreach to groups that are vulnerable in a way that affects their access to vaccination services. They may be in hard-to-reach geographical areas, have low socioeconomic status (including wealth and education), or be from groups marginalised based on caste, ethnicity, etc. This also includes interventions that set up temporary and mobile clinics to deliver vaccines, to make vaccination services more accessible.
		BB6. Outreach to migrant populations	Outreach to populations who have migrated temporarily or seasonally because of cultural or employment reasons.

[2] From Olaniran, A., Smith, H., Unkels, R., Bar-Zeev, S., & van den Broek, N. (2017). Who is a community health worker? – A systematic review of definitions. *Global Health Action*, *10*(1), 1272223. https://doi.org/10.1080/16549716.2017.1272223.

Intervention			Definition/guidance
		BB7. Home visits	Use of visits to caregivers' homes by health workers. This includes both visits to encourage caregivers to vaccinate their children, and visits to deliver vaccines, provided they are *targeted* visits to specific households for routine immunization. General door-to-door campaigns to deliver specific vaccines go under BC1.
		BB8. Campaigns to vaccinate refugee populations	Interventions that make a special effort to vaccinate populations which have been displaced temporarily or permanently because of conflict, war or famine.
	BC. Supplementary Immunisation Activities	BC1. National/sub-national immunisation days	Supplementary immunisation activities (SIA) are mass immunization campaigns which complement routine immunisation activities whereby a vaccine is taken simultaneously to many residents of a community within a defined short space of time. They have generally been conducted for polio and measles. They may be called national or sub-national immunisation days. They may happen through booth days or door to door vaccination campaigns. On a booth day a large number of fixed site booths are set up throughout the target area for children to be brought to receive a specific vaccine like polio. In door-to-door campaigns vaccination teams go door to door to every house, checking each child under five to see if they have received the specific vaccine, and if they have not that vaccination is provided.
	BD. Incentives & motivation	BD1. Material/monetary incentives for health workers	Interventions that incentivise formal or community health workers to deliver vaccination services through items with monetary value. This could be cash transfers or material goods like food or home goods.
		BD2. Non-material incentives for health workers	Interventions that use non-material incentives like social recognition to incentivise formal or community health workers to deliver vaccination services.
		BD3. Automated voice messages to health workers	Use of automatically-generated voice messages (usually delivered to a mobile phone) that remind health workers about upcoming vaccinations for community members, provide them information to help plan their work, and encourage them to conduct outreach to those community members.
		BD4. Written or pictorial messages (SMS, stickers, flyers etc.) to health workers	Use of written/pictorial messages that remind health workers about upcoming vaccinations for community members, provide them information to help plan their work, and encourage them to conduct outreach to those community members.
		BD5. Pay-for-performance schemes	Schemes whereby health centres or districts receive funding based on their performance in delivering health services (e.g., they receive a given amount of funding for each child vaccinated or each antenatal care visit completed). Also known as "results-based financing".
	BE. Infrastructure	BE1. Building & upgrading health clinics	Projects that build new permanent health clinics, or provide physical upgrades to existing clinics.
		BE2. Cold chain infrastructure improvements	Interventions that improve the ability of health systems to maintain vaccine cold chains.

For peer review only

Intervention			Definition/guidance
	BF. Health system governance, policies and financing	BF1. Health system strategic planning	Initiatives at the national or sub-national level to develop plans and governance structures designed to improve vaccination services. This also includes interventions that improve the human resource availability, strategies, policies and plans in existing health governance and delivery structures that may or may not be directly related to immunisation services.
		BF2. Vaccination guidelines	Changes to official national or sub-national guidelines about when and how vaccinations should be administered. This includes studies comparing two different approaches to administering vaccines (e.g., one measles dose vs. two), which could be made into guidelines.
		BF3. Changes to broader governance systems (beyond health systems)	Interventions that modify general governance systems not directly related to health. An example would be a policy dictating a certain level of representation for women in local or national governing bodies.
		BF4. Health system financing	Interventions that increase the national or sub-national financing of health or specifically for vaccination in absolute terms or as a proportion of GDP. This also includes results- or performance-based financing.
	BG. Technology & mHealth	BG1. New HMIS/Dashboard systems (incl. improved data collection)	New digital tools and systems designed to improve health system capacity to monitor and deliver vaccination services. This includes apps for mobile phones or tablets, as well as desktop-based software and setting up online dashboards. A common type of intervention in this category is giving health workers tablets with an app that allows them to register vaccination information (and for other health services) for community members, track their vaccination schedules and provide them readily-accessible information about vaccination.
		BG2. Capacity building (e.g., training) for existing systems	Initiatives to train people working in the health system (including frontline health workers and administrative personnel) to improve their ability to use existing digital tools and systems more effectively.
C. Other community member-oriented	CA. Other community member-oriented	CA1. Faith-based outreach/outreach using local leaders	Interventions that enlist influential community members (often religious or other traditional leaders) to promote vaccination in the community.
D. Community-level	DA. Communication & dialogue	DA1. Collaborating with whole community	Interventions that involve or plan to involve ALL community members beyond health workers in various aspects of the intervention, such as developing plans and solutions to improve immunisation outcomes in the community.
		DA2. Collaborating with selected community groups and networks	Interventions that involve *selected* groups or networks of community members beyond health workers (other than the traditional or religious leaders who fall under CA1) in developing plans and solutions to improve immunisation outcomes in the community. This includes interventions focusing on mother's groups, father clubs, self-help groups, etc.

For peer review only

Intervention			Definition/guidance
	DB. Tracking & registering	DB1. Community tracking and registering	Interventions that involve community members beyond health workers (e.g., caregivers or any other community members other than the traditional leaders who fall under CA1) in registering children with the health system so their vaccination status can be tracked, and/or tracking which children are due for vaccinations.
E. Policies and institutions	EA. Education policy and infrastructure	EA1. Education policy and infrastructure	Policy interventions that affect education levels of people in a country or region. An example would be an intervention that makes education mandatory for a particular population where it had previously been optional.
	EB. Non-health/ education infrastructure	EB1. Non-health/ education infrastructure (e.g., electrification)	General improvements in physical infrastructure beyond the health system. This may include electrification, roads, sanitation improvements, etc.
F. Multicomponent			

Outcomes

Outcome			Definition/guidance
H. Behavioural, social and practical barriers faced by caregivers and communities	HAA. Thinking and feeling (attitudes, confidence)	HAA1. Knowledge about immunisation	Caregivers' knowledge about immunisation in general (i.e., its purpose and role in preventing disease)
		HAA2. Attitudes about immunisation	Caregivers' attitudes towards immunisation in general (i.e., whether they view it favourably or unfavourably or have high or low confidence in its efficacy)
		HAA3. Attitudes about health providers	Caregivers' attitudes about health providers in general (i.e., whether they generally trust health providers to provide high-quality and appropriate care)
	HAB. Social processes	HAB1. Community norms	Community-level attitudes and beliefs about immunisation, including whether there is social pressure to vaccinate or not vaccinate. This can be measured either objectively through aggregating community-level responses or subjectively by soliciting individual community members' beliefs about the norms in their community. This includes attitudes and beliefs about immunization of key influencers in the community like traditional or religious leaders.
		HAB2. Household norms & decision-making	Norms and practices determining who in a household (e.g., mother, father, mother-in-law) provides input to decisions about whether to vaccinate, and how much decision-making power individual household members have. This also covers attitudes towards immunisation of household members other than the primary caregiver.
	HAC. Readiness to vaccinate	HAC1. Readiness to vaccinate	Caregivers' motivation, intention and plan to vaccinate their children. Note this is more specific than general attitudes towards immunisation covered under HAA2

Outcome				Definition/guidance
			HAC2. Reasons for not vaccinating	Caregivers' stated reasons for not vaccinating children. This may include factors such as convenience (which would also be coded under "perceived convenience of vaccination"), but only use this code if the factors mentioned are specifically framed as reasons for not vaccinating. Also, only use this code when the study measures *effects* of the intervention on this outcome. If the study gathers data on reasons for not vaccinating but does not provide an effect size for this as an outcome, use the cross-cutting theme but not this outcome code.
		HAD. Practical factors	HAD1. Awareness of place, time, schedule for vaccination	Caregivers' knowledge about when and where they should go for vaccinations.
			HAD2. Actual cost of vaccinating	Actual cost of vaccinating the child, including vaccine cost, transportation cost, loss of wage/income due to missed work, and providing gifts/unofficial payments to the health providers
			HAD3. Perceived convenience of vaccination	Subjective measures (i.e., caregivers' beliefs) of the convenience of taking the child for vaccination, such as opportunity costs of vaccinating a child (e.g., not able to care for a younger child), long lines at health clinic, and inconvenient day/time of vaccination.
			HAD4. Experience and satisfaction with health services	The actual experience of health services in the last visit such as duration of waiting time, availability of vaccine or vaccinator, and behaviour of the health staff (respect, rudeness). This also includes level of satisfaction with the health services, professionals and facilities.
			HAD5. Vaccination health card availability/ retention	Measures of whether caregivers possess vaccination health cards provided by the health system, and/or whether caregivers can show the vaccination health card.
			HAD6. Perception of vaccination side effects	Caregivers' perceptions of the likelihood and severity of side effects from vaccination, and their knowledge of how to recognise and treat normal side effects.
I. Delivery of vaccination services	IA. Health workforce	IAA. Comm. health workers	IAA1. Community HW motivation, capacity & performance	Any measure of CHWs' capacity to deliver quality and timely vaccination services.
			IAA2. Supply of CHWs	The total availability of CHW services in the community, taking into account both the number of CHWs and the time they have available.
		IAB. Vaccinators	IAB1. Formal HW supply	The total availability of FHW services in the community, taking into account both the number of FHWs and the time they have available.
			IAB2. Availability of HWs at vacc. point of service	Whether vaccinators are present at vaccination point of service (e.g., health clinic) when vaccination services are supposed to be offered. This includes measures of health worker "absenteeism". This is different from perception or experience of health workers from caregivers' point of view. The source of information for this outcome can be administrative data or survey of health facilities and staff.

For peer review only

Outcome				Definition/guidance
			IAB3. Formal HW motivation, capacity & performance	Any measure of FHWs' capacity to deliver quality and timely vaccination services, or of their performance in doing so.
		IAC. Administrators	IAC1. Admin staffing	The number of staff in administrative posts in the health system (i.e., those not directly involved in health service provision).
			IAC2. Capacity of health admin. responsible for vaccination	The knowledge, skills, and motivation of staff in administrative posts in the health system, including leadership positions.
	IBA. Health information systems		IBA1. Immunisation data collection (quality, completeness)	The health system's capacity for and success in collecting data about vaccination coverage and service quality for regular monitoring and accountability.
			IBA2. Defaulter tracing	The health system's capacity for and success in identifying vaccination "defaulters" (i.e., those whose children receive early vaccine doses but do not return for subsequent vaccinations).
			IBA3. Supply chain management	The health system's capacity to monitor the supply of vaccines at points of service, ensure reliable supply chains, and avoid supply "bottlenecks".
			IBA4. Immunisation data availability/ transparency	How easy it is for stakeholders within and beyond the health system to access data about vaccination service quality, coverage and timeliness.
	ICA. Vaccine availability		ICA1. Stockouts	The frequency and duration of incidents when vaccines are out of stock at points of service, or when vaccines are in stock but cannot be administered to children who are brought in (e.g., because health workers are instructed not to open a new vial if there are not enough children to receive all doses in the vial, meaning that some of the vaccine would go to waste). Source of this information is administrative data and/or survey of health facilities and staff
			ICA2. Quality of cold chain infrastructure	The availability, quality and upkeep of physical equipment and place for cold chain storage of vaccines
	IDA. Resources		IDA1. National or sub-national vaccine financing	Change in financial resources for national or sub-national vaccination programmes, policies or strategies.

JAA. Vaccination coverage	JAA01. Full routine immunisation for children	Binary measure of whether or not children have received all routine vaccinations for the relevant country or region.
	JAA02. BCG	Binary measure of whether or not children have received the BCG vaccine. This may be measured by checking whether children have a BCG vaccination scar.

For peer review only

	JAA03. DPT1	Binary measure of whether children have received first dose of the DPT/penta vaccine. If study does not specify which doses were included in the outcome measures (i.e., the outcome is just "DPT/pentavalent vaccination" then apply this code unless it is clear they are talking about full DPT/penta vaccination, in which case code it as JAA05: DPT3.
	JAA04. DPT2	Binary measure of whether or not children have received the second dose of the DPT or pentavalent
	JAA05. DPT3	Binary measure of whether or not children have received the third dose of the DPT or pentavalent vaccine. If the study does not specifically say "DPT3" (or "pentavalent 3"), but refers to "complete DPT/penta vaccination", then use this code.
	JAA06. OPV0	Binary measure of whether children have received 1st dose of oral polio vaccine (recommended for administration at birth).
	JAA07. OPV1	Binary measure of whether children have received 2nd dose of the oral polio vaccine (recommended for administration at 6 weeks).
	JAA08. OPV2	Binary measure of whether children have received the 3rd dose of the oral polio vaccine (recommended for administration at 10 weeks).
	JAA09. OPV3	Binary measure of whether children have received the 4th and final dose of the oral polio vaccine (recommended for administration at 14 weeks).
	JAA10. IPV	Binary measure of whether children have received inactivated polio vaccine, given as injection. Countries differ in their guidelines/practices regarding IPV, so, please note the number of doses and age(s) when administered.
	JAA11. Measles	Binary measure of whether or not children have received the measles vaccine
	JAA12. No vs. partial routine immunisation	Proportion of children who receive at least one vaccination versus those who are completely unvaccinated.
	JAA13. Vacc. timeliness	Proportion of vaccinations delivered on time according to the recommended schedule, vs those that are delivered late.
	JAA14. Drop out rate for multidose vaccines	Proportion of children who fail to receive the complete course of a multidose vaccine (DPT/penta, OPV, or in some cases measles) after receiving the first dose.
	JAA15. Vaccination coverage (unspecified)	Use this code if the evaluation or systematic review refers to impacts on routine vaccination coverage for children, but without specifying which vaccines.
KAA. Health outcomes	KAA1. Childhood morbidity	Incidence of vaccine-preventable diseases or symptoms associated with those diseases (e.g., diarrhoea) among children under 5.
	KAA2. Neonatal/Infant/ Child Mortality	Incidence of mortality among children below five years from all causes. Neonatal mortality refers to death of a live-born baby within the first 28 days of life. *Infant mortality* is the death of young children under the age of 1. Child mortality, refers to the mortality of children under the age of five. This category includes all-cause mortality.

For peer review only - http://bmjopen.bmj.com/site/about/guidelines.xhtml

Appendix 2: Detailed inclusion and exclusion criteria

1. Types of reviews

We include studies that self-identify as systematic reviews and/or meta-analyses of the impacts of interventions on outcomes related to routine child immunisation in L&MICs. These in turn have focused on synthesising quantitative, qualitative and or mixed methods evidence. According to the Campbell Collaboration,

> "A systematic review summarizes the best available evidence on a specific question using transparent procedures to locate, evaluate, and integrate the findings of relevant research" (The Campbell Collaboration, 2020, p.5).

In the Cochrane Handbook for Systematic Reviews of Interventions (Higgins et al., 2019), the following definition of systematic reviews is outlined:

> "A systematic review attempts to collate all empirical evidence that fits pre-specified eligibility criteria in order to answer a specific research question. It uses explicit, systematic methods that are selected with a view to minimizing bias, thus providing more reliable findings from which conclusions can be drawn and decisions made" (Section 1.1 in Lasserson, Thomas, & Higgins, 2019, p.4).

Higgins and colleagues (2019) specify the key elements a systematic review should contain:

- A set of clearly stated objectives and pre-defined eligibility criteria
- A methodology that is clearly defined allowing reproducibility
- A search strategy that allows the identification of studies meeting the pre-defined eligibility criteria
- A critical appraisal of included studies
- A systematic synthesis, in many cases systematic reviews adopt a meta-analytical approach which is a statistical method to synthesise the results of primary studies included in a systematic review

To identify meta-analyses, the Cochrane Handbook for Systematic Reviews of Interventions (Higgins et al., 2019) provides a useful definition:

> "...meta-analysis yields an overall statistic (together with its confidence interval) that summarises the effectiveness of an experimental intervention compared with a comparator intervention. (Section 10.2 in Deeks, Higgins, & Altman, 2019, p. 242).

Not every systematic review contains a meta-analysis. For example, if too much heterogeneity exists within the evidence base in terms of study designs, conceptual framings and or outcomes, then a meta-analysis may not be appropriate and instead a narrative synthesis approach may be adopted and those were included in this review of reviews.

For the purpose of this study, we adapt the definitions outlined above and use the following inclusion criteria:

1. The study systematically searches, screens, extracts data and synthesises the evidence.
2. It describes methods used for search, screening, data extraction and synthesis in sufficient detail.

We carry out a quality appraisal of the reviews, which meet the above inclusion criteria. The critical appraisal tool used for this review (more details on this below) encapsulates the stringent inclusion criteria of the Campbell and Cochrane Collaborations as outlined above.

For peer review only

Types of participants

The systematic reviews and meta-analyses we have included are diverse. For example, different questions are often addressed, and a range of interventions are examined, which may look not only at immunisation outcomes such as coverage but also intermediate outcomes such as caregiver perceptions of child vaccination or capacity of health system to deliver quality child vaccination services. While the principal population of interest is children below the age of five, there may be other populations included in our analyses because of assessments of impact on intermediate outcomes. For example, some of the outcomes may have been analysed on the population of caregivers or frontline health workers and are included in our review as long as they are related to improving routine child immunisation outcomes in L&MICs using the World Bank (2022) definition[3]. Where systematic reviews and meta-analyses include evidence from HICs, we only considered the findings if more than 50% of studies are from L&MICs or if it is less than 50%, then at least two studies are from L&MICs and/or the evidence from L&MICs has been synthesised separately; we also considered systematic reviews and meta-analyses covering particular regions within L&MICs, e.g. Sub-Saharan Africa or fragile and conflict-affected areas; additionally we considered systematic reviews that are covering only one country within L&MICs where sufficient evidence is available for synthesis.

Types of interventions

In this systematic review of reviews, we included all systematic reviews and meta-analyses that provided evidence on the impact of any intervention on routine immunisation coverage or any intermediate outcome that precedes them in the theoretical causal chain (as outlined above). Therefore, the interventions included had to assess at least one outcome in relation to routine child immunisation (see Appendix 1, intervention-outcome framework for details).

Types of outcome measures

In this systematic review of reviews, we included all systematic reviews and meta-analyses that address any of the following outcomes: Coverage rates or timeliness of full immunisation, third dose of diphtheria, pertussis and tetanus (DPT) or pentavalent, or measles; additional antigen-specific immunisation coverage outcomes; and intermediate outcomes that precede them in the theoretical causal chain (e.g., attitudes about vaccination, and access to immunisation services) (see Appendix 1).

[3] The World Bank definition of lower/middle income countries is used: https://datahelpdesk.worldbank.org/knowledgebase/articles/906519-world-bank-country-and-lending-groups

For peer review only

Appendix 3: List of excluded studies – after full-text screening

Academic databases

Sr. No.	Authors & Year	Title	Reasons
1	Asciutto et al., 2020[1]	"A systematic review of economic evaluations of neonatal and maternal healthcare in immigrant and ethnic minority women"	Exclude on country
2	Balzarini et al., 2020[2]	Does the use of personal electronic health records increase vaccine uptake? A systematic review	Exclude on country
3	Diks, Hiligsmann, & van der Putten, 2021[3]	Vaccine preferences driving vaccine-decision making of different target groups: a systematic review of choice-based experiments	Exclude on country
4	Haroune & King, 2020[4]	Factors contributing to parental 'vaccine hesitancy' for childhood immunisations	Exclude on country
5	Hutchinson & Smith, 2020[5]	Effectiveness of strategies to increase uptake of pertussis vaccination by new parents and family caregivers: A systematic review	Exclude on country
6	Jia et al., 2021[6]	Payment methods for healthcare providers working in outpatient healthcare settings	Exclude on country
7	Lambert et al., 2021[7]	Reducing burden from respiratory infections in refugees and immigrants: a systematic review of interventions in OECD, EU, EEA and EU-applicant countries	Exclude on country
8	Machado et al., 2021[8]	Effective interventions to increase routine childhood immunization coverage in low socioeconomic status communities in developed countries: A systematic review and critical appraisal of peer-reviewed literature	Exclude on country
9	Odone et al., 2021[9]	Current understandings of the impact of mandatory vaccination laws in Europe	Exclude on country
10	Ortiz-Sánchez et al., 2020[10]	Analysis of the Anti-Vaccine Movement in Social Networks: A Systematic Review	Exclude on country
11	Pires, 2021[11]	What is the state-of-the-art in clinical trials on vaccine hesitancy 2015-2020?	Exclude on country
12	Reñosa et al., 2021[12]	Nudging toward vaccination: a systematic review	Exclude on country
13	Bednarek & Klepacz, 2020[13]	Vaccinology education of nurses and the current immunoprophylaxis recommendations for children with juvenile idiopathic arthritis	Exclude on outcome
14	Chenet & Tapia-Limonchi, 2021[14]	Reaching the theoretical herd immunity threshold in Iquitos, Peru: are seroprevalence data enough?	Exclude on outcome
15	Chitkara et al., 2020[15]	Pertussis vaccination in mixed markets: Recommendations from the Global Pertussis Initiative	Exclude on outcome
16	Meteke et al., 2020[16]	Delivering infectious disease interventions to women and children in conflict settings: A systematic review	Exclude on outcome
17	Nnaji et al., 2021[17]	Implementation research approaches to promoting universal health coverage in Africa: a scoping review	Exclude on outcome
18	Chaney et al., 2021[18]	Every child on the map: A theory of change framework for improving childhood immunization coverage and equity using geospatial data and technologies	Exclude on study design, not a systematic review
19	Soans et al., 2021[19]	Vaccination in preterm and low birth weight infants in India	Exclude on study design, not a systematic review
20	Tankwanchi et al., 2020[20]	Taking stock of vaccine hesitancy among migrants: A scoping review protocol	Exclude on study design, not a systematic review

For peer review only

21	Agrawal et al., 2020[21]	Vaccine Hesitancy as a Challenge or Vaccine Confidence as an Opportunity for Childhood Immunisation in India	Not an effectiveness/intervention systematic review
22	Barbosa de Souza et al., 2021[22]	EXPERIÊNCIAS BRASILEIRAS NO ACOMPANHAMENTO DE CRESCIMENTO E DESENVOLVIMENTO INFANTIL NO CONTEXTO DA ATENÇÃO BÁSICA	Not an effectiveness/intervention systematic review
23	Berrueta et al., 2021[23]	Maternal and neonatal data collection systems in low- and middle-income countries for maternal vaccines active safety surveillance systems: A scoping review	Not an effectiveness/intervention systematic review
24	Cassocera et al., 2020[24]	40 years of immunization in Mozambique: a narrative review of literature, accomplishments, and perspectives	Not an effectiveness/intervention systematic review
25	Dansereau et al., 2020[25]	A systematic review of the agreement of recall, home-based records, facility records, BCG scar, and serology for ascertaining vaccination status in low and middle-income countries	Not an effectiveness/intervention systematic review
26	Farida, Widyaningsih, & Murti, 2020[26]	The effect of maternal education and antenatal care on basic immunization completeness in children aged 12-23 months in Asian and African: meta-analysis	Not an effectiveness/intervention systematic review
27	Galadima et al., 2021[27]	Factors influencing childhood immunisation uptake in Africa: a systematic review	Not an effectiveness/intervention systematic review
28	Kalaij, Sugiyanto, & Ilham, 2021[28]	Factors Associated With Vaccination Compliance in Southeast Asian Children: A Systematic Review	Not an effectiveness/intervention systematic review
29	Kulkarni et al., 2021[29]	Trends in Urban Immunization Coverage in India: A Meta-Analysis and Meta-Regression	Not an effectiveness/intervention systematic review
30	Laserna Jiménez et al., 2021[30]	Paediatric nursing clinical competences in primary healthcare: A systematic review	Not an effectiveness/intervention systematic review
31	Lassi et al., 2021[31]	The impact of the COVID-19 pandemic on immunization campaigns and programs: A systematic review	Not an effectiveness/intervention systematic review
32	Lieneck, Herzog, & Krips, 2021[32]	Analysis of Facilitators and Barriers to the Delivery of Routine Care during the COVID-19 Global Pandemic: A Systematic Review	Not an effectiveness/intervention systematic review
33	Wariri et al., 2021[33]	Timeliness of routine childhood vaccination in low- And middle-income countries, 1978-2021: Protocol for a scoping review to map methodologic gaps and determinants	Not an effectiveness/intervention systematic review
34	Priya et al., 2020[34]	Vaccination coverage and vaccine hesitancy among vulnerable population of India	Not an effectiveness/intervention systematic review
35	Smith, Hodson, & Rubin, 2021[35]	Parental attitudes towards mandatory vaccination; a systematic review	Not an effectiveness/intervention systematic review
36	Besnier et al., 2021[36]	Which public health interventions are effective in reducing morbidity, mortality and health inequalities from infectious diseases amongst children in low- and middle-income countries (L&MICs): An umbrella review	Exclude on study design, it is a review of reviews
37	Munk et al., 2019[37]	Systematic review of the costs and effectiveness of interventions to increase infant vaccination coverage in low- and middle-income countries	Duplicate

For peer review only

38	Omoniyi & Williams, 2020[38]	Realist Synthesis of the International Theory and Evidence on Strategies to Improve Childhood Vaccination in Low- and Middle-Income Countries: Developing Strategies for the Nigerian Healthcare System	Duplicate
39	Barbosa de Souza et al., 2021[22]	Brazilian experiences in monitoring growth and child development in the context of primary care (EXPERIÊNCIAS BRASILEIRAS NO ACOMPANHAMENTO DE CRESCIMENTO E DESENVOLVIMENTO INFANTIL NO CONTEXTO DA ATENÇÃO BÁSICA)	Duplicate

Grey literature

Sr. No.	Authors & Year	Title	Reasons
1	Siribaddana, Sahay, & Hewapathirana, 2021[39]	Deploying Electronic Immunization Information Systems in Low and Middle Income Countries in Response to COVID-19: Systematic Review of Best Evidence Practices	Not an effectiveness/intervention systematic review
2	Jain et al., 2020[40]	PROTOCOL: Use of community participation interventions to improve child immunisation in low- and middle-income countries: A systematic review and meta-analysis	Exclude as it is a protocol: completed review (unpublished at the time of updated search) has been included, Jain et al., 2022[41, 42]
3	Onwucheckwa et al., 2021[43]	Systematic Review on the Impact of Conditional Cash Transfers on Child Health Service Utilisation and Child Health in Sub-Saharan Africa	Duplicate
4	Eze, Lawani & Acharya, 2021[44]	Short message service (SMS) reminders for childhood immunisation in low-income and middle-income countries: a systematic review and meta-analysis.	Duplicate

Systematic reviews previously included in EGM, excluded with reasons:

Sr. No.	Authors & Year	Title	Reasons
1	Pantoja et al., 2017[45]	Implementation Strategies for Health Systems in Low-Income Countries: An Overview of Systematic Reviews (Review)	Exclude on study design, it is a review of reviews
2	Hatt et al., 2015[46]	Impact of Health Systems Strengthening on Health	Exclude on study design, it is a review of reviews
3	Wiysonge, 2012[47]	Building evidence for improving childhood immunisation coverage in Africa	Exclude on study design, it is a review of reviews
4	Van Velthoven et al., 2020[48]	Use of Apps to Promote Childhood Vaccination: Protocol for a Systematic Review	Exclude as it is a protocol: final published review is included, de Cock 2020
5	Abdulrahman et al., 2017[49]	mHealth: a narrative synthesis of evidence of its application in improving childhood immunization coverage	Exclude on study design, not a systematic review
6	Aaby et al., 2016[50]	Randomized Trials Comparing Inactivated Vaccine After Medium-or High-titer Measles Vaccine With Standard Titer Measles Vaccine After Inactivated Vaccine	Not an effectiveness/intervention systematic review
7	Bhutta et al., 2008[51]	Interventions to address maternal, newborn, and child survival: what difference can integrated primary health care strategies make?	Exclude on outcomes

For peer review only

Appendix 4: List of sources and search strings

Electronic searches
We conducted electronic searches of the following databases of published sources:

1. MEDLINE (via Ovid)
2. CAB Global Health (Ovid)
3. EMBASE (Ovid)
4. Cochrane Controlled Trials Register (CENTRAL)
5. CINAHL (EbscoHost)
6. PsycINFO (Ovid)
7. Popline
8. Africa-wide information
9. Academic search complete
10. Scopus
11. Campbell Library

Searching other resources
To identify relevant grey literature, we searched the following databases and institutional websites:

1. Google Scholar
2. EconLit
3. IDEAS/RePEc
4. WHO Global Index Medicus
5. Pascal-Francis
6. Open-Grey
7. Grey Literature Report
8. Social Science Research Network (SSRN)
9. Eldis
10. GAVI
11. Epistemonikos
12. Innovations for Poverty Action (IPA)
13. Abdul Latif Jameel Poverty Action Lab (J-PAL)
14. 3ie Impact Evaluation Repository
15. 3ie Systematic Review Repository
16. Registry of International Development Impact Evaluations (RIDIE)
17. Global Development Network
18. World Bank Development Impact Evaluation (DIME) and Impact Evaluation Policy Papers
19. Inter-American Development Bank
20. Center for Global Development
21. Center for Effective Global Action (CEGA)
22. DFID Research for Development (R4D)
23. USAID

Search terms
L&MICs
1. (Afghanistan or Albania or Algeria or Angola or Argentina or Armenia or Armenian or Azerbaijan or Bangladesh or Benin or Byelarus or Byelorussian or Belarus or Belorussian or Belorussia or Belize or Bhutan or Bolivia or Bosnia or Herzegovina or Hercegovina or Botswana or Brazil or Bulgaria or Burkina Faso or Burkina Fasso or Upper Volta or Burundi or Urundi or Cambodia or Khmer Republic or Kampuchea or Cameroon or Cameroons or Cameron or Camerons or Cape Verde or Cabo Verde or Central African Republic or Chad or Tchad or China or Colombia or Comoros or Comoro Islands or Comores or Mayotte or Congo or Zaire or Costa Rica or Cote d'Ivoire or Ivory Coast or Cuba or Djibouti or French Somaliland or Dominica or Dominican Republic or East Timor or East Timur or Timor Leste or Ecuador or Egypt or United Arab Republic or El Salvador or Eritrea or Ethiopia or Fiji or Gabon or Gabonese Republic or Gambia or Gaza or Georgia Republic or Georgian Republic or Ghana or Grenada or Guatemala or Guinea or Guiana or Guyana or Haiti or Honduras or India or Maldives or Indonesia or Iran or Iraq or Jamaica or Jordan or Kazakhstan or Kazakh or Kenya or Kiribati or Korea or Kosovo or Kyrgyzstan or Kirghizia or Kyrgyz Republic or Kirghiz or Kirgizstan or Lao PDR or Laos or Lebanon or Lesotho or Basutoland or Liberia or Libya or Macedonia or Madagascar or Malagasy Republic or Malaysia or Malaya or Malay or Sabah or Sarawak or Malawi or Mali or Marshall Islands or Mauritania or Mauritius or Agalega Islands or Mexico or Micronesia or Middle East or Moldova or Moldovia or Moldovian or Mongolia or Montenegro or Morocco or Ifni or Mozambique or Myanmar or Myanma or Burma or Namibia or Nepal or Netherlands Antilles or Nicaragua or Niger or Nigeria or Muscat or Pakistan or Palau or Palestine or Panama or Paraguay or Peru or Philippines or Philipines or Phillipines or Phillippines or Papua New Guinea or Romania or

For peer review only

Rumania or Roumania or Rwanda or Ruanda or Saint Lucia or St Lucia or Saint Vincent or St Vincent or Grenadines or Samoa or Samoan Islands or Navigator Island or Navigator Islands or Sao Tome or Senegal or Serbia or Montenegro or Seychelles or Sierra Leone or Sri Lanka or Solomon Islands or Somalia or Sudan or Suriname or Surinam or Swaziland or Eswatini or South Africa or Syria or Tajikistan or Tadzhikistan or Tadjikistan or Tadzhik or Tanzania or Thailand or Togo or Togolese Republic or Tonga or Tunisia or Turkey or Turkmenistan or Turkmen or Uganda or Ukraine or Uzbekistan or Uzbek or Vanuatu or New Hebrides or Venezuela or Vietnam or Viet Nam or West Bank or Yemen or Zambia or Zimbabwe or Rhodesia)

2. (Africa or Asia or Caribbean or West Indies or South America or Latin America or Central America)
3. ((developing or less* developed or least developed or under developed or underdeveloped or middle income or low* income or underserved or under served or deprived or poor* or resource limited or resource constrained) adj (countr* or nation? or population? or world or state* or emerging econom* or global south))
4. ((developing or less* developed or least developed or under developed or underdeveloped or middle income or low* income or resource limited or resource constrained) adj (economy or economies))
5. (low* adj (gdp or gnp or gross domestic or gross national))
6. (low adj3 middle adj3 countr*)
7. (lmic or lmics or third world or lami countr*)
8. transitional countr*
9. 1 OR 2 OR 3 OR 4 OR 5 OR 6 OR 7

Immunisation

1. (immuniz* or immunis* or vaccin* or inoculat* or innoculat* or immunotherap* or prophyla*)

Young children and caregivers

24. (child* or infant* or newborn* or neonat* or prenatal or pre natal or antenatal or ante natal or baby or babies or toddler* or preschool* or parent* or mother* or father* or maternal or paternal or caregiver* or grandparent* or grandmother* or grandfather* or family member*)

Impact evaluation methods

12. (random* or experiment* or (match* adj2 (propensity or coarsened or covariate)) or "propensity score" or "difference in difference*" or "difference-in-difference*" or "differences in difference*" or "differences-in-difference*" or "double difference*" or "quasi-experimental" or "quasi experimental" or "quasi-experiment" or "quasi experiment" or ((estimator or counterfactual) and evaluation*) or "instrumental variable*" or (IV adj2 (estimation or approach)) or regression discontinuity or time series or segment* regression)

Example full search strategy

Below we present a draft of the full search strategy used to search MEDLINE. Note that in MEDLINE's syntax, terms with strokes (e.g., Immunization/) denote Medical Subject Heading (MeSH) terms, while strings appended with "ti,ab,kw" are searched in the title, abstract, and keyword fields of records in the database.

Ovid MEDLINE(R) and Epub Ahead of Print, In-Process & Other Non-Indexed Citations, Daily and Versions(R) <1946 to May 15, 2019> Searched 16th May 2019

1 (immuniz* or immunis* or vaccin* or inoculat* or innoculat* or immunotherap* or prophyla*).ti,ab,kw. (672732)

2 immunization/ or immunization, passive/ or immunization schedule/ or immunization, secondary/ or immunotherapy, active/ or vaccination/ or Immunization Programs/ or mass vaccination/ (158163)

3 Tuberculosis Vaccines/ or BCG Vaccine/ or Diphtheria-Tetanus Vaccine/ or Meningococcal Vaccines/ or Pertussis Vaccine/ or Diphtheria-Tetanus-acellular Pertussis Vaccines/ or Diphtheria-Tetanus-Pertussis Vaccine/ or Diphtheria-Tetanus Vaccine/ or Measles Vaccine/ or Mumps Vaccine/ or Rubella Vaccine/ or Measles-Mumps-Rubella Vaccine/ or Poliovirus Vaccines/ or Poliovirus Vaccine, Inactivated/ or Poliovirus Vaccine, Oral/ or Japanese Encephalitis Vaccines/ or Rotavirus Vaccine/ (49639)

4 or/1-3 (720583)

5 developing countries.sh,kf. (83271)

6 (Africa or Asia or Caribbean or West Indies or South America or Latin America or Central America).ti,ab,kw. (200981)

7 Africa/ or Asia/ or Caribbean/ or West Indies/ or South America/ or Latin America/ or Central America/ (73389)

8 (Africa or Central America or South America or Caribbean or Central Asia or Afghanistan or Albania or Algeria or Angola or Argentina or Armenia or Armenian or Azerbaijan or Bangladesh or Benin or Byelarus or Byelorussian or Belarus or Belorussian or Belorussia or Belize or Bhutan or Bolivia or Bosnia or Herzegovina or Hercegovina or Botswana or Brazil or Bulgaria or Burkina Faso or Burkina Fasso or Upper Volta or Burundi or Urundi or Cambodia or Khmer Republic or Kampuchea or Cameroon or Cameroons or Cameron or Camerons or Cape Verde or Cabo Verde or Central African Republic or Chad or Tchad or China or Colombia or Comoros or

For peer review only

Comoro Islands or Comores or Mayotte or Congo or Zaire or Costa Rica or Cote d'Ivoire or Ivory Coast or Cuba or Djibouti or French Somaliland or Dominica or Dominican Republic or East Timor or East Timur or Timor Leste or Ecuador or Egypt or United Arab Republic or El Salvador or Eritrea or Ethiopia or Fiji or Gabon or Gabonese Republic or Gambia or Gaza or Georgia Republic or Georgian Republic or Ghana or Grenada or Guatemala or Guinea or Guiana or Guyana or Haiti or Honduras or India or Maldives or Indonesia or Iran or Iraq or Jamaica or Jordan or Kazakhstan or Kazakh or Kenya or Kiribati or Korea or Kosovo or Kyrgyzstan or Kirghizia or Kyrgyz Republic or Kirghiz or Kirgizstan or Lao PDR or Laos or Lebanon or Lesotho or Basutoland or Liberia or Libya or Macedonia or Madagascar or Malagasy Republic or Malaysia or Malaya or Malay or Sabah or Sarawak or Malawi or Mali or Marshall Islands or Mauritania or Mauritius or Agalega Islands or Mexico or Micronesia or Middle East or Moldova or Moldovia or Moldovian or Mongolia or Montenegro or Morocco or Ifni or Mozambique or Myanmar or Myanma or Burma or Namibia or Nepal or Netherlands Antilles or Nicaragua or Niger or Nigeria or Muscat or Pakistan or Palau or Palestine or Panama or Paraguay or Peru or Philippines or Philipines or Phillipines or Phillippines or Papua New Guinea or Romania or Rumania or Roumania or Rwanda or Ruanda or Saint Lucia or St Lucia or Saint Vincent or St Vincent or Grenadines or Samoa or Samoan Islands or Navigator Island or Navigator Islands or Sao Tome or Senegal or Serbia or Montenegro or Seychelles or Sierra Leone or Sri Lanka or Solomon Islands or Somalia or Sudan or Suriname or Surinam or Swaziland or Eswatini or South Africa or Syria or Tajikistan or Tadzhikistan or Tadjikistan or Tadzhik or Tanzania or Thailand or Togo or Togolese Republic or Tonga or Tunisia or Turkey or Turkmenistan or Turkmen or Uganda or Ukraine or Uzbekistan or Uzbek or Vanuatu or New Hebrides or Venezuela or Vietnam or Viet Nam or West Bank or Yemen or Zambia or Zimbabwe or Rhodesia).ti,ab,kw,sh. (1387951)
9 ((developing or less* developed or least developed or under developed or underdeveloped or middle income or low* income or underserved or under served or deprived or poor* or resource limited or resource constrained) adj (countr* or nation? or population? or world or state*)).ti,ab,kw. (91955)
10 ((developing or less* developed or least developed or under developed or underdeveloped or middle income or low* income or resource limited or resource constrained) adj (economy or economies)).ti,ab,kw. (504)
11 (low* adj (gdp or gnp or gross domestic or gross national)).ti,ab,kw. (234)
12 (low adj3 middle adj3 countr*).ti,ab,kw. (12819)
13 (lmic or lmics or third world or lami countr*).ti,ab,kw. (6486)
14 (transitional countr* or emerging econom* or global south).ti,ab,kw. (873)
15 or/5-14 (1487811)
16 4 and 15 (80257)
17 Parents/ or Fathers/ or Mothers/ or Grandparents/ or Caregivers/ or Single Parent/ or Pregnant Women/ or Child, Preschool/ or Infant/ or Infant, Newborn/ or Infant, Low Birth Weight/ or Infant, Small for Gestational Age/ or Infant, Very Low Birth Weight/ or Infant, Extremely Low Birth Weight/ or Infant, Postmature/ or Infant, Premature/ or Infant, Extremely Premature/ (1597959)
18 (child* or infant* or newborn* or neonat* or neo nat* or prenatal or pre natal or ante natal or antenatal or baby or babies or toddler* or preschool* or parent* or mother* or father* or maternal or paternal).ti,ab,kw. (2284298)
19 or/17-18 (2893768)
20 16 and 19 (28515)
21 (random* or experiment* or (match* adj2 (propensity or coarsened or covariate)) or "propensity score" or ("difference in difference*" or "difference-in-difference*" or "differences in difference*" or "differences-in-difference*" or "double difference*") or ("quasi-experimental" or "quasi experimental" or "quasi-experiment" or "quasi experiment") or ((estimator or counterfactual) and evaluation*) or "instrumental variable*" or (IV adj2 (estimation or approach)) or regression discontinuity or time series or segment* regression).ti,ab,kw. (2983909)
22 Randomized Controlled Trial/ or Random Allocation/ or Evaluation Studies/ or Propensity Score/ or Interrupted Time Series Analysis/ or Controlled Before-After Studies/ or Controlled Clinical Trial/ or Non-Randomized Controlled Trials as Topic/ (896249)
23 or/21-22 (3415351)
24 Cost Analysis/ or Cost-Benefit Analysis/ or Quality-Adjusted Life Years/ or Economics, Medical/ or Cost of Illness/ or Health Care Costs/ or Direct Service Costs/ or Budgets/ or Health Care Sector/ or Public Expenditures/ (193420)
25 (cost-effective* or cost-benefit).ti,ab,kw. (130711)
26 ("life year" or "life years" or qaly* or daly*).ti,ab,kw. (18749)
27 ((economic* or cost*) adj6 (mortality or death* or markov)).ti,ab,kw. (17246)
28 ("cost minimi*" or "cost-utilit*" or "economic evaluation*" or "economic review*" or "cost outcome" or "cost analys*" or "economic analys*" or "budget* impact analys*").ti,ab,kw. (27594)
29 or/24-28 (301485)
30 (review or meta-analysis).pt. (2557796)
31 meta-analysis/ or "systematic review"/ (163880)
32 cochrane database of systematic reviews.jn. (14162)
33 (systematic review or literature review).ti. (130870)
34 or/30-33 (2590211)

35	23 or 29 or 34 (5946083)
36	20 and 35 (8309)
37	exp Animals/ (22312530)
38	Humans/ (17732234)
39	37 not (37 and 38) (4580296)
40	36 not 39 (7984)

For peer review only

Appendix 5: Data extraction template

1) General Information

Sr. Number	
Study ID	
Short title	
Minutes taken to complete data extraction for this study	
Who's completing this form?	
Study title	
Year of publication	
Publication type	Journal article
	Published report
	Published working paper
	Thesis
Journal name/ publisher	
Journal Volume	
Journal Issue	
Research funding agency category	Academic institutions
	Charitable or private foundations
	Government agencies
	International aid agencies
	International financial institutions
	Non-profit organisation
	Not specified
Research funding agency name	
Included geographies	Sub-Saharan Africa
	South Asia
	East Asia and Pacific
	Latin America & the Caribbean
	Middle East & North Africa
	Eastern Europe and Central Asia
	North America
	Western Europe
	Global
	Unclear / unspecified
	If the review focuses on only one country, specify it
Location of intervention	Urban areas
	Rural areas
	Urban and rural areas
	Not reported
Any other relevant information you'd like to add for this section?	
Overarching main research question or primary hypothesis	
Includable comparators	No intervention
	"Business as usual" (existing ongoing treatment)
	Alternative intervention
	Placebo intervention
	No information on includable comparators provided
Search and inclusion	Have academic databases been searched?
	Have grey literature sites been searched?
	Dates of search reported?
	Was the review part of the update?
	PRISMA flow diagram presented?
	Number of included studies
	List of excluded studies provided?
	Explanations for exclusions provided?
Types of studies included, by study design	Quantitative studies included
	Qualitative studies included
	Mixed-methods studies included
	Unclear or information not provided
	Experimental design / RCT

For peer review only

Types of quantitative study designs included	Pipeline design
	Panel design or before/after AND with/without
	Either before/after OR with/without
	Natural experiment
	Basic survey
	Unclear or information not provided
Types of qualitative study designs included	Ethnographic study design
	Semi-structured interviews
	Narrative or life history
	Visual and/or participatory methods
	Documentary and/or content analysis
	Focus group discussions
	Unclear or information not provided
Types of quantitative analysis techniques applied in included studies	Econometric techniques (IV, PSM, 2SLS, DID, RDD, etc.)
	Multivariable analysis (OLS / regression-based approaches)
	Cross-tabulation / descriptive statistics
	Unclear or information not provided
Types of qualitative analysis techniques applied in included studies	Content or framework analysis
	Grounded theory or IPA
	Discourse analysis
	Narrative analysis
	Thematic analysis
	Unclear or information not provided
	Any other relevant information you'd like to add for this section?
What type of QUANTITATIVE data synthesis mechanisms were applied in the systematic review/meta-analysis?	Meta-regression
	Meta-analysis
	Description of range of effect sizes
	Vote counting - statistical significance
	Vote counting - direction of effect
	Descriptive (usually for narrative synthesis)
	Unclear or information not provided
What type of QUALITATIVE data synthesis mechanisms were applied in the systematic review/meta-analysis?	Narrative synthesis
	Thematic synthesis
	Meta-ethnography
	Context-mechanism analysis
	Qualitative comparative analysis
	Unclear or information not provided
Subgroup analysis	Was subgroup analysis conducted?
	Please briefly describe the nature of the subgroup analysis
Publication bias and funnel	Did the authors include a discussion of publication bias?
	Were funnel plots reported?
	Were Funnel Asymmetry and Precision Effects tests reported?
	Did the FAT-PET show evidence of publication bias? (Specific test for meta-regression)
	Any other relevant information you'd like to add for this section?
Other	Does the systematic review/meta-analysis provide a GRADE rating?
	What are the quality assessment tool(s) / risk of bias procedure in the study?
	Has the cost effectiveness analysis of the intervention(s) been included?
	If yes, what was the main cost measure that was used/calculated? (e.g., total intervention costs, intervention costs per person, incremental costs, incremental cost-effectiveness ratios (ICER) etc.)
	Did the study do sensitivity analysis?
	If yes, describe the kind of sensitivity analysis done. If no, what was the reason if acknowledged.
	Limitations of the systematic review/meta-analysis acknowledged by the authors

2) Outcome data

Intervention Code (from immunisation EGM)

For peer review only

Intervention description
Intervention notes
Outcome code (from immunisation EGM)
Outcome description
Outcome notes
Further information about outcome classification
Unit of measurement
Sample size (number of studies and number of participants)
Population level analysed
Sub-population(s) analysed
Direction of outcome (favourable, unfavourable, inconclusive)
Are meta-analysis regression results presented for this outcome?
How many effect sizes are reported for this outcome?
Summary of the key conclusions drawn
Narrative or other type of synthesis trend result
Narrative or other type of synthesis summary quote
Any other relevant information you'd like to add for this section?
Grade rating
Source (page number)

3) Meta-analysis data

Quantitative data extraction
Intervention Code (from immunisation EGM)
Outcome code (from immunisation EGM)
Dependent variable name for the effect size (exact terminology as listed in the systematic review/meta-analysis)
Type of effect size reported
Effect size (the actual figure)
Significance level
Confidence interval (95%)
Sample size (number of studies)
Heterogeneity
Did the authors describe likely explanatory factors of heterogeneity?
If so, please note the key factors put forward
Was a sensible method used to explore the extent to which key factors explained heterogeneity?
If so, which
If applicable, mention the test for heterogeneity
If applicable, provide heterogeneity statistics
If applicable, provide confidence interval of heterogeneity statistics
If meta-analysis is done by subgroup, then describe it
Effect size by subgroup, actual figure
Confidence interval of effect size by subgroup
Source (page number, table number, etc.)
Additional notes

For peer review only

Appendix 6: Quality appraisal tool

Checklist for making judgements about how much confidence to place in a systematic review of effects (adapted version of SURE checklist)

Assessed by:	
Date:	

Section A: *Methods used to identify, include and critically appraise studies*

A.1 Were the criteria used for deciding which studies to include in the review reported?<br>Did the authors specify:<br>□ Did the authors specify Types of studies?<br>□ Did the authors specify Participants/ settings/ population?<br>□ Did the authors specify Intervention(s)?<br>□ Did the authors specify Outcome(s)?	□ Yes<br>□ Partially<br>□ No<br>*Coding guide - check the answers above*<br>*YES: All four should be yes*<br>*NO: All four should be no*<br>*PARTIALLY: Any other*

For peer review only

A.2 Was the search for evidence reasonably comprehensive?<br>Were the following done:<br>□ Language bias avoided (no restriction of inclusion based on language)<br>□ No restriction of inclusion based on publication status<br>□ Relevant databases searched<br><u>Minimum criteria</u>: at least one database that includes grey/unpublished literature, as well as either: (a) for health, at least two relevant comprehensive subject databases (such as PubMed/MEDLINE, EMBASE and CENTRAL), or (b) for social sciences, at least two relevant comprehensive subject databases (such as IDEAS) and one comprehensive general database (such as EconLit, PsychInfo, Scopus. If the search is for studies in China, the CNKI (China National Knowledge infrastructure) is acceptable as a comprehensive database.<br>□ Reference lists in included articles checked<br>□ Authors/experts contacted	□ Yes<br>□ Partially<br>□ No<br>□ Can't tell<br>*Coding guide - check the answers above:*<br>*YES: All five should be yes*<br>*PARTIALLY: Relevant databases and reference lists are both reported*<br>*NO: Any other*<br>Note: Grey literature typically means research that is not published in sources such as books or journal articles. The following databases include grey literature: Academic Search Complete (includes many conference proceedings), CAB Abstracts, search conducted using CADATH checklist, clinicaltrials.gov, Cochrane Central Register of Controlled Trials (CENTRAL), Cochrane Library, Embase (includes 3.6m+ conference abstracts), Google, Google Scholar, Healthcare Management Information Consortium (HMIC), IDEAS/RePEc, National Technical Information Service (NTIS), OpenSIGLE/OpenGrey, PsycEXTRA, Scopus (includes ~10m conference papers). If you identify additional sources, please add to the list. Searching websites of relevant governmental agencies and non-governmental organisations or other organisations such as the WHO can also identify grey literature. Note that MEDLINE/PubMed, a comprehensive data base of journals, does not include grey literature: "For indexing in MEDLINE, NLM currently selects publications that it considers to be journals."; see also Citrome L. Beyond PubMed: Searching the "Grey Literature" for Clinical Trial Results. Innov Clin Neurosci. 2014;11(7-8):42-46.

For peer review only

A.3 Does the review cover an appropriate time period?<br>*Is the search period comprehensive enough that relevant literature is unlikely to be omitted?*	□ Yes<br>□ Can't tell<br>□ No<br>*Coding guide:*<br>*YES: Generally, this means searching the literature at least back to 1990*<br>*NO: Generally, if the search does not go back to 1990*<br>*CAN'T TELL: No information about time period for search*<br>*Note: With reference to the above – there may be important reasons for adopting different dates for the search, e.g., depending on the intervention. If you think there are limitations with the timeframe adopted for the search which have not been noted and justified by the authors, you should code this item as a NO and specify your reason for doing so in the comment box below. Older reviews should not be downgraded, but the fact that the search was conducted some time ago should be noted in the quality assessment. Always report the time period for the search in the comment box. If the authors do not report the search period, check the publication date of the earliest included study. If the study was published before 1990 this can be coded Yes.*
A.4 Was bias in the selection of articles avoided?<br>Did the authors specify:<br>□ Independent screening of full text by at least 2 reviewers<br>□ List of included studies provided<br>□ List of excluded studies provided	□ Yes<br>□ Partially<br>□ No<br>*Coding guide:*<br>*YES: All three should be yes, although reviews published in journals are unlikely to have a list of excluded studies (due to limits on word count) and the review should not be penalised for this.*<br>*PARTIALLY: Independent screening and list of included studies provided are both reported*<br>*NO: All other. If list of included studies provided, but the authors do not report whether or not the screening has been done by 2 reviewers review is downgraded to NO.*<br>*Note: The answer linked to list of included studies provided criteria is from the question "Was a table or summary of the results of the included studies provided?" in the data extraction form.*

For peer review

A.5 Did the authors use appropriate criteria to assess the quality and risk of bias in analysing the studies that are included?<br>□ The criteria used for assessing the quality/ risk of bias were reported<br>□ A table or summary of the assessment of each included study for each criterion was reported<br>□ Sensible criteria were used that focus on the quality/ risk of bias (and not other qualities of the studies, such as precision or applicability/external validity).<br>*"Sensible" is defined as a recognised quality appraisal tool/ checklist, or similar tool which assesses bias in included studies. Please see footnotes for details of the main types of bias such a tool should assess.*<br>*Identified tools with sensible criteria include: Academy of Nutrition and Dietetics Quality Criteria Checklist, Child Health Epidemiology Reference Group (CHERG) study design & quality standards , Cochrane Handbook, The Delphi List, Effective Public Health Practice Project (EPHPP) Quality Assessment Tool, Grading of Recommendations Assessment, Development and Evaluation (GRADE) RoB criteria, Guide to Community Preventative Services Study Quality tool, Joanna Briggs Institute Checklists for RCT/QED, National Institutes of Health's Quality Assessment Tool for Controlled Intervention Studies (sometimes labelled NHLBI tool). For case-control studies and cohort studies, the Newcastle-Ottawa Scale uses sensible criteria that are focused on risk of bias as does Methodological Index for Non-Randomised Studies (MINORS). Note that these designs typically are not as rigorous as RCTs or even QEDs.*	□ Yes<br>□ Partially<br>□ No<br>*Coding guide:*<br>*YES: All three should be yes*<br>*PARTIALLY: The first and third criteria should be reported. If the authors report the criteria for assessing risk of bias and report a summary of this assessment for each criterion, but the criteria may be only partially sensible (e.g., do not address all possible risks of bias, but do address some), we downgrade to PARTIALLY.*<br>*NO: Any other*
A.6 Overall – how much confidence do you have in the methods used to identify, include and critically appraise studies?<br>*Summary assessment score A relates to the 5 questions above.*<br>*High confidence applicable when the answers to the questions in section A are all assessed as 'yes'*<br>*Low confidence applicable when any of the following are assessed as 'NO' above: not reporting explicit selection criteria (A1), not conducting reasonably comprehensive search (A2), not avoiding bias in selection of articles (A4), not assessing the risk of bias in included studies (A5)*<br>*Medium confidence applicable for any other – i.e. section A3 is assessed as 'NO' or can't tell and remaining sections are assessed as 'partially' or 'can't tell'*	□ Low confidence (limitations are important enough that the results of the review are not reliable)<br>□ Medium confidence (limitations are important enough that it would be worthwhile to search for another systematic review and to interpret the results of this review cautiously, if a better review cannot be found)<br>□ High confidence (only minor limitations)

Section B: *Methods used to analyse the findings*

For peer review only

B.1 Were the characteristics and results of the included studies reliably reported? Was there:<br>□ Independent data extraction by at least 2 reviewers<br>□ A table or summary of the characteristics of the participants, interventions and outcomes for the included studies (Forest plots are an appropriate summary of the results, as is reporting summarises the findings by outcome domain.)<br>□ A table or summary of the results of all the included studies	□ Yes<br>□ No<br>□ Partially<br>□ Not applicable (e.g., no included studies)<br>*Coding guide:*<br>*YES: All three should be yes*<br>*PARTIALLY: Criteria one and three are yes, but some information is lacking on second criteria.*<br>*No: None of these are reported. If the review does not report whether data was independently extracted by 2 reviewers (possibly a reporting error), we downgrade to NO.*<br>*NOT APPLICABLE: if no studies/no data*<br>*Note: Independent extraction means that both extractors extracted all data without knowing what the other extractor decided (that is, one extractor and one verifier does not meet criterion). If the authors note two reviewers and do not use the word "independent" but mention a third reconciler to resolve differences, assume independence. When authors do not mention whether independent extraction was conducted by at least two reviewers, code No.*
B.2 Are the methods used by the review authors to analyse the findings of the included studies clear, including methods for calculating effect sizes if applicable?	□ Yes<br>□ Partially<br>□ No<br>□ Not applicable (e.g., no studies or no data)<br>*Coding guide:*<br>*YES: Methods used clearly reported. If it is clear that the authors use narrative synthesis, they don't need to say this explicitly.*<br>*PARTIALLY: Some reporting on methods but lack of clarity*<br>*NO: Nothing reported on methods*<br>*NOT APPLICABLE: if no studies/no data*

For peer review only

B.3 Did the review describe the extent of heterogeneity?<br>□ Did the review ensure that included studies were similar enough that it made sense to combine them, sensibly divide the included studies into homogeneous groups, or sensibly conclude that it did not make sense to combine or group the included studies?<br>□ Did the review discuss the extent to which there were important differences in the results of the included studies?<br>□ If a meta-analysis was done, was the I^2, chi square test for heterogeneity or other appropriate statistic reported? If no statistical test was reported, is a qualitative justification made for the use of random effects?	□ Yes<br>□ Partially<br>□ No<br>□ Not applicable (e.g., no studies or no data)<br><br>*Coding guide:*<br>*YES: First two should be yes, and third category should be yes if applicable should be yes*<br>*PARTIALLY: The first category is yes*<br>*NO: Any other*<br>*NOT APPLICABLE: if no studies/no data*<br>*Note: Code B3.1 No if analyses include studies with implausibly different interventions, comparisons, or populations. If a narrative synthesis, the authors need to have a rationale for why studies were not combined (such as interventions were too different) For meta-analyses, reporting a metric for heterogeneity is sufficient for B3.2. For non-meta-analysis, mentioning heterogeneity in results is enough (for example, The impacts varied from X to Y or Study A found X and Study B found Y).*

For peer review only

B.4 Were the findings of the relevant studies combined (or not combined) appropriately relative to the primary question the review addresses and the available data?<br>How was the data analysis done?<br>□ Descriptive only<br>□ Vote counting based on direction of effect<br>□ Vote counting based on statistical significance<br>□ Description of range of effect sizes<br>□ Meta-analysis<br>□ Meta-regression<br>□ Other: specify<br>□ Not applicable (e.g., no studies or no data)<br>How were the studies weighted in the analysis?<br>□ Equal weights (this is what is done when vote counting is used)<br>□ By quality or study design (this is rarely done)<br>□ Inverse variance (this is what is typically done in a meta-analysis)<br>□ Number of participants (sample size)<br>□ Other: specify<br>□ Not clear<br>□ Not applicable (e.g., no studies or no data)<br>Did the review address unit of analysis errors?<br>□ Yes - took clustering into account in the analysis (e.g., used intra-cluster correlation coefficient)<br>□ No, but acknowledged problem of unit of analysis errors<br>□ No mention of issue<br>□ Not applicable - no clustered trials or studies included	□ Yes<br>□ Partially<br>□ No<br>□ Not applicable (e.g., no studies or no data)<br>□ Can't tell<br>*Coding guide:*<br>*YES: If appropriate table, graph or meta-analysis AND appropriate weights AND unit of analysis errors addressed (if appropriate).*<br>*PARTIALLY: If appropriate table, graph or meta-analysis AND appropriate weights AND unit of analysis errors not addressed (and should have been).*<br>*NO: If narrative OR vote counting (where quantitative analyses would have been possible) OR inappropriate reporting of table, graph or meta-analyses.*<br>*NOT APPLICABLE: if no studies/no data*<br>*CAN'T TELL: if unsure (note reasons in comments below)*

For peer review only

B. 5 Does the review report evidence appropriately?<br>□ The review makes clear which evidence is subject to low risk of bias in assessing causality (attribution of outcomes to intervention), and which is likely to be biased, and does so appropriately<br>□ Where studies of differing risk of bias are included, results are reported and analysed separately by risk of bias status (No)	□ Yes<br>□ No<br>□ Partially<br>□ Not applicable<br>*Coding guide:*<br>*YES: Both criteria should be fulfilled (where applicable)*<br>*NO: Criteria not fulfilled*<br>*PARTIALLY: Only one criterion fulfilled, or when there is limited reporting of quality appraisal (the latter applies only when inclusion criteria for study design are appropriate)*<br>*NOT APPLICABLE: No included studies*<br>*Note on reporting evidence and risk of bias: For reviews of effects of 'large n' interventions, experimental and quasi-experimental designs should be included (if available). For reviews of effects of 'small n' interventions, designs appropriate to attribute changes to the intervention should be included (e.g., pre-post with assessment of confounders)*<br>*Making clear which evidence is subject to low risk of bias can be accomplished in a table listing RoB for each study or by listing RoB for each study on each RoB criterion; that is, if A5.2 is Yes, then B5.1 is Yes (but the reverse is not true). Reporting only study design is not sufficient to meet B5.1. For B5.2, narrative analysis must group or report by RoB, it is not sufficient to simply report RoB of each study. If the* systematic review *does not use sensible criteria to assess RoB, then B5.1 is No.*
B.6 Did the review examine the extent to which specific factors might explain differences in the results of the included studies?<br>□ Were factors that the review authors considered as likely explanatory factors clearly described?<br>□ Was a sensible method used to explore the extent to which key factors explained heterogeneity?<br>□ Descriptive/textual<br>□ Graphical<br>□ Meta-analysis by subgroups<br>□ Meta-regression<br>□ Other	□ Yes<br>□ Partially<br>□ No<br>□ Not applicable<br>*Coding guide:*<br>*YES: Explanatory factors clearly described and appropriate methods used to explore heterogeneity*<br>*PARTIALLY: Explanatory factors described but for meta-analyses, subgroup analysis or meta-regression not reported (when they should have been)*<br>*NO: No description or analysis of likely explanatory factors*<br>*NOT APPLICABLE: e.g., too few studies, no important differences in the results of the included studies, or the included studies were so dissimilar that it would not make sense to explore heterogeneity of the results*

For peer review only

B.7 Overall - how much confidence do you have in the methods used to analyse the findings relative to the primary question addressed in the review?<br>*Summary assessment score B relates to the 5 questions in this section, regarding the analysis.*<br>*High confidence applicable when all the answers to the questions in section B are assessed as 'yes'.*<br>*Low confidence applicable when any of the following are assessed as 'NO' above: critical characteristics of the included studies not reported (B1), not describing the extent of heterogeneity (B3), combining results inappropriately (B4), reporting evidence inappropriately (B5).*<br>*Medium confidence applicable for any other: i.e., the "Partial" option is used for any of the 6 preceding questions or questions and/or B.2 and/ or B.6 are assessed as 'no'.*	□ Low confidence (limitations are important enough that the results of the review are not reliable)<br>□ Medium confidence (limitations are important enough that it would be worthwhile to search for another systematic review and to interpret the results of this review cautiously, if a better review cannot be found)<br>□ High confidence (only minor limitations)

For peer review only

Section C: Overall assessment of the reliability of the review

C.1 Are there any other aspects of the review not mentioned before which lead you to question the results?	□ Additional methodological concerns – only one person reviewing<br>□ Robustness<br>□ Interpretation<br>□ Conflicts of interest (of the review authors or for included studies)<br>□ Other<br>□ No other quality issues identified
C.2 Are there any mitigating factors which should be taken into account in determining the reviews reliability?	□ Limitations acknowledged<br>□ No strong policy conclusions drawn (including in abstract/ summary)<br>□ Any other factors
C.3 Based on the above assessments of the methods how would you rate the reliability of the review?<br>□ <u>Low confidence in conclusions about effects</u>:<br>This review differs from the typical effectiveness review in that it combines evaluations with descriptive studies, and has no methodological exclusion criteria. Moreover, the authors do not perform any sort of risk of bias assessment on the evaluative studies, nor clearly report the methods used in included studies, making it difficult to know how much confidence to place in the results. There are major gaps in the reporting of the search (databases not listed) and screening (no discussion of how this was done). The authors do note the limitations of the review, including the language restrictions and the limited reporting on pre- and post-intervention coverage rates in the included studies.<br>□ <u>Medium confidence in conclusions about effects</u>:<br>The systematic review has the following limitations.<br>□ <u>High confidence in conclusions about effects</u>:<br>If applicable: The review has the following minor limitations...<br>*Coding guide:*<br>High confidence in conclusions about effects: high confidence noted overall for sections A and B, unless moderated by answer to C1.<br>Medium confidence in conclusions about effects: medium confidence noted overall for sections A or B, unless moderated by answer to C1 or C2.<br>Low confidence in conclusions about effects: low confidence noted overall for sections A or B, unless moderated by answer to C1 or C2.<br>Limitations should be summarised above, based on what was noted in Sections A, B and C.	

Section D

D.1. Does the review use a programme theory?<br>□ Did the authors present: A logic model articulating the intervention causal chain from inputs/activities through to outcomes?<br>□ Did the authors present: Assumptions, either in the logic model itself or discussed in supporting text?<br>□ Did the authors present: A (middle-range) theory (economic theory, e.g. trade theory; social theory, e.g. diffusion theory; etc.) which informed the logic models and/or from which inferences can be made about mechanisms and contexts under which outcomes might occur?	□ Yes<br>□ Partially<br>□ No<br>*Coding guide:*<br>*YES: Some theory is used, whether an intervention level logic model or causal chain, or formal theory, and underlying assumptions are explicitly described.*<br>*NO: None are reported.*<br>*PARTIALLY: A theory is used but underlying assumptions are not reported.*

For peer review only

D.2. Does the review incorporate qualitative evidence in the design?<br>□ Was the logic model or theory articulated at protocol stage?<br>□ Is qualitative evidence cited in the development or explanation of the theoretical approach/logic model?<br>□ Is qualitative evidence incorporated systematically (i.e., based on systematic searches), in order to develop the theoretical approach/logic model?<br>□ Is qualitative evidence cited in development of relevant questions?	□ Yes<br>□ Partially<br>□ No<br>*Coding guide:*<br>*YES: At least 1 and 2 or 3 are reported.*<br>*NO: None are reported.*<br>*PARTIALLY: 1 or 4 are reported.*
D.3. Did the review conduct analysis of intermediate and endpoint outcomes along causal chain?<br>□ Did the review conduct analysis of primary endpoint outcomes?<br>□ Did the review conduct analysis of primary intermediate outcomes?<br>□ Did the review conduct analysis of secondary endpoint outcomes?<br>□ Did the review conduct analysis of secondary intermediate outcomes?	□ Yes<br>□ Partially<br>□ No<br>□ Not applicable<br>□ Can't tell<br>*Coding guide:*<br>*YES: Boxes 1 and 2 are ticked*<br>*PARTIALLY: Boxes 1 and 4 or 2 and 3 are ticked.*<br>*NO: analysis of outcomes along causal chain is not undertaken and only endpoint outcomes are analysed (and outcomes at different stages of the causal chain were excluded).*<br>*NOT APPLICABLE: if no studies/no data*<br>*CAN'T TELL: if unsure (note reasons in comments below)*
D.4. Does the review incorporate qualitative evidence in the analysis?<br>□ To answer specific review questions about intervention design (e.g., project portfolio information)<br>□ To answer specific review questions about barriers and facilitators/enablers of implementation (assumptions or risks in the causal chain/ logic model)<br>□ To answer specific review questions about adherence or participant views?<br>□ To provide information on context for included quantitative studies (e.g., moderators or implementation fidelity)?<br>□ To provide evidence on 'middle-range' causal mechanisms or contextual factors (e.g., policy context, second order changes e.g., general equilibrium effects, sustained adherence)	□ Yes<br>□ Partially<br>□ No<br>*Coding guide:*<br>*YES: 1, 2, or 3 plus 4 or 5 are reported.*<br>*NO: None are reported.*<br>*PARTIALLY: Any other combination.*

D.5. Does the review incorporate qualitative evidence in other aspects of the analysis?<br>□ To assess applicability of synthesised evidence to particular contexts?<br>□ To discuss the quantitative findings in light of qualitative evidence, drawing on qualitative evidence collected systematically?<br>□ To discuss the quantitative findings in light of qualitative evidence (i.e., in the discussion section only), not drawing on evidence collected systematically?	□ Yes<br>□ Partially<br>□ No<br>*Coding guide:*<br>*YES: 1 or 2 are reported.*<br>*NO: None are reported.*<br>*PARTIALLY: 3 is reported.*
D.6. Does the review integrate the findings from quantitative and qualitative evidence?<br>□ Is qualitative evidence: Presented in a separate results section?<br>□ Is qualitative evidence: Integrated with evidence on effects using qualitative methods (e.g., further iterations of the theory of change)<br>□ Is qualitative evidence: Integrated with evidence on effects using quantitative methods (e.g. through formal statistical testing of moderators and subgroups identified in qualitative analysis)<br>□ Is qualitative evidence: Some other method to integrate the evidence? (specify)	□ Yes<br>□ Partially<br>□ No<br>*Coding guide:*<br>*YES: 1 and 2 or 3 are reported.*<br>*NO: None are reported.*<br>*PARTIALLY: 1 is reported only.*
D.7. Is quantitative and qualitative evidence integrated to form conclusions and implications?<br>□ Weight of quantitative evidence assessed using GRADE or other methods<br>□ Weight of qualitative evidence assessed using cerQUAL or other methods<br>□ Some method is used to integrate findings from quantitative and qualitative approaches (e.g. summary of findings table) to determine conclusions and implications?	□ Yes<br>□ Partially<br>□ No<br>*Coding guide:*<br>*YES: All are reported.*<br>*NO: None are reported.*<br>*PARTIALLY: Only 1, 2 or 3 are reported.*
Overall - how much confidence do you have in the causal chain used in the review to analyse studies and the type of evidence incorporated to inform the analysis and reporting?	□ Low confidence<br>□ Medium confidence<br>□ High confidence

For peer review only

Notes:

i. Adapted from the Specialist Unit for Review Evidence (SURE) 2018. Questions to assist with the critical appraisal of systematic reviews available at: https://www.cardiff.ac.uk/__data/assets/pdf_file/0007/1142962/SURE-CA-form-for-SR_2018.pdf

ii. **Risk of bias** is the extent to which bias may be responsible for the findings of a study.

Bias is a systematic error or deviation from the truth in results or inferences. In studies of the effects of social, economic and health care interventions, the main types of bias arise from systematic differences in the groups that are compared (selection bias), the intervention that is provided, or exposure to other factors apart from the intervention of interest (performance bias/contamination), withdrawals or exclusions of people entered into a study (attrition bias) or how outcomes are assessed (detection bias) and reported (reporting bias). Reviews of social science studies may be particularly affected by reporting bias, where a biased subset of all the relevant data and analyses is presented.

Assessments of the risk of bias are sometimes also referred to as assessments of the **validity** or **quality** of a study.

Validity is the extent to which a result (of a measurement or study) is likely to be true.

Quality is a vague notion of the strength or validity of a study, often indicating the extent of control over bias.

For peer review only

Appendix 7: Summary of included systematic reviews

Table 6.1. Moderate and high confidence systematic reviews

Authors & Year	Source	Type of review	Geographic focus		No. primary studies	Types of intervention included	Types of outcomes included	Population level analysed
			No. of regions	Regions				
High confidence reviews (18 in total)								
Diaconu et al., 2021[52]	Journal article	Systematic review	4	Sub-Saharan Africa, South Asia, East Asia and Pacific, Latin America & the Caribbean	59	BD5. Pay-for-performance schemes	JAA15. Vaccination coverage (unspecified); JAA01. Full routine immunisation for children; JAA02. BCG; JAA05. DPT3; JAA11. Measles; JAA09. OPV3; IAA1. Community HW motivation, capacity & performance; ICA1. Stockouts; HAD4. Experience and satisfaction with health services	Child; Patients; Health facility; Healthcare workers
Eze, Lawani, & Acharya, 2021[44]	Journal article	Systematic review & Meta-analysis	4	Sub-Saharan Africa, South Asia, East Asia and Pacific, Latin America & the Caribbean	18	AB4. Written or pictorial messages (SMS, stickers, flyers etc.) to caregivers	JAA15. Vaccination coverage (unspecified); JAA13. Vaccination. Timeliness	Child
Gera et al., 2016[53]	Journal article	Systematic review & Meta-analysis	2	Sub-Saharan Africa, South Asia	4	BA1. Formal health worker training and education; BF1. Health system strategic planning	JAA11. Measles; JAA05. DPT3	Child
Glenton et al., 2011[54]	Journal article	Systematic review & Meta-analysis	7	Sub-Saharan Africa, South Asia, East Asia and Pacific, Latin America & the Caribbean, Eastern Europe and Central Asia, North America, Western Europe	12	AA2. Short-term sensitization and education campaigns; BB7. Home visits; BB4. Promoting outreach to vaccine-hesitant groups; BC1. National/sub-national immunisation days; BB7. Home visits	JAA01. Full routine immunisation for children	Child

For peer review only

Authors & Year	Source	Type of review	Geographic focus		No. primary studies	Types of intervention included	Types of outcomes included	Population level analysed
			No. of regions	Regions				
Jain et al., 2022[41, 42][4]	Journal article	Systematic review & Meta-analysis	3	Sub-Saharan Africa, South Asia, Latin America & the Caribbean	61	F. Multicomponent; AB1. Material/ monetary incentives for caregivers; AB4. Written or pictorial messages (SMS, stickers, flyers etc.) to caregivers; CA1. Faith-based outreach/outreach using local leaders; DA1. Collaborating with whole community; BB5. Outreach to vulnerable populations (hard-to-reach, SES, caste, etc.); DA1. Collaborating with whole community; BD5. Pay-for-performance schemes; BF1. Health system strategic planning	JAA01. Full routine immunisation for children; JAA05. DPT3; JAA11. Measles; JAA13. Vaccination. Timeliness; JAA12. No vs. partial routine immunisation; JAA02. BCG; JAA03. DPT1; JAA04. DPT2; JAA06. OPV0; JAA07. OPV1; JAA08. OPV2; JAA09. OPV3; JAA14. Drop out rate for multidose vaccines; HAA1. Knowledge about immunisation; HAA2. Attitudes about immunisation; HAD5. Vaccination health card availability/ retention; HAD4. Experience and satisfaction with health services; IAB3. Formal HW motivation, capacity & performance	Child; Caregivers; Healthcare workers
Johri et al., 2015[55]	Journal Article	Systematic review & Meta-analysis	3	Sub-Saharan Africa, South Asia, Latin America & the Caribbean	11	F. Multicomponent; AA2. Short-term sensitization and education campaigns; AB. Incentives & motivation	JAA15. Vaccination coverage (unspecified)	Child
Kaufman et al., 2018[56]	Journal article	Systematic review & Meta-analysis	4	South Asia, East Asia and Pacific, North America, Western Europe	10	AA2. Short-term sensitization and education campaigns	JAA12. No vs. partial routine immunisation;	Child

[4] Our search timeframe was up to and including 2021, thus Jain et al. (2022) may appear to be at odds with this. However, Jain et al. (2021) was identified through the grey literature search but was subsequently published in 2022, hence we cite it as Jain et al. (2022) throughout.

For peer review only

Authors & Year	Source	Type of review	Geographic focus		No. primary studies	Types of intervention included	Types of outcomes included	Population level analysed
			No. of regions	Regions				
Lagarde & Palmer, 2009[57]	Journal article	Systematic review	3	South Asia, East Asia and Pacific, Latin America & the Caribbean	3	BF4. Health system financing	JAA01. Full routine immunisation for children	Child
Lagarde, Haines, & Palmer, 2009[58]	Journal article	Systematic review	2	Sub-Saharan Africa, Latin America & the Caribbean	10	AB1. Material/ monetary incentives for caregivers	JAA02. BCG; JAA11. Measles; JAA03. DPT1; JAA05. DPT3; JAA01. Full routine immunisation for children	Child
Linde et al., 2009[59]	Journal article	Systematic review & Meta-analysis	2	Sub-Saharan Africa, Middle East & North Africa	38	AB4. Written or pictorial messages (SMS, stickers, flyers etc.) to caregivers	JAA15. Vaccination coverage (unspecified)	Child
Mekonnen et al., 2019[60]	Journal Article	Systematic review & Meta-analysis	3	Sub-Saharan Africa, Latin America & the Caribbean, North America	10	AB4. Written or pictorial messages (SMS, stickers, flyers etc.) to caregivers	JAA15. Vaccination coverage (unspecified)	Child
Molina et al., 2016[61]	Published report	Systematic review & Meta-analysis	4	Sub-Saharan Africa, South Asia, East Asia and Pacific, Latin America & the Caribbean	15	DA1. Collaborating with whole community; AA2. Short-term sensitization and education campaigns; DA1. Collaborating with whole community	JAA15. Vaccination coverage (unspecified)	Child
Odendaal et al., 2018[62]	Journal article	Systematic review	2	East Asia and Pacific, Latin America & the Caribbean	2	BF4. Health system financing	JAA15. Vaccination coverage (unspecified); ICA1. Stockouts	Child; Health facility

For peer review only

Authors & Year	Source	Type of review	Geographic focus		No. primary studies	Types of intervention included	Types of outcomes included	Population level analysed
			No. of regions	Regions				
Oyo-Ita et al., 2016[63]	Journal article	Systematic review & Meta-analysis	4	Sub-Saharan Africa, South Asia, Latin America & the Caribbean, Eastern Europe and Central Asia	14	AA1. Sustained sensitization and education campaigns; AA2. Short-term sensitization and education campaigns; AB4. Written or pictorial messages (SMS, stickers, flyers etc.) to caregivers; BA1. Formal health worker training and education; BF1. Health system strategic planning; AB1. Material/ monetary incentives for caregivers; BB5. Outreach to vulnerable populations (hard-to-reach, SES, caste, etc; AB1. Material/ monetary incentives for caregivers; BB7. Home visits; BF1. Health system strategic planning	JAA05. DPT3; JAA01. Full routine immunisation for children; JAA11. Measles; JAA12. No vs. partial routine immunisation; JAA13. Vaccination Timeliness; JAA02. BCG; JAA03. DPT1; JAA09. OPV3	Child
Palmer et al., 2020[64]	Journal article	Systematic review & Meta-analysis	6	Sub-Saharan Africa, South Asia, East Asia and Pacific, Latin America & the Caribbean, Eastern Europe and Central Asia, North America	27	AB4. Written or pictorial messages (SMS, stickers, flyers etc.) to caregivers; AB3. Automated voice messages to caregivers	JAA15. Vaccination coverage (unspecified); JAA13. Vaccination Timeliness	Child
Saeterdal et al., 2014[65]	Journal Article	Systematic review	1	South Asia	2	AA2. Short-term sensitization and education campaigns	HAA1. Knowledge about immunisation; JAA15. Vaccination coverage (unspecified); JAA11. Measles; JAA05. DPT3; JAA09. OPV3; HAA2. Attitudes about immunisation; HAB2. Household norms & decision-making	Caregivers; Child

For peer review only

Authors & Year	Source	Type of review	Geographic focus		No. primary studies	Types of intervention included	Types of outcomes included	Population level analysed
			No. of regions	Regions				
Willey et al., 2013[66]	Published report	Systematic review	4	Sub-Saharan Africa, South Asia, East Asia and Pacific, Latin America & the Caribbean	12	BA1. Formal health worker training and education; BF1. Health system strategic planning	JAA15. Vaccination coverage (unspecified); JAA11. Measles	Child
Witter et al., 2012[67]	Journal article	Systematic review	2	Sub-Saharan Africa, East Asia and Pacific	9	BD5. Pay-for-performance schemes	JAA15. Vaccination coverage (unspecified)	Child
Medium confidence reviews (6 in total)								
Atkinson et al., 2019[68]	Journal article	Systematic review & Meta-analysis	4	Sub-Saharan Africa, Latin America & the Caribbean, Middle East & North Africa, North America	12	AB4. Written or pictorial messages (SMS, stickers, flyers etc.) to caregivers	JAA01. Full routine immunisation for children	Child
Bassani et al., 2013[69]	Journal article	Systematic review & Meta-analysis	3	Sub-Saharan Africa, South Asia, Latin America & the Caribbean	25	AB1. Material/ monetary incentives for caregivers	JAA15. Vaccination coverage (unspecified); JAA01. Full routine immunisation for children	Child
Chutiyami, Wyver, & Amin, 2019[70]	Journal article	Systematic review & Meta-analysis	7	Sub-Saharan Africa, South Asia, East Asia and Pacific, Latin America & the Caribbean, Middle East & North Africa, North America, Western Europe	32	AB4. Written or pictorial messages (SMS, stickers, flyers etc.) to caregivers	JAA15. Vaccination coverage (unspecified); HAA1. Knowledge about immunisation	Child; Caregivers
Desai et al., 2020[71]	Journal article	Systematic review	1	South Asia	99	DA2. Collaborating with selected community groups and networks	JAA15. Vaccination coverage (unspecified)	Child
Gilmore & McAuliffe 2013[72]	Journal Article	Systematic review	4	Sub-Saharan Africa, South Asia, East Asia and Pacific, Latin America & the Caribbean	17	AA2. Short-term sensitization and education campaigns	JAA01. Full routine immunisation for children; JAA15. Vaccination coverage (unspecified)	Child

For peer review only

Authors & Year	Source	Type of review	Geographic focus		No. primary studies	Types of intervention included	Types of outcomes included	Population level analysed
			No. of regions	Regions				
Lukusa et al., 2018[73]	Journal Article	Systematic review & Meta-analysis	1	South Asia	6	AA2. Short-term sensitization and education campaigns	JAA15. Vaccination coverage (unspecified); JAA05. DPT3	Child

For peer review only

Table 6.2. Low confidence systematic reviews

Authors & Year	Source	Type of review	Geographic focus		No. primary studies	Types of intervention included	Types of outcomes included	Population level analysed
			No. of regions	Regions				
Baequni, 2012[74]	Journal article	Systematic review & Meta-analysis	2	South Asia, East Asia and Pacific	4	AB4. Written or pictorial messages (SMS, stickers, flyers etc.) to caregivers	JAA15. Vaccination coverage (unspecified)	Child
Batt, Fox-Rushby, & Castillo-Riquelme, 2004[75]	Journal Article	Systematic review	5	Sub-Saharan Africa, South Asia, East Asia and Pacific, Latin America & the Caribbean, Middle East & North Africa	34	F. Multicomponent	JAA01. Full routine immunisation for children	Child
Bright et al., 2017[76]	Journal article	Systematic review	5	Sub-Saharan Africa, South Asia, East Asia and Pacific, Latin America & the Caribbean, Middle East & North Africa	57	BB5. Outreach to vulnerable populations (hard-to-reach, SES, caste, etc.); AB1. Material/ monetary incentives for caregivers; BB7. Home visits; BF1. Health system strategic planning; BD5. Pay-for-performance schemes; AA2. Short-term sensitization and education campaigns; AB4. Written or pictorial messages (SMS, stickers, flyers etc.) to caregivers; DA2. Collaborating with selected community groups and networks; BD4. Written or pictorial messages (SMS, stickers, flyers etc.) to health workers; F. Multicomponent	JAA15. Vaccination coverage (unspecified)	Child

For peer review only

Authors & Year	Source	Type of review	Geographic focus		No. primary studies	Types of intervention included	Types of outcomes included	Population level analysed
			No. of regions	Regions				
Crocker-Buque et al., 2017[77]	Journal article	Systematic review	4	Sub-Saharan Africa, South Asia, East Asia and Pacific, Latin America & the Caribbean	20	F. Multicomponent; BB5. Outreach to vulnerable populations (hard-to-reach, SES, caste, etc.); AB4. Written or pictorial messages (SMS, stickers, flyers etc.) to caregivers; AA2. Short-term sensitization and education campaigns; AB1. Material/ monetary incentives for caregivers	JAA15. Vaccination coverage (unspecified)	Child
Das, Gopalan, & Chandramohan, 2016[78]	Journal article	Systematic review	3	Sub-Saharan Africa, East Asia and Pacific, Middle East & North Africa	8	BD5. Pay-for-performance schemes	ICA1. Stockouts	Health facility
de Cock et al., 2020[79]	Journal Article	Systematic review	5	Sub-Saharan Africa, South Asia, East Asia and Pacific, North America, Western Europe	28	BG1. New HMIS/Dashboard systems (incl. improved data collection)	JAA15. Vaccination coverage (unspecified)	Child
de Souza Cruz et al., 2017[80]	Journal Article	Systematic review	3	Sub-Saharan Africa, South Asia, Latin America & the Caribbean	17	AB1. Material/ monetary incentives for caregivers	JAA15. Vaccination coverage (unspecified); JAA01. Full routine immunisation for children	Child
Deardorff et al., 2018[81]	Journal article	Systematic review & Meta-analysis	5	Sub-Saharan Africa, South Asia, East Asia and Pacific, Latin America & the Caribbean, Middle East & North Africa	28	AA2. Short-term sensitization and education campaigns; AA3. Public information campaigns; CA1. Faith-based outreach/outreach using local leaders; BC1. National/sub-national immunisation days	JAA15. Vaccination coverage (unspecified); JAA11. Measles	Child

For peer review only

Authors & Year	Source	Type of review	Geographic focus		No. primary studies	Types of intervention included	Types of outcomes included	Population level analysed
			No. of regions	Regions				
Freeman et al., 2017[82]	Journal article	Systematic review	8	Sub-Saharan Africa, South Asia, East Asia and Pacific, Latin America & the Caribbean, Middle East & North Africa, Eastern Europe and Central Asia, North America, Western Europe	489	BB2. Community health worker involvement in planning & monitoring; BB7. Home visits; CA1. Faith-based outreach/outreach using local leaders; AA3. Public information campaigns; AA2. Short-term sensitization and education campaigns; AB1. Material/ monetary incentives for caregivers; BC1. National/sub-national immunisation days; BB7. Home visits	JAA15. Vaccination coverage (unspecified); JAA01. Full routine immunisation for children	Child
Giedion, Andrés Alfonso, & Díaz, 2013[83]	Published report	Systematic review	6	Sub-Saharan Africa, South Asia, East Asia and Pacific, Latin America & the Caribbean, Middle East & North Africa, Eastern Europe and Central Asia	105	AB5. Changes to health system user fees	JAA01. Full routine immunisation for children	Child
Harvey et al., 2015[84]	Journal article	Systematic review & Meta-analysis	5	Sub-Saharan Africa, South Asia, East Asia and Pacific, North America, Western Europe	28	AA2. Short-term sensitization and education campaigns; AB4. Written or pictorial messages (SMS, stickers, flyers etc.) to caregivers	JAA15. Vaccination coverage (unspecified)	Child
Iwu et al., 2019[85]	Journal Article	Systematic review	3	Sub-Saharan Africa, South Asia, North America	4	BF1. Health system strategic planning; BG1. New HMIS/Dashboard systems (incl. improved data collection); BG2. Capacity building (e.g., training) for existing systems	ICA1. Stockouts; IBA1. Immunisation data collection (quality, completeness); HAD2. Actual cost of vaccinating	Health facility; Child

For peer review only

Authors & Year	Source	Type of review	Geographic focus		No. primary studies	Types of intervention included	Types of outcomes included	Population level analysed
			No. of regions	Regions				
Jarrett et al., 2015[86]	Journal Article	Systematic review	4	Sub-Saharan Africa, South Asia, Eastern Europe and Central Asia, Western Europe	13	CA1. Faith-based outreach/outreach using local leaders; F. Multicomponent; AA2. Short-term sensitization and education campaigns; BA1. Formal health worker training and education; AB1. Material/ monetary incentives for caregivers; AB4. Written or pictorial messages (SMS, stickers, flyers etc.) to caregivers	JAA09. OPV3; JAA15. Vaccination coverage (unspecified); JAA05. DPT3	Child
Karageorgos et al., 2019[87]	Journal Article	Systematic review	5	Sub-Saharan Africa, South Asia, East Asia and Pacific, Latin America & the Caribbean, Middle East & North Africa	98	AB4. Written or pictorial messages (SMS, stickers, flyers etc.) to caregivers	JAA15. Vaccination coverage (unspecified)	Child
Kim, Patel, & Hinman, 2017[88]	Journal Article	Systematic review	4	Sub-Saharan Africa, South Asia, East Asia and Pacific, Latin America & the Caribbean	26	AB4. Written or pictorial messages (SMS, stickers, flyers etc.) to caregivers; BG1. New HMIS/Dashboard systems (incl. improved data collection); AA2. Short-term sensitization and education campaigns	JAA15. Vaccination coverage (unspecified); JAA13. Vacc. Timeliness; IBA1. Immunisation data collection (quality, completeness); HAC1. Readiness to vaccinate	Child; Healthcare workers; Caregivers
Loevinsohn & Harding, 2004[89]	Discussion paper	Systematic review	4	Sub-Saharan Africa, South Asia, East Asia and Pacific, Latin America & the Caribbean	10	BF4. Health system financing	JAA15. Vaccination coverage (unspecified);	Child;
Munk et al., 2019[37]	Journal Article	Systematic review	4	Sub-Saharan Africa, South Asia, East Asia and Pacific, Latin America & the Caribbean	14	F. Multicomponent	JAA15. Vaccination coverage (unspecified); HAD2. Actual cost of vaccinating	Child

For peer review only

Authors & Year	Source	Type of review	Geographic focus		No. primary studies	Types of intervention included	Types of outcomes included	Population level analysed
			No. of regions	Regions				
Mureed et al., 2015[90]	Journal Article	Systematic review & Meta-analysis	3	Sub-Saharan Africa, South Asia, Middle East & North Africa	10	F. Multicomponent	JAA15. Vaccination coverage (unspecified); JAA05. DPT3; JAA09. OPV3; JAA11. Measles; JAA01. Full routine immunisation for children	Child
Naugle & Hornik, 2014[91]	Journal Article	Systematic review	6	Sub-Saharan Africa, South Asia, East Asia and Pacific, Latin America & the Caribbean, Middle East & North Africa, Eastern Europe and Central Asia	106	AA3. Public information campaigns	JAA15. Vaccination coverage (unspecified)	Child
Nelson et al., 2016[92]	Journal Article	Systematic review	6	Sub-Saharan Africa, South Asia, East Asia and Pacific, Latin America & the Caribbean, Middle East & North Africa, Eastern Europe and Central Asia	15	F. Multicomponent; BF1. Health system strategic planning	JAA01. Full routine immunisation for children; JAA05. DPT3	Child
Nutman, McKee, & Khoshnood, 2013[93]	Journal Article	Systematic review	1	Sub-Saharan Africa	21	BF1. Health system strategic planning	JAA15. Vaccination coverage (unspecified)	Child
Oliver-Williams et al., 2017[94]	Journal article	Systematic review	3	Sub-Saharan Africa, South Asia, East Asia and Pacific	21	AB3. Automated voice messages to caregivers; AB4. Written or pictorial messages (SMS, stickers, flyers etc.) to caregivers	JAA15. Vaccination coverage (unspecified)	Child

Authors & Year	Source	Type of review	Geographic focus		No. primary studies	Types of intervention included	Types of outcomes included	Population level analysed
			No. of regions	Regions				
Omoniyi & Williams, 2020[38]	Journal article	Systematic review	5	Sub-Saharan Africa, South Asia, East Asia and Pacific, Latin America & the Caribbean, Middle East & North Africa	27	AA2. Short-term sensitization and education campaigns; CA1. Faith-based outreach/outreach using local leaders; AB4. Written or pictorial messages (SMS, stickers, flyers etc.) to caregivers; AB1. Material/ monetary incentives for caregivers; BB7. Home visits; CA1. Faith-based outreach/outreach using local leaders; BA1. Formal health worker training and education; BF1. Health system strategic planning; F. Multicomponent	JAA15. Vaccination coverage (unspecified)	Child
Onwuchekwa, Verdonck, & Marchal, 2021[43]	Journal Article	Systematic review	1	Sub-Saharan Africa	9	AB1. Material/ monetary incentives for caregivers	JAA15. Vaccination coverage (unspecified)	Child
Owusu-Addo & Cross, 2014[95]	Journal article	Systematic review	2	Sub-Saharan Africa, Latin America & the Caribbean	16	AB1. Material/ monetary incentives for caregivers	JAA15. Vaccination coverage (unspecified)	Child
Ozawa, Yemeke, & Thompson, 2018[96]	Journal article	Systematic review	4	Sub-Saharan Africa, South Asia, East Asia and Pacific, Latin America & the Caribbean	42	F. Multicomponent	0	Child

For peer review only

Authors & Year	Source	Type of review	Geographic focus		No. primary studies	Types of intervention included	Types of outcomes included	Population level analysed
			No. of regions	Regions				
Pal, Goodyear-Smith, & Exeter, 2016[97]	Journal article	Systematic review	2	South Asia, East Asia and Pacific	14	AA2. Short-term sensitization and education campaigns; AB4. Written or pictorial messages (SMS, stickers, flyers etc.) to caregivers; BA1. Formal health worker training and education; DA1. Collaborating with whole community; AB1. Material/ monetary incentives for caregivers	JAA05. DPT3; JAA03. DPT1	Child
Parrado-Jiménez, 2013[98]	Unpublished research	Systematic review	5	Sub-Saharan Africa, South Asia, East Asia and Pacific, Latin America & the Caribbean, Middle East & North Africa	21	BD5. Pay-for-performance schemes	JAA01. Full routine immunisation for children	Child
Pegurri, Fox-Rushby, & Damian, 2005[99]	Journal article	Systematic review	3	Sub-Saharan Africa, South Asia, Latin America & the Caribbean	60	F. Multicomponent	JAA15. Vaccination coverage (unspecified)	Child
Pinzón-Flórez et al., 2015[100]	Journal article	Systematic review	4	Sub-Saharan Africa, South Asia, East Asia and Pacific, Latin America & the Caribbean	19	AA2. Short-term sensitization and education campaigns	JAA01. Full routine immunisation for children; HAD5. Vaccination health card availability/ retention	Child
Rahman et al., 2013[101]	Journal Article	Systematic review & Meta-analysis	4	Sub-Saharan Africa, South Asia, East Asia and Pacific, Latin America & the Caribbean	13	BB7. Home visits	JAA01. Full routine immunisation for children	Child
Ranganathan & Lagarde, 2012[102]	Journal Article	Systematic review	3	Sub-Saharan Africa, South Asia, Latin America & the Caribbean	18	AB1. Material/ monetary incentives for caregivers	JAA15. Vaccination coverage (unspecified)	Child

For peer review only

Authors & Year	Source	Type of review	Geographic focus		No. primary studies	Types of intervention included	Types of outcomes included	Population level analysed
			No. of regions	Regions				
Ryman, Dietz, & Cairns, 2008[103]	Journal Article	Systematic review	4	Sub-Saharan Africa, South Asia, East Asia and Pacific, Latin America & the Caribbean	25	AA2. Short-term sensitization and education campaigns; BB5. Outreach to vulnerable populations (hard-to-reach, SES, caste, etc.); BB7. Home visits; AA1. Sustained sensitization and education campaigns; AA3. Public information campaigns; AA1. Sustained sensitization and education campaigns; BF1. Health system strategic planning; AB4. Written or pictorial messages (SMS, stickers, flyers etc.) to caregivers; BF4. Health system financing; BA1. Formal health worker training and education; BD1. Material/monetary incentives for health workers	JAA15. Vaccination coverage (unspecified); JAA13. Vacc. Timeliness	Child
Shea, Andersson, & Henry, 2009[104]	Journal Article	Systematic review	5	Sub-Saharan Africa, South Asia, East Asia and Pacific, Latin America & the Caribbean, Middle East & North Africa	8	AA3. Public information campaigns; F. Multicomponent; BB5. Outreach to vulnerable populations (hard-to-reach, SES, caste, etc.); AA2. Short-term sensitization and education campaigns; BD1. Material/monetary incentives for health workers; BB5. Outreach to vulnerable populations (hard-to-reach, SES, caste, etc.); BB7. Home visits; AA. Information & education	JAA01. Full routine immunisation for children; HAA1. Knowledge about immunisation; JAA15. Vaccination coverage (unspecified); JAA11. Measles; HAD2. Actual cost of vaccinating	Child; Caregivers
Smith et al., 2018[105]	Journal Article	Systematic review	1	Sub-Saharan Africa	28	BF1. Health system strategic planning	JAA15. Vaccination coverage (unspecified)	Child

For peer review only

Authors & Year	Source	Type of review	Geographic focus		No. primary studies	Types of intervention included	Types of outcomes included	Population level analysed
			No. of regions	Regions				
Watterson, Walsh, & Madeka, 2015[106]	Journal Article	Systematic review	2	Sub-Saharan Africa, East Asia and Pacific	10	AB4. Written or pictorial messages (SMS, stickers, flyers etc.) to caregivers; BG1. New HMIS/Dashboard systems (incl. improved data collection); AB1. Material/ monetary incentives for caregivers; AB3. Automated voice messages to caregivers; AA2. Short-term sensitization and education campaigns	JAA13. Vacc. Timeliness; HAC1. Readiness to vaccinate; JAA11. Measles	Child; Caregivers
Yuan et al., 2014[107]	Journal Article	Systematic review	4	Sub-Saharan Africa, South Asia, East Asia and Pacific, Latin America & the Caribbean	22	BB5. Outreach to vulnerable populations (hard-to-reach, SES, caste, etc.); AA3. Public information campaigns; BF1. Health system strategic planning	JAA11. Measles	Child
Yunusa et al., 2021[108]	Journal article in progress	Systematic review & Meta-analysis	3	Sub-Saharan Africa, South Asia, Latin America & the Caribbean	11	AB4. Written or pictorial messages (SMS, stickers, flyers etc.) to caregivers; AB3. Automated voice messages to caregivers	JAA01. Full routine immunisation for children; JAA15. Vaccination coverage (unspecified); JAA13. Vacc. Timeliness	Child

For peer review only

Appendix 8: Risk of bias of included systematic reviews

Sr. No.	Included systematic review	Confidence rating in RoR	Confidence rating in EGM	Type of synthesis	Reasons, if confidence rating differs between RoR and EGM
**High confidence studies (18 in total)**					
1	Diaconu et al., 2021[52]	High	NA#	Range of ES + DC	
2	Eze et al., 2021[44]	High	NA	MR + MA + VC direction of effect	
3	Gera et al., 2016[53]	High	High	MA + NS	
4	Glenton et al., 2011[54]	High	NA	MA + NS	
5	Jain et al., 2022[41, 42]	High	NA	MR + MA + TS	
6	Johri et al., 2015[55]	High	Medium	MR + MA	Upgraded to high confidence: EGM coders marked section A as low confidence because the search was not comprehensive. RoR coders marked section A as high confidence as search included studies from other published reviews dating back to 1996. However, we acknowledge some limitations of the search, but these were not sufficient for a low confidence rating.
7	Kaufman et al., 2018[56]	High	High	MA	
8	Lagarde et al., 2009[58]	High	High	NS	
9	Lagarde & Palmer, 2009[57] (update cited as Odendaal et al., 2018[62])	High	High	DC + NS	
10	Linde et al., 2009[59]	High	High	MA + NS	
11	Mekonnen et al., 2019[60]	High	Medium	MR + MA	Upgraded to high confidence: No obvious difference between grading of RoR and EGM coders, however, mitigating factors (e.g., Kappa statistics and Grade assessment) which influenced the overall assessment to high confidence. We acknowledge the search was restricted to English only and unit of analysis error was not reported but this is not sufficient to justify a medium confidence rating.
12	Molina et al., 2016[61]	High	High	MA + DC + NS	

Sr. No.	Included systematic review	Confidence rating in RoR	Confidence rating in EGM	Type of synthesis	Reasons, if confidence rating differs between RoR and EGM
13	Odendaal et al., 2018[62]	High	High	DC + NS	
14	Oyo-Ita et al., 2016[63]	High	High	MA + DC	
15	Palmer et al., 2020[64]	High	NA	MA + DC + NS	
16	Saeterdal et al., 2014[65]	High	High~	DC + NS	
17	Willey et al., 2012[66]	High	High	NS + NS	
18	Witter et al., 2012[67] (update cited as Diaconu et al., 2021[52])	High	High	DC + NS	
**Medium confidence studies (6 in total)**					
19	Atkinson et al., 2019[68]	Medium	Medium	MA	
20	Bassani et al., 2013[69]	Medium	Medium	MA	
21	Chutiyami et al., 2019[70]	Medium	Low	MA + Range of ES + VC statistical significance + VC direction of effect + DC + NS	Upgraded to medium confidence: EGM coders marked section A as low confidence as search was partial, search period not documented and no sufficient criteria to assess study quality. RoR coders marked section A as medium because included studies dated back to 1984 and sufficient criteria were used to assess quality while acknowledging some search limitations. EGM coders marked section B as low. RoR coders upgraded this to medium because 20% of studies were randomly extracted by two independent researchers. Also, section B3 was upgraded as heterogeneity was acknowledged and statistical tests to demonstrate heterogeneity were performed. Unit of analysis error was not documented.
22	Desai et al., 2020[71]	Medium	NA	NS + TS	
23	Gilmore & McAuliffe, 2013[72]	Medium	Medium	Range of ES + NS	
24	Lukusa et al., 2018[73]	Medium	Medium	MA	

Sr. No.	Included systematic review	Confidence rating in RoR	Confidence rating in EGM	Type of synthesis	Reasons, if confidence rating differs between RoR and EGM
**Low confidence studies (38 in total):**					
25	Baequni, 2012[74]	Low	Low	MA	
26	Batt et al., 2004[75]	Low	Low	DC	
27	Bright et al., 2017[76]	Low	Low	VC statistical significance + NS	
28	Crocker-Buque et al., 2017[77]	Low	Low	NS + TS	
29	Das et al., 2016[78]	Low	Low	DC + NS	
30	de Cock et al., 2020[79]	Low	NA	VC direction of effect + NS	
31	de Souza Cruz et al., 2017[80]	Low	Low	NS	
32	Deardorff et al., 2018[81]	Low	Low	MA + DC + NS	
33	Freeman et al., 2017[82]	Low	Low	NS	
34	Giedion et al., 2013[83]	Low	Low	NS	
35	Harvey et al., 2015[84]	Low	Low	MA + Range of ES	
36	Iwu et al., 2019[85]	Low	Low	NS	
37	Jarrett et al., 2015[86]	Low	Low	DC + NS	
38	Karageorgos et al., 2018[87]	Low	Low	NS	
39	Kim et al., 2017[88]	Low	Low	NS	
40	Loevinsohn & Harding, 2004[89]	Low	Low	Range of ES + DC + NS	
41	Munk et al., 2019[37]	Low	Low	Range of ES + DC	
42	Mureed et al., 2015[90]	Low	Low	MA + VC direction of effect	
43	Naugle & Hornik, 2014[91]	Low	Low	VC direction of effect + DC + NS	
44	Nelson et al., 2016[92]	Low	Low	Range of ES + NS	
45	Nutman et al., 2013[93]	Low	Low	Range of ES + VC statistical significance + DC + NS	
46	Oliver-Williams et al., 2017[94]	Low	Low	NS	
47	Omoniyi & Williams, 2020[38]	Low	Low	TS	
48	Onwuchekwa et al., 2021[43]	Low	NA	VC direction of effect + NS + TS	
49	Owusu-Addo & Cross, 2014[95]	Low	Low	NS	

Sr. No.	Included systematic review	Confidence rating in RoR	Confidence rating in EGM	Type of synthesis	Reasons, if confidence rating differs between RoR and EGM
50	Ozawa et al., 2018[96]	Low	Low	DC	
51	Pal et al., 2016[97]	Low	Low	NS	
52	Parrado-Jiménez, 2013[98]	Low	Low	VC statistical significance & direction of effect	
53	Pegurri et al., 2005[99]	Low	Low	DC	
54	Pinzón-Flórez et al., 2015[100]	Low	NA	NS	
55	Rahman et al., 2013[101]	Low	Low	MA + NS	
56	Ranganathan & Lagarde, 2012[102]	Low	Low	NS	
57	Ryman et al., 2008[103]	Low	Low	NS	
58	Shea et al., 2009[104]	Low	Low	NS	
59	Smith et al., 2018[105]	Low	Low	NS	
60	Watterson et al., 2015[106]	Low	Low	NS	
61	Yuan et al. 2014[107]	Low	Medium	NS	Downgraded to low confidence: Authors did not extract data independently. Additionally had further limitations: A summary of the assessment of each included study for each quality assessment criterion was not reported, unit of analysis error was not reported in spite of including cRCTs.
62	Yunusa et al., 2021[108]	Low	NA	MA + NS	

Notes: ~Saeterdal et al., 2014[65] was mistakenly marked as medium confidence in the earlier draft of the EGM manuscript and online map, but this will be rectified before publication.
#NA in column, confidence rating in EGM: these are the systematic reviews identified through our updated search and were not included in the EGM.
Abbreviations: cRCTs: Cluster randomised controlled trials; DC: Descriptive; EGM: Evidence Gap Maps; ES: Effect sizes; MA: Meta-analysis; MR: Meta regression; NS: Narrative synthesis; RoR: Review of reviews; TS: Thematic synthesis; VC: Vote counting.

Appendix 9: Summary of main findings

Intervention (1st, 2nd, 3rd tier)			# SRs	SRs (Low confidence SRs in parenthesis)	Evidence as reported in the high & medium confidence SRs		Quantitative findings from RoR	Overall findings**
					Quantitative findings	Qualitative findings		
A. Caregiver-oriented interventions (1st tier)	AA. Information & education (2nd tier)	AA1. Sustained sensitisation and education campaigns (3rd tier)	1	(Ryman et al., 2008[31])	None	None	None	None (Low confidence evidence)
		AA2. One-time sensitisation and education campaigns (3rd tier)	12	Kaufman et al., 2018; Gilmore & McAuliffe, 2013; Johri et al., 2015; Lukusa et al., 2018; Oyo-Ita et al., 2016; Saeterdal et al., 2014[6, 8, 9, 11, 12, 32] (Crocker-Buque et al., 2017; Deardorff et al.,	Seven pooled estimates are available from 4 SRs; of which 6 were positive and statistically significant. RR ranged between 1·2 and 1·68.	Most SRs included small number of primary studies and reported favourable effects of AA2 intervention on range of outcomes: DPT3, Measles, Vaccination coverage (unspecified) and other secondary outcomes.	The average RR was 1·38 (95% CI: 1·33 to 1·44). Refer to Figure 2	Positive and statistically significant

For peer review only

Intervention (1st, 2nd, 3rd tier)			# SRs	SRs (Low confidence SRs in parenthesis)	Evidence as reported in the high & medium confidence SRs		Quantitative findings from RoR	Overall findings**
					Quantitative findings	Qualitative findings		
				2018; Jarette et al., 2015; Kim et al., 2017; Pinzón-Flórez et al., 2015; Shea et al., 2009[33-38])				
		AA3. Public information campaigns (3rd tier)	3	(Naugle & Hornik, 2014; Shea et al. 2009; Yuan et al. 2014[38-40])	None	None	None	None (Low confidence evidence)

For peer review only - http://bmjopen.bmj.com/site/about/guidelines.xhtml

	AB. Incentives & motivation (2nd tier)	AB1. Material/ monetary incentives for caregivers (3rd tier)	12	Bassani et al., 2013; Lagarde et al., 2009; Oyo-Ita et al., 2016[11, 41, 42] (Bright et al., 2017; Crocker-Buque et al., 2017; de Souza Cruz et al., 2017; Jarette et al., 2015; Onwuchekwa et al., 2021; Omoniyi 2020; Owusu-Addo & Cross, 2014; Pal et al., 2016; Ranganathan & Lagarde, 2012[33, 35, 43-49])	Meta-analysis was done by two SRs, both found statistically insignificant results (RR: 1·05 [0·90, 1·23], SMD ranging from 0·05 to 0·06).	Most outcomes were narratively synthesised with small number of primary studies, suggesting either inconclusive or favourable effect.	MA was not feasible.	Mixed effects

For peer review only

Intervention (1st, 2nd, 3rd tier)			# SRs	SRs (Low confidence SRs in parenthesis)	Evidence as reported in the high & medium confidence SRs		Quantitative findings from RoR	Overall findings**
					Quantitative findings	Qualitative findings		
		AB2. Non-material incentives for caregivers (3rd tier)	0	NA	None	None	None	No evidence
		AB3. Automated voice messages to caregivers (3rd tier)	1	(Yunusa et al., 2021[50])	None	None	None	None (Low confidence evidence)

For peer review only

		AB4. Written or pictorial messages (SMS, stickers, flyers etc.) to caregivers (3rd tier)	13	Atkinson et al., 2019; Chutiyami et al., 2019; Eze et al., 2021; Linde et al., 2019; Mekonnen et al., 2019; Palmer et al., 2020[5, 10, 51-54] (Baequni, 2012; Crocker-Buque et al., 2017; Jarette et al., 2015; Karageorgos et al., 2019; Kim et al., 2017; Omoniyi & Williams, 2020; Yunusa et al., 2021[33, 35, 36, 45, 50, 55, 56])	Meta-analysis was done by all high & medium confidence SRs reporting nine estimates for a range of outcomes, all found positive and statistically significant effects. RR or OR ranged between 1·13 and 3·69.	Intervention was found to be effective in improving Vaccination coverage (unspecified), DPT 3, Full routine immunization, and Vaccination timeliness.	RR of 1·24 (95% CI: 1·11 to 1·36).<br>Refer to Figure 3	Positive and statistically significant

Intervention (1st, 2nd, 3rd tier)			# SRs	SRs (Low confidence SRs in parenthesis)	Evidence as reported in the high & medium confidence SRs		Quantitative findings from RoR	Overall findings**
					Quantitative findings	Qualitative findings		
		AB5. Changes to health system user fees (3rd tier)	1	(Giedion et al., 2013[57])	None	None	None	None (Low confidence evidence)
B. Health system-oriented (1st tier)	BA. Education & training (2nd tier)	BA1. Formal health worker training and education (3rd tier)	3	Oyo-Ita et al., 2016[11] (Jarette et al., 2015; Pal et al., 2016[35, 48])	None	Inconclusive effect on outcomes based on single primary study.	None	Limited evidence
		BA2. Community health worker training and education (3rd tier)	0	None	None	None	None	No evidence

Intervention (1st, 2nd, 3rd tier)			# SRs	SRs (Low confidence SRs in parenthesis)	Evidence as reported in the high & medium confidence SRs		Quantitative findings from RoR	Overall findings**
					Quantitative findings	Qualitative findings		
	BB. Planning, implementation, monitoring (2nd tier)	BB1. Formal health worker involvement in planning & monitoring (3rd tier)	0	None	None	None	None	No evidence
		BB2. Community health worker involvement in planning & monitoring (3rd tier)	0	None	None	None	None	No evidence
		BB3. Paper-based tracking (3rd tier)	0	None	None	None	None	No evidence
		BB4. Promoting outreach to vaccine-hesitant groups (3rd tier)	0	None	None	None	None	No evidence

For peer review only

Intervention (1st, 2nd, 3rd tier)			# SRs	SRs (Low confidence SRs in parenthesis)	Evidence as reported in the high & medium confidence SRs		Quantitative findings from RoR	Overall findings**
					Quantitative findings	Qualitative findings		
		BB5. Outreach to vulnerable populations (hard-to-reach, SES, caste, etc.) (3rd tier)	3	Oyo-Ita et al., 2016[11] (Crocker-Buque et al., 2017; Yuan et al., 2014[33, 40])	None	Low certainty favourable effect on the outcome. However, the results are based on a single primary study.	None	Limited evidence
		BB6. Outreach to migrant populations (3rd tier)	0	None	None	None	None	No evidence
		BB7. Home visits (3rd tier)	5	Glenton et al., 2011; Oyo-Ita et al., 2016[11, 58] (Freeman et al., 2017; Rahman et al., 2013; Shea et al., 2009[38, 59, 60])	None	Favourable effect of intervention on OPV3, measles and full routine immunisation but the results were based on a single primary study for each outcome.	None	Positive

Intervention (1st, 2nd, 3rd tier)			# SRs	SRs (Low confidence SRs in parenthesis)	Evidence as reported in the high & medium confidence SRs		Quantitative findings from RoR	Overall findings**
					Quantitative findings	Qualitative findings		
		BB8. Campaigns to vaccinate refugee populations (3rd tier)	0	None	None	None	None	No evidence
	BC. Supplementary Immunisation Activities (2nd tier)	BC1. National/sub-national immunisation days (3rd tier)	2	(Deardoff 2018; Freeman et al., 2017[34, 59])	None	None	None	None (Low confidence evidence)
	BD. Incentives & motivation (2nd tier)	BD1. Material/monetary incentives for health workers (3rd tier)	1	(Shea et al., 2009[38])	None	None	None	None (Low confidence evidence)
		BD2. Non-material incentives for health workers (3rd tier)	0	None	None	None	None	No evidence
		BD3. Automated voice messages to health workers (3rd tier)	0	None	None	None	None	No evidence

Intervention (1st, 2nd, 3rd tier)			# SRs	SRs (Low confidence SRs in parenthesis)	Evidence as reported in the high & medium confidence SRs		Quantitative findings from RoR	Overall findings**
					Quantitative findings	Qualitative findings		
		BD4. Written or pictorial messages (SMS, stickers, flyers etc.) to health workers (3rd tier)	1	(Bright et al., 2017[43])	None	None	None	None (Low confidence evidence)

For peer review only

		BD5. Pay-for-performance schemes (3rd tier)	5	Diaconu et al., 2021; Witter et al., 2012[7, 61] (Bright et al., 2017; Das et al., 2016; Parrado-Jiménez, 2013[43, 62, 63])	None	For most vaccination coverage related outcomes, the effect was inconclusive (low certainty evidence). While, for some of the intermediate outcomes it was positive and statistically significant. As per review authors, the aforesaid findings could be because of diversity in these schemes and the differences in outcome measures by the primary studies.	None	Mixed effects

Intervention (1st, 2nd, 3rd tier)			# SRs	SRs (Low confidence SRs in parenthesis)	Evidence as reported in the high & medium confidence SRs		Quantitative findings from RoR	Overall findings**
					Quantitative findings	Qualitative findings		
	BE. Infrastructure (2nd tier)	BE1. Building & upgrading health clinics (3rd tier)	0	None	None	None	None	No evidence
		BE2. Cold chain infrastructure improvements (3rd tier)	0	None	None	None	None	No evidence
	BF. Health system governance, policies and financing (2nd tier)	BF1. Health system strategic planning (3rd tier)	7	Oyo-Ita et al., 2016[11] (Bright et al., 2017; Nelson et al., 2016; Nutman et al., 2013; Omoniyi & Williams, 2020; Smith et al., 2018; Yuan et al., 2014[40, 43, 45, 64-66])	None	Inconclusive results for most outcomes, from a single primary study.	None	Limited evidence

Intervention (1st, 2nd, 3rd tier)			# SRs	SRs (Low confidence SRs in parenthesis)	Evidence as reported in the high & medium confidence SRs		Quantitative findings from RoR	Overall findings**
					Quantitative findings	Qualitative findings		
		BF2. Vaccination guidelines (3rd tier)	0	None	None	None	None	No evidence
		BF3. Changes to broader governance systems (beyond health systems) (3rd tier)	0	None	None	None	None	No evidence
		BF4. Health system financing (3rd tier)	3	Lagarde & Palmer 2009; Odendaal et al., 2018[67, 68] (Loevinsohn & Harding, 2004[69])	None	Inconclusive results for a range of outcomes. The results are based on one or two primary studies for each outcome.	None	Inconclusive

For peer review only

Intervention (1st, 2nd, 3rd tier)			# SRs	SRs (Low confidence SRs in parenthesis)	Evidence as reported in the high & medium confidence SRs		Quantitative findings from RoR	Overall findings**
					Quantitative findings	Qualitative findings		
	BG. Technology & mHealth (2nd tier)	BG1. New HMIS/Dashboard systems (incl. improved data collection) (3rd tier)	3	(de Cock et al., 2020; Kim et al., 2017; Watterson et al., 2015[36, 70, 71])	None	None	None	None (Low confidence evidence)
		BG2. Capacity building (e.g., training) for existing systems (3rd tier)	0	None	None	None	None	No evidence
C. Other community member-oriented (1st tier)	CA. Other community member- oriented (2nd tier)	CA1. Faith-based outreach/outreach using local leaders (3rd tier)	1	(Jarrette et al., 2015[35])	None	None	None	None (Low confidence evidence)

For peer review only

Intervention (1st, 2nd, 3rd tier)			# SRs	SRs (Low confidence SRs in parenthesis)	Evidence as reported in the high & medium confidence SRs		Quantitative findings from RoR	Overall findings**
					Quantitative findings	Qualitative findings		
D. Community-level (1st tier)	DA. Communication & dialogue (2nd tier)	DA1. Collaborating with whole community (3rd tier)	1	Molina et al., 2016[72]	None	Inconclusive results based on a single primary study.	None	Limited evidence
		DA2. Collaborating with selected community groups and networks (3rd tier)	1	Desai et al., 2020[73]	None	Small and statistically insignificant result based on two primary studies.	None	Limited evidence
	DB. Tracking & registering (2nd tier)	DB1. Community tracking and registering (3rd tier)	0	None	None	None	None	No evidence
E. Policies and institutions (1st tier)	EA. Education policy and infrastructure (2nd tier)	EA1. Education policy and infrastructure (3rd tier)	0	None	None	None	None	No evidence

For peer review only

Intervention (1st, 2nd, 3rd tier)			# SRs	SRs (Low confidence SRs in parenthesis)	Evidence as reported in the high & medium confidence SRs		Quantitative findings from RoR	Overall findings**
					Quantitative findings	Qualitative findings		
	EB. Non-health/ education infrastructure (2nd tier)	EB1. Non-health/ education infrastructure (e.g., electrification) (3rd tier)	0	None	None	None	None	No evidence
F. Multicomponent (1st tier)			1*	Jain et al., 2022[29, 30]	Meta-analysis done on a range of outcomes and found positive and statistically significant effect on most of the outcomes. The SMD ranged between 0·06 and 0·24	None	None	Positive and statistically significant

For peer review only

Notes: *Apart from Jain et al. 2022 there were 25 systematic reviews that assessed the effects of more than one intervention that are not part of this table. Of these reviews, the majority (n = 18) were low confidence SRs and the intervention components were heterogenous. We provide summary of Jain et al. 2022 in this table because it is one of the only included SRs that assessed community engagement interventions. Intervention and outcome codes are taken from Appendix 1.

** The last column, 'overall findings' are summarized from evidence as reported in the systematic reviews, qualitative and quantitative findings and quantitative findings from review of reviews (RoR). The current table (in Appendic 9) is the condensed form of Table 2 in main manuscript file.

For peer review only

Appendix 10: RVE analysis – 1st tier synthesis

Figure A8.1: RVE of all interventions under Type A, 1st tier, mean baseline coverage values

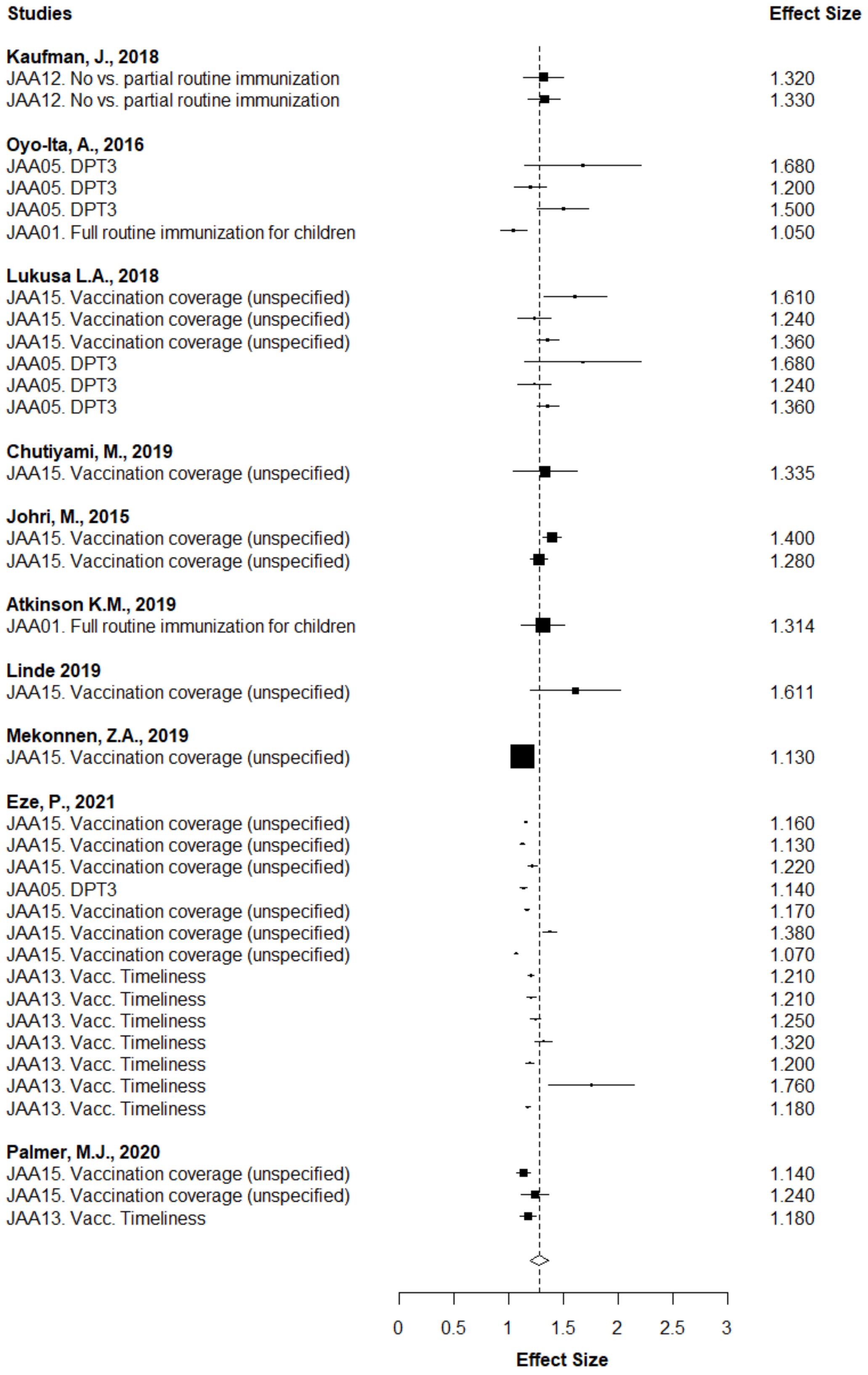

Notes: Drawing on k = 10 and 35 effect sizes.

Additional RVE analysis – 1st tier synthesis

Figure A8.2: RVE of all interventions under Type A, 1st tier, one standard deviation above the mean (baseline coverage rate of 70%)

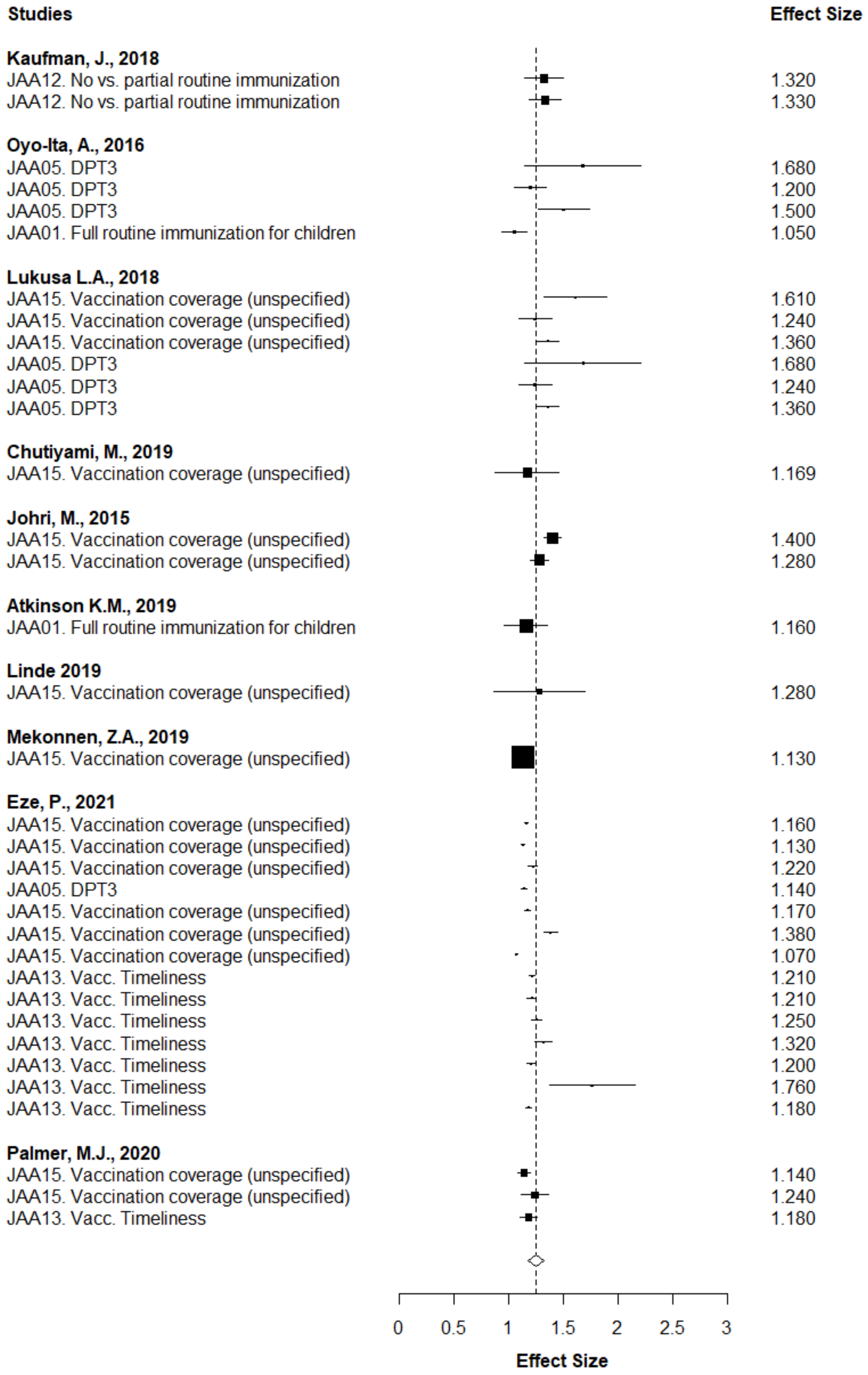

Notes: Drawing on k = 10 and 35 effect sizes.

Figure A8.3: RVE of all interventions under Type A, 1st tier, one standard deviation below the mean (baseline coverage rate of 26%)

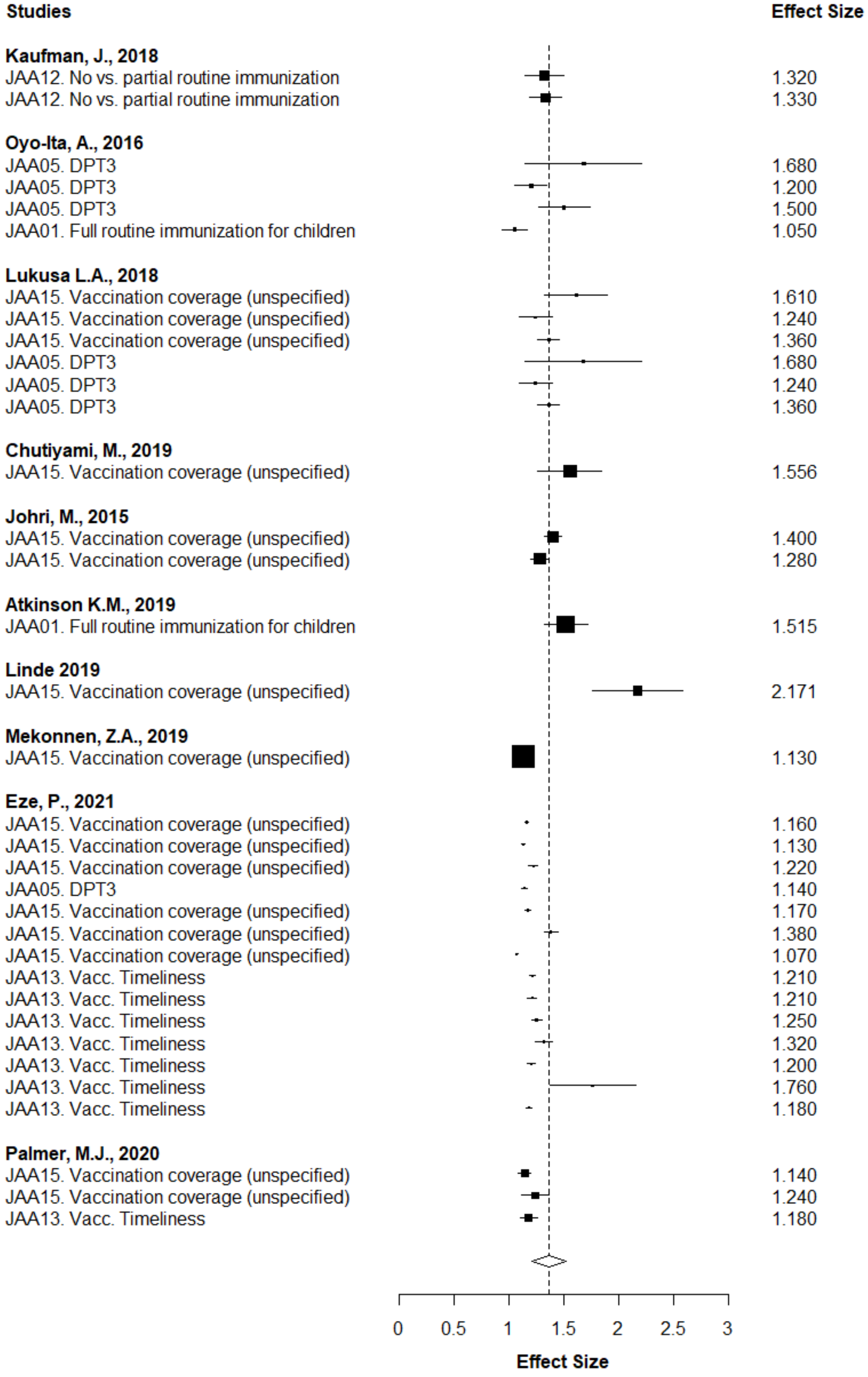

Notes: Drawing on k = 10 and 35 effect sizes.

Appendix 11: RVE analysis – 2nd tier synthesis

Figure 9.1: RVE of all interventions under Type AB, 2nd tier, mean baseline coverage values

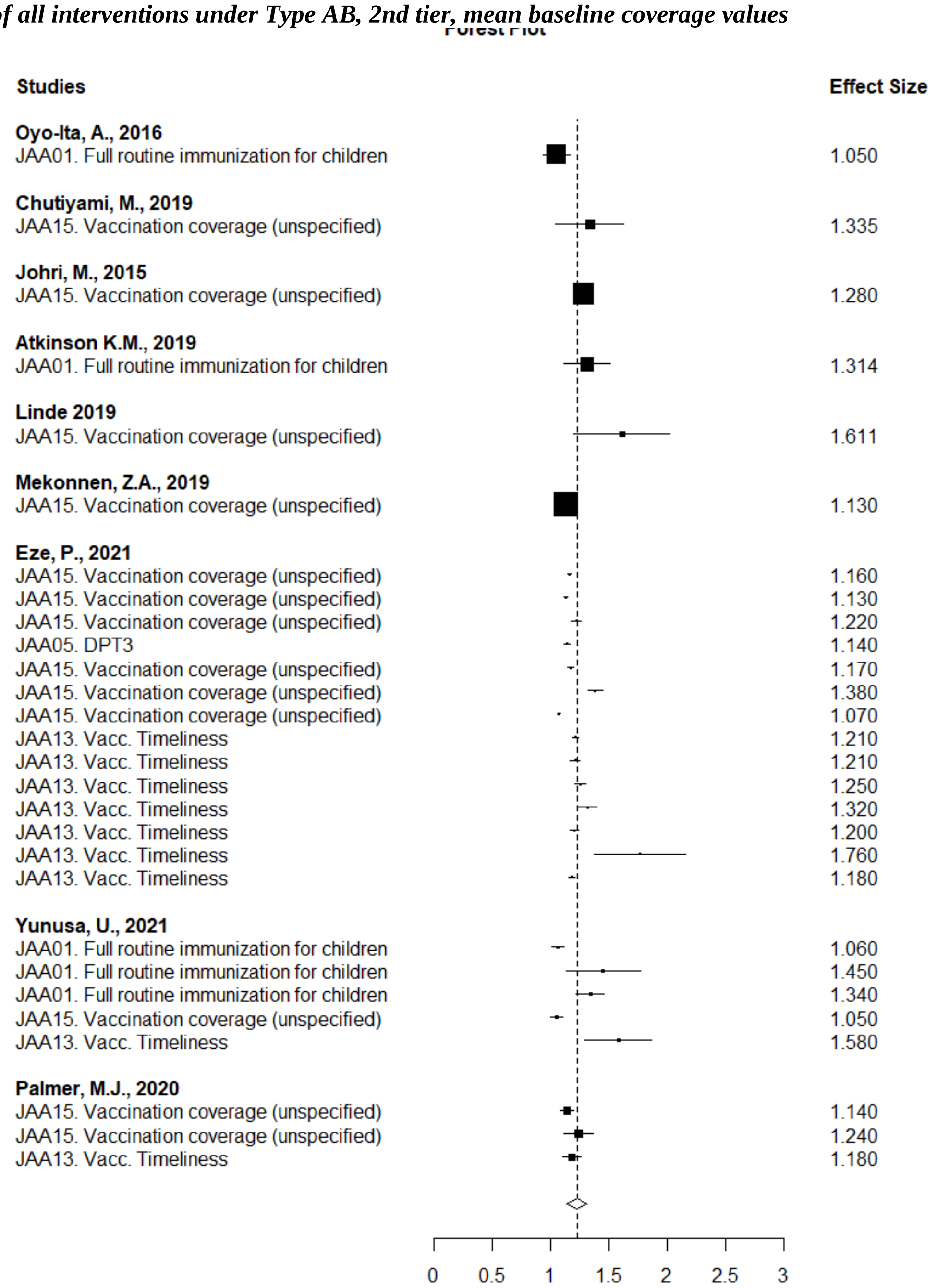

Notes: Drawing on k = 9 and 28 effect sizes.

Additional RVE analysis – 2nd tier synthesis

Figure A9.2: RVE of all interventions under Type AB, 2nd tier, one standard deviation above the mean (baseline coverage rate of 70%)

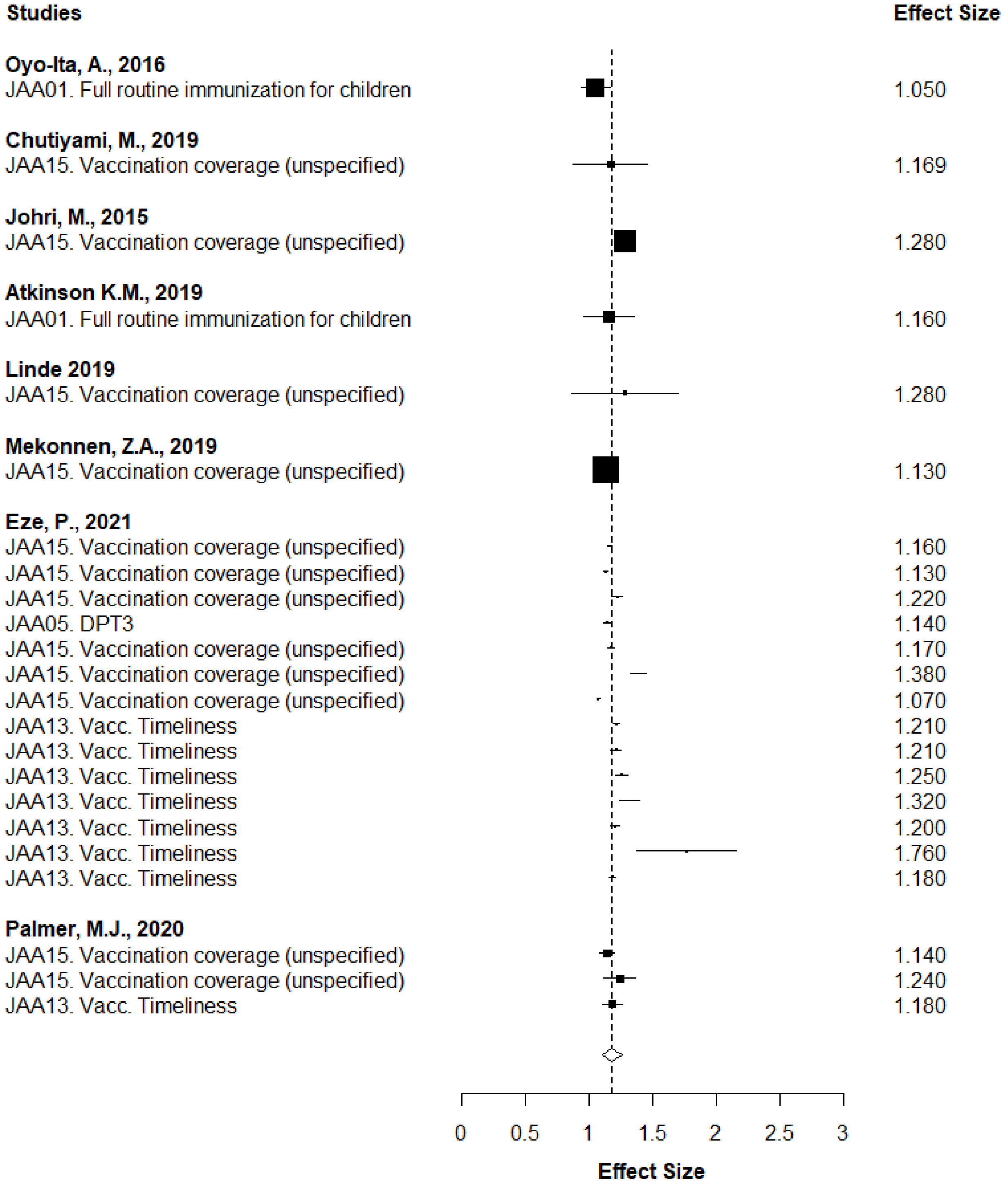

Notes: Drawing on k = 8 and 23 effect sizes.

Figure A9.3: RVE of all interventions under Type AB, 2nd tier, one standard deviation below the mean (baseline coverage rate of 26%)

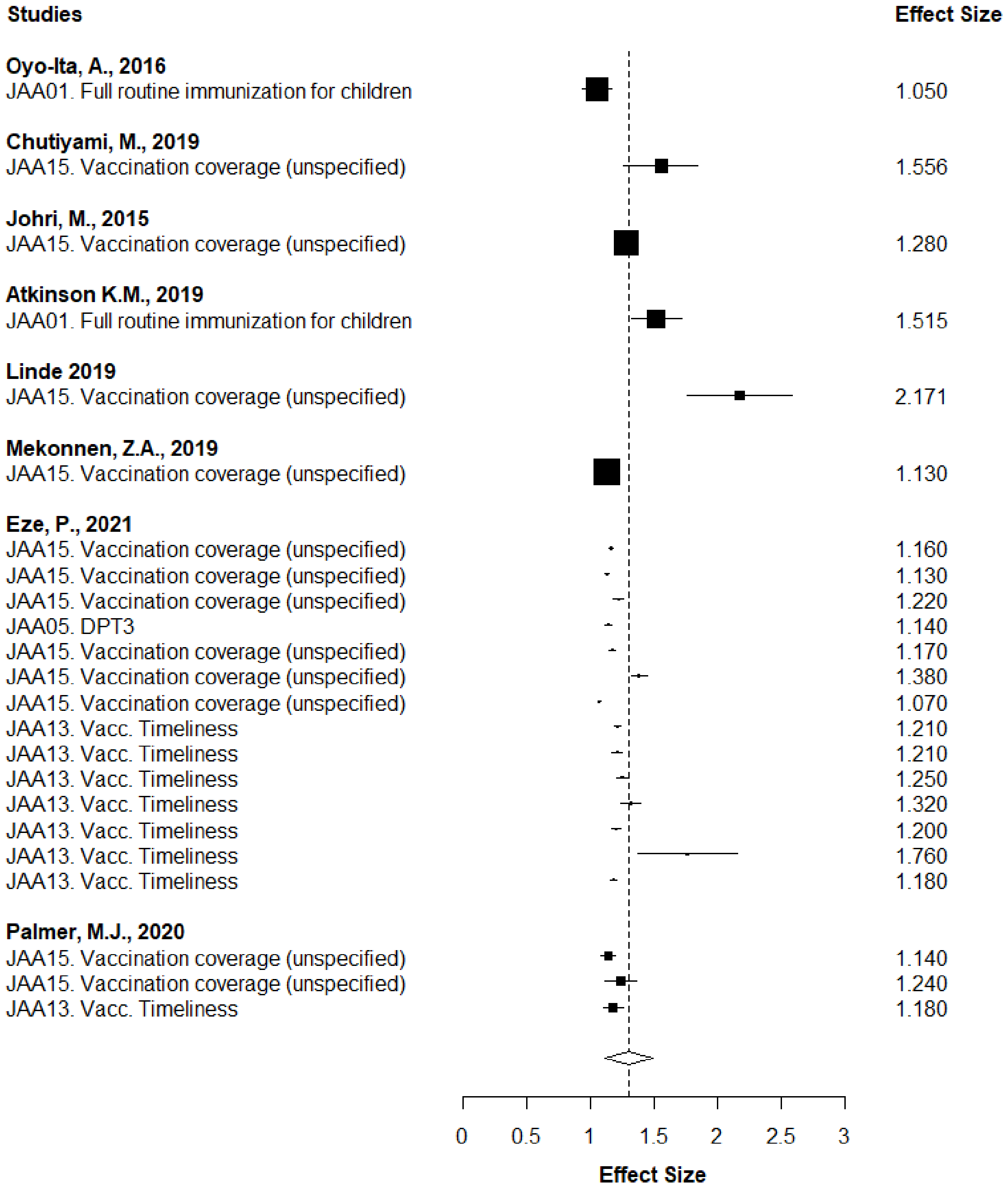

Notes: Drawing on k = 8 and 23 effect sizes.

Appendix 12: Health-systems oriented interventions

This section synthesises the high and medium confidence evidence in relation to health-systems oriented interventions (B). We have insufficient quantitative data for health-systems oriented interventions (B), hence, this section adopts a narrative approach. We begin with a summary of what we know about health-systems oriented interventions (B). The two tables below are based on 9 reviews that discuss health-systems oriented intervention categories (B), 2 of these reviews[54, 63] are captured in Table 11 above already as they are discussing complementary interventions spanning the broader categories caregiver (A) and health-systems oriented interventions (B).

Table 12 lists six reviews presenting a mixed picture in terms of intervention categories, outcomes and findings. 2 reviews each examine home visits (BB7), pay-for-performance schemes (BD5) and health system financing (BF4) in more depth. As with caregiver-oriented intervention categories (A), the evidence is based on very small study numbers. E.g., for home visits (BB7), two reviews[54, 63], find favourable effects on OPV3, measles and full routine immunisation for children. Diaconu et al.[52] and Witter et al.[67] examine pay-for-performance schemes (BD5) finding mixed results. E.g., Diaconu et al. (2021)[52] drawing on 2 to 9 studies (all low certainty) across a wide range of outcomes suggest that pay-for-performance schemes (BD5) are effective in terms of improving full routine immunisation for children, measles coverage and community health worker motivation with the remaining results being inconclusive. Witter et al. (2012)[67] only investigate the link between pay-for-performance schemes (BD5) and vaccination coverage more broadly finding inconclusive results drawing on 2 low certainty studies. Caution is required regarding over-interpreting the results of pay-for-performance schemes. A review of payment-by-results schemes for health interventions in L&MICs[109] argues that these schemes typically focus on short-term output measures such as vaccination coverage that obscure the bigger picture, e.g., the need to reform the wider health system to ensure better delivery of immunisation interventions, and not necessarily provide a long-term measure of the effectiveness of a programme. Lagarde & Palmer (2009)[57] and Odendaal et al. (2018)[62] examine health system financing (BF4) finding inconclusive results, again working with a small number of studies and across heterogenous outcomes. The high and medium confidence evidence for the remaining health-systems oriented interventions (B) is difficult to unpack systematically as we only have 1 review each for formal health worker training and education (BA1), outreach to vulnerable populations (hard-to-reach, SES, caste, etc.) (BB5) and health system strategic planning (BF1). In summary, we have no evidence on the majority of the 3rd tier health-systems oriented interventions (B) except for formal health worker training and education (BA1), promoting outreach to vaccine-hesitant groups (BB4) (in combination with national/sub-national immunisation days (BC1), see Table 13), outreach to vulnerable populations (hard-to-reach, SES, caste, etc.) (BB5), home visits (BB7), pay-for-performance schemes (BD5), health system strategic planning (BF1) and health system financing (BF4).

Table A10.1: B. Health-systems oriented interventions (all high confidence systematic reviews) – one intervention only

Systematic review	Intervention, 3rd tier level	Outcome	Finding
Oyo-Ita et al., 2016[63]	BA1. Formal health worker training and education	JAA05. DPT3; JAA09. OPV3	• DPT3: inconclusive effect, low certainty (k = 1 study) [p.16]<br>• OPV3: inconclusive effect, low certainty (k = 1 study) [p.17]
Oyo-Ita et al., 2016[63]	BB5. Outreach to vulnerable populations (hard-to-reach, SES, caste, etc.)	JAA01. Full routine immunization for children	• Full routine immunization for children: favourable effect, low certainty (k = 1 study) [p.17]
Oyo-Ita et al., 2016[63]	BB7. Home visits	JAA09. OPV3; JAA11. Measles	• OPV3: favourable effect, low certainty (k = 1 study) [p.18]<br>• Measles: favourable effect, low certainty (k = 1 study) [p.18]
Glenton et al., 2011[54]	BB7. Home visits	JAA01. Full routine immunization for children	• Full routine immunization for children: favourable effect (k = 1 study) [p.1049]

For peer review only

Diaconu et al., 2021[52]	BD5. Pay-for-performance schemes	JAA15. Vaccination coverage (unspecified); JAA01. Full routine immunisation for children; JAA02. BCG; JAA05. DPT3; JAA11. Measles; JAA09. OPV3; IAA1. Community HW motivation, capacity & performance; ICA1. Stockouts; HAD4. Experience and satisfaction with health services	• Vaccination coverage (unspecified): Inconsistent effects of small magnitude, low certainty evidence (k = 5 studies) [p. 177, 210]. Effective among poorest group for the targeted outcome (k = 2 studies), low certainty evidence, however, not for untargeted outcome, wherein it was very low certainty evidence (k = 1 study) [p.193]<br>• Full immunisation coverage: effective, low certainty evidence (k = 8 studies) [p. 178], however, for targeted measures for provider performance there was inconclusive effects, low certainty evidence (k = 3 studies) [p. 210]<br>• DPT3 coverage: Little or no effects, low certainty evidence for DPT 3 (k = 6 studies) or Pentavalent (k = 3 studies) [p. 178, 210]<br>• Measles coverage: Desirable effects, low certainty evidence (k = 6 studies) [p. 178]<br>• BCG: inconclusive (k = 9 studies) [Appendix 1, 2]<br>• OPV 3 coverage: inconclusive (k = 7 studies) [Appendix 1]<br>• Community health worker motivation, capacity and performance: favourable (k = 8 studies) [Appendix 1, 2]<br>• Stockouts: inconclusive (k = 4 studies) [Appendix 1]<br>• Experience and satisfaction with health services: favourable for overall satisfaction untargeted outcome (k = 4 studies) and inconclusive for targeted outcome (k = 2 studies) [Appendix 1]
Witter et al., 2012[67]	BD5. Pay-for-performance schemes	JAA15. Vaccination coverage (unspecified)	• Vaccination coverage (unspecified): (k = 2 studies): inconclusive, very low certainty evidence [p. 3, pp. 60-1, Table 8]
Oyo-Ita et al., 2016[63]	BF1. Health system strategic planning	JAA05. DPT3; JAA11. Measles; JAA03. DPT1; JAA02. BCG	• DPT3: favourable effect, low certainty (k = 1 study) [p.17]<br>• Measles: inconclusive effect, low certainty (k = 2 studies) [p.18]<br>• DPT 1: inconclusive effect, low certainty (k = 1 study) [p.18]<br>• BCG: inconclusive effect, low certainty (k = 1 study) [p.18]
Odendaal et al., 2018[62]	BF4. Health system financing	JAA15. Vaccination coverage (unspecified); ICA1. Stockouts	• Vaccination coverage (unspecified): little or no impact (k = 1 study), moderate certainty evidence [p.4 & p.21]<br>• Stockouts: inconclusive (k = 1 study) [refer p.22]
Lagarde & Palmer, 2009[57]	BF4. Health system financing	JAA01. Full routine immunization for children	• Full immunisation coverage: no significant impact (k = 1 study) [Table 2, p.18]

As in the case of caregiver-oriented intervention categories (A), we find a number of combinations where 3rd tier intervention categories are combined with each other. Table 13 below summarises these combinations in the context of health-systems oriented interventions (B). Table 13 is based on only 4 reviews. Gera et al. (2016)[53] and Willey et al. (2013)[66] focus on formal health worker training and education (BA1) and health system strategic planning (BF1) with Gera et al. (2016)[53] finding no evidence suggesting that DPT3 or measles coverage has improved while Willey et al. (2013)[66] find favourable evidence on vaccination (unspecified) and measles coverage. Both reviews rely on small study numbers (1-3 studies) not making any suggestions as to which one of the interventions components may have driven the results. Glenton et al. (2011)[54] looked at promoting outreach to vaccine-hesitant groups (BB4) and national/sub-national immunisation days (BC1) suggesting inconclusive effects on full routine immunisation for children and Jain et al. (2022)[41, 42] focus on outreach to vulnerable populations (hard-to-reach, SES, caste, etc.) (BB5) and collaborating with whole community (DA1) also presenting inconclusive effects. Jain et al. (2022)[41, 42] also looked at pay-for-

For peer review only

performance schemes (BD5) in combination with health system strategic planning (BF1) and collaborating with whole community (DA1), again, finding inconclusive effects drawing on small study numbers. It is not clear what we conclude from this rather heterogenous evidence base in relation to different combinations of health-systems oriented interventions (B) as the study numbers are too small, the outcomes too varied and the quality of the evidence base is questionable.

For peer review only

Table A10.2: B. Health-systems oriented interventions - more than one intervention (all high confidence systematic reviews)

Systematic review	Intervention, 3rd tier level	Outcome	Finding
Gera et al., 2016[53]	BA1. Formal health worker training and education; BF1. Health system strategic planning	JAA11. Measles; JAA05. DPT3	• DPT 3 coverage: little or no evidence of improved coverage (k = 2 studies) [p.18, Analysis 1.6]<br>• Measles coverage: little or no evidence of improved coverage, moderate certainty evidence (k = 3 studies) [p.18, Analysis 1.5
Willey et al., 2013[66]	BA1. Formal health worker training and education; BF1. Health system strategic planning	JAA15. Vaccination coverage (unspecified); JAA11. Measles;	• Vaccination coverage (unspecified): improved (k = 1 study) [p. 58, Table 3.5]<br>• Measles coverage: improved (k = 1 study) [p. 65, p.69, Table 3.6]
Glenton et al., 2011	BB4. Promoting outreach to vaccine-hesitant groups; BC1. National/sub-national immunisation days	JAA01. Full routine immunization for children	• Full routine immunization for children: inconclusive effect (k = 2 studies) [p.1050]
Jain et al., 2022[41, 42]	BB5. Outreach to vulnerable populations (hard-to-reach, SES, caste, etc.); DA1. Collaborating with whole community	HAD5. Vaccination health card availability/ retention	• Vaccination health cared availability/ retention: inconclusive (k = 4 studies) [see Supplementary Figure CECA, Appendix 8].
Jain et al., 2022[41, 42]	BD5. Pay-for-performance schemes; BF1. Health system strategic planning	IAB3. Formal HW motivation, capacity & performance	• Formal HW motivation, capacity & performance: inconclusive effect (k = 2 studies) [refer fig. 94, Appendix 8]
Jain et al., 2022[41, 42]	BD5. Pay-for-performance schemes; DA1. Collaborating with whole community	HAD4. Experience and satisfaction with health services	• Experience and satisfaction with health services: inconclusive (k = 2 studies) [see Supplementary Figure 17].

Notes: Oyo-Ita et al. (2016)[63] captures intervention combinations spanning across A. caregiver-oriented and B. health-systems oriented interventions (e.g., BB5. pay-for-performance schemes and AB1. material/ monetary incentives for caregivers; BF1. Health system strategic planning and AB1. material/ monetary incentives for caregivers), thus this study has been taken out from this table as captured in Table 11 already. Likewise, Glenton et al. (2011)[54] examining BB7. home visits And AA2. Short-term sensitization and education campaigns. This table contains studies examining more than one intervention: Two health-system oriented interventions or one health system-oriented intervention with any other intervention.

For peer review only - http://bmjopen.bmj.com/site/about/guidelines.xhtml

Appendix 13: Community-level interventions

We are combining intervention categories C., D, and F. as we have very limited evidence on these categories. As none of this evidence contains sufficient quantitative information, we adopt a narrative approach. For other community-member oriented interventions (C), we have identified no high or medium confidence studies that provide information on this intervention category. As for community-level interventions (D), Table A7 summarises the only 2 high and medium reviews we have found for this intervention category. For multicomponent interventions (F), we have identified 2 reviews.[41, 42, 55] However, only Jain et al. (2022)[41, 42] contains information on community-level interventions (as well as on health-systems oriented interventions (B), thus Jain et al (2022)[41, 42] has been included into the discussion here.

Table A11: D. Community-level interventions

Systematic review	Confidence	Intervention, 3rd tier level	Outcome	Finding
Molina et al., 2016[61]	High	DA1. Collaborating with whole community	JAA15. Vaccination coverage (unspecified)	Vaccination coverage (unspecified): significantly improved (k = 2 studies) and inconclusive (k = 1 study) [P.60, table 10, Figure 7]
Desai et al., 2020[71]	Medium	DA2. Collaborating with selected community groups and networks	JAA15. Vaccination coverage (unspecified)	Vaccination coverage (unspecified): Inconclusive (k = 2 studies) [p.4]

Let us take a closer look at community-level interventions (D), we only have 2 reviews to consider, the one by Molina et al. (2016)[61] investigates collaborating with whole community (DA1) presenting mixed results, 2 of their included studies seem to suggest significant improvements for vaccination coverage (unspecified) while 1 of their studies suggests inconclusive effects for the same outcome. Jain et al. (2022)[41, 42] have also examined collaborating with whole community (DA1) in relation to vaccination health card availability and satisfaction with health services finding inconclusive effects. The other study listed in Table A8 is the one by Desai et al. (2020)[71] which is of medium confidence and focusing on collaborating with selected community groups and networks (DA2). The outcome of interest is vaccination coverage (unspecified), and the results are inconclusive drawing on 2 studies. These results do not fill us with much confidence with regard to understanding what type of community-level interventions are effective in terms of improving immunisation outcomes. Looking at Jain et al. (2022)[41, 42] more closely, we can detect some favourable effects. They use a nuanced framework to classify single and multicomponent interventions based on the process of engaging communities, which does not fit neatly into the community-oriented categorisation of interventions used in this review. Nevertheless, drawing on 28 studies, which are a combination of demand and supply side interventions that include aspects of caregiver-oriented interventions (A) focusing on education, incentives, written messages and general community engagement, have favourable effects on full immunisation coverage. The same combination of interventions has favourable effects also on DPT3 vaccination coverage (drawing on 22 studies), on measles coverage (20 studies), on BCG (12 studies), on partial immunisation, OPV3, and immunisation knowledge (all 9 studies) as well as on vaccination timeliness of DPT3 (7 studies) and on vaccination timeliness for full immunisation, DPT2, OPV1 and 2 (all 5 studies). However, these findings confirm our previous observations, namely that the evidence base is highly heterogenous in terms of intervention categories and outcomes, it is also relying on a very limited pool of studies with varying quality. Except for caregiver-oriented interventions (A), we have an insufficient knowledge of the effectiveness of health-systems oriented interventions (B) and single community-level interventions (C and D). We found no evidence on non-health related policies and institutions (E) and have yet to understand which interventions best complement one another to enhance immunisation outcomes.

For peer review only - http://bmjopen.bmj.com/site/about/guidelines.xhtml

References

1. Asciutto R, Di Napoli A, Vecchi S, Sicuro J, Mirisola C, Petrelli A. A systematic review of economic evaluations of neonatal and maternal healthcare in immigrant and ethnic minority women. Epidemiologia e prevenzione. 2020;44(5-6 Suppl 1):142-52.
2. Balzarini F, Frascella B, Oradini-Alacreu A, Gaetti G, Lopalco PL, Edelstein M, et al. Does the use of personal electronic health records increase vaccine uptake? A systematic review. Vaccine. 2020;38(38):5966-78.
3. Diks ME, Hiligsmann M, van der Putten IM. Vaccine preferences driving vaccine-decision making of different target groups: a systematic review of choice-based experiments. BMC infectious diseases. 2021;21(1):879.
4. Haroune V, King L. Factors contributing to parental 'vaccine hesitancy' for childhood immunisations. Nursing children and young people. 2020;32(4):20-5.
5. Hutchinson AF, Smith SM. Effectiveness of strategies to increase uptake of pertussis vaccination by new parents and family caregivers: A systematic review. Midwifery. 2020;87:102734.
6. Jia L, Meng Q, Scott A, Yuan B, Zhang L. Payment methods for healthcare providers working in outpatient healthcare settings. The Cochrane database of systematic reviews. 2021;1(1):Cd011865.
7. Lambert JF, Stete K, Balmford J, Bockey A, Kern W, Rieg S, et al. Reducing burden from respiratory infections in refugees and immigrants: a systematic review of interventions in OECD, EU, EEA and EU-applicant countries. BMC infectious diseases. 2021;21(1):872.
8. Machado AA, Edwards SA, Mueller M, Saini V. Effective interventions to increase routine childhood immunization coverage in low socioeconomic status communities in developed countries: A systematic review and critical appraisal of peer-reviewed literature. Vaccine. 2021;39(22):2938-64.
9. Odone A, Dallagiacoma G, Frascella B, Signorelli C, Leask J. Current understandings of the impact of mandatory vaccination laws in Europe. Expert review of vaccines. 2021;20(5):559-75.
10. Ortiz-Sánchez E, Velando-Soriano A, Pradas-Hernández L, Vargas-Román K, Gómez-Urquiza JL, Cañadas-De la Fuente GA, et al. Analysis of the Anti-Vaccine Movement in Social Networks: A Systematic Review. International journal of environmental research and public health. 2020;17(15).
11. Pires C. What Is the State-of-the-Art in Clinical Trials on Vaccine Hesitancy 2015-2020? Vaccines. 2021;9(4).
12. Reñosa MDC, Landicho J, Wachinger J, Dalglish SL, Bärnighausen K, Bärnighausen T, et al. Nudging toward vaccination: a systematic review. BMJ Glob Health. 2021;6(9).
13. Bednarek A, Klepacz R. Vaccinology Education of Nurses and the Current Immunoprophylaxis Recommendations for Children with Juvenile Idiopathic Arthritis. Journal of clinical medicine. 2020;9(11).
14. Chenet SM, Tapia-Limonchi R. Reaching the theoretical herd immunity threshold in Iquitos, Peru: are seroprevalence data enough? The Lancet Global health. 2021;9(7):e881-e2.
15. Chitkara AJ, Pujadas Ferrer M, Forsyth K, Guiso N, Heininger U, Hozbor DF, et al. Pertussis vaccination in mixed markets: Recommendations from the Global Pertussis Initiative. International journal of infectious diseases : IJID : official publication of the International Society for Infectious Diseases. 2020;96:482-8.
16. Meteke S, Stefopulos M, Als D, Gaffey M, Kamali M, Siddiqui FJ, et al. Delivering infectious disease interventions to women and children in conflict settings: a systematic reviefw. BMJ Glob Health. 2020;5(Suppl 1).
17. Nnaji CA, Wiysonge CS, Okeibunor JC, Malinga T, Adamu AA, Tumusiime P, et al. Implementation research approaches to promoting universal health coverage in Africa: a scoping review. BMC Health Serv Res. 2021;21(1):414.
18. Chaney SC, Mechael P, Thu NM, Diallo MS, Gachen C. Every Child on the Map: A Theory of Change Framework for Improving Childhood Immunization Coverage and Equity Using Geospatial Data and Technologies. Journal of medical Internet research. 2021;23(8):e29759.

For peer review only

19. Soans S, Mihalyi A, Berlaimont V, Kolhapure S, Dash R, Agrawal A. Vaccination in preterm and low birth weight infants in India. Human vaccines & immunotherapeutics. 2022;18(1):1-12.
20. Tankwanchi AS, Jaca A, Larson HJ, Wiysonge CS, Vermund SH. Taking stock of vaccine hesitancy among migrants: a scoping review protocol. BMJ open. 2020;10(5):e035225.
21. Agrawal A, Kolhapure S, Di Pasquale A, Rai J, Mathur A. Vaccine Hesitancy as a Challenge or Vaccine Confidence as an Opportunity for Childhood Immunisation in India. Infectious diseases and therapy. 2020;9(3):421-32.
22. de Souza L, da Silva Jacob L, de Souza Lucena E, de Oliveira Costa R. Experiências brasileiras no acompanhamento de crescimento e desenvolvimento infantil no contexto da Atenção Básica. Enfermagem em Foco. 2021;12(2).
23. Berrueta M, Ciapponi A, Bardach A, Cairoli FR, Castellano FJ, Xiong X, et al. Maternal and neonatal data collection systems in low- and middle-income countries for maternal vaccines active safety surveillance systems: A scoping review. BMC pregnancy and childbirth. 2021;21(1):217.
24. Cassocera M, Chissaque A, Martins MRO, Deus N. 40 years of immunization in Mozambique: a narrative review of literature, accomplishments, and perspectives. Cadernos de saude publica. 2020;36Suppl 2(Suppl 2):e00038320.
25. Dansereau E, Brown D, Stashko L, Danovaro-Holliday MC. A systematic review of the agreement of recall, home-based records, facility records, BCG scar, and serology for ascertaining vaccination status in low and middle-income countries. Gates open research. 2019;3:923.
26. Farida Farida, Vitri Widyaningsih, Murti B. The Effect of Maternal Education and Antenatal Care on Basic Immunization Completeness in Children aged 12-23 Months in Asian and African: Meta-Analysis. Journal of Maternal and Child Health. 2020;5(6):614-28.
27. Galadima AN, Zulkefli NAM, Said SM, Ahmad N. Factors influencing childhood immunisation uptake in Africa: a systematic review. BMC public health. 2021;21(1):1475-.
28. Kalaij AGI, Sugiyanto M, Ilham AF. Factors Associated With Vaccination Compliance in Southeast Asian Children: A Systematic Review. Asia-Pacific journal of public health. 2021;33(5):479-88.
29. Kulkarni S, Thampi V, Deshmukh D, Gadhari M, Chandrasekar R, Phadke M. Trends in Urban Immunization Coverage in India: A Meta-Analysis and Meta-Regression. Indian journal of pediatrics. 2021:1-11.
30. Laserna Jiménez C, López Poyato M, Casado Montañés I, Guix-Comellas EM, Fabrellas N. Paediatric nursing clinical competences in primary healthcare: A systematic review. Journal of advanced nursing. 2021;77(6):2662-79.
31. Lassi ZS, Naseem R, Salam RA, Siddiqui F, Das JK. The Impact of the COVID-19 Pandemic on Immunization Campaigns and Programs: A Systematic Review. International journal of environmental research and public health. 2021;18(3).
32. Lieneck C, Herzog B, Krips R. Analysis of Facilitators and Barriers to the Delivery of Routine Care during the COVID-19 Global Pandemic: A Systematic Review. Healthcare (Basel, Switzerland). 2021;9(5).
33. Wariri O, Okomo U, Kwarshak YK, Murray KA, Grundy C, Kampmann B. Timeliness of routine childhood vaccination in low- and middle-income countries, 1978-2021: Protocol for a scoping review to map methodologic gaps and determinants. PloS one. 2021;16(6):e0253423.
34. Priya PK, Pathak VK, Giri AK. Vaccination coverage and vaccine hesitancy among vulnerable population of India. Human vaccines & immunotherapeutics. 2020;16(7):1502-7.
35. Smith LE, Hodson A, Rubin GJ. Parental attitudes towards mandatory vaccination; a systematic review. Vaccine. 2021;39(30):4046-53.
36. Besnier E, Thomson K, Stonkute D, Mohammad T, Akhter N, Todd A, et al. Which public health interventions are effective in reducing morbidity, mortality and health inequalities from infectious diseases amongst children in low- and middle-income countries (LMICs): An umbrella review. PloS one. 2021;16(6):e0251905.

For peer review only

37. Munk C, Portnoy A, Suharlim C, Clarke-Deelder E, Brenzel L, Resch SC, et al. Systematic review of the costs and effectiveness of interventions to increase infant vaccination coverage in low- and middle-income countries. BMC Health Services Research. 2019;19(1):741.
38. Omoniyi OS, Williams I. Realist Synthesis of the International Theory and Evidence on Strategies to Improve Childhood Vaccination in Low- and Middle-Income Countries: Developing Strategies for the Nigerian Healthcare System. International journal of health policy and management. 2020;9(7):274-85.
39. Siribaddana P, Sahay S, Hewapathirana R. Deploying Electronic Immunization Information Systems in Low and Middle Income Countries in Response to COVID-19: Systematic Review of Best Evidence Practices. SSRN; 2021.
40. Jain M, Engelbert M, Gaarder M, Bagai A, Eyers J. PROTOCOL: Use of community participation interventions to improve child immunisation in low- and middle-income countries: A systematic review and meta-analysis. Campbell Systematic Reviews. 2020;16(4):e1119.
41. Jain M, Shisler S, Lane C, Bagai A, Brown E, Engelbert M. Use of community engagement interventions to improve child immunisation in low-income and middle-income countries: a systematic review and meta-analysis. BMJ open. 2022;12(11):e061568.
42. Jain M, Shisler S, Lane C, Bagai A, Brown E, Engelbert M, et al. Use of community engagement interventions to improve child immunisation in low- and middle-income countries: A systematic review and meta-analysis. Campbell Systematic Reviews. 2022;18(3):e1253.
43. Onwuchekwa C, Verdonck K, Marchal B. Systematic Review on the Impact of Conditional Cash Transfers on Child Health Service Utilisation and Child Health in Sub-Saharan Africa. Frontiers in public health. 2021;9:643621.
44. Eze P, Lawani LO, Acharya Y. Short message service (SMS) reminders for childhood immunisation in low-income and middle-income countries: a systematic review and meta-analysis. BMJ Global Health. 2021;6(7):e005035.
45. Pantoja T, Opiyo N, Lewin S, Paulsen E, Ciapponi A, Wiysonge CS, et al. Implementation strategies for health systems in low-income countries: an overview of systematic reviews. The Cochrane database of systematic reviews. 2017;9(9):Cd011086.
46. Hatt L, Johns B, Connor C, Meline M, Kukla M, Moat K. Impact of health systems strengthening on health. Bethesda, MD: Health Finance and Governance Project, Abt Associates Inc; 2015.
47. Wiysonge S. Building evidence for improving childhood immunisation coverage in Africa: University of Cape Town; 2012.
48. Van Velthoven MH, Milne-Ives M, de Cock C, Mooney M, Meinert E. Use of Apps to Promote Childhood Vaccination: Protocol for a Systematic Review. JMIR research protocols. 2020;9(2):e16929.
49. Abiola Abdulrahman S, Olaosebikan MO. mHealth: a narrative synthesis of evidence of its application in improving childhood immunization coverage. Journal of Hospital Management and Health Policy. 2017;1(4).
50. Aaby P, Ravn H, Benn CS, Rodrigues A, Samb B, Ibrahim SA, et al. Randomized Trials Comparing Inactivated Vaccine After Medium- or High-titer Measles Vaccine With Standard Titer Measles Vaccine After Inactivated Vaccine: A Meta-analysis. The Pediatric Infectious Disease Journal. 2016;35(11).
51. Bhutta ZA, Ali S, Cousens S, Ali TM, Haider BA, Rizvi A, et al. Alma-Ata: Rebirth and Revision 6 Interventions to address maternal, newborn, and child survival: what difference can integrated primary health care strategies make? Lancet (London, England). 2008;372(9642):972-89.
52. Diaconu K, Falconer J, Verbel A, Fretheim A, Witter S. Paying for performance to improve the delivery of health interventions in low- and middle-income countries. The Cochrane database of systematic reviews. 2021;5(5):Cd007899.

For peer review only

53. Gera T, Shah D, Garner P, Richardson M, Sachdev HS. Integrated management of childhood illness (IMCI) strategy for children under five. The Cochrane database of systematic reviews. 2016(6):Cd010123.
54. Glenton C, Scheel IB, Lewin S, Swingler GH. Can lay health workers increase the uptake of childhood immunisation? Systematic review and typology. Tropical medicine & international health : TM & IH. 2011;16(9):1044-53.
55. Johri M, Pérez MC, Arsenault C, Sharma JK, Pai NP, Pahwa S, et al. Strategies to increase the demand for childhood vaccination in low- and middle-income countries: a systematic review and meta-analysis. Bulletin of the World Health Organization. 2015;93(5):339-46c.
56. Kaufman J, Ryan R, Walsh L, Horey D, Leask J, Robinson P, et al. Face-to-face interventions for informing or educating parents about early childhood vaccination. The Cochrane database of systematic reviews. 2018;5(5):Cd010038.
57. Lagarde M, Palmer N. The impact of contracting out on health outcomes and use of health services in low and middle-income countries. The Cochrane database of systematic reviews. 2009(4):Cd008133.
58. Lagarde M, Haines A, Palmer N. The impact of conditional cash transfers on health outcomes and use of health services in low and middle income countries. The Cochrane database of systematic reviews. 2009;2009(4):Cd008137.
59. Linde DS, Korsholm M, Katanga J, Rasch V, Lundh A, Andersen MS. One-way SMS and healthcare outcomes in Africa: Systematic review of randomised trials with meta-analysis. PloS one. 2019;14(6):e0217485.
60. Mekonnen ZA, Gelaye KA, Were MC, Gashu KD, Tilahun BC. Effect of mobile text message reminders on routine childhood vaccination: a systematic review and meta-analysis. Systematic reviews. 2019;8(1):154.
61. Molina E, Carella L, Pacheco A, Cruces G, Gasparini L. Community monitoring interventions to curb corruption and increase access and quality of service delivery in low- and middle-income countries: a systematic review. Campbell Systematic Reviews. 2016;12(1):1-204.
62. Odendaal WA, Ward K, Uneke J, Uro-Chukwu H, Chitama D, Balakrishna Y, et al. Contracting out to improve the use of clinical health services and health outcomes in low- and middle-income countries. The Cochrane database of systematic reviews. 2018;4(4):Cd008133.
63. Oyo-Ita A, Wiysonge CS, Oringanje C, Nwachukwu CE, Oduwole O, Meremikwu MM. Interventions for improving coverage of childhood immunisation in low- and middle-income countries. The Cochrane database of systematic reviews. 2016;7(7):Cd008145.
64. Palmer MJ, Henschke N, Bergman H, Villanueva G, Maayan N, Tamrat T, et al. Targeted client communication via mobile devices for improving maternal, neonatal, and child health. The Cochrane database of systematic reviews. 2020;8(8):Cd013679.
65. Saeterdal I, Lewin S, Austvoll-Dahlgren A, Glenton C, Munabi-Babigumira S. Interventions aimed at communities to inform and/or educate about early childhood vaccination. The Cochrane database of systematic reviews. 2014(11):Cd010232.
66. Willey B, Smith Paintain L, Mangham-Jefferies L, Car J, Armstrong Schellenberg J. Effectiveness of interventions to strengthen national health service delivery on coverage, access, quality and equity in the use of health services in low and lower middle income countries. Armstrong: EPPI-Centre, Social Science Research Unit, Institute of Education, University of London; 2013.
67. Witter S, Fretheim A, Kessy FL, Lindahl AK. Paying for performance to improve the delivery of health interventions in low- and middle-income countries The Cochrane database of systematic reviews. 2012(2):Cd007899.
68. Atkinson KM, Wilson K, Murphy MSQ, El-Halabi S, Kahale LA, Laflamme LL, et al. Effectiveness of digital technologies at improving vaccine uptake and series completion - A systematic review and meta-analysis of randomized controlled trials. Vaccine. 2019;37(23):3050-60.

For peer review only

69. Bassani DG, Arora P, Wazny K, Gaffey MF, Lenters L, Bhutta ZA. Financial incentives and coverage of child health interventions: a systematic review and meta-analysis. BMC public health. 2013;13 Suppl 3(Suppl 3):S30.
70. Chutiyami M, Wyver S, Amin J. Are Parent-Held Child Health Records a Valuable Health Intervention? A Systematic Review and Meta-Analysis. International journal of environmental research and public health. 2019;16(2).
71. Desai S, Misra M, Das A, Singh RJ, Sehgal M, Gram L, et al. Community interventions with women's groups to improve women's and children's health in India: a mixed-methods systematic review of effects, enablers and barriers. BMJ Glob Health. 2020;5(12).
72. Gilmore B, McAuliffe E. Effectiveness of community health workers delivering preventive interventions for maternal and child health in low- and middle-income countries: a systematic review. BMC public health. 2013;13:847.
73. Lukusa LA, Ndze VN, Mbeye NM, Wiysonge CS. A systematic review and meta-analysis of the effects of educating parents on the benefits and schedules of childhood vaccinations in low and middle-income countries. Human vaccines & immunotherapeutics. 2018;14(8):2058-68.
74. Baequni NY. Is Maternal and Child Health Handbook effective? : Meta-Analysis of the Effects of MCH Handbook. Kokusai Hoken Iryo (Journal of International Health). 2012;27(2):121-7.
75. Batt K, Fox-Rushby JA, Castillo-Riquelme M. The costs, effects and cost-effectiveness of strategies to increase coverage of routine immunizations in low- and middle-income countries: systematic review of the grey literature. Bulletin of the World Health Organization. 2004;82(9):689-96.
76. Bright T, Felix L, Kuper H, Polack S. A systematic review of strategies to increase access to health services among children in low and middle income countries. BMC Health Services Research. 2017;17(1):252.
77. Crocker-Buque T, Mindra G, Duncan R, Mounier-Jack S. Immunization, urbanization and slums - a systematic review of factors and interventions. BMC public health. 2017;17(1):556.
78. Das A, Gopalan SS, Chandramohan D. Effect of pay for performance to improve quality of maternal and child care in low- and middle-income countries: a systematic review. BMC public health. 2016;16:321.
79. de Cock C, van Velthoven M, Milne-Ives M, Mooney M, Meinert E. Use of Apps to Promote Childhood Vaccination: Systematic Review. JMIR mHealth and uHealth. 2020;8(5):e17371.
80. de Souza Cruz RC, Moura LBA, Soares Neto JJ. Conditional cash transfers and the creation of equal opportunities of health for children in low and middle-income countries: a literature review. International journal for equity in health. 2017;16(1):161.
81. Deardorff KV, Rubin Means A, Ásbjörnsdóttir KH, Walson J. Strategies to improve treatment coverage in community-based public health programs: A systematic review of the literature. PLoS neglected tropical diseases. 2018;12(2):e0006211.
82. Freeman PA, Schleiff M, Sacks E, Rassekh BM, Gupta S, Perry HB. Comprehensive review of the evidence regarding the effectiveness of community-based primary health care in improving maternal, neonatal and child health: 4. child health findings. Journal of global health. 2017;7(1):010904.
83. Giedion U, Andrés Alfonso E, Díaz Y. The impact of universal coverage schemes in the developing world: a review of the existing evidence. Washington DC: World Bank; 2013. Contract No.: 25.
84. Harvey H, Reissland N, Mason J. Parental reminder, recall and educational interventions to improve early childhood immunisation uptake: A systematic review and meta-analysis. Vaccine. 2015;33(25):2862-80.
85. Iwu CJ, Jaca A, Abdullahi LH, Ngcobo NJ, Wiysonge CS. A scoping review of interventions for vaccine stock management in primary health-care facilities. Human vaccines & immunotherapeutics. 2019;15(11):2666-72.

For peer review only

86. Jarrett C, Wilson R, O'Leary M, Eckersberger E, Larson HJ. Strategies for addressing vaccine hesitancy - A systematic review. Vaccine. 2015;33(34):4180-90.
87. Karageorgos G, Andreadis I, Psychas K, Mourkousis G, Kiourti A, Lazzi G, et al. The Promise of Mobile Technologies for the Health Care System in the Developing World: A Systematic Review. IEEE reviews in biomedical engineering. 2019;12:100-22.
88. Kim SS, Patel M, Hinman A. Use of m-Health in polio eradication and other immunization activities in developing countries. Vaccine. 2017;35(10):1373-9.
89. Loevinsohn B, & , Harding A. Contracting for the delivery of community health services: A review of Global Experience. Washington, DC: World Bank; 2004.
90. Mureed S, Somronghtong R, Kumar R, Ghaffar A, Chapman RS. Enhanced immunization coverage through interventions for childhood cluster diseases in developing countries. Journal of Ayub Medical College, Abbottabad : JAMC. 2015;27(1):223-7.
91. Naugle DA, Hornik RC. Systematic review of the effectiveness of mass media interventions for child survival in low- and middle-income countries. Journal of health communication. 2014;19 Suppl 1(sup1):190-215.
92. Nelson KN, Wallace AS, Sodha SV, Daniels D, Dietz V. Assessing strategies for increasing urban routine immunization coverage of childhood vaccines in low and middle-income countries: A systematic review of peer-reviewed literature. Vaccine. 2016;34(46):5495-503.
93. Nutman S, McKee D, Khoshnood K. Externalities of prevention of mother-to-child transmission programs: a systematic review. AIDS and behavior. 2013;17(2):445-60.
94. Oliver-Williams C, Brown E, Devereux S, Fairhead C, Holeman I. Using Mobile Phones to Improve Vaccination Uptake in 21 Low- and Middle-Income Countries: Systematic Review. JMIR mHealth and uHealth. 2017;5(10):e148.
95. Owusu-Addo E, Cross R. The impact of conditional cash transfers on child health in low- and middle-income countries: a systematic review. International journal of public health. 2014;59(4):609-18.
96. Ozawa S, Yemeke TT, Thompson KM. Systematic review of the incremental costs of interventions that increase immunization coverage. Vaccine. 2018;36(25):3641-9.
97. Pal M, Goodyear-Smith F, Exeter D. Systematic review of pertussis immunisation among Asians. International Journal of Human Rights in Healthcare. 2016;9(2):135-46.
98. Parrado Jiménez D. Revisión sistemática de la literatura del efecto de pago por desempeño en la calidad de la atención en países de bajos y medianos ingresos. Bogota Colombia Universidad de Los Andes; 2013.
99. Pegurri E, Fox-Rushby JA, Damian W. The effects and costs of expanding the coverage of immunisation services in developing countries: a systematic literature review. Vaccine. 2005;23(13):1624-35.
100. Pinzón Flórez C, Díaz-Quijano D, Yáñez Álvarez I, Catalina Mesa D. Effectiveness of community workers on preventive measures to maternal and child health in low and middle income countries: systematic review of the literature. Revista Salud Uninorte. 2015;31(2):309-28.
101. Rahman A, Fisher J, Bower P, Luchters S, Tran T, Yasamy MT, et al. Interventions for common perinatal mental disorders in women in low- and middle-income countries: a systematic review and meta-analysis. Bulletin of the World Health Organization. 2013;91(8):593-601i.
102. Ranganathan M, Lagarde M. Promoting healthy behaviours and improving health outcomes in low and middle income countries: a review of the impact of conditional cash transfer programmes. Preventive medicine. 2012;55 Suppl:S95-s105.
103. Ryman TK, Dietz V, Cairns KL. Too little but not too late: results of a literature review to improve routine immunization programs in developing countries. BMC Health Serv Res. 2008;8:134.
104. Shea B, Andersson N, Henry D. Increasing the demand for childhood vaccination in developing countries: a systematic review. BMC international health and human rights. 2009;9 Suppl 1(Suppl 1):S5.

For peer review only

105. Smith BL, Zizzo S, Amzel A, Wiant S, Pezzulo MC, Konopka S, et al. Integration of Neonatal and Child Health Interventions with Pediatric HIV Interventions in Global Health. International journal of MCH and AIDS. 2018;7(1):192-206.
106. Watterson JL, Walsh J, Madeka I. Using mHealth to Improve Usage of Antenatal Care, Postnatal Care, and Immunization: A Systematic Review of the Literature. BioMed research international. 2015;2015:153402.
107. Yuan B, Målqvist M, Trygg N, Qian X, Ng N, Thomsen S. What interventions are effective on reducing inequalities in maternal and child health in low- and middle-income settings? A systematic review. BMC public health. 2014;14:634.
108. Yunusa U, Garba SN, Umar AB, Idris SH, Bello UL, Abdulrashid I, et al. Mobile phone reminders for enhancing uptake, completeness and timeliness of routine childhood immunization in low and middle income countries: A systematic review and meta-analysis. Vaccine. 2021;39(2):209-21.
109. Duvendack M. Payment-by-results for health interventions in low- and middle-income countries: A critical review. Development Policy Review. 2022;40(1):e12538.

For peer review only

PRIOR Checklist

(Gates M, Gates A, Pieper D, et al. Reporting guideline for overviews of reviews of healthcare interventions: development of the PRIOR statement. *BMJ* 2022;378:e070849. doi:10.1136/bmj-2022-070849.)

**Section** Topic	#	**Item**	**Location reported**
**TITLE**			
Title	1	Identify the report as an overview of reviews.	1
**ABSTRACT**			
Abstract	2	Provide a comprehensive and accurate summary of the purpose, methods, and results of the overview of reviews.	2, 3
**INTRODUCTION**			
Rationale	3	Describe the rationale for conducting the overview of reviews in the context of existing knowledge.	4
Objectives	4	Provide an explicit statement of the objective(s) or question(s) addressed by the overview of reviews.	5
**METHODS**			
Eligibility criteria	5a	Specify the inclusion and exclusion criteria for the overview of reviews. If supplemental primary studies were included, this should be stated, with a rationale.	5, 6
	5b	Specify the definition of 'systematic review' as used in the inclusion criteria for the overview of reviews.	6
Information sources	6	Specify all databases, registers, websites, organizations, reference lists, and other sources searched or consulted to identify systematic reviews and supplemental primary studies (if included). Specify the date when each source was last searched or consulted.	6
Search strategy	7	Present the full search strategies for all databases, registers and websites, such that they could be reproduced. Describe any search filters and limits applied.	6
Selection process	8a	Describe the methods used to decide whether a systematic review or supplemental primary study (if included) met the inclusion criteria of the overview of reviews.	6
	8b	Describe how overlap in the populations, interventions, comparators, and/or outcomes of systematic reviews was identified and managed during study selection.	6, 7
Data collection process	9a	Describe the methods used to collect data from reports.	6
	9b	If applicable, describe the methods used to identify and manage primary study overlap at the level of the comparison and outcome during data collection. For each outcome, specify the method used to illustrate and/or quantify the degree of primary study overlap across systematic reviews.	6, 7
	9c	If applicable, specify the methods used to manage discrepant data across systematic reviews during data collection.	6
Data items	10	List and define all variables and outcomes for which data were sought. Describe any assumptions made and/or measures taken to identify and clarify missing or unclear information.	7, Appendix 4
Risk of bias assessment	11a	Describe the methods used to *assess* risk of bias or methodological quality of the included systematic reviews.	7
	11b	Describe the methods used to *collect* data on (from the systematic reviews) and/or *assess* the risk of bias of the primary studies included in the systematic reviews. Provide a justification for instances where flawed, incomplete, or missing assessments are identified but not re-assessed.	7
	11c	Describe the methods used to *assess* the risk of bias of supplemental primary studies (if included).	7
Synthesis methods	12a	Describe the methods used to summarize or synthesize results and provide a rationale for the choice(s).	8
	12b	Describe any methods used to explore possible causes of heterogeneity among results.	8
	12c	Describe any sensitivity analyses conducted to assess the robustness of the synthesized results.	8
Reporting bias assessment	13	Describe the methods used to *collect* data on (from the systematic reviews) and/or *assess* the risk of bias due to missing results in a summary or synthesis (arising from reporting biases at the levels of the systematic reviews, primary studies, and supplemental primary studies, if included).	7-9
Certainty assessment	14	Describe the methods used to *collect* data on (from the systematic reviews) and/or *assess* certainty (or confidence) in the body of evidence for an outcome.	9
**RESULTS**			
Systematic review and supplemental primary study selection	15a	Describe the results of the search and selection process, including the number of records screened, assessed for eligibility, and included in the overview of reviews, ideally with a flow diagram.	9
	15b	Provide a list of studies that might appear to meet the inclusion criteria, but were excluded, with the main reason for exclusion.	6, Appendix 2

For peer review only

Section Topic	#	Item	Location reported
Characteristics of systematic reviews and supplemental primary studies	16	Cite each included systematic review and supplemental primary study (if included) and present its characteristics.	9, Appendix 6
Primary study overlap	17	Describe the extent of primary study overlap across the included systematic reviews.	10
Risk of bias in systematic reviews, primary studies, and supplemental primary studies	18a	Present assessments of risk of bias or methodological quality for each included systematic review.	10
	18b	Present assessments (*collected* from systematic reviews or *assessed* anew) of the risk of bias of the primary studies included in the systematic reviews.	7
	18c	Present assessments of the risk of bias of supplemental primary studies (if included).	7
Summary or synthesis of results	19a	For all outcomes, summarize the evidence from the systematic reviews and supplemental primary studies (if included). If meta-analyses were done, present for each the summary estimate and its precision and measures of statistical heterogeneity. If comparing groups, describe the direction of the effect.	13-17
	19b	If meta-analyses were done, present results of all investigations of possible causes of heterogeneity.	8
	19c	If meta-analyses were done, present results of all sensitivity analyses conducted to assess the robustness of synthesized results.	8, 18, 19
Reporting biases	20	Present assessments (*collected* from systematic reviews and/or *assessed* anew) of the risk of bias due to missing primary studies, analyses, or results in a summary or synthesis (arising from reporting biases at the levels of the systematic reviews, primary studies, and supplemental primary studies, if included) for each summary or synthesis assessed.	7-9
Certainty of evidence	21	Present assessments (*collected* or *assessed* anew) of certainty (or confidence) in the body of evidence for each outcome.	7-9
**DISCUSSION**			
Discussion	22a	Summarize the main findings, including any discrepancies in findings across the included systematic reviews and supplemental primary studies (if included).	17, 18
	22b	Provide a general interpretation of the results in the context of other evidence.	17, 18
	22c	Discuss any limitations of the evidence from systematic reviews, their primary studies, and supplemental primary studies (if included) included in the overview of reviews. Discuss any limitations of the overview of reviews methods used.	18, 19
	22d	Discuss implications for practice, policy, and future research (both systematic reviews and primary research). Consider the relevance of the findings to the end users of the overview of reviews, e.g., healthcare providers, policymakers, patients, among others.	19, 20
**OTHER INFORMATION**			
Registration and protocol	23a	Provide registration information for the overview of reviews, including register name and registration number, or state that the overview of reviews was not registered.	22
	23b	Indicate where the overview of reviews protocol can be accessed, or state that a protocol was not prepared.	22
	23c	Describe and explain any amendments to information provided at registration or in the protocol. Indicate the stage of the overview of reviews at which amendments were made.	22
Support	24	Describe sources of financial or non-financial support for the overview of reviews, and the role of the funders or sponsors in the overview of reviews.	23
Competing interests	25	Declare any competing interests of the overview of reviews' authors.	22, 23
Author information	26a	Provide contact information for the corresponding author.	1
	26b	Describe the contributions of individual authors and identify the guarantor of the overview of reviews.	22
Availability of data and other materials	27	Report which of the following are available, where they can be found, and under which conditions they may be accessed: template data collection forms; data collected from included systematic reviews and supplemental primary studies; analytic code; any other materials used in the overview of reviews.	23

For peer review only
